# Age prediction of children and adolescents aged 6-17 years: an epigenome-wide analysis of DNA methylation

**DOI:** 10.18632/aging.101445

**Published:** 2018-05-12

**Authors:** Chunxiao Li, Wenjing Gao, Ying Gao, Canqing Yu, Jun Lv, Ruoran Lv, Jiali Duan, Ying Sun, Xianghui Guo, Weihua Cao, Liming Li

**Affiliations:** 1 Department of Epidemiology and Biostatistics, School of Public Health, Peking University, Beijing 100191, China; 2 Beijing Center for Disease Control and Prevention, Beijing 100013, China; 3 Chaoyang District Center for Disease Control and Prevention, Beijing 100021, China

**Keywords:** aging, DNA methylation, children, adolescent, prediction, Illumina Infinium MethylationEPIC Beadchip

## Abstract

The DNA methylation age, a good reflection of human aging process, has been used to predict chronological age of adults and newborns. However, the prediction model for children and adolescents was absent. In this study, we aimed to generate a prediction model of chronological age for children and adolescents aged 6-17 years by using age-specific DNA methylation patterns from 180 Chinese twin individuals. We identified 6,350 age-related CpGs from the epigenome-wide association analysis (N=179). 116 known age-related sites in children were confirmed. 83 novel CpGs were selected as predictors from all age-related loci by elastic net regression and they could accurately predict the chronological age of the pediatric population, with a correlation of 0.99 and the error of 0.23 years in the training dataset (N=90). The predictive accuracy in the testing dataset (N=89) was high (correlation=0.93, error=0.62 years). Among the 83 predictors, 49 sites were novel probes not existing on the Illumina 450K BeadChip. The top two predictors of age were on the *PRKCB* and *REG4* genes, which are associated with diabetes and cancer, respectively. Our results suggest that the chronological age can be accurately predicted among children and adolescents aged 6-17 years by 83 newly identified CpG sites.

## Introduction

Epigenetics refers to the molecular mechanisms regulating gene expression without changing the DNA sequence [1]. The mostly studied epigenetic marker is DNA methylation, the presence of methyl groups at CpG dinucleotides [2]. Previous evidence suggested that global levels of DNA methylation increased over the first few years of life [3] and then decreased in late adulthood [4,5], suggesting that epigenetic modifications might play a vital role in the human’s aging process [6,7].

A growing body of evidence confirmed the presence of age-related epigenome-wide DNA methylation patterns [8,9]. It has been shown that the methylation levels at specific age-related CpG sites represent stable and reproducible biomarkers of age. Several studies have identified age-related CpG sites in blood, but the results are inconsistent [9–16]. The age prediction model using a group of age-specific CpG sites has been widely used in adults and newborns for age prediction [14,17]. However, the age prediction model for children and adolescents using DNA methylation biomarkers was scarce [9].

It has been revealed that age-related DNA methylation changed more rapidly during childhood and adolescence. DNA methylation studies should be matched carefully to age [3,18]. It is unknown whether the accuracy and precision of age prediction model in adults would be affected when used among children and adolescents. The DNA methylation age (DNAm age) has been proved to be associated with cancer and mortality [19,20]. The accurate age prediction among children could potentially be applied to understand the development mechanism of children and to predict the risk of age-related phenotypes and diseases in adulthood. Therefore, in the present study, we aimed to develop an age prediction model for children and adolescents using DNA methylation data of over 850,000 CpG sites from the Chinese National Twin Registry.

## RESULTS

The basic characteristics of the participants are shown in Table 1. In the present study, 179 samples and 817,471 CpG sites passed quality control (QC) in the training and testing dataset (N=90 and N=89 respectively). In total, the study consisted of 101 male and 78 female singletons with an age range from 6 to 17 years (mean 10.7). The quality control results are provided in Figure S1 and Table S2 in supplements.

**Table 1:** The number, gender and zygosity distribution of subjects by age.

Age, y	No. of all	Boys, No. (%)	MZ, No. (%)
6	3	0 (0)	3 (100)
7	20	13 (65)	8 (40)
8	14	8 (57)	8 (57)
9	24	12 (50)	16 (67)
10	30	16 (53)	14 (47)
11	20	16 (80)	10 (50)
12	28	14 (50)	12 (43)
13	10	4 (40)	6 (60)
14	20	14 (70)	8 (40)
15	2	1 (50)	0 (0)
16	6	3 (50)	4 (67)
17	2	0 (0)	2 (100)
Total	179	101 (56)	91 (51)

a. Age and sex were self-reported by subjects and their parents;

b. The zygosity was determined from gene detection;

c. The mean ± SD of age:10.7±2.5 years; MZ: monozygotic twin.

### Identification of age-related DNAm sites by EWAS

**Figure 1:**
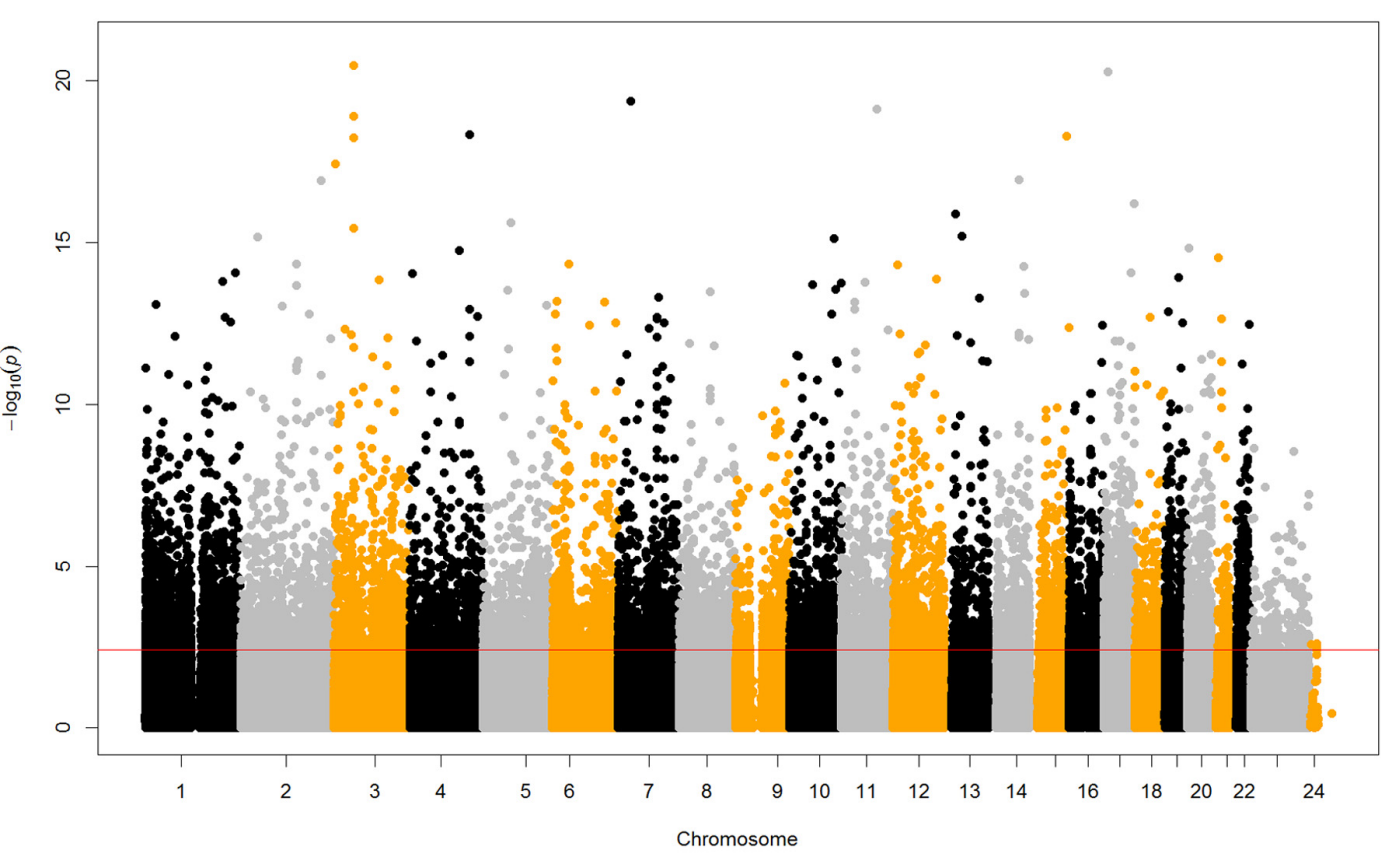
**Manhattan plot of epigenome-wide DNA methylation analysis and chronological age.** The red horizontal line indicating the P values reached the significant level of FDR < 0.05. The epigenome-wide analysis identified 6,350 CpG sites related with age.

To determine the age-related DNA methylation sites, we conducted an epigenome-wide association study (EWAS) and fitted a linear mixed-effects regression model, adjusting for sex and surrogate variables as fixed effects and family ID as a random effect. Overall, 6,350 sites of them (0.78%) were significantly related with chronological age in the EWAS (FDR < 0.05, Figure 1) and they were then selected for the subsequent prediction modeling. 116 out of the 6,350 CpG sites were confirmed given the public accessible dataset "GSE27097". It can be downloaded from the PubMed (https://www.ncbi.nlm.nih.gov/geo/query/acc.cgi?acc=GSE27097), which have focused on the age-related DNA methylation sites by Illumina 27K Beadchip among children aged 3 to 17 years old. Detailed accounts of the individual aging markers and their genomic features are presented in the Table S1 and Table S2.

### Predicting DNA methylation age in training data

In the training dataset, elastic net regression was performed, and it finally screened a set of 83 CpG sites (Table S3) predictive of age from the 6,350 age-related CpG sites. The correlation between the resulting predictor (DNAm age) and chronological age was 0.99 (*P*<2.20E-16; Figure 2a). The error (median absolute difference) of chronological age was only 0.23 years. In the sensitive analysis, we further added sex as covariate in elastic net regression model. The variable of sex was excluded from the model automatically, and the final predictive model was stable with 83 CpG sites above.

**Figure 2a:**
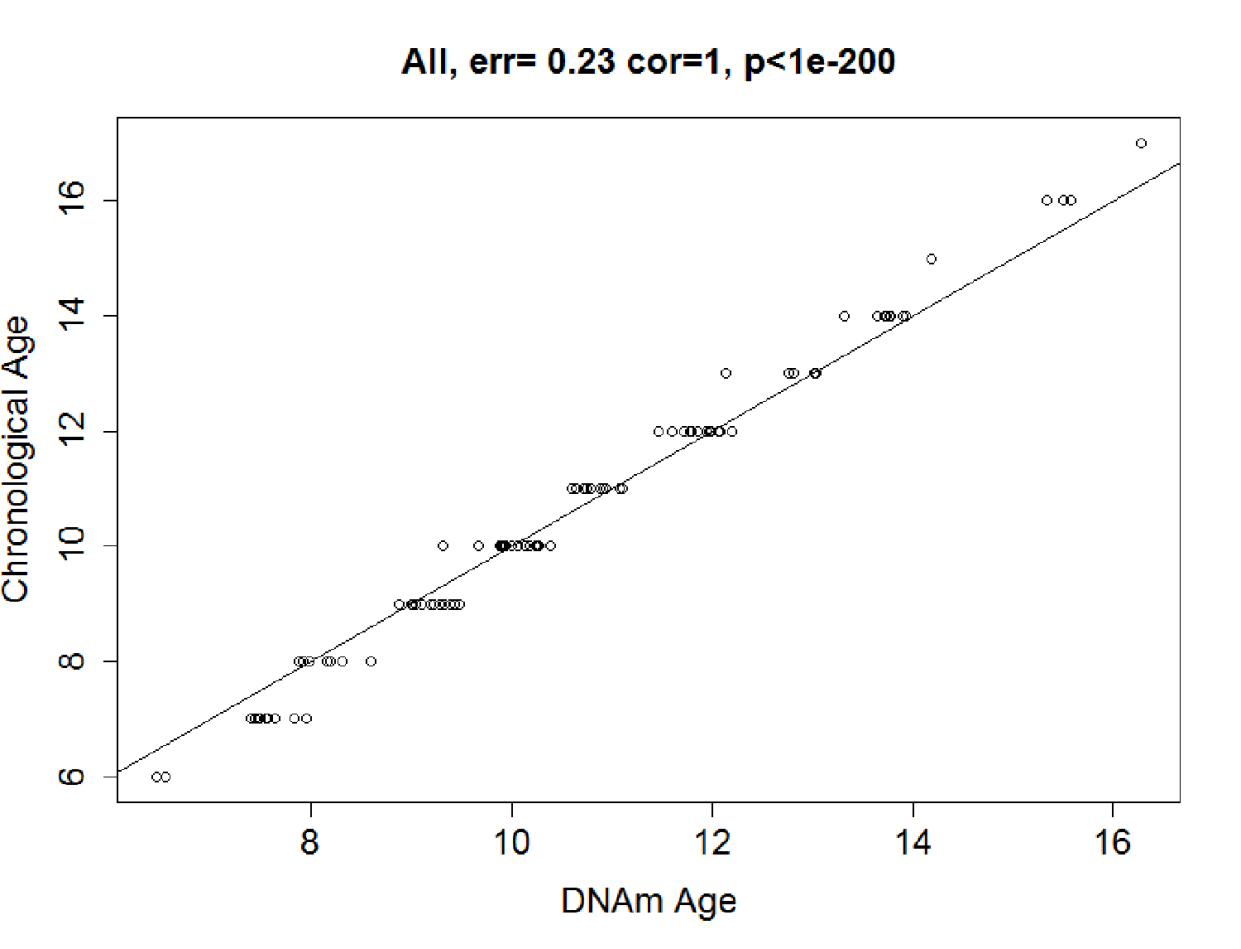
**Correlation between Chronological age and DNAm age.** In the training data, chronological age and DNAm age were highly correlated in the training dataset: r = 0.99, median error = 0.23 years.

Among the age predictive features, 21 CpG sites were positively correlated with age while 62 CpG sites were negatively correlated with age (Table S 3). Nearly half markers in the model lay within or near genes with known functions, such as diabetes, cancer, neurons function, oxidative stress, DNA damage, and other age-related conditions. 49 of the 83 age-predictive CpG sites were newly identified probes not existing on the 450K BeadChip array. The top 20 CpG sites with the largest predicted effect values were presented in Table 2. All the absolute coefficient values of the upper most 20 sites were over two, and all of them located on autosomal chromosomes. The largest coefficient effect was observed for cg00497086 (coefficient value=10.0) located in the body of the *PRKCB* (protein kinase C beta) gene on chromosome 16 and in the open sea. It was a new probe on the 850K Beadchip and related to familial Meniere's disease and diabetes.

**Table 2:** The top 20 chronological age predictive CpGs in the model.

Probename	CHR	Gene Name	Gene Group	Relation to CpG Island	Coefficient Values	Methylation β Values, means (SD)
cg00497086	16	*PRKCB*	Body	Open Sea	10.0	0.79 (0.02)
cg01231611	1	*REG4*	TSS200	Open Sea	-9.7	0.86 (0.02)
cg06072257	1	*-*	Other	Open Sea	-8.6	0.68 (0.02)
cg21242642	1	*-*	Other	Open Sea	6.2	0.16 (0.02)
cg06711259*	22	*JOSD1*	1stExon	N_Shore	-4.0	0.80 (0.02)
cg00303541*	3	*GRM2*	5'UTR	Island	3.9	0.26 (0.04)
cg03579624*	3	*-*	Other	N_Shore	3.6	0.28 (0.05)
cg04955914*	2	*CNPPD1*	Body	N_Shore	-3.5	0.58 (0.02)
cg27406001	10	*-*	Other	Open Sea	-3.5	0.57 (0.05)
cg10816468	6	*-*	Other	Open Sea	-3.1	0.64 (0.04)
cg13993467	3	*CNTN4*	Body	Open Sea	-2.9	0.64 (0.04)
cg24388008	12	*-*	Other	Open Sea	-2.7	0.10 (0.02)
cg02772754	22	*MED15*	Body	Open Sea	2.6	0.51 (0.04)
cg07219494*	5	*-*	Other	S_Shelf	-2.5	0.71 (0.06)
cg13274149*	9	*TOR4A*	3'UTR	Island	2.4	0.35 (0.05)
cg12642568	1	*CALML6*	5'UTR	N_Shelf	-2.4	0.69 (0.02)
cg13612317*	10	*KIF5B*	TSS1500	S_Shore	-2.1	0.60 (0.04)
cg07465899*	4	*-*	Other	N_Shore	-2.1	0.60 (0.02)
cg02478540	4	*-*	Other	Open Sea	-2.0	0.20 (0.02)
cg16119613*	12	*-*	Other	N_Shelf	-2.0	0.40 (0.03)

“-” means not on the known gene.

“*” means also on the Illumina 450K Beadchip.

### The predictive accuracy and validation of the model

The predictive accuracy of the model was tested in 89 co-twin singletons. In the testing dataset, we calculated the DNAm age using the 83 CpGs from the discovery stage, and found that the DNAm age was highly consistent with chronological age, with a correlation of 0.93 and an error of 0.62 years (*P*< 2.20E-16; Figure 2b).

**Figure 2b:**
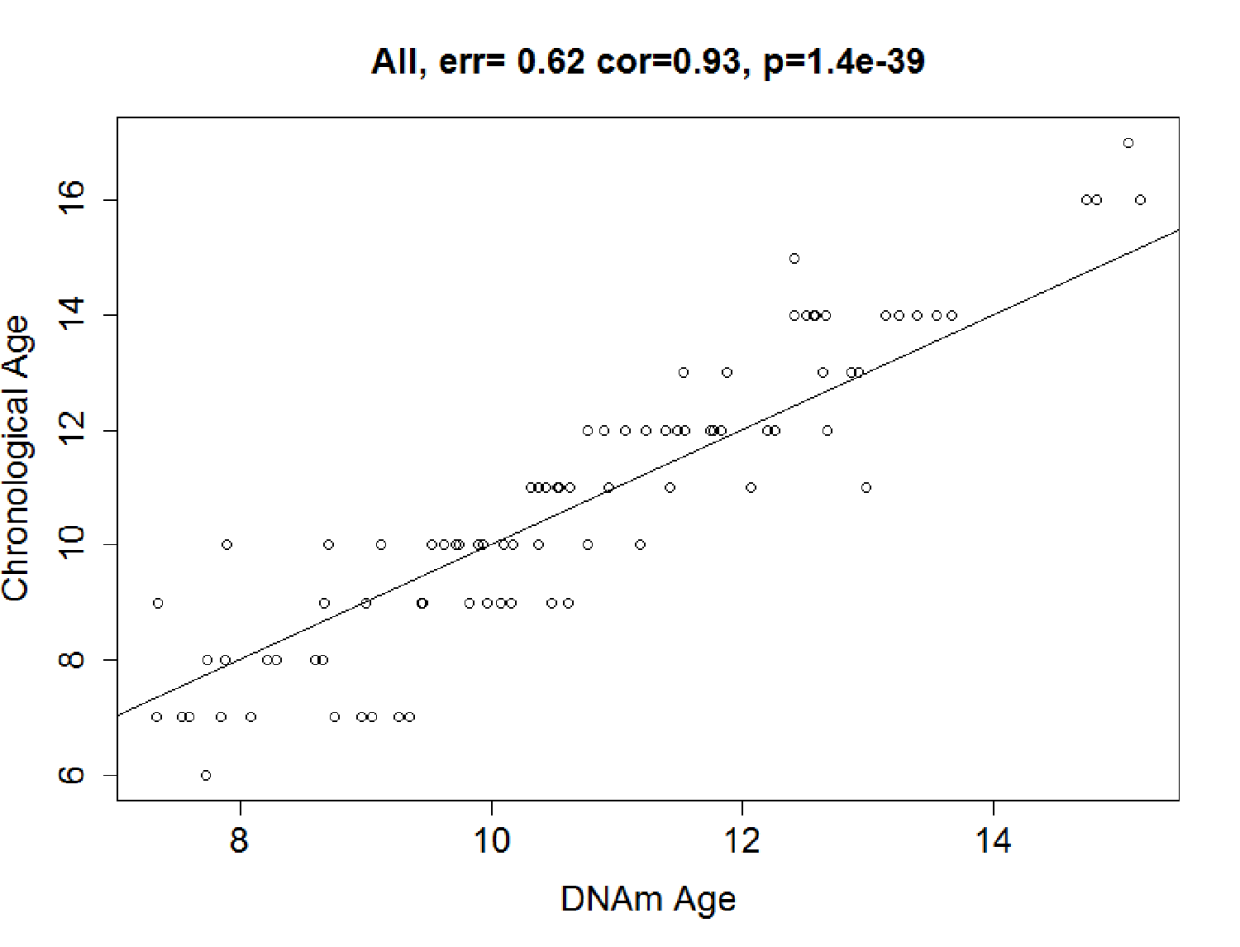
**Correlation between Chronological age and DNAm age.** DNAm age were also highly correlated with chronological age in the testing dataset: r = 0.93, median error = 0.64 years. Solid line = regression line.

We further replicated the 110 CpG sites observed in the shrunken age prediction model of Horvath [15,21]. None of them intersected with our 83 DNAm age predictors. Besides, there were only 106 predictors still remaining on the 850K Beadchip. It was unable to achieve similar predictive power using those probes which had a moderate correlation but quite high error with chronological age (correlation = 0.66, error=11.44 years, *P*<2.2E-16). We did not evaluate the Hannum predictor because some studies suggested it was less accurate than the Horvath age predictor among children [17].

### The genomic distribution of age-related CpG sites

**Figure 3:**
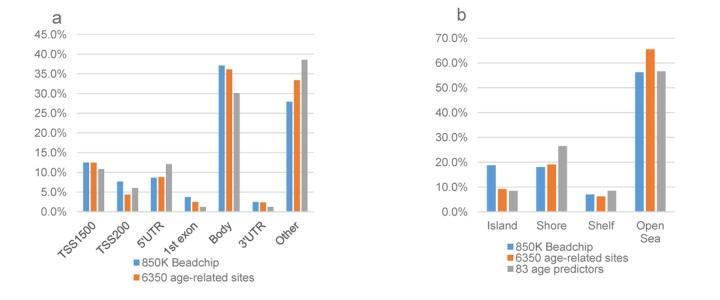
**The genomic distribution of age-associated sites compared with all 850K probes passed QC.** (**a**)The gene region distribution: frequency of age-related CpG sites according to the gene location; (**b**) The CpG islands distribution: frequency of age-related CpG sites according to the proximity to a CpG island. The ordinate represents the % CpG sites. The genomic distributions among the 83 age predictive sites, 6,350 chronological age-related CpG sites, and all the probes passed QC located on the 850 K BeadChip array were different. The annotation to be inside a CpG island was significantly over-represented on the 850k array (18.8%) compared to the 6,350 age-related CpGs and the 83 DNAm age predictors (9.2%, 8.4%), both with *P* <0.05. There was no differences in the distribution of the CpG sites with regard to other types of genomic distribution. The blue bar represents the all the probes passed QC located on the 850 K BeadChip array; the orange bar represents the 6,350 age-related CpGs; and the grey bar represents the 83 DNAm age predictors.

The comparison of genomic distribution among the 83 age-predictive features, chronological age-related CpG sites, and all the probes passed QC located on the 850K BeadChip array was shown in Figure 3. With regard to gene structure, we found both the 83 and the 6,350 sites were enriched in gene body regions as all Illumina QC probes (all over 30%), but they accounted for a smaller proportion than all QC probes (*P*_1_ =0.23, *P* _2_=0.12, Pearson’s Chi-squared test) (Figure 3a). In addition, both the age-predictive CpG sites and the chronological age-related CpG sites spread over the CpG island shores. Although CpG islands were enriched on the 850K array (18.8% of all probes are in CpG islands); only 9.2% of our 6,350 age-related CpGs and 8.4% of the 83 DNAm age predictors were located in CpG islands (both with *P* <0.05 in Pearson’s Chi-squared test) (Figure 3b). The enrichment GO terms were shown in Table S4. The biological progress included axon guidance, neuron projection guidance and neuron cell-cell adhesion (FDR>0.05).

## DISCUSSION

In this study, we identified 6,350 age-related CpG sites from the EWAS among 817,471 QC probes in 179 children (aged 6 to 17 years). In the training dataset, we finally selected 83 novel CpG sites predictive of age from all those age-related CpG sites by elastic net regression. Chronological age of the pediatric population could be accurately predicted by the DNA methylation values of the 83 CpG sites, which provided an accurate prediction of age with a correlation of 0.99 and an error of 0.23 years for the training dataset, with a robust correlation of 0.93 and an error of 0.62 years in the testing dataset.

We retrieved little literature describing the age-related DNA methylation CpG sites in children, and a proper model for pediatric age prediction was lacked [9]. Alisch et al. [18] found significant age-associated changes in DNAm at 2078 loci from 398 boys, aged 3-17 years in peripheral blood DNA, accounting for only 1/3 of our findings. 116 CpG sites were confirmed, and 83 DNAm age predictors were newly identified in the present study. However, our study did not confirm the 110 CpG sites observed in the shrunken prediction model of Horvath [15,21], suggesting that the CpG sites predictive of age in children are different from those age predictors aimed at adults since their correlations are particular to the unique developmental changes of childhood and adolescence [9].

The results represented the highest-resolution collection of DNA methylation data produced for the study of aging in children and adolescents, providing an unprecedented chance to understand the role of DNA methylation in the aging process. The accuracy of this model was similar to a study about the epigenetic clock of gestational age, in which correlations of 0.99 for the training dataset (error=0.35 weeks) and 0.91 for the testing dataset (error=1.24 weeks) were reported for 148 CpG predictors. Moreover, it seemed that the predictive power of DNA methylation was larger at younger ages. Since the prediction errors were less than three years among Horvath's subjects aged from 0 to 100 years and even much larger (error=3.88 years) among Hannum’s study participants aged from 19 to 101 years [14,15].

The age-predicted CpG sites located in genes were related to biological adhesion and cellular progress according to the gene ontology enrichment analysis, but none of them reached the significant level. The top two predictors of age (cg00497086 and cg01231611) belonged to *PRKCB* and *REG4* genes. These two genes are associated with diabetes and cancer, respectively. In fact, many studies have confirmed the correlation between DNA methylation and cancer or chronic diseases, and some of the disease-related methylation sites were associated with age [22–25]. It suggested that the DNA methylation level of critical sites might be a potential mechanism for aging and disease. However, the mechanism of association between age and methylation needs further investigations.

The mechanisms that drive DNA methylation to change with age are not well understood. Previous evidence suggests that both environmental and stochastic factors are associated with aging methylome. It is possible that environmental factors may activate cellular programs associated with changes in the epigenome over time, which at least are partly heritable through cell divisions [26,27]. The accumulation of these external exposures may contribute to DNAm change with age. It is worth noting that spontaneous changes may occur ascribe to disruption of DNA methyl groups or errors during DNA replication, leading to fundamentally unpredictable differences in the methylome [28]. These mechanisms suggest that quantitative measurements of DNAm may identify factors involved in changed rates of aging.

Several strengths of the present study merit consideration. Firstly, the identified age predictive CpG sites were specially performed from pediatric populations aged from 6 to 17 years old. Children and adolescents suffer from less confounding factors in the aspect of medication or smoking, which are more common for adults. To date, the age prediction model for children was sparse [9], and it was inappropriate to directly use the adult DNAm age predictors in childhood. To the best of our knowledge, this was the first study that uncovered new specific age-related DNA methylation sites for age prediction. Our findings improved the accuracy of the model among children whose age-related DNA methylation in blood changed more rapidly. Secondly, the present study used the Illumina 850K Beadchip which covers more DNA metylation sites than the Illumina 27K or 450K Beadchip used in previous studies. With the advance of microarray and next-generation sequencing technologies, the 850K Beadchip has the entire benefits of its predecessor (450K Beadchip) and double the amount of probes [29,30]. Thus, it provides us a more useful method to discover novel age-related DNA methylation patterns. In this study, apart from 2,686 age-related CpG sites that existed on 450K Beadchip, we newly identified over 3,000 novel loci using the 850K Beadchip. Finally yet importantly, we did this research using our first-hand data in Chinese children, instead of datasets on the open database. It was convenient for us to carry out stringent quality control for both samples and probes. Moreover, it added our information and achievement to the global DNA methylation studies and age prediction exploration.

However, there were still some limitations. Firstly, we derived DNA from blood tissues. Even though DNA methylation was known to be tissue and cell specific, it has been revealed that aging was associated with similar methylation pattern across multiple human tissues since aging was a general process affecting all cells [15]. Blood would be a more available tissue in large epidemiological researches. Several studies showed that DNA methylation measured in whole blood could be a marker for less accessible tissues that were directly involved in disease [31–33]. The second limitation was that external replication was unable to perform. Although methylation studies have increased gradually with many open data on GEO database, methylation data in children remain relatively scarce. What’s more, the data available currently was mainly based on the 27K or 450K Beadchip. Most of our identified probes via 850K Beadchip did not exist on those arrays. However, we randomly selected one singleton of a twin pair for training model and his co-twin for validation. As twins share 50%-100% of the genetic background, they can be considered replicates of similar developmental and aging trial [10]. Nevertheless, the generalizability of the results still need to be validated in more study populations with different characteristics.

In summary, our results suggest that the chronological age can be accurately predicted between 6 and 17 years old using the 83 CpG sites. As a biomarker, DNA methylation age has potential applications in research studies of development mechanism, clinical estimation, disease prediction and medicolegal expertise among pediatric population. Further studies with the 850K methylation array are required to test the generalization of this model and help deeply understand the mechanisms of human aging.

## METHODS

### Study participants and design

The data in this study were derived from the Chinese National Twin Registry (CNTR) [34]. We used whole blood samples to assess for epigenome-wide methylation from 180 school age twin individuals (44 monozygotic and 46 dizygotic twin pairs) aged from 6 to 17 years. They were recruited from Beijing, China in 2016, based on the Primary and Secondary School Health Care Center and Disease Control and Prevention Center. The chronological age was measured using the date of birth provided by the parents. In total, 179 of 180 blood samples passed the quality control and retained for the following model training and testing. Written informed consent has been obtained from all participants. The study has been conducted according to principles expressed in the Declaration of Helsinki. Biomedical Ethics Committee at Peking University, Beijing, China approved the study protocol (IRB00001052-15029).

The data were divided into two sets: one of the twins were randomly selected to the discovery group for model training, and the other one of the twins were used for replication. It could help to grasp the characteristics of the model with independent samples and to minimize the effects of data discrepancies by ensuring the similarity between the training and the testing datasets.

### Infinium MethylationEPIC BeadChip data

The DNA was extracted from fasting venous blood samples drawn by nurses in the morning (8:00 to 10:30 am). In both the discovery and replication groups, genomic DNA from whole blood was bisulfite treated using the ZYMO EZ DNA Methylation-Gold kit (ZYMO Research Corp, Irvine, CA, USA). Then DNA methylation fraction values were measured with the Illumina Infinium MethylationEPIC BeadChip (Illumina, San Diego, USA) at the EMTD Institute of Biotechnology. This procedure used bisulfate-treated DNA and two site-specific probes for each marker, which bound to the associated methylated and unmethylated sequences.

### DNA methylation quality control and processing

The raw intensity files (idat) were imported into the R software and were transformed into β values (range from 0 to 1) using R package minfi [35]. The β values were calculated from the intensity ratio of the methylated signals over the total (methylated and unmethylated) signals for each site, representing the percentage of methylation at a given cytosine for an individual across his blood cells.

Then we performed sample-level and probe-level quality control for filtering as follows. All samples passed the Illumina quality control (Figure S1 QC plot in supplements), and one sample was deleted according to the Multiple Dimension Scale (Figure S2 MDS plot in supplements). Then samples having 1% of sites with a detection p-value greater than 0.01 were removed (zero sample). Sites having 1% of samples with a detection p-value greater than 0.01 (4019 sites) or sites with beadcounts < 3 in 5% of samples (1499 sites) were removed. Additionally, since probe binding might be affected by SNPs in the binding area, sites containing SNPs or with a minor allele frequency (MAF) of at least 5% were also excluded from the data set [36]. At last, 817,471 probes passing quality control in all datasets were included.

In a further step, DASEN was applied to normalize the distribution of InflI and InfII probes together, using R package wateRmelon [37].

### Statistical analyses

#### Deriving age-related DNAm sites in epigenome-wide analysis

For the first step of modeling, we conducted epigenome-wide association scans (EWAS) to select age-related CpG sites across the EPIC (850K) array. we fitted a linear mixed-effect model [38] in R packages nlme, regressing methylation levels on the chronological age at each CpG sites of the individuals. The model adjusted for sex and surrogate variables [39] as fixed effects and family ID as a random effect to make sure the independent of twin individuals. The surrogate variable analysis (SVA) has been recommend as a stable way to correct for whole blood cellular heterogeneity in genome-wide epigenetic studies [40,41]. It can also adjust other potential confounding factors (genetic, environmental or technical) to get accurate results and can increase the study reproducibility [42].

#### Elastic net regression and data training

Then the chosen sites were kept as features for the subsequent model training of age prediction, based on the elastic net algorithm implemented in the glmnet package in R [43,44] in the training dataset (N=90). The elastic net regression is a penalized regression model that could explore a large number of CpG sites to keep the best variable set in predicting of age. In epigenetic applications, there are a lot of correlations among the CpG sites. The ridge regression can limit the coefficient size, but it usually encourages coefficients of highly correlated variables to be averaged. The lasso can make the model more interpretable but it is usually indifferent to the choice among the correlated variable sets. The elastic net regression is a combination of traditional lasso and ridge regression methods that could avoid too complex models and thus prevent over-fitting. It is ideal for building this model under conditions where the number of features greatly outweighs the number of samples, particularly for genetic data. The coefficients are also as interpretable as those in the general linear regression model [45].

In this study, the elastic net mixing parameter alpha was set to 0.5 allowing for the equal contribution of the lasso and ridge methods. The parameter lambda was chosen by a 10-fold cross-validation.We did not include extra covariates other than the methylation of age-related CpG sites in the analysis, consistent with the development of the DNAm age predictor by Horvath and Hannum [14,15].

#### Age prediction and validation

The CpG sites selected from the regression and their training coefficient values were used to fit a linear model to calculate predicted values of age, marked as DNAm age. The prediction accuracy of this model was assessed by the correlation coefficients of linear association between DNAm age and chronological age. This prediction model was subsequently validated in the test dataset of 89 samples.

#### Genomic distribution and functional classification of probes related to aging

To annotate the location of the selected DNAm age predictors or age related CpG sites, and to compare their distribution with those probes on the 850K Beadchip which passed the QC, we used the manufacturer supplied annotation data MethylationEPIC_v-1-0_B2. We also used the online software Gorilla (http://cbl-gorilla.cs.technion.ac.il/) to conduct the gene ontology enrichment analysis.

#### Sensitive analysis

As epigenetic aging rates have been suggested to be associated with sex [46], we conducted sensitive analysis by adding gender to the elastic net regression model.

To correct for multiple comparisons, a epigenome-wide significance level of the false discovery rate (FDR) < 0.05 was used and determined according to the Benjamini & Hochberg method [47].

## Supplementary Material

Supplementary Figures

Table S1

Table S2

Table S3

Table S4
