# Supplementary material for: Age prediction of children and adolescents aged 6-17 years: an epigenome-wide analysis of DNA methylation
Source: Aging (Albany NY). 2018 May 12;10(5):1015–26. doi: 10.18632/aging.101445 (PMC5990383; doi:10.18632/aging.101445)
Supplement: Supplementary Figures [file aging-10-101445-s001.pdf]

SUPPLEMENTARY MATERIAL

Please browse the Full Text version to see the data of Supplementary Tables related to this manuscript.

Table S1. 6,350 age-related CpGsites with annotation

Table S2. 116 overlapped age predictive CpG sites between GSE27097 data and this study in children and adolescents

Table S3. 83 predictive sites with annotation

Table S4. The gene ontology enrichment analysis results of 83 age predicted CpG sites ($P<10^{-3}$)

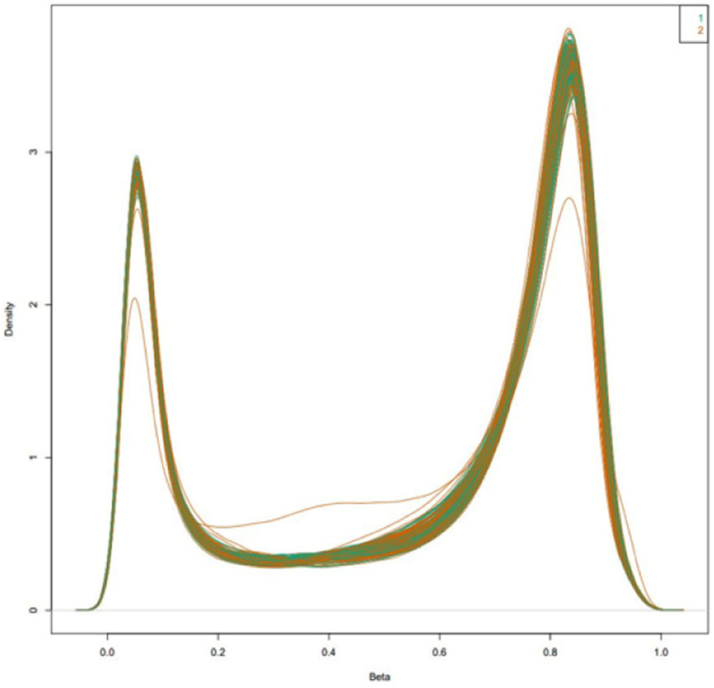

Figure S1. DNA methylation detection quality control report plot in 180 subjects.

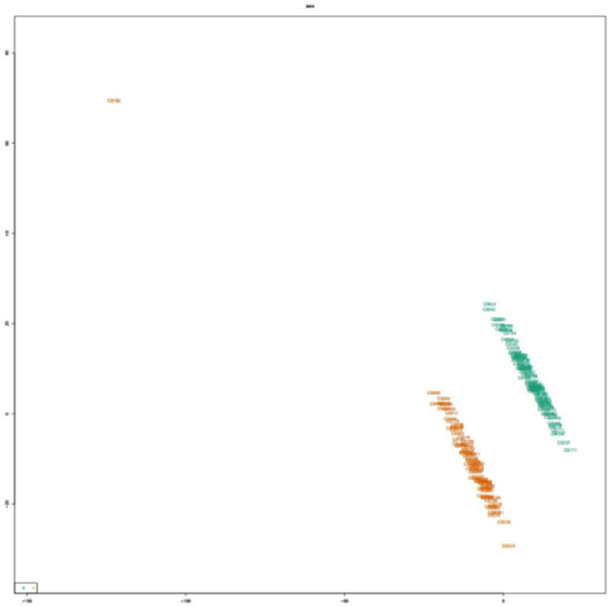

Figure S2. Multiple Dimension Scale (MDS) in 180 subjects.
