# Supplementary material for: Age prediction of children and adolescents aged 6-17 years: an epigenome-wide analysis of DNA methylation
Source: Aging (Albany NY). 2018 May 12;10(5):1015–26. doi: 10.18632/aging.101445 (PMC5990383; doi:10.18632/aging.101445)
Supplement: Table S1 [file aging-10-101445-s002.docx]

| **Table S1. 6,350 age-related sites with annotation** | | | | | | | | |
| --- | --- | --- | --- | --- | --- | --- | --- | --- |
| Rank | Probename | eAGE | FDR | CHR | Gene Name | Gene Group | Methyl 450 Loci | Relation to CpG Island |
| 1 | cg00303541 | 0.0104 | 2.15E-15 | 3 | *GRM2* | 5'UTR | TRUE | Island |
| 2 | cg13108341 | -0.0162 | 2.15E-15 | 17 | *D-H9* | Body | NA | - |
| 3 | cg26947034 | -0.0072 | 1.12E-14 | 7 | *-* | - | NA | - |
| 4 | cg03409868 | -0.0066 | 1.48E-14 | 11 | *HEPHL1* | Body | NA | - |
| 5 | cg21213853 | 0.0128 | 2.02E-14 | 3 | *GRM2* | 5'UTR | TRUE | S_Shore |
| 6 | cg16902294 | 0.0115 | 5.89E-14 | 4 | *LRAT* | TSS200 | NA | Island |
| 7 | cg18311495 | -0.0174 | 5.89E-14 | 15 | *-* | - | NA | - |
| 8 | cg26079664 | 0.0095 | 5.89E-14 | 3 | *GRM2* | TSS200 | NA | Island |
| 9 | cg13993467 | -0.0162 | 3.36E-13 | 3 | *CNTN4* | Body | NA | - |
| 10 | cg26638716 | -0.014 | 8.84E-13 | 14 | *NRXN3* | Body | NA | - |
| 11 | cg01949324 | -0.0149 | 8.84E-13 | 2 | *-* | - | NA | - |
| 12 | cg19761273 | -0.0039 | 4.28E-12 | 17 | *CSNK1D* | TSS1500 | TRUE | S_Shore |
| 13 | cg12893271 | -0.0073 | 8.07E-12 | 13 | *FLT1* | Body | NA | - |
| 14 | cg17238334 | -0.0155 | 1.44E-11 | 5 | *LOC102477328* | Body | NA | - |
| 15 | cg12934382 | 0.0138 | 1.93E-11 | 3 | *GRM2* | 1stExon | TRUE | Island |
| 16 | cg04308040 | -0.0086 | 3.15E-11 | 13 | *-* | - | TRUE | - |
| 17 | cg01124297 | -0.0077 | 3.15E-11 | 2 | *THADA* | Body | NA | - |
| 18 | cg22493216 | -0.0088 | 3.34E-11 | 10 | *PLEKHS1* | Body | NA | - |
| 19 | cg10595017 | -0.0107 | 6.37E-11 | 20 | *ADRA1D* | Body | NA | - |
| 20 | cg02773764 | -0.0097 | 7.11E-11 | 4 | *-* | - | NA | - |
| 21 | cg23204757 | -0.0278 | 1.14E-10 | 21 | *-* | - | NA | - |
| 22 | cg24488281 | -0.009 | 1.60E-10 | 2 | *ZEB2AS1* | Body | NA | S_Shelf |
| 23 | cg12542768 | -0.0034 | 1.60E-10 | 6 | *-* | - | NA | - |
| 24 | cg23256579 | -0.0099 | 1.64E-10 | 12 | *PRR4* | TSS1500 | TRUE | - |
| 25 | cg00787180 | 0.0111 | 1.73E-10 | 14 | *CCDC88C* | Body | TRUE | N_Shelf |
| 26 | cg13420364 | -0.0124 | 2.53E-10 | 1 | *-* | - | TRUE | - |
| 27 | cg16358867 | -0.0052 | 2.53E-10 | 17 | *D-I2* | Body | TRUE | - |
| 28 | cg15366841 | -0.0068 | 2.65E-10 | 4 | *-* | - | NA | S_Shelf |
| 29 | cg09310092 | -0.0074 | 3.35E-10 | 19 | *SCN1B* | Body | TRUE | N_Shelf |
| 30 | cg19848940 | -0.0083 | 3.62E-10 | 12 | *-* | - | TRUE | - |
| 31 | cg00329615 | -0.0083 | 3.62E-10 | 3 | *IGSF11* | Body | TRUE | - |
| 32 | cg13385220 | -0.0066 | 4.02E-10 | 1 | *LGR6* | Body | TRUE | - |
| 33 | cg25465557 | -0.0131 | 4.16E-10 | 11 | *AH-K* | Body | NA | - |
| 34 | cg05646745 | -0.0044 | 4.33E-10 | 10 | *FUOM* | TSS1500 | NA | S_Shore |
| 35 | cg21945120 | -0.0067 | 4.67E-10 | 10 | *-* | - | NA | - |
| 36 | cg05322294 | -0.011 | 4.67E-10 | 2 | *ZEB2AS1* | Body | NA | S_Shelf |
| 37 | cg19991948 | -0.0096 | 6.24E-10 | 10 | *TIAL1* | 3'UTR | TRUE | - |
| 38 | cg21878650 | -0.0161 | 6.24E-10 | 5 | *ADAMTS6* | Body | TRUE | - |
| 39 | cg16976808 | -0.0083 | 6.87E-10 | 8 | *TPD52* | Body | NA | - |
| 40 | cg02091781 | -0.0082 | 7.64E-10 | 14 | *-* | - | TRUE | - |
| 41 | cg12690401 | -0.011 | 9.71E-10 | 7 | *-* | - | NA | - |
| 42 | cg10244988 | -0.0093 | 1.02E-09 | 13 | *LINC00379* | Body | NA | - |
| 43 | cg16867657 | 0.0085 | 1.21E-09 | 6 | *ELOVL2* | TSS1500 | TRUE | Island |
| 44 | cg05866990 | -0.0051 | 1.28E-09 | 11 | *-* | - | NA | - |
| 45 | cg16511396 | -0.0097 | 1.28E-09 | 6 | *-* | - | NA | - |
| 46 | cg23847017 | -0.0078 | 1.47E-09 | 1 | *PHACTR4* | 1stExon | TRUE | - |
| 47 | cg23510764 | -0.0097 | 1.54E-09 | 5 | *-* | - | TRUE | - |
| 48 | cg24348981 | -0.0153 | 1.55E-09 | 2 | *-* | - | NA | - |
| 49 | cg23201812 | -0.0202 | 1.87E-09 | 11 | *-* | - | TRUE | - |
| 50 | cg08271909 | 0.0091 | 1.90E-09 | 4 | *-* | - | TRUE | Island |
| 51 | cg04295144 | 0.0155 | 2.18E-09 | 19 | *ICAM5* | Body | TRUE | Island |
| 52 | cg27406001 | -0.0103 | 2.46E-09 | 10 | *-* | - | NA | - |
| 53 | cg14807892 | -0.0072 | 2.46E-09 | 2 | *OSBPL6* | 5'UTR | NA | - |
| 54 | cg07747616 | -0.0087 | 2.48E-09 | 6 | *BMP6* | Body | TRUE | S_Shore |
| 55 | cg21322034 | -0.0084 | 2.79E-09 | 4 | *-* | - | NA | - |
| 56 | cg06269443 | -0.0061 | 2.80E-09 | 18 | *LINC00907* | Body | NA | - |
| 57 | cg10501210 | -0.0035 | 2.80E-09 | 1 | *-* | - | TRUE | - |
| 58 | cg27008901 | -0.0067 | 2.84E-09 | 7 | *TRIP6* | TSS1500 | NA | S_Shore |
| 59 | cg11575350 | -0.006 | 3.01E-09 | 21 | *-* | - | NA | - |
| 60 | cg19279257 | -0.0105 | 3.01E-09 | 7 | *SLC12A9* | Body | TRUE | N_Shore |
| 61 | cg06295292 | -0.0065 | 3.74E-09 | 1 | *CAPN2* | Body | NA | - |
| 62 | cg23565569 | -0.0112 | 3.74E-09 | 7 | *CPED1* | 5'UTR | NA | - |
| 63 | cg04689048 | 0.0043 | 3.74E-09 | 19 | *PNMAL2* | TSS200 | TRUE | Island |
| 64 | cg00988187 | -0.0061 | 3.74E-09 | 6 | *-* | - | NA | - |
| 65 | cg27166177 | -0.0042 | 4.11E-09 | 22 | *-* | - | TRUE | Island |
| 66 | cg25179877 | -0.0046 | 4.26E-09 | 16 | *-* | - | NA | - |
| 67 | cg03043157 | -0.0087 | 4.28E-09 | 6 | *FHL5* | 5'UTR | TRUE | - |
| 68 | cg09841598 | -0.0061 | 4.92E-09 | 15 | *-* | - | NA | - |
| 69 | cg19690031 | -0.0121 | 5.12E-09 | 7 | *CAC-2D1* | Body | NA | - |
| 70 | cg01654035 | -0.008 | 5.53E-09 | 3 | *-* | - | NA | - |
| 71 | cg17389813 | -0.0093 | 5.68E-09 | 11 | *LOC399959* | Body | TRUE | - |
| 72 | cg18597220 | 0.007 | 7.22E-09 | 14 | *NRXN3* | Body | TRUE | - |
| 73 | cg20843466 | -0.0117 | 7.38E-09 | 12 | *PIK3C2G* | Body | NA | - |
| 74 | cg06997537 | 0.0042 | 7.85E-09 | 3 | *CCR9* | 1stExon | NA | - |
| 75 | cg11298844 | -0.0078 | 8.06E-09 | 13 | *RXFP2* | Body | NA | - |
| 76 | cg27008177 | -0.0048 | 8.32E-09 | 1 | *USP33* | Body | NA | - |
| 77 | cg22398226 | 0.0132 | 8.32E-09 | 4 | *-* | - | TRUE | N_Shore |
| 78 | cg12967723 | -0.0086 | 8.46E-09 | 7 | *SLC12A9* | Body | TRUE | Island |
| 79 | cg11301505 | -0.0057 | 8.49E-09 | 14 | *NRXN3* | Body | NA | - |
| 80 | cg10716444 | -0.0087 | 8.75E-09 | 3 | *-* | - | NA | - |
| 81 | cg19147608 | -0.0104 | 9.42E-09 | 2 | *-* | - | NA | - |
| 82 | cg19838043 | -0.0087 | 9.55E-09 | 14 | *ZFYVE21* | Body | TRUE | S_Shore |
| 83 | cg22078805 | 0.0114 | 1.06E-08 | 17 | *FAM171A2* | Body | TRUE | Island |
| 84 | cg12318914 | -0.0075 | 1.06E-08 | 4 | *-* | - | NA | - |
| 85 | cg12857881 | -0.0073 | 1.07E-08 | 17 | *-* | - | NA | - |
| 86 | cg22029239 | -0.0182 | 1.19E-08 | 13 | *-* | - | NA | S_Shelf |
| 87 | cg21867571 | -0.0042 | 1.19E-08 | 8 | *CLU* | 5'UTR | NA | N_Shelf |
| 88 | cg09278098 | -0.0091 | 1.37E-08 | 12 | *-* | - | TRUE | - |
| 89 | cg11246419 | -0.0124 | 1.39E-08 | 8 | *-* | - | NA | - |
| 90 | cg14973055 | -0.0064 | 1.46E-08 | 17 | *D-I2* | Body | TRUE | - |
| 91 | cg00664406 | 0.0063 | 1.53E-08 | 3 | *GRM2* | TSS1500 | TRUE | Island |
| 92 | cg00213822 | -0.0161 | 1.65E-08 | 6 | *-* | - | NA | - |
| 93 | cg22724353 | -0.0096 | 1.66E-08 | 5 | *MAST4* | Body | NA | - |
| 94 | cg10891352 | -0.0063 | 2.09E-08 | 12 | *-* | - | NA | - |
| 95 | cg15208139 | -0.0084 | 2.13E-08 | 11 | *LDLRAD3* | 5'UTR | NA | - |
| 96 | cg22551157 | -0.0101 | 2.24E-08 | 12 | *HMGA2* | Body | NA | - |
| 97 | cg00589520 | -0.0071 | 2.39E-08 | 7 | *-* | - | TRUE | N_Shore |
| 98 | cg22138735 | 0.0153 | 2.39E-08 | 20 | *SOX18* | Body | TRUE | Island |
| 99 | cg23812938 | -0.0089 | 2.49E-08 | 4 | *AGPAT9* | Body | NA | - |
| 100 | cg16022195 | -0.0144 | 2.49E-08 | 10 | *-* | - | NA | S_Shore |
| 101 | cg16152753 | -0.0091 | 2.59E-08 | 10 | *-* | - | TRUE | S_Shelf |
| 102 | cg16595241 | -0.0048 | 2.76E-08 | 3 | *ADGRG7* | 1stExon | NA | - |
| 103 | cg17015290 | -0.0049 | 3.10E-08 | 20 | *KIAA1755* | 1stExon | NA | - |
| 104 | cg16193278 | -0.0035 | 3.43E-08 | 13 | *UBAC2* | Body | TRUE | - |
| 105 | cg21572722 | 0.0043 | 3.43E-08 | 6 | *ELOVL2* | TSS1500 | TRUE | Island |
| 106 | cg23453858 | -0.007 | 3.43E-08 | 2 | *-* | - | NA | - |
| 107 | cg24199558 | -0.0109 | 3.43E-08 | 10 | *-* | - | NA | - |
| 108 | cg01511232 | 0.0136 | 3.58E-08 | 4 | *-* | - | TRUE | Island |
| 109 | cg01499479 | -0.0095 | 3.63E-08 | 13 | *COL4A2* | Body | NA | - |
| 110 | cg13329407 | -0.0174 | 3.63E-08 | 21 | *-* | - | NA | - |
| 111 | cg07082267 | -0.006 | 3.76E-08 | 16 | *-* | - | TRUE | - |
| 112 | cg17983164 | -0.0062 | 3.81E-08 | 4 | *SCFD2* | Body | NA | - |
| 113 | cg14746775 | -0.0134 | 3.86E-08 | 10 | *-* | - | NA | - |
| 114 | cg21323642 | -0.0032 | 3.93E-08 | 22 | *-* | - | NA | - |
| 115 | cg26628907 | -0.006 | 4.37E-08 | 17 | *MED24* | Body | TRUE | - |
| 116 | cg18826637 | -0.0083 | 4.37E-08 | 2 | *-* | - | TRUE | - |
| 117 | cg19115041 | -0.0048 | 4.48E-08 | 3 | *ARMC8* | 5'UTR | NA | - |
| 118 | cg04413138 | -0.012 | 4.52E-08 | 1 | *LOC101928404* | TSS200 | NA | - |
| 119 | cg07168526 | -0.0068 | 4.70E-08 | 7 | *ST7* | Body | TRUE | - |
| 120 | cg04479264 | -0.0068 | 5.05E-08 | 19 | *-* | - | NA | - |
| 121 | cg12642568 | -0.0038 | 5.09E-08 | 1 | *CALML6* | 5'UTR | NA | N_Shelf |
| 122 | cg20231882 | -0.0117 | 5.37E-08 | 11 | *-* | - | NA | - |
| 123 | cg16267679 | -0.0115 | 5.96E-08 | 2 | *LINC01412* | TSS1500 | NA | S_Shelf |
| 124 | cg21020378 | -0.0084 | 6.35E-08 | 18 | *-* | - | NA | - |
| 125 | cg26305174 | -0.0099 | 6.54E-08 | 7 | *SLC12A9* | Body | TRUE | N_Shore |
| 126 | cg07914614 | -0.0084 | 7.41E-08 | 1 | *-* | - | NA | - |
| 127 | cg24442367 | -0.0079 | 7.41E-08 | 5 | *ANKRD55* | Body | NA | - |
| 128 | cg05609159 | -0.0096 | 7.81E-08 | 2 | *-* | - | TRUE | - |
| 129 | cg17812637 | -0.0071 | 9.06E-08 | 10 | *NRP1* | Body | NA | - |
| 130 | cg02231404 | 0.0184 | 9.15E-08 | 20 | *SOX18* | Body | TRUE | Island |
| 131 | cg23749518 | -0.0161 | 9.19E-08 | 12 | *-* | - | NA | - |
| 132 | cg00029246 | -0.0116 | 9.51E-08 | 7 | *DGKI* | Body | TRUE | - |
| 133 | cg18738190 | -0.0102 | 1.04E-07 | 10 | *CHST3* | 5'UTR | TRUE | - |
| 134 | cg05308819 | -0.0038 | 1.09E-07 | 1 | *-* | - | TRUE | - |
| 135 | cg07785757 | -0.0068 | 1.09E-07 | 6 | *-* | - | NA | - |
| 136 | cg09231529 | 0.007 | 1.19E-07 | 20 | *-* | - | NA | - |
| 137 | cg05498680 | -0.0105 | 1.19E-07 | 7 | *-* | - | NA | - |
| 138 | cg18618815 | -0.0057 | 1.24E-07 | 17 | *COL1A1* | Body | TRUE | N_Shore |
| 139 | cg25430089 | -0.0093 | 1.28E-07 | 9 | *PBX3* | Body | NA | - |
| 140 | cg25595813 | -0.0079 | 1.43E-07 | 18 | *DT-* | TSS1500 | NA | - |
| 141 | cg18933331 | -0.0054 | 1.43E-07 | 1 | *-* | - | TRUE | S_Shore |
| 142 | cg26090940 | -0.0057 | 1.47E-07 | 12 | *SLC16A7* | Body | TRUE | - |
| 143 | cg18647570 | -0.0101 | 1.47E-07 | 17 | *DHX8* | Body | TRUE | - |
| 144 | cg01008256 | -0.0116 | 1.52E-07 | 7 | *SLC12A9* | Body | TRUE | Island |
| 145 | cg18568497 | -0.0097 | 1.56E-07 | 12 | *SLC2A13* | Body | NA | - |
| 146 | cg03579624 | 0.0124 | 1.61E-07 | 3 | *-* | - | TRUE | N_Shore |
| 147 | cg21516752 | 0.0098 | 1.62E-07 | 18 | *-* | - | NA | - |
| 148 | cg03817394 | -0.007 | 1.83E-07 | 8 | *TPD52* | Body | NA | - |
| 149 | cg17991030 | -0.0049 | 1.90E-07 | 3 | *IL12AAS1* | Body | NA | - |
| 150 | cg04765439 | -0.0044 | 1.96E-07 | 20 | *-* | - | NA | - |
| 151 | cg19076536 | -0.0173 | 2.08E-07 | 18 | *-* | - | NA | - |
| 152 | cg02872426 | -0.018 | 2.09E-07 | 6 | *DDO* | TSS200 | TRUE | - |
| 153 | cg11387340 | -0.0099 | 2.09E-07 | 6 | *RPS6KA2* | Body | TRUE | - |
| 154 | cg09376188 | -0.008 | 2.13E-07 | 4 | *SCFD2* | Body | NA | - |
| 155 | cg03372207 | -0.0157 | 2.13E-07 | 21 | *-* | - | NA | - |
| 156 | cg04453050 | 0.0071 | 2.13E-07 | 3 | *GRM2* | TSS200 | TRUE | Island |
| 157 | cg15150970 | 0.0044 | 2.14E-07 | 2 | *DNMT3A* | Body | TRUE | N_Shore |
| 158 | cg15981571 | -0.0058 | 2.15E-07 | 17 | *-* | - | NA | - |
| 159 | cg19665390 | -0.0056 | 2.15E-07 | 10 | *PTPRE* | 5'UTR | NA | - |
| 160 | cg02481642 | -0.0067 | 2.16E-07 | 20 | *WISP2* | TSS200 | TRUE | - |
| 161 | cg04615529 | -0.007 | 2.16E-07 | 5 | *-* | - | NA | - |
| 162 | cg09294739 | -0.0073 | 2.25E-07 | 16 | *-* | - | TRUE | - |
| 163 | cg23447195 | -0.004 | 2.33E-07 | 12 | *SPRYD3* | Body | NA | - |
| 164 | cg04370442 | 0.0088 | 2.33E-07 | 16 | *TEPP* | Body | TRUE | Island |
| 165 | cg14829814 | -0.0089 | 2.41E-07 | 12 | *-* | - | TRUE | N_Shelf |
| 166 | cg03989378 | -0.0053 | 2.43E-07 | 20 | *-* | - | NA | N_Shore |
| 167 | cg05821197 | -0.0076 | 2.48E-07 | 8 | *STMN2* | Body | NA | - |
| 168 | cg25809905 | -0.0071 | 2.48E-07 | 17 | *ITGA2B* | TSS1500 | TRUE | - |
| 169 | cg13552692 | -0.0082 | 2.54E-07 | 18 | *CCDC102B* | 5'UTR | NA | - |
| 170 | cg07905908 | 0.0051 | 2.71E-07 | 4 | *LEF1* | Body | NA | - |
| 171 | cg21242642 | 0.0031 | 2.85E-07 | 1 | *-* | - | NA | - |
| 172 | cg24892069 | -0.0141 | 3.09E-07 | 10 | *NRP1* | Body | TRUE | - |
| 173 | cg02707558 | -0.006 | 3.17E-07 | 2 | *LINC01122* | Body | NA | - |
| 174 | cg17343432 | -0.0121 | 3.45E-07 | 7 | *CPED1* | 1stExon | NA | - |
| 175 | cg04837920 | -0.0131 | 3.47E-07 | 8 | *-* | - | NA | - |
| 176 | cg19996105 | -0.0092 | 3.50E-07 | 1 | *LOC440704* | Body | NA | - |
| 177 | cg06783429 | 0.0106 | 3.65E-07 | 7 | *SND1* | Body | TRUE | S_Shore |
| 178 | cg13391244 | -0.0122 | 3.65E-07 | 7 | *C7orf58* | 5'UTR | TRUE | - |
| 179 | cg00602811 | -0.0098 | 3.81E-07 | 2 | *ZEB2* | TSS1500 | TRUE | N_Shelf |
| 180 | cg22603452 | -0.007 | 3.84E-07 | 1 | *FCER1A* | 5'UTR | NA | - |
| 181 | cg17552686 | -0.0049 | 4.16E-07 | 3 | *ZBTB20AS4* | TSS1500 | NA | - |
| 182 | cg18450254 | -0.0092 | 4.21E-07 | 3 | *PRICKLE2* | 5'UTR | TRUE | - |
| 183 | cg26608718 | -0.0062 | 4.26E-07 | 19 | *AKAP8L* | TSS1500 | TRUE | S_Shore |
| 184 | cg15307593 | -0.0046 | 4.33E-07 | 7 | *PHKG1* | TSS1500 | NA | - |
| 185 | cg22273555 | -0.0044 | 4.39E-07 | 6 | *-* | - | TRUE | S_Shore |
| 186 | cg04742719 | -0.0088 | 4.51E-07 | 7 | *SLC12A9* | Body | TRUE | Island |
| 187 | cg08596835 | -0.0048 | 4.53E-07 | 12 | *-* | - | NA | - |
| 188 | cg12202498 | -0.004 | 4.53E-07 | 3 | *EAF1* | 3'UTR | NA | - |
| 189 | cg11935615 | -0.0081 | 4.70E-07 | 16 | *KIAA0430* | Body | NA | - |
| 190 | cg10986043 | -0.0044 | 4.71E-07 | 17 | *TCAP* | TSS1500 | TRUE | N_Shelf |
| 191 | cg21860825 | -0.0074 | 4.73E-07 | 12 | *PLEKHA5* | 5'UTR | TRUE | - |
| 192 | cg01097408 | -0.0044 | 4.90E-07 | 1 | *-* | - | TRUE | N_Shore |
| 193 | cg17290454 | -0.0083 | 5.08E-07 | 1 | *HHAT* | Body | NA | - |
| 194 | cg07024568 | -0.0062 | 5.33E-07 | 15 | *THSD4* | Body | NA | - |
| 195 | cg22737154 | -0.008 | 5.33E-07 | 2 | *-* | - | TRUE | - |
| 196 | cg06161779 | -0.0059 | 5.33E-07 | 21 | *-* | - | NA | - |
| 197 | cg03834031 | -0.0049 | 5.41E-07 | 22 | *-* | - | TRUE | N_Shore |
| 198 | cg09875519 | -0.0053 | 5.41E-07 | 17 | *-* | - | NA | - |
| 199 | cg27037708 | -0.0042 | 5.41E-07 | 20 | *PRNT* | TSS200 | NA | - |
| 200 | cg21406967 | -0.0044 | 5.63E-07 | 7 | *TRIP6* | TSS1500 | TRUE | S_Shore |
| 201 | cg11525710 | -0.0043 | 5.68E-07 | 1 | *-* | - | NA | - |
| 202 | cg01715572 | 0.004 | 5.70E-07 | 2 | *ARMC9* | Body | TRUE | - |
| 203 | cg17110586 | 0.0025 | 5.70E-07 | 19 | *-* | - | TRUE | S_Shelf |
| 204 | cg24833706 | 0.0073 | 6.00E-07 | 15 | *CAPN3* | TSS1500 | NA | - |
| 205 | cg26985681 | -0.0039 | 6.14E-07 | 16 | *NTAN1* | Body | TRUE | - |
| 206 | cg10370275 | -0.0081 | 6.40E-07 | 9 | *TMEM246AS1* | Body | NA | - |
| 207 | cg06570224 | 0.0028 | 6.53E-07 | 3 | *-* | - | TRUE | Island |
| 208 | cg26290219 | -0.0069 | 6.54E-07 | 6 | *-* | - | TRUE | N_Shore |
| 209 | cg25533247 | -0.0062 | 6.64E-07 | 19 | *AKAP8L* | TSS1500 | TRUE | S_Shore |
| 210 | cg03465600 | -0.0099 | 6.68E-07 | 2 | *PARD3B* | Body | NA | - |
| 211 | cg16112727 | 0.0096 | 6.68E-07 | 1 | *PEAR1* | Body | NA | - |
| 212 | cg19935040 | 0.0104 | 7.45E-07 | 17 | *FAM171A2* | Body | TRUE | Island |
| 213 | cg18555620 | -0.0048 | 7.46E-07 | 1 | *PBX1* | Body | NA | - |
| 214 | cg06094762 | -0.0083 | 7.57E-07 | 11 | *LDLRAD3* | 5'UTR | NA | - |
| 215 | cg06213060 | -0.005 | 7.57E-07 | 3 | *-* | - | TRUE | - |
| 216 | cg14836499 | -0.0074 | 7.57E-07 | 7 | *CPED1* | 5'UTR | NA | - |
| 217 | cg14255824 | -0.0063 | 8.53E-07 | 9 | *TJP2* | Body | TRUE | - |
| 218 | cg19990527 | -0.0074 | 8.53E-07 | 13 | *-* | - | NA | - |
| 219 | cg13254363 | -0.0105 | 8.61E-07 | 10 | *-* | - | NA | - |
| 220 | cg00977926 | -0.0072 | 8.78E-07 | 5 | *ARL15* | Body | NA | - |
| 221 | cg16885221 | -0.0056 | 8.79E-07 | 2 | *-* | - | NA | - |
| 222 | cg00767119 | -0.0039 | 8.86E-07 | 3 | *EAF1* | 3'UTR | NA | - |
| 223 | cg06760004 | 0.0073 | 9.53E-07 | 15 | *CAPN3* | TSS1500 | NA | - |
| 224 | cg03818930 | -0.0037 | 9.55E-07 | 6 | *-* | - | NA | - |
| 225 | cg02343594 | 0.0043 | 9.61E-07 | 6 | *APOBEC2* | Body | NA | - |
| 226 | cg20804828 | 0.0023 | 9.89E-07 | 12 | *LOC440117* | Body | NA | - |
| 227 | cg13585080 | -0.0096 | 1.03E-06 | 2 | *TEX41* | Body | NA | - |
| 228 | cg21347981 | 0.0056 | 1.03E-06 | 7 | *VWC2* | Body | NA | S_Shore |
| 229 | cg22454769 | 0.0075 | 1.03E-06 | 2 | *FHL2* | TSS200 | TRUE | Island |
| 230 | cg02265177 | -0.0035 | 1.05E-06 | 2 | *-* | - | NA | - |
| 231 | cg12058774 | 0.0053 | 1.06E-06 | 16 | *SLC6A2* | Body | NA | - |
| 232 | cg06247837 | -0.0064 | 1.07E-06 | 17 | *TCAP* | TSS1500 | TRUE | N_Shelf |
| 233 | cg25336785 | -0.0081 | 1.10E-06 | 5 | *HTR4* | TSS1500 | NA | - |
| 234 | cg03830443 | -0.0125 | 1.12E-06 | 8 | *ZFPM2* | Body | NA | S_Shore |
| 235 | cg15787636 | 0.0039 | 1.12E-06 | 17 | *TBCD* | Body | TRUE | S_Shelf |
| 236 | cg20064307 | -0.0066 | 1.13E-06 | 10 | *ADIRF* | TSS1500 | NA | N_Shore |
| 237 | cg26188729 | -0.0046 | 1.13E-06 | 7 | *-* | - | NA | - |
| 238 | cg01620970 | -0.0052 | 1.14E-06 | 4 | *-* | - | NA | - |
| 239 | cg26617011 | -0.0102 | 1.15E-06 | 7 | *-* | - | NA | - |
| 240 | cg03169270 | -0.0036 | 1.16E-06 | 2 | *-* | - | NA | - |
| 241 | cg25317548 | -0.0103 | 1.17E-06 | 2 | *SLC35F5* | Body | NA | - |
| 242 | cg27447795 | -0.0068 | 1.17E-06 | 1 | *MAST2* | Body | NA | - |
| 243 | cg09549505 | -0.0047 | 1.17E-06 | 9 | *ZNF618* | Body | NA | - |
| 244 | cg11180750 | -0.0166 | 1.18E-06 | 12 | *SLCO1B1* | TSS1500 | NA | - |
| 245 | cg13270631 | -0.0076 | 1.18E-06 | 4 | *-* | - | NA | - |
| 246 | cg16960758 | -0.0064 | 1.22E-06 | 2 | *NOSTRIN* | 5'UTR | TRUE | - |
| 247 | cg14583999 | -0.009 | 1.31E-06 | 3 | *TMEM111* | Body | TRUE | - |
| 248 | cg04000271 | -0.0079 | 1.34E-06 | 10 | *-* | - | NA | - |
| 249 | cg00818853 | -0.0054 | 1.35E-06 | 8 | *PPP2CB* | Body | NA | - |
| 250 | cg17675043 | -0.0085 | 1.38E-06 | 4 | *-* | - | NA | - |
| 251 | cg01062621 | -0.0095 | 1.40E-06 | 6 | *-* | - | NA | - |
| 252 | cg20076659 | 0.0063 | 1.43E-06 | 14 | *NRXN3* | Body | TRUE | - |
| 253 | cg21757266 | -0.0059 | 1.50E-06 | 13 | *FLT1* | Body | NA | - |
| 254 | cg22794504 | -0.0095 | 1.58E-06 | 19 | *-* | - | NA | - |
| 255 | cg24986840 | -0.0061 | 1.64E-06 | 11 | *-* | - | TRUE | - |
| 256 | cg19377250 | -0.0076 | 1.76E-06 | 7 | *SLC12A9* | Body | TRUE | N_Shore |
| 257 | cg26081717 | -0.0092 | 1.78E-06 | 9 | *TMEM246* | TSS1500 | NA | S_Shore |
| 258 | cg07201319 | -0.0069 | 1.82E-06 | 6 | *-* | - | TRUE | - |
| 259 | cg02858060 | -0.0093 | 1.83E-06 | 3 | *-* | - | NA | - |
| 260 | cg02729030 | -0.0048 | 1.83E-06 | 7 | *IMMP2L* | Body | TRUE | - |
| 261 | cg08570034 | -0.0086 | 1.83E-06 | 5 | *CPLX2* | 5'UTR | TRUE | S_Shelf |
| 262 | cg02028617 | -0.0084 | 1.84E-06 | 6 | *FARS2* | Body | NA | - |
| 263 | cg23564243 | -0.0041 | 1.89E-06 | 22 | *LOC100271722* | TSS1500 | TRUE | S_Shore |
| 264 | cg13615971 | -0.0062 | 1.89E-06 | 15 | *-* | - | TRUE | N_Shelf |
| 265 | cg20830627 | 0.0056 | 1.89E-06 | 12 | *-* | - | NA | - |
| 266 | cg04640687 | -0.0049 | 1.91E-06 | 13 | *FGF14* | Body | NA | - |
| 267 | cg25719685 | -0.0133 | 1.93E-06 | 3 | *ADGRG7* | TSS1500 | NA | - |
| 268 | cg14257429 | -0.0039 | 1.96E-06 | 9 | *-* | - | TRUE | - |
| 269 | cg05895618 | -0.0081 | 2.01E-06 | 11 | *CSRP3* | 5'UTR | TRUE | - |
| 270 | cg19419575 | -0.0045 | 2.08E-06 | 12 | *SP1* | 3'UTR | NA | - |
| 271 | cg19870470 | -0.0069 | 2.26E-06 | 10 | *MSRB2* | TSS1500 | NA | N_Shore |
| 272 | cg01176339 | -0.0087 | 2.26E-06 | 1 | *-* | - | NA | - |
| 273 | cg15434778 | 0.006 | 2.30E-06 | 14 | *NRXN3* | Body | TRUE | - |
| 274 | cg26543112 | -0.0039 | 2.35E-06 | 6 | *-* | - | TRUE | - |
| 275 | cg12634306 | 0.0106 | 2.36E-06 | 1 | *HEYL* | Body | TRUE | - |
| 276 | cg06493994 | 0.0021 | 2.39E-06 | 6 | *SCGN* | 1stExon | TRUE | Island |
| 277 | cg07653477 | -0.005 | 2.43E-06 | 22 | *SYN3* | Body | NA | - |
| 278 | cg07605566 | -0.0102 | 2.49E-06 | 9 | *TMEM246AS1* | TSS200 | NA | - |
| 279 | cg17268658 | 0.0051 | 2.49E-06 | 2 | *FHL2* | TSS200 | NA | Island |
| 280 | cg16614489 | -0.0045 | 2.52E-06 | 14 | *ABHD4* | TSS1500 | NA | N_Shore |
| 281 | cg07914804 | 0.0039 | 2.55E-06 | 5 | *GRAMD3* | TSS200 | NA | - |
| 282 | cg05694021 | -0.0104 | 2.55E-06 | 12 | *-* | - | TRUE | - |
| 283 | cg15109150 | -0.0077 | 2.55E-06 | 20 | *FAM65C* | TSS1500 | NA | - |
| 284 | cg16069986 | -0.0117 | 2.55E-06 | 11 | *SHANK2* | Body | TRUE | - |
| 285 | cg16264807 | 0.0038 | 2.56E-06 | 17 | *MTMR4* | Body | TRUE | N_Shore |
| 286 | cg07712766 | -0.0046 | 2.63E-06 | 4 | *LIMCH1* | 5'UTR | NA | - |
| 287 | cg00484358 | 0.0027 | 2.95E-06 | 1 | *ALX3* | Body | TRUE | Island |
| 288 | cg13065415 | -0.0083 | 2.98E-06 | 17 | *ATP2A3* | Body | NA | - |
| 289 | cg07191657 | -0.0049 | 3.09E-06 | 10 | *MIR1265* | TSS200 | TRUE | - |
| 290 | cg16313343 | 0.003 | 3.11E-06 | 14 | *BRF1* | TSS1500 | TRUE | S_Shore |
| 291 | cg07416237 | 0.0041 | 3.14E-06 | 13 | *CLYBL* | 3'UTR | TRUE | Island |
| 292 | cg13026729 | 0.005 | 3.14E-06 | 6 | *EZR* | TSS1500 | TRUE | Island |
| 293 | cg16392442 | -0.0035 | 3.29E-06 | 12 | *-* | - | NA | - |
| 294 | cg18215449 | -0.0064 | 3.56E-06 | 12 | *-* | - | TRUE | - |
| 295 | cg09026706 | -0.0042 | 3.63E-06 | 15 | *CTDSPL2* | 5'UTR | NA | - |
| 296 | cg02188223 | -0.0057 | 3.68E-06 | 1 | *-* | - | NA | - |
| 297 | cg03776853 | -0.003 | 3.71E-06 | 22 | *-* | - | NA | N_Shore |
| 298 | cg09911083 | 0.0033 | 3.72E-06 | 17 | *KDM6B* | Body | TRUE | Island |
| 299 | cg11693709 | -0.007 | 3.73E-06 | 15 | *PAK6* | 5'UTR | TRUE | N_Shelf |
| 300 | cg21242123 | -0.0035 | 3.73E-06 | 12 | *KRT7* | Body | NA | N_Shore |
| 301 | cg19713833 | -0.0072 | 3.90E-06 | 13 | *-* | - | NA | - |
| 302 | cg18955951 | -0.0053 | 3.94E-06 | 8 | *NRG1* | Body | NA | - |
| 303 | cg20052760 | -0.0044 | 3.96E-06 | 6 | *-* | - | TRUE | - |
| 304 | cg09612768 | -0.0044 | 4.09E-06 | 19 | *-* | - | NA | - |
| 305 | cg10305603 | -0.0075 | 4.24E-06 | 11 | *-* | - | NA | - |
| 306 | cg19796584 | 0.005 | 4.37E-06 | 17 | *-* | - | NA | N_Shelf |
| 307 | cg01424393 | -0.0082 | 4.54E-06 | 11 | *ZNF143* | Body | NA | - |
| 308 | cg21038450 | -0.0052 | 4.64E-06 | 21 | *-* | - | NA | - |
| 309 | cg22116858 | -0.0035 | 4.78E-06 | 10 | *NEURL1AS1* | Body | NA | - |
| 310 | cg25478227 | 0.0026 | 4.85E-06 | 6 | *CDKAL1* | Body | NA | - |
| 311 | cg19716264 | -0.0081 | 4.89E-06 | 1 | *EFCAB2* | Body | NA | - |
| 312 | cg10934519 | -0.0044 | 5.04E-06 | 3 | *-* | - | NA | - |
| 313 | cg01855540 | -0.0049 | 5.18E-06 | 12 | *DUSP16* | TSS1500 | NA | S_Shore |
| 314 | cg05107803 | -0.0059 | 5.27E-06 | 19 | *ZNF625* | TSS1500 | NA | S_Shore |
| 315 | cg06162038 | -0.0072 | 5.32E-06 | 2 | *HNMT* | Body | TRUE | - |
| 316 | cg09116468 | 0.0086 | 5.61E-06 | 8 | *FAM135B* | 5'UTR | NA | - |
| 317 | cg06887726 | -0.0032 | 5.68E-06 | 3 | *-* | - | NA | - |
| 318 | cg03660876 | -0.0073 | 5.76E-06 | X | *ARHGAP6* | 5'UTR | TRUE | - |
| 319 | cg11766468 | 0.0104 | 5.78E-06 | 19 | *EVI5L* | Body | TRUE | N_Shelf |
| 320 | cg13959371 | -0.0073 | 5.81E-06 | 1 | *-* | - | NA | - |
| 321 | cg00232868 | -0.008 | 5.84E-06 | 21 | *-* | - | NA | - |
| 322 | cg16381169 | -0.0045 | 6.02E-06 | 21 | *-* | - | NA | - |
| 323 | cg26121389 | -0.0073 | 6.08E-06 | 16 | *-* | - | NA | - |
| 324 | cg05218021 | -0.0041 | 6.08E-06 | 1 | *-* | - | NA | - |
| 325 | cg01943641 | -0.0058 | 6.10E-06 | 12 | *ZBTB39* | TSS1500 | TRUE | S_Shore |
| 326 | cg22796704 | -0.0077 | 6.10E-06 | 10 | *ARHGAP22* | Body | TRUE | N_Shore |
| 327 | cg20669012 | -0.0033 | 6.12E-06 | 3 | *-* | - | TRUE | - |
| 328 | cg06437747 | -0.0042 | 6.15E-06 | 1 | *-* | - | TRUE | S_Shelf |
| 329 | cg00374672 | -0.0076 | 6.18E-06 | 7 | *SLC12A9* | Body | TRUE | N_Shore |
| 330 | cg11076306 | -0.0095 | 6.18E-06 | 4 | *LIMCH1* | Body | TRUE | - |
| 331 | cg26881761 | -0.0063 | 6.18E-06 | 1 | *-* | - | TRUE | S_Shore |
| 332 | cg20168635 | -0.0033 | 6.31E-06 | 15 | *-* | - | NA | - |
| 333 | cg25571095 | 0.0059 | 6.43E-06 | 6 | *PPARD* | 5'UTR | NA | - |
| 334 | cg06064664 | 0.0072 | 6.46E-06 | 16 | *SLC6A2* | Body | NA | - |
| 335 | cg10382675 | -0.0047 | 6.47E-06 | 2 | *-* | - | NA | S_Shelf |
| 336 | cg03483944 | -0.0048 | 6.47E-06 | 19 | *FAM71E2* | TSS200 | TRUE | S_Shore |
| 337 | cg27360186 | -0.008 | 6.68E-06 | X | *-* | - | NA | - |
| 338 | cg27613273 | -0.007 | 7.04E-06 | 11 | *MAML2* | Body | NA | - |
| 339 | cg23575571 | -0.008 | 7.13E-06 | 1 | *PDE4DIP* | Body | NA | - |
| 340 | cg23731304 | -0.0047 | 7.13E-06 | 7 | *LOC541472* | Body | NA | - |
| 341 | cg01119503 | -0.01 | 7.16E-06 | 12 | *-* | - | NA | - |
| 342 | cg12041885 | -0.0029 | 7.16E-06 | 4 | *-* | - | NA | - |
| 343 | cg08355042 | 0.0088 | 7.39E-06 | 1 | *HEYL* | Body | NA | - |
| 344 | cg25865528 | -0.0034 | 7.53E-06 | 19 | *-* | - | NA | - |
| 345 | cg26015149 | -0.004 | 7.61E-06 | 1 | *-* | - | NA | - |
| 346 | cg12706260 | -0.0104 | 7.67E-06 | 4 | *ABCG2* | 5'UTR | NA | - |
| 347 | cg00135497 | 0.0084 | 7.94E-06 | 4 | *-* | - | TRUE | Island |
| 348 | cg05369942 | -0.005 | 8.05E-06 | 4 | *-* | - | TRUE | - |
| 349 | cg00941666 | -0.0035 | 8.11E-06 | 2 | *-* | - | NA | - |
| 350 | cg26544530 | -0.0058 | 8.15E-06 | 13 | *FLT1* | Body | NA | - |
| 351 | cg03905236 | -0.0064 | 8.15E-06 | 15 | *SH3GL3* | 5'UTR | NA | S_Shore |
| 352 | cg06460691 | -0.0044 | 8.18E-06 | 5 | *FAM169A* | TSS1500 | TRUE | S_Shore |
| 353 | cg09472506 | -0.0083 | 8.20E-06 | 11 | *ARHGAP42* | Body | NA | - |
| 354 | cg14112935 | 0.0121 | 8.20E-06 | 16 | *SLC6A2* | TSS200 | NA | N_Shore |
| 355 | cg09889350 | -0.0127 | 8.21E-06 | 16 | *CETP* | TSS200 | TRUE | - |
| 356 | cg13731483 | -0.0049 | 8.21E-06 | 1 | *PRDM16* | Body | NA | - |
| 357 | cg13274149 | 0.0097 | 8.26E-06 | 9 | *C9orf167* | 3'UTR | TRUE | Island |
| 358 | cg12547279 | -0.0029 | 8.43E-06 | 10 | *PCAT5* | Body | NA | - |
| 359 | cg22193385 | -0.005 | 8.46E-06 | 12 | *KRT7* | Body | TRUE | N_Shore |
| 360 | cg14488626 | -0.009 | 8.53E-06 | 5 | *SLIT3* | Body | NA | - |
| 361 | cg21761885 | -0.004 | 8.73E-06 | 9 | *-* | - | NA | - |
| 362 | cg14956327 | -0.0142 | 8.73E-06 | 6 | *DDO* | TSS1500 | TRUE | - |
| 363 | cg24276374 | -0.005 | 8.76E-06 | 2 | *-* | - | NA | - |
| 364 | cg12456505 | -0.0076 | 8.77E-06 | 12 | *-* | - | NA | - |
| 365 | cg13266245 | -0.0035 | 8.80E-06 | 11 | *CCDC34* | Body | NA | - |
| 366 | cg05042708 | -0.0045 | 8.89E-06 | 10 | *CALHM2* | TSS1500 | TRUE | S_Shore |
| 367 | cg22016779 | -0.0044 | 9.03E-06 | 2 | *DNER* | Body | TRUE | - |
| 368 | cg10303912 | 0.0051 | 9.03E-06 | 3 | *-* | - | NA | N_Shore |
| 369 | cg00170483 | -0.0043 | 9.03E-06 | 1 | *G-I3* | TSS1500 | NA | N_Shore |
| 370 | cg12465973 | -0.006 | 9.03E-06 | 1 | *ROR1* | Body | NA | - |
| 371 | cg03323911 | -0.0098 | 9.03E-06 | 1 | *EDARADD* | TSS1500 | NA | N_Shore |
| 372 | cg14900860 | 0.0036 | 9.03E-06 | 1 | *-* | - | NA | - |
| 373 | cg10628205 | -0.0055 | 9.10E-06 | 1 | *NFIA* | Body | TRUE | N_Shore |
| 374 | cg14122922 | -0.0047 | 9.10E-06 | 20 | *WISP2* | TSS1500 | NA | - |
| 375 | cg15931839 | 0.0057 | 9.10E-06 | 6 | *TRAF3IP2* | 3'UTR | TRUE | - |
| 376 | cg04387445 | -0.0082 | 9.10E-06 | 9 | *TMEM246* | TSS1500 | NA | S_Shore |
| 377 | cg07164639 | -0.0173 | 9.53E-06 | 6 | *DDO* | TSS1500 | TRUE | - |
| 378 | cg01234420 | -0.006 | 9.53E-06 | 22 | *LOC150381* | Body | TRUE | N_Shelf |
| 379 | cg10044623 | -0.0106 | 9.55E-06 | 21 | *LOC100133286* | Body | NA | - |
| 380 | cg05993870 | -0.0054 | 9.61E-06 | 4 | *-* | - | NA | - |
| 381 | cg12179916 | -0.006 | 9.61E-06 | 12 | *-* | - | NA | - |
| 382 | cg17696409 | -0.0047 | 9.61E-06 | 7 | *LOC100630923* | Body | NA | - |
| 383 | cg09726879 | -0.0055 | 9.64E-06 | 10 | *-* | - | NA | - |
| 384 | cg23753748 | -0.005 | 9.77E-06 | 10 | *CALHM2* | TSS1500 | TRUE | S_Shore |
| 385 | cg14574044 | -0.0052 | 9.98E-06 | 6 | *PDE7B* | Body | NA | - |
| 386 | cg04064963 | -0.0051 | 1.01E-05 | 6 | *SNX9* | TSS1500 | TRUE | N_Shore |
| 387 | cg17372101 | -0.0081 | 1.02E-05 | 7 | *CNT-P2* | Body | TRUE | - |
| 388 | cg17773914 | -0.0058 | 1.02E-05 | 1 | *-* | - | NA | - |
| 389 | cg04455146 | 0.0066 | 1.02E-05 | 7 | *CADPS2* | Body | NA | - |
| 390 | cg07593390 | -0.0081 | 1.03E-05 | 3 | *IL20RB* | Body | TRUE | - |
| 391 | cg13208063 | -0.0065 | 1.04E-05 | 19 | *NWD1* | TSS1500 | NA | - |
| 392 | cg15341124 | 0.0036 | 1.06E-05 | 14 | *DIO3* | 5'UTR | TRUE | Island |
| 393 | cg21165089 | 0.003 | 1.06E-05 | 11 | *C11orf85* | TSS200 | NA | Island |
| 394 | cg01557798 | 0.0054 | 1.11E-05 | 1 | *OBSCN* | Body | TRUE | Island |
| 395 | cg19791728 | -0.0036 | 1.11E-05 | 17 | *KPNB1* | Body | NA | - |
| 396 | cg26759223 | -0.0073 | 1.14E-05 | 22 | *C22orf26* | Body | TRUE | N_Shore |
| 397 | cg22266107 | -0.0074 | 1.15E-05 | 8 | *-* | - | NA | - |
| 398 | cg13930000 | -0.0034 | 1.17E-05 | 5 | *-* | - | NA | - |
| 399 | cg01933040 | 0.0073 | 1.17E-05 | 12 | *TPH2* | Body | NA | - |
| 400 | cg09183146 | 0.0053 | 1.20E-05 | 16 | *UNKL* | TSS200 | TRUE | Island |
| 401 | cg19613915 | -0.0066 | 1.20E-05 | 5 | *-* | - | TRUE | - |
| 402 | cg16312514 | -0.0112 | 1.21E-05 | 11 | *SHANK2* | Body | TRUE | - |
| 403 | cg12211691 | -0.0041 | 1.21E-05 | 1 | *ADGRB2* | Body | NA | S_Shelf |
| 404 | cg21493814 | -0.0045 | 1.25E-05 | 1 | *PBX1* | Body | NA | - |
| 405 | cg27478635 | -0.0069 | 1.26E-05 | 20 | *-* | - | NA | - |
| 406 | cg18064714 | 0.0022 | 1.28E-05 | 7 | *SP8* | Body | TRUE | Island |
| 407 | cg00804078 | -0.0116 | 1.31E-05 | 6 | *DDO* | TSS200 | TRUE | - |
| 408 | cg07951201 | -0.0043 | 1.31E-05 | 13 | *C13orf39* | Body | TRUE | - |
| 409 | cg09890339 | -0.0045 | 1.33E-05 | 12 | *CAC-1C* | Body | TRUE | - |
| 410 | cg09862081 | -0.0088 | 1.36E-05 | 15 | *SHC4* | Body | NA | - |
| 411 | cg18007249 | -0.0047 | 1.36E-05 | 15 | *UACA* | Body | NA | - |
| 412 | cg25212397 | -0.0069 | 1.40E-05 | 11 | *-* | - | NA | N_Shelf |
| 413 | cg08122706 | -0.0054 | 1.41E-05 | 3 | *-* | - | NA | - |
| 414 | cg26413501 | -0.0053 | 1.42E-05 | 6 | *-* | - | NA | - |
| 415 | cg04436528 | -0.0035 | 1.42E-05 | 8 | *BAI1* | Body | TRUE | N_Shore |
| 416 | cg05412028 | -0.0097 | 1.42E-05 | 13 | *ABCC4* | Body | TRUE | N_Shore |
| 417 | cg20060108 | -0.0076 | 1.44E-05 | 2 | *IL1RL1* | 5'UTR | TRUE | - |
| 418 | cg12557374 | -0.0038 | 1.46E-05 | 1 | *PKN2* | Body | NA | - |
| 419 | cg08262002 | -0.006 | 1.48E-05 | 4 | *LDB2* | Body | TRUE | - |
| 420 | cg13895343 | 0.0064 | 1.49E-05 | 17 | *SARM1* | Body | TRUE | Island |
| 421 | cg14193434 | -0.0084 | 1.49E-05 | 13 | *PCDH9* | 5'UTR | NA | N_Shore |
| 422 | cg07408456 | -0.006 | 1.52E-05 | 19 | *PGLYRP2* | TSS1500 | TRUE | - |
| 423 | cg14517133 | -0.0092 | 1.53E-05 | 6 | *PRPH2* | 3'UTR | TRUE | - |
| 424 | cg04304450 | 0.0051 | 1.53E-05 | 22 | *BIK* | 3'UTR | TRUE | - |
| 425 | cg21780506 | -0.0063 | 1.57E-05 | 10 | *-* | - | NA | - |
| 426 | cg07504615 | -0.0028 | 1.62E-05 | 3 | *-* | - | NA | - |
| 427 | cg07259219 | -0.0121 | 1.62E-05 | 5 | *-* | - | NA | - |
| 428 | cg25164623 | -0.0062 | 1.62E-05 | 12 | *-* | - | NA | - |
| 429 | cg08744915 | 0.0042 | 1.62E-05 | 12 | *KDM2B* | Body | NA | - |
| 430 | cg03409300 | -0.0044 | 1.65E-05 | 8 | *-* | - | NA | - |
| 431 | cg15831217 | -0.0077 | 1.69E-05 | 14 | *NRXN3* | Body | NA | - |
| 432 | cg25086144 | -0.0085 | 1.71E-05 | 8 | *FAM110B* | 5'UTR | NA | - |
| 433 | cg00956142 | -0.0067 | 1.75E-05 | 1 | *PHACTR4* | Body | TRUE | - |
| 434 | cg22460123 | -0.0042 | 1.78E-05 | 12 | *KRT7* | Body | TRUE | N_Shore |
| 435 | cg14305526 | -0.015 | 1.84E-05 | 11 | *-* | - | NA | - |
| 436 | cg23696863 | -0.0076 | 1.86E-05 | 5 | *FBN2* | Body | NA | - |
| 437 | cg11207533 | 0.0101 | 1.87E-05 | 17 | *HOXBAS3* | Body | NA | S_Shelf |
| 438 | cg24328774 | -0.0029 | 1.87E-05 | 6 | *-* | - | TRUE | - |
| 439 | cg02478540 | -0.0041 | 1.90E-05 | 4 | *-* | - | NA | - |
| 440 | cg12841266 | 0.0027 | 1.92E-05 | 3 | *LHFPL4* | Body | NA | Island |
| 441 | cg09462203 | -0.0047 | 1.93E-05 | 22 | *-* | - | NA | - |
| 442 | cg05403444 | -0.0047 | 1.93E-05 | 1 | *-* | - | NA | - |
| 443 | cg14177623 | -0.0066 | 1.93E-05 | 3 | *NLGN1AS1* | Body | NA | - |
| 444 | cg25851044 | -0.0044 | 1.93E-05 | 3 | *-* | - | NA | - |
| 445 | cg00448875 | -0.0031 | 1.93E-05 | 3 | *RAB6B* | Body | TRUE | N_Shore |
| 446 | cg01761228 | -0.0047 | 1.93E-05 | 2 | *DPP10* | Body | NA | N_Shelf |
| 447 | cg07715777 | -0.0042 | 1.93E-05 | 6 | *-* | - | TRUE | N_Shore |
| 448 | cg09124496 | -0.0066 | 1.94E-05 | 7 | *LOC285954* | Body | TRUE | - |
| 449 | cg16911349 | 0.0026 | 1.96E-05 | 19 | *ICAM5* | Body | TRUE | Island |
| 450 | cg21395629 | -0.0028 | 1.96E-05 | 17 | *-* | - | NA | - |
| 451 | cg03763997 | -0.0076 | 2.00E-05 | 10 | *MSRB2* | TSS1500 | NA | N_Shore |
| 452 | cg07573085 | 0.0051 | 2.01E-05 | 4 | *LETM1* | Body | NA | S_Shelf |
| 453 | cg02879554 | 0.0097 | 2.01E-05 | 16 | *MIR1225* | TSS1500 | NA | Island |
| 454 | cg05191675 | 0.0037 | 2.03E-05 | 6 | *APOBEC2* | Body | NA | - |
| 455 | cg09401532 | -0.0039 | 2.02E-05 | 16 | *-* | - | NA | - |
| 456 | cg10123952 | -0.0098 | 2.08E-05 | 3 | *-* | - | TRUE | - |
| 457 | cg02064830 | 0.0033 | 2.08E-05 | 19 | *FAM129C* | Body | NA | - |
| 458 | cg02910018 | 0.0109 | 2.14E-05 | 12 | *TMTC1* | Body | NA | - |
| 459 | cg13734646 | -0.0068 | 2.16E-05 | 20 | *WISP2* | TSS1500 | NA | - |
| 460 | cg20272935 | 0.0058 | 2.19E-05 | 11 | *UNC93B1* | Body | TRUE | S_Shore |
| 461 | cg10585061 | -0.0101 | 2.19E-05 | 8 | *-* | - | NA | - |
| 462 | cg16054275 | -0.0048 | 2.21E-05 | 1 | *F5* | TSS1500 | TRUE | - |
| 463 | cg10537261 | -0.0065 | 2.26E-05 | 11 | *-* | - | NA | - |
| 464 | cg05816791 | -0.0041 | 2.28E-05 | 1 | *-* | - | NA | - |
| 465 | cg16254756 | -0.0034 | 2.32E-05 | 1 | *SHC1* | 5'UTR | TRUE | N_Shelf |
| 466 | cg07252010 | -0.0057 | 2.33E-05 | 4 | *ABCG2* | 5'UTR | NA | - |
| 467 | cg17254414 | -0.0038 | 2.33E-05 | 14 | *-* | - | NA | - |
| 468 | cg23942708 | 0.0049 | 2.33E-05 | 2 | *-* | - | NA | - |
| 469 | cg23327200 | 0.0042 | 2.33E-05 | 17 | *TTYH2* | 5'UTR | NA | - |
| 470 | cg03089032 | -0.006 | 2.35E-05 | 15 | *MYO1E* | Body | NA | - |
| 471 | cg20767370 | -0.0064 | 2.35E-05 | 18 | *LINC00907* | Body | NA | - |
| 472 | cg23606718 | 0.0018 | 2.35E-05 | 2 | *FAM123C* | 5'UTR | TRUE | Island |
| 473 | cg03653399 | -0.0096 | 2.38E-05 | 8 | *SLC45A4* | Body | TRUE | S_Shore |
| 474 | cg18207133 | -0.0042 | 2.39E-05 | 14 | *JPH4* | Body | NA | N_Shelf |
| 475 | cg03231117 | -0.0032 | 2.41E-05 | 9 | *LCN8* | TSS200 | NA | N_Shore |
| 476 | cg25439632 | -0.0096 | 2.41E-05 | 8 | *-* | - | TRUE | S_Shore |
| 477 | cg27212232 | 0.0037 | 2.44E-05 | 20 | *DOK5* | TSS1500 | TRUE | N_Shore |
| 478 | cg00449067 | -0.0064 | 2.45E-05 | 2 | *CALCRL* | 5'UTR | TRUE | - |
| 479 | cg27207809 | -0.0077 | 2.51E-05 | 1 | *-* | - | NA | - |
| 480 | cg16714760 | -0.004 | 2.52E-05 | 4 | *DCHS2* | Body | NA | - |
| 481 | cg01188867 | -0.0067 | 2.58E-05 | 1 | *HMCN1* | Body | NA | - |
| 482 | cg12079303 | -0.0065 | 2.59E-05 | 1 | *NFIA* | Body | TRUE | N_Shore |
| 483 | cg20773033 | -0.0041 | 2.59E-05 | 22 | *PDGFB* | Body | TRUE | N_Shelf |
| 484 | cg02326883 | -0.0043 | 2.63E-05 | 17 | *STARD3* | 3'UTR | NA | N_Shelf |
| 485 | cg08410918 | -0.0057 | 2.63E-05 | 14 | *-* | - | NA | - |
| 486 | cg15750549 | -0.008 | 2.64E-05 | 16 | *LOC101928708* | TSS1500 | NA | - |
| 487 | cg18147181 | -0.0045 | 2.67E-05 | 12 | *KRT7* | Body | NA | N_Shore |
| 488 | cg06914505 | -0.0042 | 2.73E-05 | 15 | *-* | - | TRUE | - |
| 489 | cg15581911 | -0.0048 | 2.74E-05 | 1 | *-* | - | NA | - |
| 490 | cg12962542 | -0.0098 | 2.77E-05 | 3 | *MECOM* | Body | TRUE | - |
| 491 | cg14730524 | -0.0063 | 2.79E-05 | 11 | *FAT3* | Body | TRUE | S_Shore |
| 492 | cg03193328 | -0.0059 | 2.80E-05 | 7 | *TPST1* | Body | TRUE | - |
| 493 | cg09057685 | -0.0099 | 2.80E-05 | 5 | *-* | - | NA | - |
| 494 | cg00009602 | -0.0056 | 2.88E-05 | 3 | *MCF2L2* | Body | NA | - |
| 495 | cg00507010 | -0.0126 | 2.90E-05 | 11 | *APIP* | Body | NA | - |
| 496 | cg27308414 | -0.0043 | 2.90E-05 | 13 | *-* | - | NA | - |
| 497 | cg01561629 | -0.0077 | 2.92E-05 | 1 | *OSBPL9* | TSS1500 | TRUE | - |
| 498 | cg10302347 | -0.0058 | 2.93E-05 | 16 | *TMEM204* | TSS1500 | NA | S_Shelf |
| 499 | cg15144237 | -0.0061 | 2.93E-05 | 2 | *-* | - | NA | - |
| 500 | cg17891166 | -0.0043 | 2.93E-05 | 7 | *ELN* | TSS200 | NA | - |
| 501 | cg20702887 | 0.0039 | 2.95E-05 | 17 | *TTYH2* | 5'UTR | NA | - |
| 502 | cg00886293 | -0.0056 | 2.97E-05 | 20 | *-* | - | NA | - |
| 503 | cg27180636 | 0.0122 | 2.97E-05 | 3 | *FRG2C* | 3'UTR | TRUE | N_Shelf |
| 504 | cg17471102 | -0.0044 | 2.97E-05 | 19 | *FUT3* | 5'UTR | TRUE | - |
| 505 | cg03277049 | 0.0063 | 3.01E-05 | 3 | *-* | - | TRUE | Island |
| 506 | cg01597480 | -0.0095 | 3.03E-05 | 7 | *C7orf58* | 5'UTR | TRUE | - |
| 507 | cg08952306 | 0.0103 | 3.04E-05 | 7 | *SH2B2* | 3'UTR | TRUE | Island |
| 508 | cg04229059 | -0.0028 | 3.10E-05 | 16 | *SLC38A7* | TSS1500 | TRUE | S_Shore |
| 509 | cg24597353 | 0.009 | 3.12E-05 | 4 | *VEGFC* | TSS1500 | NA | S_Shore |
| 510 | cg08072685 | -0.0046 | 3.13E-05 | 8 | *RNF19A* | Body | NA | - |
| 511 | cg08440987 | 0.0035 | 3.14E-05 | 11 | *-* | - | NA | - |
| 512 | cg05765011 | -0.0064 | 3.15E-05 | 16 | *ADCY9* | Body | TRUE | - |
| 513 | cg00101260 | -0.0027 | 3.16E-05 | 17 | *-* | - | TRUE | - |
| 514 | cg19076985 | -0.005 | 3.17E-05 | 3 | *C3orf22* | 5'UTR | NA | - |
| 515 | cg14879089 | -0.0074 | 3.19E-05 | 4 | *CRMP1* | Body | NA | - |
| 516 | cg27300045 | -0.0019 | 3.20E-05 | 19 | *ZC3H4* | Body | TRUE | N_Shelf |
| 517 | cg18627729 | -0.0056 | 3.24E-05 | 13 | *-* | - | NA | - |
| 518 | cg01477894 | -0.0083 | 3.29E-05 | 12 | *-* | - | NA | - |
| 519 | cg11313529 | 0.0039 | 3.30E-05 | 22 | *TRABD* | 5'UTR | NA | N_Shore |
| 520 | cg24681499 | 0.0035 | 3.38E-05 | 1 | *FOXJ3* | Body | TRUE | - |
| 521 | cg05579534 | -0.0038 | 3.48E-05 | 9 | *-* | - | NA | - |
| 522 | cg19542445 | -0.0047 | 3.50E-05 | 12 | *CAC-1C* | Body | TRUE | - |
| 523 | cg25940196 | -0.0064 | 3.52E-05 | 14 | *NPAS3* | Body | NA | - |
| 524 | cg19283806 | -0.0094 | 3.56E-05 | 18 | *CCDC102B* | 5'UTR | TRUE | - |
| 525 | cg24445391 | -0.0042 | 3.61E-05 | 5 | *ADAMTS2* | Body | NA | - |
| 526 | cg03381973 | 0.0052 | 3.72E-05 | 9 | *STXBP1* | Body | NA | - |
| 527 | cg14890220 | -0.0079 | 3.74E-05 | 3 | *NLGN1* | Body | TRUE | - |
| 528 | cg14898223 | -0.008 | 3.74E-05 | 2 | *-* | - | TRUE | S_Shore |
| 529 | cg08726667 | -0.0052 | 3.80E-05 | 16 | *CCDC101* | 5'UTR | TRUE | - |
| 530 | cg08371497 | -0.0051 | 3.91E-05 | 22 | *-* | - | NA | - |
| 531 | cg21899500 | 0.0051 | 3.91E-05 | 3 | *GRM2* | TSS1500 | TRUE | Island |
| 532 | cg10756618 | -0.0057 | 3.91E-05 | 3 | *CHCHD6* | Body | NA | - |
| 533 | cg01720616 | -0.0103 | 3.92E-05 | 7 | *-* | - | TRUE | - |
| 534 | cg04686634 | -0.0033 | 3.94E-05 | 4 | *LOC101929095* | Body | NA | - |
| 535 | cg10101600 | 0.0041 | 3.96E-05 | 2 | *THADA* | Body | TRUE | - |
| 536 | cg16570547 | -0.0056 | 3.96E-05 | 20 | *CBFA2T2* | Body | NA | - |
| 537 | cg19784428 | -0.0046 | 3.98E-05 | 19 | *NWD1* | TSS200 | TRUE | - |
| 538 | cg19270739 | -0.0083 | 4.00E-05 | 1 | *-* | - | TRUE | N_Shore |
| 539 | cg09423126 | 0.0043 | 4.05E-05 | 8 | *PEBP4* | Body | TRUE | - |
| 540 | cg12875837 | -0.0033 | 4.05E-05 | 10 | *SUFU* | Body | NA | - |
| 541 | cg14209730 | -0.0071 | 4.05E-05 | 2 | *-* | - | TRUE | - |
| 542 | cg06413398 | -0.013 | 4.06E-05 | 6 | *DDO* | TSS200 | TRUE | - |
| 543 | cg13612317 | -0.0062 | 4.06E-05 | 10 | *KIF5B* | TSS1500 | TRUE | S_Shore |
| 544 | cg15656455 | -0.004 | 4.06E-05 | 6 | *-* | - | NA | - |
| 545 | cg18651026 | -0.0037 | 4.07E-05 | 6 | *COL11A2* | Body | TRUE | - |
| 546 | cg15034962 | -0.0082 | 4.07E-05 | 7 | *PLEKHA8* | 3'UTR | TRUE | - |
| 547 | cg06639320 | 0.005 | 4.14E-05 | 2 | *FHL2* | TSS200 | TRUE | Island |
| 548 | cg26921969 | 0.0027 | 4.14E-05 | 5 | *-* | - | TRUE | - |
| 549 | cg12499872 | 0.0065 | 4.18E-05 | 16 | *TEPP* | Body | TRUE | Island |
| 550 | cg17186667 | -0.0043 | 4.18E-05 | 18 | *-* | - | NA | - |
| 551 | cg12096762 | -0.0053 | 4.19E-05 | 15 | *-* | - | TRUE | - |
| 552 | cg11105610 | 0.0051 | 4.20E-05 | 17 | *LGALS3BP* | TSS1500 | TRUE | - |
| 553 | cg07446248 | -0.0092 | 4.33E-05 | 6 | *-* | - | NA | - |
| 554 | cg14950321 | -0.0043 | 4.40E-05 | 19 | *PLIN5* | Body | TRUE | N_Shore |
| 555 | cg00387658 | -0.0048 | 4.41E-05 | 20 | *CASS4* | TSS1500 | TRUE | - |
| 556 | cg14317491 | -0.0037 | 4.42E-05 | 5 | *EDIL3* | Body | NA | - |
| 557 | cg08234504 | -0.0025 | 4.47E-05 | 5 | *-* | - | TRUE | N_Shelf |
| 558 | cg18773260 | 0.0077 | 4.47E-05 | 17 | *HOXB7* | Body | TRUE | Island |
| 559 | cg25181130 | -0.0071 | 4.47E-05 | 17 | *RHBDL3* | 3'UTR | NA | - |
| 560 | cg21223983 | -0.0046 | 4.48E-05 | 12 | *-* | - | TRUE | - |
| 561 | cg11148327 | -0.0038 | 4.49E-05 | 6 | *-* | - | NA | - |
| 562 | cg21860785 | -0.0067 | 4.54E-05 | 1 | *ESRRG* | Body | NA | - |
| 563 | cg12100089 | -0.0037 | 4.55E-05 | 5 | *-* | - | NA | - |
| 564 | cg18147543 | -0.0024 | 4.55E-05 | 1 | *ZBTB17* | 5'UTR | TRUE | - |
| 565 | cg11041817 | 0.0104 | 4.63E-05 | 17 | *HOXB7* | Body | TRUE | Island |
| 566 | cg26766373 | -0.0036 | 4.73E-05 | 11 | *ARRB1* | TSS1500 | TRUE | S_Shore |
| 567 | cg12822816 | 0.0031 | 4.76E-05 | 10 | *INPP5A* | Body | TRUE | Island |
| 568 | cg12099677 | -0.0088 | 4.77E-05 | 3 | *ZBTB20* | Body | NA | - |
| 569 | cg07553761 | 0.0056 | 4.80E-05 | 3 | *TRIM59* | TSS1500 | TRUE | Island |
| 570 | cg03443943 | -0.0043 | 4.86E-05 | 4 | *-* | - | NA | - |
| 571 | cg27286120 | -0.0061 | 4.86E-05 | 13 | *PCDH9* | 5'UTR | NA | N_Shore |
| 572 | cg27263966 | -0.0054 | 4.86E-05 | 2 | *-* | - | NA | - |
| 573 | cg03779374 | -0.0091 | 4.93E-05 | 3 | *ZBTB20* | 5'UTR | TRUE | - |
| 574 | cg01815259 | -0.0048 | 4.93E-05 | 2 | *-* | - | NA | - |
| 575 | cg03609058 | -0.0039 | 4.93E-05 | 17 | *ITGA2B* | TSS1500 | NA | - |
| 576 | cg06247012 | -0.0064 | 4.93E-05 | 19 | *FBXO17* | TSS1500 | NA | S_Shore |
| 577 | cg13113967 | -0.0036 | 4.93E-05 | 14 | *CCDC85C* | Body | NA | - |
| 578 | cg03431918 | -0.0031 | 4.98E-05 | 17 | *-* | - | TRUE | S_Shore |
| 579 | cg03224418 | -0.0033 | 4.98E-05 | 20 | *SAMD10* | TSS1500 | TRUE | N_Shore |
| 580 | cg01252023 | -0.0062 | 5.01E-05 | 11 | *CORO1B* | Body | TRUE | S_Shelf |
| 581 | cg09149737 | -0.0034 | 5.03E-05 | 20 | *PRPF6* | TSS1500 | NA | S_Shore |
| 582 | cg10077239 | 0.0061 | 5.03E-05 | 14 | *PRKD1* | TSS1500 | TRUE | S_Shore |
| 583 | cg10288525 | -0.006 | 5.03E-05 | 13 | *-* | - | TRUE | - |
| 584 | cg00868728 | 0.0039 | 5.03E-05 | X | *USP9X* | 5'UTR | NA | - |
| 585 | cg01146320 | -0.0029 | 5.03E-05 | 11 | *OAF* | TSS1500 | TRUE | N_Shore |
| 586 | cg04697265 | -0.0046 | 5.06E-05 | 11 | *-* | - | TRUE | - |
| 587 | cg19750282 | -0.0043 | 5.06E-05 | 6 | *-* | - | TRUE | - |
| 588 | cg23453610 | -0.0059 | 5.12E-05 | 2 | *-* | - | NA | - |
| 589 | cg05308107 | -0.008 | 5.14E-05 | 13 | *PCDH9* | 5'UTR | NA | N_Shore |
| 590 | cg01171329 | -0.0033 | 5.14E-05 | 9 | *CCL19* | Body | NA | - |
| 591 | cg17737621 | 0.0028 | 5.17E-05 | 20 | *-* | - | TRUE | S_Shore |
| 592 | cg10328813 | -0.0028 | 5.17E-05 | 9 | *-* | - | NA | - |
| 593 | cg17600785 | -0.0047 | 5.27E-05 | 3 | *ERC2* | Body | NA | - |
| 594 | cg20360286 | -0.0066 | 5.27E-05 | 2 | *LYPD6* | 5'UTR | TRUE | - |
| 595 | cg26521497 | -0.0052 | 5.27E-05 | 8 | *-* | - | NA | - |
| 596 | cg25693132 | 0.0044 | 5.29E-05 | 3 | *GRM2* | TSS1500 | TRUE | Island |
| 597 | cg03834786 | -0.0059 | 5.36E-05 | 7 | *-* | - | NA | - |
| 598 | cg14123326 | -0.0038 | 5.36E-05 | 2 | *LINC01119* | Body | NA | - |
| 599 | cg01101873 | -0.0071 | 5.49E-05 | 1 | *PRDM16* | Body | TRUE | - |
| 600 | cg16932827 | -0.0036 | 5.55E-05 | 3 | *-* | - | TRUE | S_Shore |
| 601 | cg21191743 | 0.0028 | 5.55E-05 | 1 | *VTCN1* | Body | NA | - |
| 602 | cg26133399 | -0.0067 | 5.59E-05 | 12 | *FAM19A2* | 5'UTR | TRUE | N_Shore |
| 603 | cg12033216 | -0.0034 | 5.69E-05 | 14 | *-* | - | TRUE | - |
| 604 | cg09932507 | -0.0057 | 5.77E-05 | 17 | *LOC100288866* | Body | NA | N_Shore |
| 605 | cg23095368 | 0.0054 | 5.84E-05 | 4 | *-* | - | NA | - |
| 606 | cg04212103 | -0.0041 | 5.84E-05 | 14 | *-* | - | NA | - |
| 607 | cg18881380 | -0.0048 | 5.90E-05 | 2 | *IL1R1* | TSS1500 | NA | N_Shore |
| 608 | cg20918823 | -0.0027 | 6.09E-05 | 17 | *ANKFN1* | Body | NA | - |
| 609 | cg10700560 | 0.0056 | 6.10E-05 | 10 | *LHPP* | Body | NA | - |
| 610 | cg04193160 | 0.0048 | 6.10E-05 | 1 | *OBSCN* | Body | TRUE | Island |
| 611 | cg24951886 | 0.0063 | 6.10E-05 | 10 | *BEND7* | Body | TRUE | - |
| 612 | cg06472341 | -0.0037 | 6.26E-05 | 11 | *MUC2* | TSS1500 | TRUE | - |
| 613 | cg13959344 | -0.0036 | 6.30E-05 | 6 | *-* | - | TRUE | - |
| 614 | cg09285095 | -0.003 | 6.34E-05 | 22 | *LOC100271722* | Body | TRUE | Island |
| 615 | cg00541293 | -0.0056 | 6.36E-05 | 4 | *-* | - | NA | - |
| 616 | cg25841970 | -0.0045 | 6.37E-05 | 7 | *GRID2IP* | Body | NA | N_Shore |
| 617 | cg23496836 | -0.0134 | 6.37E-05 | 4 | *-* | - | NA | - |
| 618 | cg21051972 | 0.0046 | 6.43E-05 | 11 | *-* | - | TRUE | Island |
| 619 | cg19727165 | -0.0059 | 6.53E-05 | 3 | *-* | - | NA | - |
| 620 | cg14887028 | -0.0021 | 6.60E-05 | 2 | *-* | - | NA | N_Shore |
| 621 | cg27005906 | -0.0037 | 6.74E-05 | 12 | *-* | - | TRUE | - |
| 622 | cg23108580 | 0.0067 | 6.82E-05 | 12 | *KIF21A* | Body | TRUE | - |
| 623 | cg01569067 | -0.0053 | 6.83E-05 | 9 | *-* | - | NA | - |
| 624 | cg18769729 | -0.0046 | 6.84E-05 | 17 | *ASPA* | 5'UTR | NA | S_Shelf |
| 625 | cg23222302 | -0.002 | 7.22E-05 | 2 | *-* | - | NA | - |
| 626 | cg00227665 | 0.0044 | 7.26E-05 | 16 | *LOC100129637* | Body | TRUE | N_Shore |
| 627 | cg07447769 | -0.0033 | 7.26E-05 | 13 | *-* | - | TRUE | - |
| 628 | cg00454305 | 0.0077 | 7.26E-05 | 16 | *UNKL* | TSS1500 | TRUE | Island |
| 629 | cg06717633 | 0.0051 | 7.26E-05 | 14 | *PRKD1* | Body | NA | - |
| 630 | cg13628404 | -0.0047 | 7.26E-05 | 14 | *NRXN3* | Body | NA | - |
| 631 | cg16545019 | -0.0046 | 7.26E-05 | 9 | *NFIB* | Body | NA | - |
| 632 | cg25745729 | 0.0054 | 7.59E-05 | 10 | *-* | - | NA | - |
| 633 | cg25329021 | -0.0072 | 7.61E-05 | 11 | *-* | - | NA | - |
| 634 | cg18811095 | -0.0039 | 7.61E-05 | 6 | *-* | - | NA | - |
| 635 | cg26906217 | -0.0039 | 7.61E-05 | 11 | *-* | - | TRUE | S_Shore |
| 636 | cg15218354 | -0.0035 | 7.62E-05 | 1 | *ABCA4* | Body | NA | - |
| 637 | cg15827870 | 0.0031 | 7.62E-05 | 2 | *MTA3* | 1stExon | NA | N_Shore |
| 638 | cg08191886 | -0.0066 | 7.63E-05 | 6 | *-* | - | NA | - |
| 639 | cg01566232 | -0.0072 | 7.63E-05 | X | *SPRY3* | 5'UTR | NA | - |
| 640 | cg00059652 | -0.0047 | 7.69E-05 | 17 | *-* | - | TRUE | - |
| 641 | cg03680238 | 0.0039 | 7.74E-05 | 11 | *EEF1G* | TSS1500 | NA | S_Shore |
| 642 | cg23681440 | -0.0068 | 7.80E-05 | 13 | *-* | - | TRUE | - |
| 643 | cg08023684 | -0.0054 | 8.00E-05 | 10 | *-* | - | NA | - |
| 644 | cg02657721 | -0.0031 | 8.05E-05 | 3 | *SEMA3B* | TSS1500 | NA | - |
| 645 | cg23580639 | 0.0045 | 8.09E-05 | 17 | *ACACA* | Body | NA | - |
| 646 | cg04751276 | 0.0027 | 8.24E-05 | 15 | *-* | - | TRUE | Island |
| 647 | cg05637940 | 0.0069 | 8.24E-05 | 14 | *CCDC88C* | Body | NA | N_Shelf |
| 648 | cg21053786 | -0.0054 | 8.24E-05 | 3 | *-* | - | NA | - |
| 649 | cg11084334 | 0.0035 | 8.28E-05 | 3 | *LHFPL4* | Body | TRUE | Island |
| 650 | cg00706656 | -0.0039 | 8.30E-05 | 5 | *-* | - | NA | N_Shelf |
| 651 | cg03784270 | -0.0028 | 8.51E-05 | 3 | *-* | - | NA | - |
| 652 | cg10250177 | 0.0051 | 8.51E-05 | 1 | *LCK* | 5'UTR | TRUE | N_Shore |
| 653 | cg00075507 | 0.0048 | 8.57E-05 | 17 | *SARM1* | Body | TRUE | Island |
| 654 | cg03090572 | -0.0017 | 8.68E-05 | 3 | *LRRFIP2* | TSS200 | NA | N_Shore |
| 655 | cg12285003 | -0.0029 | 8.81E-05 | 20 | *SLC17A9* | Body | NA | - |
| 656 | cg11637118 | 0.0033 | 8.95E-05 | 16 | *CDIP1* | 5'UTR | NA | - |
| 657 | cg19368440 | -0.0117 | 9.05E-05 | 1 | *LOC100505795* | TSS200 | NA | - |
| 658 | cg15389227 | -0.0075 | 9.10E-05 | 7 | *CADPS2* | Body | NA | - |
| 659 | cg08655800 | -0.0036 | 9.11E-05 | 1 | *KIAA1324* | Body | TRUE | - |
| 660 | cg13053302 | -0.0084 | 9.11E-05 | 5 | *MAP1B* | Body | NA | - |
| 661 | cg27097923 | 0.0055 | 9.11E-05 | 6 | *TPBG* | TSS1500 | TRUE | N_Shore |
| 662 | cg04357717 | -0.0078 | 9.11E-05 | 3 | *ZPLD1* | Body | TRUE | - |
| 663 | cg10786503 | -0.0092 | 9.39E-05 | 9 | *ADAMTSL1* | Body | NA | - |
| 664 | cg00096705 | -0.0076 | 9.39E-05 | 12 | *A2ML1* | 1stExon | NA | - |
| 665 | cg18741807 | -0.0044 | 9.56E-05 | 16 | *-* | - | NA | - |
| 666 | cg03054277 | 0.0094 | 9.66E-05 | 1 | *OBSCN* | Body | TRUE | Island |
| 667 | cg14776578 | -0.0054 | 9.68E-05 | 10 | *KIF5B* | TSS1500 | TRUE | S_Shore |
| 668 | cg23665568 | -0.0048 | 9.77E-05 | 1 | *BCL10* | TSS1500 | TRUE | S_Shelf |
| 669 | cg24752967 | 0.0045 | 9.96E-05 | 8 | *SORBS3* | 5'UTR | NA | S_Shelf |
| 670 | cg01323777 | 0.0156 | 0.0001 | 17 | *KC-B3* | TSS200 | TRUE | Island |
| 671 | cg14206172 | -0.008 | 0.000101 | 19 | *FBXO17* | TSS1500 | TRUE | S_Shore |
| 672 | cg23392367 | -0.0037 | 0.000101 | 4 | *-* | - | NA | - |
| 673 | cg01800052 | -0.0041 | 0.000101 | 2 | *-* | - | NA | - |
| 674 | cg00443981 | 0.004 | 0.000102 | 17 | *C17orf64* | TSS200 | TRUE | S_Shore |
| 675 | cg22219054 | -0.0068 | 0.000102 | 5 | *-* | - | NA | - |
| 676 | cg22682811 | 0.002 | 0.000102 | 20 | *-* | - | TRUE | Island |
| 677 | cg23585673 | 0.0039 | 0.000102 | 3 | *GRM2* | 5'UTR | NA | S_Shore |
| 678 | cg13332381 | -0.0043 | 0.000103 | 12 | *-* | - | NA | - |
| 679 | cg19671120 | 0.0019 | 0.000105 | 2 | *CNGA3* | 1stExon | TRUE | Island |
| 680 | cg24791731 | -0.0091 | 0.000106 | 10 | *PLXDC2* | Body | NA | S_Shore |
| 681 | cg07650421 | 0.006 | 0.000106 | 9 | *-* | - | NA | - |
| 682 | cg16916987 | 0.0052 | 0.000106 | 20 | *ZMYND8* | 5'UTR | NA | - |
| 683 | cg13215110 | -0.0052 | 0.000108 | 12 | *FAM19A2* | 5'UTR | NA | - |
| 684 | cg23005170 | -0.0057 | 0.000108 | 6 | *-* | - | NA | - |
| 685 | cg23487414 | -0.0067 | 0.000108 | 6 | *NRM* | TSS1500 | NA | S_Shelf |
| 686 | cg03164932 | -0.0064 | 0.000109 | 3 | *-* | - | NA | - |
| 687 | cg09015115 | -0.0062 | 0.00011 | 14 | *TCL6* | Body | NA | - |
| 688 | cg06060874 | -0.007 | 0.000111 | 1 | *PRDM16* | Body | TRUE | - |
| 689 | cg22945467 | 0.0046 | 0.000113 | 1 | *FAIM3* | TSS1500 | TRUE | - |
| 690 | cg22276442 | -0.0038 | 0.000113 | 1 | *SEMA4A* | TSS1500 | NA | - |
| 691 | cg01632288 | 0.006 | 0.000114 | 19 | *HOOK2* | Body | TRUE | Island |
| 692 | cg10562405 | -0.0038 | 0.000114 | 17 | *-* | - | NA | - |
| 693 | cg05617469 | 0.0019 | 0.000115 | 13 | *ZIC5* | TSS200 | TRUE | Island |
| 694 | cg07974441 | -0.0035 | 0.000115 | 10 | *ADIRF* | TSS1500 | NA | N_Shore |
| 695 | cg15393221 | -0.0037 | 0.000115 | 19 | *PRX* | TSS1500 | TRUE | - |
| 696 | cg19612068 | -0.0044 | 0.000115 | 6 | *-* | - | TRUE | S_Shore |
| 697 | cg18853490 | -0.0044 | 0.000115 | 1 | *CHRM3* | 5'UTR | TRUE | - |
| 698 | cg25015038 | -0.0052 | 0.000116 | 14 | *-* | - | TRUE | - |
| 699 | cg04937184 | 0.0038 | 0.000117 | 14 | *BMP4* | TSS1500 | TRUE | S_Shore |
| 700 | cg06732228 | 0.0014 | 0.000119 | 19 | *LTBP4* | Body | TRUE | Island |
| 701 | cg17094269 | 0.0033 | 0.000119 | 10 | *-* | - | NA | - |
| 702 | cg06835483 | -0.0031 | 0.00012 | 17 | *ALOX12B* | TSS200 | TRUE | - |
| 703 | cg03211864 | -0.0029 | 0.00012 | 10 | *BTBD16* | Body | TRUE | - |
| 704 | cg13790734 | -0.0074 | 0.00012 | 2 | *-* | - | NA | - |
| 705 | cg14179401 | -0.0037 | 0.000121 | 17 | *-* | - | TRUE | - |
| 706 | cg14978069 | -0.0043 | 0.000121 | 4 | *SCLT1* | Body | NA | - |
| 707 | cg00365672 | -0.0042 | 0.000122 | 6 | *-* | - | NA | - |
| 708 | cg08230167 | -0.0047 | 0.000122 | 9 | *LURAP1LAS1* | TSS200 | NA | - |
| 709 | cg17002328 | 0.0047 | 0.000122 | 14 | *CCDC88C* | Body | TRUE | N_Shelf |
| 710 | cg25183883 | -0.0089 | 0.000122 | 13 | *KLHL1* | Body | NA | - |
| 711 | cg09258479 | -0.0043 | 0.000122 | 1 | *PDZK1IP1* | TSS200 | TRUE | - |
| 712 | cg10526506 | 0.0044 | 0.000122 | 5 | *-* | - | NA | - |
| 713 | cg13888445 | 0.003 | 0.000123 | 9 | *AK1* | 3'UTR | TRUE | - |
| 714 | cg26242531 | -0.005 | 0.000123 | 14 | *ZFYVE21* | Body | TRUE | N_Shelf |
| 715 | cg08468401 | -0.0063 | 0.000124 | 3 | *-* | - | TRUE | - |
| 716 | cg21201760 | 0.0038 | 0.000125 | 2 | *INPP4A* | 1stExon | NA | - |
| 717 | cg03978682 | -0.0055 | 0.000127 | 5 | *ARL15* | Body | TRUE | - |
| 718 | cg11891925 | -0.0044 | 0.000127 | 10 | *-* | - | TRUE | - |
| 719 | cg15866363 | 0.0041 | 0.000127 | 1 | *LOC100506801* | Body | NA | S_Shelf |
| 720 | cg20515136 | -0.0042 | 0.000127 | 3 | *IL12A* | Body | TRUE | S_Shore |
| 721 | cg14482712 | 0.0036 | 0.000128 | 15 | *-* | - | TRUE | - |
| 722 | cg08997126 | -0.0058 | 0.00013 | 1 | *-* | - | NA | - |
| 723 | cg01703858 | -0.0057 | 0.000132 | 6 | *-* | - | NA | - |
| 724 | cg04749503 | -0.0055 | 0.000132 | 2 | *-* | - | NA | - |
| 725 | cg13949829 | 0.0125 | 0.000132 | 16 | *MIR1225* | TSS1500 | NA | Island |
| 726 | cg18480675 | -0.0054 | 0.000132 | 3 | *CMC1* | TSS1500 | TRUE | N_Shore |
| 727 | cg13147016 | -0.0047 | 0.000135 | 18 | *-* | - | NA | - |
| 728 | cg18171433 | -0.0043 | 0.000135 | 7 | *-* | - | NA | - |
| 729 | cg19142089 | -0.0052 | 0.000135 | 7 | *-* | - | NA | - |
| 730 | cg25826143 | -0.0054 | 0.000135 | 9 | *KANK1* | Body | NA | - |
| 731 | cg02844688 | -0.0124 | 0.000136 | 20 | *-* | - | NA | - |
| 732 | cg08082321 | -0.0033 | 0.000136 | 8 | *CCDC26* | TSS1500 | NA | - |
| 733 | cg14398464 | -0.0028 | 0.000136 | 22 | *NOL12* | TSS1500 | TRUE | N_Shore |
| 734 | cg24621690 | 0.0035 | 0.000136 | 7 | *-* | - | NA | - |
| 735 | cg02988947 | 0.0044 | 0.000136 | 17 | *LIMD2* | TSS1500 | TRUE | S_Shore |
| 736 | cg05335422 | -0.0037 | 0.000137 | 12 | *CELA1* | 1stExon | NA | - |
| 737 | cg07219494 | -0.0098 | 0.000137 | 5 | *-* | - | TRUE | S_Shelf |
| 738 | cg04654868 | -0.0041 | 0.000138 | 6 | *TPD52L1* | 5'UTR | NA | - |
| 739 | cg06942979 | 0.0074 | 0.000138 | 22 | *SELM* | Body | TRUE | N_Shore |
| 740 | cg05267739 | -0.0034 | 0.000138 | 11 | *-* | - | NA | S_Shore |
| 741 | cg14203218 | -0.004 | 0.000138 | 7 | *-* | - | TRUE | - |
| 742 | cg06069616 | -0.0027 | 0.00014 | 21 | *C21orf63* | TSS1500 | TRUE | N_Shore |
| 743 | cg01965047 | 0.0061 | 0.000143 | 16 | *PKD1* | Body | TRUE | Island |
| 744 | cg21244580 | 0.0135 | 0.000143 | 8 | *-* | - | TRUE | - |
| 745 | cg19810426 | -0.0069 | 0.000144 | 6 | *STXBP5AS1* | TSS1500 | NA | S_Shore |
| 746 | cg10384676 | 0.0026 | 0.000146 | 5 | *PRLR* | 5'UTR | NA | - |
| 747 | cg13423770 | -0.0033 | 0.000149 | 1 | *RHBG* | TSS1500 | TRUE | Island |
| 748 | cg07134623 | -0.005 | 0.00015 | 4 | *COPS4* | Body | NA | - |
| 749 | cg13129591 | -0.003 | 0.00015 | 3 | *-* | - | NA | - |
| 750 | cg24390713 | -0.0047 | 0.00015 | 3 | *-* | - | NA | - |
| 751 | cg25847702 | -0.004 | 0.00015 | 2 | *LTBP1* | Body | NA | - |
| 752 | cg27345144 | -0.0073 | 0.00015 | 3 | *-* | - | NA | - |
| 753 | cg01966866 | -0.0047 | 0.00015 | 7 | *AQP1* | Body | NA | - |
| 754 | cg04401758 | -0.0038 | 0.00015 | 22 | *-* | - | NA | - |
| 755 | cg07073140 | -0.0036 | 0.000151 | 5 | *NEURL1B* | Body | NA | S_Shore |
| 756 | cg01412886 | -0.0023 | 0.000153 | 14 | *AH-K2* | TSS1500 | TRUE | S_Shore |
| 757 | cg03010094 | -0.0042 | 0.000153 | X | *ATP2B3* | Body | NA | - |
| 758 | cg01820962 | -0.007 | 0.000155 | 6 | *NT5DC1* | Body | TRUE | - |
| 759 | cg10933497 | -0.0038 | 0.000156 | 4 | *-* | - | NA | - |
| 760 | cg06272997 | -0.0037 | 0.000157 | 1 | *-* | - | NA | - |
| 761 | cg03982897 | -0.0033 | 0.000157 | 11 | *-* | - | TRUE | N_Shore |
| 762 | cg03298700 | -0.0049 | 0.000159 | 2 | *-* | - | TRUE | N_Shelf |
| 763 | cg27584135 | -0.0067 | 0.000159 | 3 | *-* | - | TRUE | - |
| 764 | cg14248220 | -0.0048 | 0.00016 | 1 | *-* | - | NA | - |
| 765 | cg22416867 | -0.0028 | 0.00016 | 15 | *-* | - | NA | - |
| 766 | cg08036772 | -0.004 | 0.000161 | 5 | *CTNND2* | Body | NA | - |
| 767 | cg19825856 | -0.0029 | 0.000163 | 2 | *ST6GAL2* | 5'UTR | TRUE | N_Shore |
| 768 | cg09638344 | -0.0017 | 0.000163 | 4 | *WFS1* | TSS1500 | TRUE | N_Shore |
| 769 | cg01769750 | -0.0024 | 0.000166 | 4 | *-* | - | NA | - |
| 770 | cg11010561 | 0.0086 | 0.000166 | 4 | *-* | - | TRUE | - |
| 771 | cg20404850 | -0.0125 | 0.000166 | 9 | *CDC14B* | Body | NA | - |
| 772 | cg21463449 | -0.0055 | 0.000166 | 1 | *-* | - | NA | - |
| 773 | cg09364988 | -0.0043 | 0.000167 | 2 | *ARHGAP25* | Body | TRUE | - |
| 774 | cg06831571 | -0.0041 | 0.000167 | 11 | *-* | - | NA | - |
| 775 | cg18684863 | -0.0035 | 0.000167 | 4 | *CCDC110* | Body | NA | - |
| 776 | cg20153322 | -0.005 | 0.000167 | 12 | *PXN* | TSS1500 | TRUE | S_Shore |
| 777 | cg09461021 | -0.0057 | 0.000168 | 22 | *-* | - | NA | - |
| 778 | cg22248488 | -0.0071 | 0.000168 | 8 | *-* | - | NA | - |
| 779 | cg07217030 | -0.0056 | 0.000168 | 8 | *SLC45A4* | Body | TRUE | S_Shore |
| 780 | cg01355392 | 0.0099 | 0.000171 | 20 | *SOX18* | 3'UTR | TRUE | N_Shore |
| 781 | cg08745595 | -0.0043 | 0.000171 | 1 | *F5* | TSS1500 | NA | - |
| 782 | cg13490273 | -0.004 | 0.000171 | 5 | *-* | - | NA | - |
| 783 | cg16686396 | -0.0074 | 0.000171 | 1 | *PRDM16* | Body | TRUE | - |
| 784 | cg09870137 | -0.0033 | 0.000174 | 22 | *WNT7B* | Body | TRUE | S_Shore |
| 785 | cg13969327 | -0.0051 | 0.000174 | 7 | *SND1* | Body | TRUE | - |
| 786 | cg01028226 | -0.0035 | 0.000174 | 1 | *-* | - | NA | - |
| 787 | cg01797043 | -0.0028 | 0.000174 | 16 | *RPL3L* | TSS200 | TRUE | - |
| 788 | cg18018605 | -0.0071 | 0.000174 | 7 | *-* | - | NA | - |
| 789 | cg26463806 | -0.0051 | 0.000174 | 1 | *-* | - | NA | - |
| 790 | cg07812250 | -0.0072 | 0.000176 | 16 | *C16orf45* | Body | NA | - |
| 791 | cg17723057 | -0.0067 | 0.000175 | 6 | *-* | - | NA | - |
| 792 | cg00218103 | -0.0029 | 0.000176 | 22 | *-* | - | TRUE | Island |
| 793 | cg00698382 | -0.0079 | 0.000176 | 5 | *-* | - | NA | - |
| 794 | cg03199996 | 0.0046 | 0.000177 | 20 | *FAM65C* | Body | TRUE | - |
| 795 | cg06668837 | -0.0055 | 0.000177 | 6 | *SLC22A23* | 5'UTR | NA | - |
| 796 | cg20053878 | -0.0039 | 0.000177 | 16 | *-* | - | NA | - |
| 797 | cg04595171 | -0.0038 | 0.000178 | 12 | *DCTN2* | Body | NA | - |
| 798 | cg24600306 | -0.0035 | 0.000179 | 20 | *CHR-4* | 5'UTR | NA | N_Shore |
| 799 | cg20398091 | -0.0068 | 0.00018 | 19 | *NWD1* | TSS1500 | NA | - |
| 800 | cg11659663 | -0.0054 | 0.000181 | 11 | *MACROD1* | Body | TRUE | S_Shore |
| 801 | cg25303955 | -0.005 | 0.000181 | 15 | *ARNT2* | Body | NA | - |
| 802 | cg26181218 | -0.003 | 0.000181 | 5 | *-* | - | NA | - |
| 803 | cg03250223 | 0.003 | 0.000183 | 3 | *UBE2E2* | 5'UTR | NA | S_Shelf |
| 804 | cg06017039 | 0.0058 | 0.000183 | 12 | *TPH2* | Body | NA | - |
| 805 | cg25135018 | -0.0035 | 0.000185 | 1 | *IL6R* | Body | TRUE | - |
| 806 | cg00067751 | -0.0065 | 0.000187 | 9 | *TMEM246* | TSS1500 | NA | S_Shore |
| 807 | cg21184711 | 0.0069 | 0.000187 | 7 | *CADPS2* | Body | TRUE | - |
| 808 | cg10816468 | -0.0065 | 0.000187 | 6 | *-* | - | NA | - |
| 809 | cg27151122 | -0.0025 | 0.000187 | 12 | *-* | - | TRUE | N_Shelf |
| 810 | cg05404236 | 0.0057 | 0.000189 | 13 | *IRS2* | 1stExon | TRUE | Island |
| 811 | cg27446735 | -0.0061 | 0.000189 | 1 | *SLC19A2* | Body | NA | - |
| 812 | cg06506328 | -0.006 | 0.00019 | 10 | *CTN-3* | Body | NA | - |
| 813 | cg26532090 | 0.003 | 0.000191 | 6 | *-* | - | NA | - |
| 814 | cg06271237 | -0.0043 | 0.000194 | 6 | *PLAGL1* | Body | TRUE | - |
| 815 | cg14024017 | -0.0049 | 0.000195 | 10 | *PLXDC2* | Body | NA | - |
| 816 | cg03048622 | -0.0032 | 0.000196 | 6 | *TPD52L1* | 5'UTR | NA | - |
| 817 | cg15442434 | 0.0045 | 0.000196 | 6 | *-* | - | TRUE | - |
| 818 | cg01795822 | 0.0047 | 0.000198 | 1 | *DFFA* | Body | NA | - |
| 819 | cg01461211 | 0.0029 | 0.000199 | 16 | *-* | - | TRUE | - |
| 820 | cg03354870 | -0.0062 | 0.000199 | 14 | *-* | - | NA | - |
| 821 | cg00498942 | -0.0068 | 0.0002 | 1 | *AK4* | 3'UTR | NA | - |
| 822 | cg02247068 | 0.0033 | 0.0002 | 15 | *ITPKA* | Body | TRUE | N_Shore |
| 823 | cg13705457 | -0.0045 | 0.0002 | 11 | *-* | - | NA | - |
| 824 | cg05768419 | -0.0075 | 0.0002 | 1 | *GPR52* | 1stExon | TRUE | - |
| 825 | cg08426696 | -0.0049 | 0.0002 | 22 | *SEZ6L* | Body | NA | - |
| 826 | cg13601925 | -0.0054 | 0.0002 | 3 | *GMPS* | Body | NA | - |
| 827 | cg15044533 | -0.0102 | 0.000201 | 9 | *PBX3* | Body | NA | - |
| 828 | cg01748712 | -0.0044 | 0.000202 | 9 | *GFI1B* | 5'UTR | NA | S_Shore |
| 829 | cg22039482 | 0.0018 | 0.000205 | 5 | *-* | - | NA | S_Shelf |
| 830 | cg04105200 | -0.0042 | 0.000205 | 3 | *-* | - | NA | - |
| 831 | cg07803108 | 0.0025 | 0.000209 | 17 | *#NAME?* | TSS200 | TRUE | N_Shore |
| 832 | cg20557296 | -0.0042 | 0.000209 | 8 | *RSPO2* | Body | NA | - |
| 833 | cg24554840 | -0.0056 | 0.000209 | 4 | *-* | - | NA | - |
| 834 | cg26759486 | -0.0043 | 0.00021 | 6 | *LOC645434* | Body | NA | - |
| 835 | cg02972988 | 0.0041 | 0.000211 | 11 | *YPEL4* | 5'UTR | NA | S_Shore |
| 836 | cg24935633 | -0.006 | 0.000211 | 7 | *AGMO* | Body | NA | - |
| 837 | cg02661687 | 0.0043 | 0.000211 | 9 | *ST6GAL-C4* | Body | NA | N_Shore |
| 838 | cg15951188 | 0.0104 | 0.000213 | 17 | *KC-B3* | 1stExon | TRUE | Island |
| 839 | cg04347161 | -0.0027 | 0.000214 | 16 | *POLR2C* | TSS1500 | TRUE | N_Shore |
| 840 | cg16621189 | -0.0025 | 0.000214 | 19 | *-* | - | NA | - |
| 841 | cg25105522 | -0.0087 | 0.000214 | 17 | *MAP3K14* | Body | TRUE | - |
| 842 | cg17400925 | 0.003 | 0.000216 | 1 | *GUK1* | 5'UTR | NA | S_Shelf |
| 843 | cg04782823 | -0.0073 | 0.000216 | 9 | *SMARCA2* | Body | NA | - |
| 844 | cg18555368 | -0.0055 | 0.000217 | 3 | *LINC00883* | Body | NA | - |
| 845 | cg24481841 | -0.004 | 0.000218 | 19 | *NCR-00085* | Body | TRUE | N_Shelf |
| 846 | cg21192606 | -0.0061 | 0.000219 | 12 | *-* | - | NA | - |
| 847 | cg23202167 | -0.0039 | 0.000222 | 19 | *-* | - | TRUE | N_Shore |
| 848 | cg02834737 | 0.0076 | 0.000222 | 3 | *-* | - | NA | - |
| 849 | cg26841040 | 0.0027 | 0.000222 | 1 | *GUK1* | TSS200 | TRUE | S_Shelf |
| 850 | cg06682024 | -0.0046 | 0.000222 | 2 | *MERTK* | Body | TRUE | - |
| 851 | cg00491255 | 0.0037 | 0.000223 | 16 | *TEPP* | Body | TRUE | Island |
| 852 | cg07268926 | 0.005 | 0.000223 | 11 | *IGSF9B* | Body | NA | - |
| 853 | cg18158739 | -0.0071 | 0.000223 | 14 | *-* | - | NA | - |
| 854 | cg04865726 | -0.0069 | 0.000224 | 1 | *-* | - | TRUE | S_Shelf |
| 855 | cg08662753 | 0.0062 | 0.000224 | 9 | *COL5A1* | Body | NA | - |
| 856 | cg06479793 | -0.0043 | 0.000227 | 4 | *CCDC158* | Body | NA | - |
| 857 | cg11253886 | -0.0057 | 0.000227 | 2 | *-* | - | TRUE | - |
| 858 | cg03380960 | 0.004 | 0.000227 | 10 | *FAM53B* | Body | NA | - |
| 859 | cg07919707 | -0.0038 | 0.000229 | 3 | *LMCD1AS1* | Body | NA | - |
| 860 | cg12564453 | -0.0117 | 0.00023 | 16 | *CETP* | 5'UTR | TRUE | - |
| 861 | cg24192058 | -0.0057 | 0.000229 | 5 | *LINC01339* | Body | NA | - |
| 862 | cg13929323 | -0.0071 | 0.00023 | 9 | *-* | - | TRUE | S_Shelf |
| 863 | cg23322833 | -0.0058 | 0.00023 | 18 | *-* | - | NA | - |
| 864 | cg02293445 | -0.0024 | 0.00023 | 1 | *-* | - | NA | - |
| 865 | cg21414411 | -0.0069 | 0.00023 | 3 | *-* | - | NA | - |
| 866 | cg10822172 | -0.0044 | 0.00023 | 7 | *CREB5* | TSS200 | TRUE | - |
| 867 | cg15711508 | -0.0044 | 0.000232 | 1 | *-* | - | TRUE | - |
| 868 | cg26677315 | -0.0071 | 0.000232 | 15 | *-* | - | NA | - |
| 869 | cg25882544 | 0.0039 | 0.000233 | 17 | *TOM1L2* | Body | NA | - |
| 870 | cg18880175 | -0.0044 | 0.000233 | 2 | *DPP10* | Body | NA | - |
| 871 | cg24898675 | -0.0038 | 0.000235 | 15 | *SMAD3* | Body | NA | - |
| 872 | cg13706582 | 0.0051 | 0.000235 | 3 | *ZNF385D* | TSS1500 | TRUE | - |
| 873 | cg05056825 | -0.0041 | 0.000236 | 2 | *-* | - | TRUE | - |
| 874 | cg12654941 | -0.006 | 0.000236 | 13 | *-* | - | NA | - |
| 875 | cg02069100 | -0.0037 | 0.000239 | 9 | *SLC2A6* | TSS1500 | NA | S_Shore |
| 876 | cg09836921 | -0.0054 | 0.00024 | 16 | *IFT140* | Body | TRUE | N_Shore |
| 877 | cg04881720 | 0.0026 | 0.000242 | 20 | *SOX18* | Body | NA | Island |
| 878 | cg05360958 | -0.0064 | 0.000244 | 12 | *MGP* | Body | TRUE | - |
| 879 | cg12751127 | -0.0083 | 0.000246 | 8 | *-* | - | NA | - |
| 880 | cg04053132 | -0.0037 | 0.000247 | 20 | *LINC01270* | Body | NA | - |
| 881 | cg06836102 | 0.0036 | 0.000247 | 15 | *-* | - | TRUE | - |
| 882 | cg27236973 | -0.003 | 0.000247 | 17 | *KRT17* | TSS1500 | TRUE | - |
| 883 | cg08120188 | -0.0088 | 0.000248 | 1 | *PGBD5* | Body | NA | S_Shelf |
| 884 | cg01552919 | -0.0021 | 0.000255 | 4 | *GAK* | Body | TRUE | S_Shore |
| 885 | cg01620154 | 0.0034 | 0.000255 | 12 | *HDAC7* | Body | NA | - |
| 886 | cg13080716 | 0.0022 | 0.000256 | 7 | *-* | - | TRUE | S_Shore |
| 887 | cg06748146 | 0.0047 | 0.000256 | 10 | *HK1* | Body | TRUE | - |
| 888 | cg17457912 | -0.0038 | 0.000256 | 17 | *C17orf91* | Body | TRUE | N_Shelf |
| 889 | cg01902066 | -0.0029 | 0.000257 | 16 | *-* | - | TRUE | - |
| 890 | cg18797449 | 0.0061 | 0.000258 | 7 | *RALA* | 5'UTR | NA | - |
| 891 | cg03977382 | -0.0068 | 0.000263 | 11 | *CNTN5* | Body | NA | - |
| 892 | cg01169725 | -0.0043 | 0.000266 | 1 | *CREG1* | Body | NA | N_Shore |
| 893 | cg06477069 | -0.0038 | 0.000266 | 20 | *C20orf117* | TSS1500 | TRUE | S_Shore |
| 894 | cg09050300 | -0.0054 | 0.000266 | 5 | *LOC101929710* | Body | NA | - |
| 895 | cg13357714 | -0.0029 | 0.000266 | 5 | *SGCD* | Body | TRUE | - |
| 896 | cg26394870 | -0.0044 | 0.000267 | 1 | *LOC102724312* | Body | NA | N_Shelf |
| 897 | cg05199226 | -0.0062 | 0.000269 | 20 | *FAM65C* | TSS1500 | NA | - |
| 898 | cg20249566 | -0.0039 | 0.000269 | 19 | *NWD1* | TSS200 | TRUE | - |
| 899 | cg16789844 | -0.0068 | 0.00027 | 7 | *PDE1C* | TSS200 | NA | S_Shore |
| 900 | cg20697702 | -0.0056 | 0.000275 | 4 | *-* | - | NA | - |
| 901 | cg00607630 | 0.003 | 0.000275 | 16 | *USP7* | Body | TRUE | - |
| 902 | cg17465752 | 0.0041 | 0.000275 | 8 | *-* | - | TRUE | Island |
| 903 | cg12769615 | -0.0037 | 0.000278 | 4 | *-* | - | NA | - |
| 904 | cg01803886 | -0.0068 | 0.000281 | 3 | *CNTN4* | Body | TRUE | - |
| 905 | cg13810149 | 0.0074 | 0.000281 | 15 | *MYO5C* | Body | NA | - |
| 906 | cg26042814 | -0.0034 | 0.000281 | 11 | *RNF121* | Body | NA | - |
| 907 | cg18645642 | -0.0038 | 0.000281 | 1 | *RERE* | Body | TRUE | - |
| 908 | cg20265043 | 0.002 | 0.000281 | 7 | *IGF2BP3* | TSS200 | TRUE | S_Shore |
| 909 | cg25486993 | -0.0037 | 0.000282 | 4 | *APBB2* | Body | NA | - |
| 910 | cg03214130 | -0.0027 | 0.000283 | 7 | *-* | - | TRUE | - |
| 911 | cg07317306 | -0.0023 | 0.000284 | 1 | *-* | - | NA | - |
| 912 | cg16924779 | -0.0032 | 0.000285 | 6 | *STXBP5AS1* | Body | NA | - |
| 913 | cg10616795 | -0.0025 | 0.000286 | 5 | *-* | - | TRUE | - |
| 914 | cg11920206 | -0.0043 | 0.000286 | 4 | *STOX2* | Body | NA | - |
| 915 | cg03875330 | -0.0034 | 0.000287 | 19 | *TMEM205* | Body | TRUE | N_Shore |
| 916 | cg05026650 | 0.0023 | 0.000288 | 17 | *GNGT2* | TSS1500 | NA | - |
| 917 | cg08119153 | 0.0075 | 0.000289 | 14 | *NRXN3* | 5'UTR | NA | - |
| 918 | cg26311703 | -0.002 | 0.000289 | 11 | *TM7SF2* | TSS1500 | NA | N_Shore |
| 919 | cg00592995 | 0.005 | 0.000289 | 20 | *ZBP1* | TSS1500 | NA | - |
| 920 | cg09735851 | 0.0025 | 0.000289 | 15 | *-* | - | TRUE | Island |
| 921 | cg13499473 | -0.0071 | 0.000289 | X | *ARHGAP6* | Body | NA | - |
| 922 | cg15477096 | -0.0049 | 0.000289 | 14 | *NPAS3* | TSS1500 | NA | S_Shelf |
| 923 | cg21462693 | 0.0059 | 0.000289 | 6 | *NOTCH4* | Body | TRUE | - |
| 924 | cg25471682 | -0.0022 | 0.00029 | 3 | *-* | - | NA | - |
| 925 | cg26222035 | -0.0022 | 0.00029 | 17 | *-* | - | NA | - |
| 926 | cg05930053 | -0.0076 | 0.00029 | 20 | *CTNNBL1* | 5'UTR | NA | - |
| 927 | cg25311764 | -0.0034 | 0.000291 | 15 | *GCNT3* | 5'UTR | TRUE | - |
| 928 | cg21625786 | 0.0036 | 0.000294 | 21 | *UMODL1* | Body | NA | - |
| 929 | cg07861288 | -0.0034 | 0.000295 | 13 | *GPC5* | Body | NA | - |
| 930 | cg17397004 | -0.0041 | 0.000295 | 22 | *KDELR3* | TSS1500 | TRUE | N_Shore |
| 931 | cg20856960 | -0.0035 | 0.000295 | 11 | *0* | Body | NA | - |
| 932 | cg15751131 | 0.0036 | 0.000297 | 7 | *SLC37A3* | 5'UTR | TRUE | - |
| 933 | cg16983588 | -0.0085 | 0.000298 | 11 | *PRDM10* | Body | TRUE | - |
| 934 | cg21308429 | -0.0036 | 0.000302 | 2 | *LIMS1* | Body | NA | - |
| 935 | cg15776315 | -0.0072 | 0.000302 | 1 | *-* | - | NA | - |
| 936 | cg02836313 | -0.0029 | 0.000303 | X | *NLGN4X* | 5'UTR | NA | - |
| 937 | cg26373518 | -0.0041 | 0.000305 | 22 | *INPP5J* | TSS200 | TRUE | - |
| 938 | cg22805381 | -0.0038 | 0.000305 | 14 | *-* | - | TRUE | - |
| 939 | cg09445550 | -0.006 | 0.000306 | 20 | *PC-* | 5'UTR | NA | N_Shore |
| 940 | cg13377102 | 0.0128 | 0.000306 | 17 | *KC-B3* | TSS200 | TRUE | Island |
| 941 | cg23884784 | -0.0028 | 0.000307 | 14 | *LTB4R* | 5'UTR | NA | N_Shelf |
| 942 | cg03641016 | 0.0022 | 0.000308 | 2 | *TET3* | 1stExon | NA | S_Shore |
| 943 | cg04666029 | -0.0015 | 0.000308 | 11 | *KCNQ1* | Body | TRUE | N_Shore |
| 944 | cg01966091 | 0.002 | 0.000308 | 16 | *HAS3* | 5'UTR | TRUE | Island |
| 945 | cg26395694 | -0.0042 | 0.000309 | 3 | *ITPR1* | Body | TRUE | - |
| 946 | cg12470493 | -0.004 | 0.000313 | 3 | *-* | - | NA | - |
| 947 | cg20222376 | -0.0038 | 0.000313 | 19 | *AKAP8L* | TSS1500 | TRUE | S_Shore |
| 948 | cg09363128 | 0.0028 | 0.000314 | 16 | *-* | - | TRUE | - |
| 949 | cg25209176 | -0.0045 | 0.000315 | 2 | *TCF7L1* | Body | NA | - |
| 950 | cg17998343 | -0.0034 | 0.000316 | 12 | *CRACR2A* | Body | NA | - |
| 951 | cg27553955 | 0.0031 | 0.000316 | 2 | *KCNG3* | 1stExon | TRUE | Island |
| 952 | cg08119150 | -0.0063 | 0.000316 | 7 | *RADIL* | Body | TRUE | N_Shore |
| 953 | cg11291879 | -0.0031 | 0.000316 | 20 | *-* | - | NA | - |
| 954 | cg14655917 | -0.0042 | 0.000316 | 7 | *-* | - | NA | - |
| 955 | cg15308037 | -0.004 | 0.000316 | 2 | *NPAS2* | Body | NA | - |
| 956 | cg01542019 | -0.0068 | 0.000317 | 19 | *TECR* | Body | TRUE | N_Shelf |
| 957 | cg07497042 | -0.0056 | 0.000317 | 1 | *EDARADD* | TSS1500 | NA | N_Shore |
| 958 | cg12556644 | -0.0026 | 0.000317 | 11 | *MIR3163* | TSS1500 | NA | - |
| 959 | cg05644947 | -0.0028 | 0.00032 | 3 | *-* | - | NA | - |
| 960 | cg07264586 | -0.0073 | 0.00032 | 8 | *-* | - | TRUE | - |
| 961 | cg12905221 | -0.0041 | 0.00032 | 16 | *-* | - | NA | - |
| 962 | cg13291298 | -0.0029 | 0.00032 | 15 | *PAQR5* | Body | NA | - |
| 963 | cg19624375 | 0.0028 | 0.000321 | 1 | *KP-6* | 3'UTR | NA | - |
| 964 | cg13407335 | 0.0145 | 0.000322 | 17 | *KC-B3* | TSS200 | TRUE | Island |
| 965 | cg24661245 | -0.0028 | 0.000323 | 20 | *LINC00176* | Body | NA | N_Shelf |
| 966 | cg03969996 | 0.0054 | 0.000323 | 19 | *PNMAL2* | 1stExon | TRUE | Island |
| 967 | cg07639287 | 0.0017 | 0.000325 | 22 | *-* | - | TRUE | S_Shore |
| 968 | cg13100137 | -0.004 | 0.000325 | 1 | *-* | - | TRUE | N_Shore |
| 969 | cg03751727 | -0.0064 | 0.000326 | 10 | *SLC16A12* | Body | NA | - |
| 970 | cg14209784 | -0.0047 | 0.000328 | 10 | *AGAP11* | TSS1500 | TRUE | N_Shore |
| 971 | cg09809672 | -0.0079 | 0.000328 | 1 | *EDARADD* | TSS1500 | TRUE | N_Shore |
| 972 | cg11868231 | -0.0043 | 0.000328 | 1 | *-* | - | NA | - |
| 973 | cg27642528 | -0.0029 | 0.000328 | 10 | *GLUD1* | Body | TRUE | - |
| 974 | cg10806711 | 0.0045 | 0.000332 | 1 | *FAM19A3* | Body | TRUE | Island |
| 975 | cg04834892 | -0.0025 | 0.000335 | 7 | *CTTNBP2* | Body | NA | - |
| 976 | cg15785594 | 0.0032 | 0.000337 | 11 | *FIBP* | Body | NA | N_Shelf |
| 977 | cg06259248 | 0.0023 | 0.000338 | 1 | *CIART* | 5'UTR | NA | S_Shore |
| 978 | cg11969213 | 0.0038 | 0.000343 | 17 | *LOC100133991* | TSS1500 | TRUE | N_Shore |
| 979 | cg21116474 | -0.0023 | 0.000343 | 10 | *-* | - | NA | - |
| 980 | cg20435325 | -0.006 | 0.000344 | 12 | *SLC16A7* | Body | NA | - |
| 981 | cg25912474 | -0.0133 | 0.000346 | 4 | *-* | - | NA | - |
| 982 | cg24205998 | 0.0052 | 0.000347 | 10 | *-* | - | NA | - |
| 983 | cg13663572 | -0.0048 | 0.000348 | 6 | *-* | - | NA | - |
| 984 | cg16653042 | -0.0027 | 0.000353 | 1 | *-* | - | NA | - |
| 985 | cg11811960 | 0.0033 | 0.000355 | 1 | *-* | - | NA | - |
| 986 | cg04004558 | 0.0047 | 0.000356 | 16 | *SOCS1* | 3'UTR | TRUE | Island |
| 987 | cg05321495 | -0.0053 | 0.000356 | 19 | *CHST8* | 5'UTR | NA | - |
| 988 | cg00982799 | -0.0025 | 0.000356 | 10 | *PTPRE* | Body | TRUE | - |
| 989 | cg01928659 | -0.0025 | 0.000356 | 10 | *-* | - | NA | S_Shore |
| 990 | cg03381616 | -0.011 | 0.000359 | 7 | *-* | - | NA | - |
| 991 | cg08743044 | 0.006 | 0.000359 | 16 | *SLC6A2* | Body | NA | S_Shore |
| 992 | cg25391820 | -0.0023 | 0.00036 | 5 | *MATR3* | TSS1500 | TRUE | N_Shore |
| 993 | cg04918548 | -0.0067 | 0.000364 | 1 | *-* | - | NA | - |
| 994 | cg07738259 | -0.0047 | 0.000366 | 1 | *ROR1* | Body | NA | - |
| 995 | cg06265072 | -0.0049 | 0.000367 | 15 | *ACTC1* | Body | NA | - |
| 996 | cg04602007 | -0.0032 | 0.00037 | 16 | *-* | - | TRUE | - |
| 997 | cg20786223 | -0.0034 | 0.00037 | 6 | *-* | - | NA | - |
| 998 | cg16660091 | -0.0131 | 0.000372 | 16 | *CETP* | 5'UTR | TRUE | - |
| 999 | cg13302154 | -0.0074 | 0.000373 | 12 | *MGP* | TSS1500 | TRUE | - |
| 1000 | cg04541799 | -0.003 | 0.000376 | 19 | *ZNF490* | Body | NA | - |
| 1001 | cg07208649 | 0.0026 | 0.000376 | 17 | *GLTPD2* | TSS200 | NA | N_Shore |
| 1002 | cg02394601 | -0.0047 | 0.000378 | 10 | *-* | - | NA | N_Shelf |
| 1003 | cg22022041 | 0.0036 | 0.000382 | 3 | *CCR9* | 5'UTR | TRUE | - |
| 1004 | cg06467327 | -0.0047 | 0.000382 | 9 | *-* | - | NA | N_Shore |
| 1005 | cg05418408 | -0.0029 | 0.000383 | 15 | *APBA2* | Body | NA | - |
| 1006 | cg16080273 | -0.0034 | 0.000383 | 10 | *-* | - | NA | S_Shore |
| 1007 | cg06313468 | -0.0035 | 0.000385 | 2 | *-* | - | NA | - |
| 1008 | cg14371212 | -0.0043 | 0.000387 | 3 | *MYH15* | Body | NA | - |
| 1009 | cg00687135 | 0.0076 | 0.000389 | 3 | *ZNF385D* | TSS200 | TRUE | - |
| 1010 | cg07695202 | -0.0045 | 0.000389 | 14 | *-* | - | NA | - |
| 1011 | cg16219535 | -0.004 | 0.00039 | 14 | *RPS29* | TSS1500 | TRUE | S_Shore |
| 1012 | cg13889950 | 0.0031 | 0.000395 | 3 | *-* | - | NA | - |
| 1013 | cg14028115 | -0.0044 | 0.000396 | 2 | *NOSTRIN* | TSS200 | NA | - |
| 1014 | cg08006730 | -0.0081 | 0.0004 | 19 | *-* | - | NA | - |
| 1015 | cg08804636 | 0.0028 | 0.0004 | 5 | *-* | - | NA | Island |
| 1016 | cg02590287 | -0.0015 | 0.000401 | X | *CLDN2* | 5'UTR | TRUE | - |
| 1017 | cg10439209 | -0.0041 | 0.000406 | 22 | *EIF3L* | Body | NA | - |
| 1018 | cg27138166 | -0.005 | 0.00041 | 4 | *LINC01088* | Body | NA | - |
| 1019 | cg14319127 | -0.003 | 0.000411 | 3 | *-* | - | NA | - |
| 1020 | cg17967818 | -0.0064 | 0.000411 | 6 | *-* | - | TRUE | - |
| 1021 | cg10488031 | 0.003 | 0.000414 | 13 | *IRS2* | 1stExon | TRUE | Island |
| 1022 | cg15851905 | -0.004 | 0.000414 | 4 | *11-Sep* | Body | NA | - |
| 1023 | cg21713473 | -0.0028 | 0.000414 | 4 | *-* | - | TRUE | N_Shore |
| 1024 | cg09317107 | -0.0022 | 0.000419 | 1 | *C1orf168* | Body | NA | - |
| 1025 | cg22221831 | -0.0072 | 0.000419 | 12 | *MGP* | TSS1500 | TRUE | - |
| 1026 | cg16325587 | -0.0044 | 0.000423 | 11 | *KIRREL3* | Body | NA | - |
| 1027 | cg19541622 | -0.0062 | 0.000423 | 4 | *ZNF718* | TSS1500 | NA | S_Shelf |
| 1028 | cg09143195 | -0.0097 | 0.000424 | 12 | *CNTN1* | TSS1500 | TRUE | N_Shore |
| 1029 | cg18268027 | -0.0043 | 0.000425 | 3 | *ITPR1* | Body | NA | - |
| 1030 | cg06997767 | -0.0073 | 0.000426 | 8 | *TRPS1* | Body | TRUE | - |
| 1031 | cg19856897 | 0.0068 | 0.000427 | 7 | *-* | - | TRUE | S_Shore |
| 1032 | cg16102930 | -0.0046 | 0.000428 | 17 | *4-Sep* | Body | NA | N_Shelf |
| 1033 | cg06507987 | 0.0039 | 0.000428 | 1 | *MAP3K6* | Body | TRUE | N_Shore |
| 1034 | cg05812200 | -0.006 | 0.000428 | 4 | *-* | - | NA | - |
| 1035 | cg10654598 | -0.0036 | 0.00043 | 1 | *KCNJ10* | 5'UTR | NA | - |
| 1036 | cg21890448 | 0.0045 | 0.00043 | 1 | *VANGL1* | Body | NA | - |
| 1037 | cg23459424 | 0.0032 | 0.00043 | 2 | *BRE* | 3'UTR | TRUE | - |
| 1038 | cg23715749 | -0.0048 | 0.00043 | 1 | *GRIK3* | Body | TRUE | - |
| 1039 | cg11603447 | -0.0034 | 0.000433 | 3 | *-* | - | NA | - |
| 1040 | cg19843425 | -0.0055 | 0.000433 | 6 | *-* | - | NA | - |
| 1041 | cg16775629 | -0.0029 | 0.000434 | 6 | *FOXO3* | Body | NA | - |
| 1042 | cg22366787 | 0.0031 | 0.000434 | 12 | *PITPNM2* | 5'UTR | NA | - |
| 1043 | cg05613083 | 0.0105 | 0.000438 | 16 | *KLHDC4* | Body | TRUE | N_Shelf |
| 1044 | cg17744295 | -0.0038 | 0.000438 | 19 | *TPM4* | TSS1500 | TRUE | N_Shore |
| 1045 | cg13756273 | -0.0034 | 0.000438 | 20 | *TUBB1* | 5'UTR | TRUE | - |
| 1046 | cg20824804 | -0.0041 | 0.000439 | 16 | *LOC101928708* | TSS1500 | NA | - |
| 1047 | cg04027111 | -0.0035 | 0.000439 | 10 | *LOXL4* | TSS1500 | NA | S_Shore |
| 1048 | cg27511678 | 0.0043 | 0.000439 | 18 | *MEX3C* | Body | TRUE | N_Shore |
| 1049 | cg10742957 | -0.0065 | 0.00044 | 19 | *FBXO17* | TSS1500 | TRUE | S_Shore |
| 1050 | cg25258518 | -0.0055 | 0.000441 | 7 | *-* | - | NA | - |
| 1051 | cg17538538 | -0.0029 | 0.000445 | 6 | *-* | - | NA | - |
| 1052 | cg11404915 | -0.0047 | 0.000446 | 12 | *-* | - | NA | - |
| 1053 | cg10734665 | 0.0052 | 0.000446 | 15 | *ATP10A* | Body | TRUE | N_Shore |
| 1054 | cg10740245 | -0.004 | 0.000446 | 17 | *-* | - | NA | - |
| 1055 | cg06711259 | -0.0029 | 0.000446 | 22 | *JOSD1* | 1stExon | TRUE | N_Shore |
| 1056 | cg06602546 | 0.0035 | 0.00045 | 20 | *SOX18* | Body | NA | Island |
| 1057 | cg25532677 | 0.0026 | 0.00045 | 12 | *DDX54* | Body | NA | - |
| 1058 | cg24023388 | -0.0031 | 0.00045 | 5 | *TTC33* | Body | NA | - |
| 1059 | cg19703023 | -0.0038 | 0.000451 | 17 | *-* | - | NA | - |
| 1060 | cg26914205 | -0.0039 | 0.000452 | 18 | *ADNP2* | TSS1500 | TRUE | - |
| 1061 | cg27412899 | -0.0052 | 0.000456 | 2 | *-* | - | NA | - |
| 1062 | cg23993283 | 0.0044 | 0.000456 | 14 | *WDR25* | Body | NA | - |
| 1063 | cg05741395 | -0.003 | 0.000459 | 15 | *SPTBN5* | Body | TRUE | N_Shore |
| 1064 | cg06119452 | -0.0107 | 0.00046 | 6 | *THBS2* | Body | TRUE | N_Shore |
| 1065 | cg13930219 | -0.0029 | 0.00046 | 1 | *-* | - | NA | - |
| 1066 | cg21625331 | -0.0046 | 0.00046 | 1 | *LINC01135* | Body | NA | - |
| 1067 | cg11299543 | -0.0097 | 0.000462 | 9 | *PDCD1LG2* | 1stExon | NA | - |
| 1068 | cg21541638 | 0.003 | 0.000464 | 3 | *FHIT* | 5'UTR | TRUE | N_Shore |
| 1069 | cg17052497 | -0.0052 | 0.000464 | 18 | *-* | - | NA | - |
| 1070 | cg26081188 | -0.0042 | 0.000464 | 11 | *0* | Body | NA | - |
| 1071 | cg08704764 | -0.0051 | 0.000474 | 12 | *-* | - | NA | - |
| 1072 | cg04100595 | -0.0055 | 0.000477 | 12 | *-* | - | TRUE | - |
| 1073 | cg18346273 | -0.0055 | 0.000481 | 5 | *POC5* | Body | NA | - |
| 1074 | cg26002422 | -0.0035 | 0.000482 | 19 | *-* | - | NA | - |
| 1075 | cg14684925 | -0.0069 | 0.000492 | 19 | *-* | - | TRUE | - |
| 1076 | cg10608615 | -0.0035 | 0.000492 | 5 | *KCNIP1* | TSS200 | TRUE | - |
| 1077 | cg00821310 | -0.0054 | 0.000493 | 1 | *ACOT7* | Body | TRUE | - |
| 1078 | cg00573770 | -0.0132 | 0.000493 | 2 | *ZEB2* | TSS1500 | TRUE | N_Shelf |
| 1079 | cg23166773 | -0.0043 | 0.000494 | 1 | *SDCCAG8* | Body | TRUE | - |
| 1080 | cg25331593 | -0.0046 | 0.000495 | 9 | *NRARP* | TSS1500 | NA | S_Shore |
| 1081 | cg04742397 | -0.0026 | 0.000499 | 14 | *-* | - | TRUE | S_Shelf |
| 1082 | cg09153462 | -0.0041 | 0.000499 | 8 | *ASPH* | Body | NA | - |
| 1083 | cg11220950 | 0.0034 | 0.000499 | 16 | *SYNGR3* | Body | TRUE | Island |
| 1084 | cg21449597 | -0.0029 | 0.000505 | 4 | *-* | - | TRUE | - |
| 1085 | cg11492723 | -0.0059 | 0.000508 | 3 | *-* | - | TRUE | - |
| 1086 | cg03979241 | -0.0054 | 0.00051 | 8 | *EPB49* | 1stExon | TRUE | S_Shelf |
| 1087 | cg09489811 | 0.0026 | 0.000518 | 4 | *ARHGEF38* | TSS1500 | NA | - |
| 1088 | cg18186343 | -0.0053 | 0.00052 | 14 | *MIR770* | TSS1500 | TRUE | - |
| 1089 | cg06623197 | -0.0034 | 0.000521 | 22 | *MTMR3* | Body | TRUE | - |
| 1090 | cg27162435 | 0.0128 | 0.000521 | 17 | *KC-B3* | TSS1500 | TRUE | Island |
| 1091 | cg01250446 | -0.0029 | 0.000524 | 16 | *-* | - | NA | - |
| 1092 | cg24866418 | 0.0023 | 0.000524 | 3 | *LHFPL4* | Body | NA | Island |
| 1093 | cg03140410 | 0.0049 | 0.000525 | 17 | *RHBDL3* | Body | NA | S_Shore |
| 1094 | cg12150097 | -0.0054 | 0.000527 | 4 | *-* | - | NA | - |
| 1095 | cg10970328 | -0.0036 | 0.000528 | 12 | *GPR162* | 5'UTR | TRUE | N_Shore |
| 1096 | cg15798153 | 0.0059 | 0.000533 | 7 | *CDK14* | 1stExon | TRUE | - |
| 1097 | cg18533870 | -0.0031 | 0.000533 | 11 | *SLC3A2* | 1stExon | NA | S_Shore |
| 1098 | cg06121226 | -0.0064 | 0.000533 | 4 | *SLC4A4* | Body | TRUE | - |
| 1099 | cg12460140 | -0.0126 | 0.000536 | 12 | *-* | - | NA | - |
| 1100 | cg17077319 | -0.0085 | 0.000537 | 3 | *-* | - | NA | - |
| 1101 | cg09428934 | -0.0043 | 0.000544 | 14 | *PCNX* | Body | NA | - |
| 1102 | cg13848598 | 0.0031 | 0.000547 | 10 | *ADRB1* | 1stExon | TRUE | Island |
| 1103 | cg05619598 | -0.0031 | 0.000548 | 19 | *-* | - | TRUE | N_Shelf |
| 1104 | cg11218872 | -0.0029 | 0.000548 | 3 | *-* | - | NA | S_Shore |
| 1105 | cg25236398 | -0.0056 | 0.000549 | 12 | *-* | - | NA | - |
| 1106 | cg05148931 | -0.0031 | 0.000551 | 22 | *-* | - | NA | - |
| 1107 | cg09801824 | -0.003 | 0.000553 | 10 | *-* | - | TRUE | N_Shelf |
| 1108 | cg07568841 | -0.0045 | 0.000553 | 7 | *ZNRF2* | Body | TRUE | - |
| 1109 | cg09312590 | -0.0038 | 0.000555 | 10 | *HECTD2AS1* | Body | NA | - |
| 1110 | cg03246461 | -0.0014 | 0.000555 | 19 | *-* | - | NA | - |
| 1111 | cg23950157 | -0.0031 | 0.000555 | 17 | *COL1A1* | Body | TRUE | N_Shore |
| 1112 | cg10989288 | 0.0034 | 0.000555 | 19 | *TMEM91* | Body | NA | S_Shore |
| 1113 | cg10604091 | 0.0036 | 0.00056 | 13 | *DLEU2* | Body | NA | - |
| 1114 | cg01369611 | -0.0056 | 0.000561 | 5 | *LOC101929710* | Body | NA | - |
| 1115 | cg25526547 | 0.0039 | 0.000561 | 12 | *MED13L* | Body | NA | - |
| 1116 | cg06913958 | -0.0031 | 0.000561 | 1 | *BCL10* | TSS1500 | TRUE | S_Shelf |
| 1117 | cg09863066 | -0.003 | 0.000566 | 22 | *PVALB* | 5'UTR | TRUE | S_Shore |
| 1118 | cg13873632 | -0.0032 | 0.000567 | 4 | *LDB2* | Body | TRUE | - |
| 1119 | cg10424974 | -0.0028 | 0.00057 | 16 | *-* | - | TRUE | - |
| 1120 | cg07004025 | -0.0052 | 0.000572 | 15 | *ARNT2* | Body | NA | - |
| 1121 | cg17516475 | -0.0026 | 0.000577 | 22 | *LINC00899* | Body | NA | Island |
| 1122 | cg17157422 | -0.0029 | 0.000587 | 12 | *CAC-1C* | Body | NA | - |
| 1123 | cg03928812 | -0.0044 | 0.000591 | 11 | *TCN1* | TSS1500 | TRUE | - |
| 1124 | cg05620847 | 0.0058 | 0.000591 | 6 | *BACH2* | Body | NA | - |
| 1125 | cg15053090 | -0.0032 | 0.000595 | 17 | *FLJ45079* | Body | NA | - |
| 1126 | cg11405195 | -0.0041 | 0.000595 | 10 | *-* | - | NA | - |
| 1127 | cg14358262 | -0.0049 | 0.000595 | 8 | *LOC101929315* | Body | NA | - |
| 1128 | cg23776628 | 0.0103 | 0.000596 | 1 | *HEYL* | Body | NA | - |
| 1129 | cg12220285 | 0.0044 | 0.000599 | 12 | *B4GALNT3* | Body | NA | - |
| 1130 | cg21586223 | -0.0048 | 0.000602 | 13 | *-* | - | TRUE | - |
| 1131 | cg26427492 | -0.0043 | 0.000602 | X | *-* | - | NA | - |
| 1132 | cg03726236 | -0.0034 | 0.000604 | 16 | *RABEP2* | Body | TRUE | N_Shore |
| 1133 | cg19080839 | 0.0051 | 0.000607 | 7 | *SMARCD3* | Body | TRUE | N_Shelf |
| 1134 | cg18992688 | -0.0026 | 0.000608 | 1 | *AVPR1B* | TSS1500 | TRUE | N_Shore |
| 1135 | cg07772733 | 0.004 | 0.000608 | 3 | *FOXP1* | 5'UTR | NA | - |
| 1136 | cg18982976 | 0.0071 | 0.000608 | 17 | *TANC2* | Body | NA | - |
| 1137 | cg16973808 | -0.0042 | 0.00061 | 18 | *B4GALT6* | Body | NA | - |
| 1138 | cg01918195 | 0.0038 | 0.000611 | 8 | *-* | - | NA | S_Shelf |
| 1139 | cg19254533 | -0.0041 | 0.000613 | 6 | *CPNE5* | Body | NA | - |
| 1140 | cg24871743 | 0.0046 | 0.000613 | 1 | *DIRAS3* | Body | TRUE | Island |
| 1141 | cg21571548 | 0.005 | 0.000613 | 2 | *DIRC3* | Body | NA | - |
| 1142 | cg01649837 | -0.0063 | 0.000614 | 19 | *NWD1* | TSS200 | NA | - |
| 1143 | cg08643824 | -0.0033 | 0.000616 | 11 | *LPXN* | 3'UTR | TRUE | - |
| 1144 | cg13208413 | -0.0036 | 0.000617 | 12 | *-* | - | TRUE | N_Shore |
| 1145 | cg20631720 | -0.0057 | 0.000617 | 7 | *-* | - | NA | - |
| 1146 | cg22320795 | -0.0061 | 0.000617 | 16 | *CLEC19A* | Body | NA | - |
| 1147 | cg04072670 | -0.0036 | 0.000617 | 11 | *-* | - | NA | - |
| 1148 | cg20274944 | -0.0038 | 0.000617 | 15 | *C15orf52* | TSS200 | NA | - |
| 1149 | cg24924051 | 0.0035 | 0.000617 | 3 | *CMTM8* | Body | TRUE | - |
| 1150 | cg19097880 | 0.003 | 0.000618 | 11 | *-* | - | TRUE | - |
| 1151 | cg04400489 | -0.0031 | 0.000619 | 18 | *DLGAP1* | Body | NA | - |
| 1152 | cg09783609 | -0.0035 | 0.000623 | 11 | *PRR5L* | 5'UTR | NA | - |
| 1153 | cg03032497 | 0.0026 | 0.000623 | 14 | *-* | - | TRUE | N_Shore |
| 1154 | cg08993878 | -0.0053 | 0.000627 | 12 | *-* | - | TRUE | - |
| 1155 | cg15262503 | -0.0048 | 0.000627 | 10 | *LOC101927964* | Body | NA | - |
| 1156 | cg04754956 | -0.005 | 0.000627 | 10 | *-* | - | NA | N_Shelf |
| 1157 | cg26621020 | -0.0052 | 0.000627 | 8 | *-* | - | NA | - |
| 1158 | cg14374913 | -0.0025 | 0.000628 | 1 | *FCN3* | Body | NA | S_Shelf |
| 1159 | cg07810884 | -0.0023 | 0.000631 | 19 | *TPM4* | TSS1500 | TRUE | N_Shore |
| 1160 | cg12513254 | -0.0054 | 0.000636 | 7 | *TPK1* | Body | NA | - |
| 1161 | cg26733897 | 0.0026 | 0.000638 | 17 | *RPTOR* | Body | TRUE | - |
| 1162 | cg19817104 | -0.004 | 0.000642 | 1 | *ZBTB41* | Body | NA | - |
| 1163 | cg14354161 | -0.0028 | 0.000643 | 7 | *HECW1* | Body | NA | - |
| 1164 | cg05922563 | -0.0048 | 0.000646 | 17 | *ATP6V0A1* | 5'UTR | TRUE | S_Shore |
| 1165 | cg15048069 | 0.005 | 0.000651 | 12 | *TPH2* | Body | NA | - |
| 1166 | cg05331856 | -0.0023 | 0.000652 | 13 | *-* | - | TRUE | - |
| 1167 | cg18204696 | -0.0055 | 0.000654 | 6 | *NOX3* | Body | NA | - |
| 1168 | cg24864397 | -0.0058 | 0.000659 | 7 | *GRB10* | Body | NA | - |
| 1169 | cg09738242 | 0.0033 | 0.000659 | 1 | *-* | - | NA | - |
| 1170 | cg13761004 | -0.0025 | 0.000664 | 20 | *PCED1A* | TSS1500 | NA | S_Shore |
| 1171 | cg14989815 | 0.0025 | 0.000665 | 1 | *-* | - | TRUE | - |
| 1172 | cg08587685 | -0.0033 | 0.000673 | 10 | *ABLIM1* | Body | NA | S_Shore |
| 1173 | cg03715305 | -0.003 | 0.000673 | 19 | *NDUFA11* | 3'UTR | TRUE | S_Shelf |
| 1174 | cg10076902 | -0.0031 | 0.000675 | 11 | *SYT8* | Body | TRUE | - |
| 1175 | cg18238078 | -0.0072 | 0.00068 | 4 | *-* | - | NA | - |
| 1176 | cg04603976 | -0.0028 | 0.00068 | 19 | *ZBTB7A* | Body | TRUE | N_Shore |
| 1177 | cg12445970 | -0.0057 | 0.000681 | 3 | *-* | - | NA | - |
| 1178 | cg17898760 | -0.0037 | 0.000681 | 10 | *LOC101926942* | Body | NA | - |
| 1179 | cg08176240 | -0.0037 | 0.000681 | 15 | *-* | - | NA | - |
| 1180 | cg10566581 | 0.0029 | 0.000682 | 20 | *TGIF2* | 3'UTR | TRUE | - |
| 1181 | cg20263741 | 0.0028 | 0.000684 | 2 | *RHBDD1* | Body | NA | - |
| 1182 | cg11333566 | -0.0037 | 0.000685 | 1 | *HMCN1* | Body | NA | - |
| 1183 | cg19386949 | -0.0064 | 0.00069 | 17 | *-* | - | NA | - |
| 1184 | cg03530962 | -0.005 | 0.000693 | 3 | *-* | - | TRUE | N_Shore |
| 1185 | cg00431114 | -0.003 | 0.000702 | 20 | *TTPAL* | TSS1500 | TRUE | N_Shore |
| 1186 | cg05348216 | 0.008 | 0.000702 | 6 | *PRSS16* | Body | TRUE | S_Shelf |
| 1187 | cg05380920 | -0.0076 | 0.000702 | 5 | *EDIL3* | Body | NA | - |
| 1188 | cg13641317 | 0.0045 | 0.000702 | 3 | *-* | - | TRUE | - |
| 1189 | cg02456062 | 0.0031 | 0.000705 | 17 | *-* | - | NA | S_Shore |
| 1190 | cg04105166 | -0.0042 | 0.000703 | 12 | *COPS7A* | Body | TRUE | S_Shelf |
| 1191 | cg04503319 | -0.013 | 0.000704 | 16 | *ANKRD11* | Body | TRUE | N_Shelf |
| 1192 | cg07547765 | 0.0123 | 0.000704 | 17 | *HOXB7* | Body | TRUE | Island |
| 1193 | cg01233392 | -0.004 | 0.000727 | 1 | *SYDE2* | Body | TRUE | N_Shore |
| 1194 | cg05738655 | -0.0032 | 0.000726 | 2 | *-* | - | NA | - |
| 1195 | cg07541020 | -0.0071 | 0.000725 | 10 | *ZNF239* | 5'UTR | TRUE | N_Shore |
| 1196 | cg16118387 | 0.0033 | 0.000727 | 1 | *C1orf133* | TSS1500 | TRUE | S_Shore |
| 1197 | cg25408172 | 0.0033 | 0.00073 | 1 | *-* | - | NA | N_Shore |
| 1198 | cg05657694 | -0.0101 | 0.000735 | 3 | *ADGRG7* | TSS1500 | NA | - |
| 1199 | cg17190891 | 0.0076 | 0.000735 | 7 | *SH2B2* | 3'UTR | TRUE | Island |
| 1200 | cg26550989 | -0.0044 | 0.000733 | 2 | *-* | - | NA | N_Shore |
| 1201 | cg01724838 | -0.0037 | 0.000741 | 8 | *-* | - | NA | - |
| 1202 | cg09248660 | -0.0037 | 0.000737 | 6 | *-* | - | TRUE | - |
| 1203 | cg05318142 | -0.0045 | 0.000746 | 2 | *ATOH8* | Body | TRUE | - |
| 1204 | cg16008966 | -0.0045 | 0.000745 | 1 | *-* | - | TRUE | - |
| 1205 | cg03172226 | -0.0038 | 0.000749 | 19 | *IRF3* | 3'UTR | TRUE | - |
| 1206 | cg23964606 | 0.0037 | 0.000754 | 4 | *LOC728175* | TSS200 | NA | - |
| 1207 | cg04457572 | -0.0034 | 0.000754 | 10 | *CDH23* | Body | TRUE | - |
| 1208 | cg14251252 | -0.0022 | 0.000754 | 6 | *-* | - | NA | - |
| 1209 | cg21691116 | -0.0041 | 0.000758 | 1 | *NDUFS2* | 5'UTR | TRUE | Island |
| 1210 | cg23163948 | 0.0031 | 0.000754 | 22 | *-* | - | NA | - |
| 1211 | cg26069719 | -0.0019 | 0.000754 | 3 | *TEX264* | Body | TRUE | - |
| 1212 | cg11071401 | 0.0036 | 0.000759 | 17 | *CAC-1G* | TSS1500 | TRUE | Island |
| 1213 | cg15949111 | 0.0034 | 0.000759 | 1 | *PPM1J* | Body | NA | S_Shelf |
| 1214 | cg27571201 | -0.0049 | 0.00076 | 4 | *ARHGAP24* | 5'UTR | NA | - |
| 1215 | cg20118553 | -0.0082 | 0.000763 | 13 | *STARD13* | 5'UTR | NA | - |
| 1216 | cg04021562 | -0.0023 | 0.000771 | 6 | *SLC44A4* | Body | TRUE | - |
| 1217 | cg09815261 | -0.0022 | 0.000771 | 11 | *OTOG* | Body | NA | - |
| 1218 | cg22133973 | -0.0063 | 0.000775 | 6 | *-* | - | TRUE | S_Shore |
| 1219 | cg16680942 | 0.0039 | 0.000781 | 17 | *RARAAS1* | TSS1500 | NA | N_Shore |
| 1220 | cg20982546 | -0.0066 | 0.00078 | 19 | *-* | - | NA | - |
| 1221 | cg24360230 | -0.0078 | 0.000781 | 12 | *CNTN1* | TSS1500 | NA | N_Shore |
| 1222 | cg24970172 | -0.0042 | 0.000781 | 14 | *-* | - | NA | - |
| 1223 | cg07222015 | -0.0046 | 0.000788 | 12 | *LINC00944* | Body | NA | - |
| 1224 | cg22483154 | -0.0023 | 0.000784 | 22 | *FBLN1* | Body | NA | - |
| 1225 | cg02606535 | 0.0046 | 0.000791 | 16 | *TSC2* | Body | NA | N_Shore |
| 1226 | cg24964298 | -0.0034 | 0.000791 | 1 | *-* | - | NA | - |
| 1227 | cg09728487 | -0.0048 | 0.000795 | 4 | *-* | - | TRUE | S_Shelf |
| 1228 | cg14381047 | -0.003 | 0.000799 | 7 | *MKLN1* | TSS1500 | NA | N_Shore |
| 1229 | cg22736354 | 0.0025 | 0.000799 | 6 | *NHLRC1* | 1stExon | TRUE | Island |
| 1230 | cg09094866 | -0.0043 | 0.0008 | 19 | *PIP5K1C* | TSS1500 | NA | S_Shore |
| 1231 | cg14850784 | 0.0062 | 0.0008 | 16 | *UNKL* | TSS1500 | TRUE | Island |
| 1232 | cg22234488 | -0.0042 | 0.0008 | 2 | *-* | - | NA | - |
| 1233 | cg08774778 | -0.0085 | 0.000806 | 5 | *-* | - | NA | - |
| 1234 | cg10261512 | 0.0023 | 0.000806 | 7 | *FOXK1* | Body | NA | - |
| 1235 | cg14918082 | 0.0148 | 0.000806 | 17 | *KC-B3* | TSS1500 | TRUE | N_Shore |
| 1236 | cg06821999 | 0.0019 | 0.000809 | 14 | *-* | - | TRUE | Island |
| 1237 | cg07169660 | -0.004 | 0.000811 | 17 | *LOC100130581* | TSS1500 | TRUE | - |
| 1238 | cg16276850 | 0.0027 | 0.000811 | 17 | *RARA* | 5'UTR | TRUE | Island |
| 1239 | cg19159381 | -0.0062 | 0.000811 | X | *PIN4* | Body | NA | - |
| 1240 | cg23560414 | -0.0042 | 0.000811 | 2 | *TFPI* | Body | NA | - |
| 1241 | cg01555198 | -0.0067 | 0.000815 | 13 | *BIVMERCC5* | Body | NA | - |
| 1242 | cg05175318 | 0.0027 | 0.000812 | 14 | *NRXN3* | Body | TRUE | S_Shore |
| 1243 | cg08575141 | 0.0062 | 0.000815 | 20 | *-* | - | NA | S_Shelf |
| 1244 | cg21940640 | -0.0053 | 0.000812 | 7 | *PHKG1* | TSS1500 | TRUE | - |
| 1245 | cg24862514 | 0.0017 | 0.000812 | 11 | *TP53I11* | TSS200 | TRUE | Island |
| 1246 | cg10276834 | -0.0064 | 0.000818 | 7 | *CHRM2* | 5'UTR | TRUE | - |
| 1247 | cg26669397 | -0.0044 | 0.000815 | 1 | *-* | - | TRUE | N_Shore |
| 1248 | cg24515883 | 0.0046 | 0.000823 | 2 | *-* | - | NA | - |
| 1249 | cg00213082 | -0.0045 | 0.000827 | 12 | *-* | - | NA | - |
| 1250 | cg14132585 | -0.0042 | 0.000827 | 5 | *LOC101929710* | Body | NA | - |
| 1251 | cg20351734 | -0.0045 | 0.000837 | X | *-* | - | NA | - |
| 1252 | cg00698916 | -0.0039 | 0.000838 | 6 | *-* | - | TRUE | N_Shore |
| 1253 | cg18168448 | 0.0048 | 0.000846 | 1 | *LRRC52* | 1stExon | TRUE | - |
| 1254 | cg21279804 | -0.0048 | 0.000847 | 2 | *-* | - | NA | N_Shelf |
| 1255 | cg22211173 | 0.0033 | 0.000846 | 17 | *NXN* | Body | NA | - |
| 1256 | cg27291126 | -0.0078 | 0.000845 | X | *DIAPH2* | Body | NA | - |
| 1257 | cg11669003 | -0.0044 | 0.000851 | 22 | *CELSR1* | Body | NA | - |
| 1258 | cg16513459 | 0.013 | 0.00085 | 17 | *KC-B3* | TSS200 | TRUE | Island |
| 1259 | cg26588779 | -0.0048 | 0.000848 | X | *MSN* | Body | NA | - |
| 1260 | cg02530533 | -0.0028 | 0.000856 | 8 | *LINC00534* | TSS200 | NA | - |
| 1261 | cg01750375 | -0.0057 | 0.000862 | 20 | *WISP2* | TSS1500 | TRUE | - |
| 1262 | cg09614261 | -0.0034 | 0.000862 | 13 | *-* | - | NA | - |
| 1263 | cg21064713 | -0.003 | 0.000857 | 11 | *PPP6R3* | 5'UTR | NA | - |
| 1264 | cg09905935 | -0.0078 | 0.000865 | 18 | *CABLES1* | TSS1500 | NA | N_Shore |
| 1265 | cg07128371 | -0.0019 | 0.000871 | 3 | *LSM3* | TSS1500 | NA | N_Shore |
| 1266 | cg21851496 | 0.0067 | 0.00087 | 8 | *SYBU* | 1stExon | NA | N_Shore |
| 1267 | cg03557417 | -0.0049 | 0.000874 | 14 | *NRXN3* | Body | NA | - |
| 1268 | cg12261786 | -0.0036 | 0.000874 | 10 | *C10orf116* | TSS1500 | TRUE | N_Shore |
| 1269 | cg23743591 | -0.0087 | 0.000877 | 14 | *-* | - | NA | - |
| 1270 | cg15261605 | -0.0014 | 0.000881 | 10 | *CDH23* | Body | TRUE | - |
| 1271 | cg17288636 | 0.0024 | 0.000878 | 8 | *-* | - | NA | - |
| 1272 | cg25322319 | -0.0047 | 0.000878 | 13 | *-* | - | NA | - |
| 1273 | cg06011204 | 0.005 | 0.000885 | 3 | *-* | - | NA | - |
| 1274 | cg12957013 | -0.0035 | 0.000883 | 20 | *FAM65C* | Body | NA | - |
| 1275 | cg14468077 | -0.0057 | 0.000883 | 9 | *PALM2* | Body | NA | - |
| 1276 | cg23322172 | 0.0064 | 0.000888 | 10 | *TMEM180* | Body | TRUE | - |
| 1277 | cg24718722 | 0.01 | 0.000889 | 10 | *FOXI2* | Body | TRUE | Island |
| 1278 | cg27015931 | -0.0017 | 0.000889 | 16 | *C16orf65* | 1stExon | TRUE | - |
| 1279 | cg16596294 | -0.0044 | 0.000892 | 10 | *EXOC6* | TSS1500 | NA | N_Shore |
| 1280 | cg18409356 | -0.0047 | 0.000893 | 5 | *-* | - | NA | - |
| 1281 | cg23074481 | 0.0049 | 0.000899 | 18 | *-* | - | NA | - |
| 1282 | cg26099158 | -0.0056 | 0.000897 | 12 | *-* | - | TRUE | - |
| 1283 | cg02222982 | -0.0049 | 0.000902 | 11 | *CORO1B* | Body | TRUE | S_Shelf |
| 1284 | cg04362997 | -0.0032 | 0.0009 | 12 | *DCTN2* | Body | NA | - |
| 1285 | cg17817168 | -0.0061 | 0.000905 | 12 | *ST8SIA1* | Body | TRUE | N_Shore |
| 1286 | cg25997480 | -0.0057 | 0.000907 | 10 | *-* | - | NA | - |
| 1287 | cg00417826 | -0.004 | 0.000914 | 9 | *GALNT12* | Body | NA | - |
| 1288 | cg19695041 | -0.0037 | 0.000913 | 8 | *TACC1* | 5'UTR | TRUE | S_Shore |
| 1289 | cg07872519 | -0.009 | 0.000918 | 7 | *ABCB1* | TSS1500 | NA | - |
| 1290 | cg12076562 | -0.0045 | 0.000917 | 2 | *-* | - | NA | - |
| 1291 | cg20193323 | -0.0047 | 0.000917 | 6 | *-* | - | NA | - |
| 1292 | cg02203380 | -0.0047 | 0.000937 | 7 | *C7orf41* | TSS1500 | TRUE | N_Shore |
| 1293 | cg16001165 | -0.004 | 0.000932 | 4 | *-* | - | TRUE | - |
| 1294 | cg02439189 | -0.0017 | 0.000937 | 20 | *UBOX5* | 1stExon | NA | - |
| 1295 | cg10052691 | -0.006 | 0.000937 | 3 | *ABI3BP* | Body | NA | - |
| 1296 | cg21548231 | -0.0052 | 0.000961 | 3 | *CMTM8* | Body | NA | - |
| 1297 | cg23459817 | -0.0045 | 0.000964 | 17 | *RAD51C* | Body | NA | - |
| 1298 | cg26600490 | -0.0033 | 0.000962 | 13 | *FOXO1* | Body | NA | - |
| 1299 | cg04860338 | -0.0042 | 0.000967 | 1 | *-* | - | NA | - |
| 1300 | cg06175101 | -0.0041 | 0.000967 | 1 | *ABCA4* | Body | NA | - |
| 1301 | cg07888917 | -0.0055 | 0.000967 | 2 | *-* | - | NA | - |
| 1302 | cg08079596 | -0.0031 | 0.000967 | 2 | *ATOH8* | Body | TRUE | - |
| 1303 | cg14097568 | -0.0031 | 0.000969 | 1 | *-* | - | TRUE | - |
| 1304 | cg00260802 | 0.0019 | 0.000972 | 20 | *-* | - | TRUE | Island |
| 1305 | cg08123705 | -0.0055 | 0.000973 | 2 | *-* | - | NA | - |
| 1306 | cg15936351 | -0.0043 | 0.000972 | X | *-* | - | NA | - |
| 1307 | cg01838523 | -0.0052 | 0.000976 | 20 | *WISP2* | TSS1500 | NA | - |
| 1308 | cg06791972 | 0.0029 | 0.000976 | 5 | *MAN2A1* | Body | NA | - |
| 1309 | cg13833437 | 0.0047 | 0.000976 | 2 | *UCN* | TSS200 | TRUE | Island |
| 1310 | cg00221718 | -0.0022 | 0.000987 | 3 | *-* | - | TRUE | - |
| 1311 | cg21017569 | 0.0119 | 0.000988 | 17 | *KC-B3* | TSS200 | TRUE | Island |
| 1312 | cg04789835 | -0.0023 | 0.000994 | 1 | *-* | - | NA | - |
| 1313 | cg06594010 | 0.0028 | 0.000993 | 2 | *HES6* | TSS1500 | NA | S_Shore |
| 1314 | cg10538119 | -0.0071 | 0.000995 | 15 | *DET1* | Body | NA | - |
| 1315 | cg14177184 | 0.0039 | 0.000995 | 22 | *MCAT* | Body | NA | - |
| 1316 | cg04332349 | 0.0034 | 0.001001 | 2 | *EPAS1* | Body | NA | - |
| 1317 | cg14323210 | -0.0035 | 0.001001 | 7 | *FASTK* | TSS1500 | TRUE | Island |
| 1318 | cg15160624 | 0.0103 | 0.001004 | 3 | *ZNF385D* | TSS1500 | NA | - |
| 1319 | cg05893843 | -0.0047 | 0.00101 | 5 | *PRR16* | Body | TRUE | - |
| 1320 | cg12628061 | -0.0114 | 0.001006 | 1 | *-* | - | TRUE | - |
| 1321 | cg07547549 | 0.0028 | 0.001012 | 20 | *SLC12A5* | Body | TRUE | Island |
| 1322 | cg15798350 | -0.0024 | 0.001014 | 16 | *ADCY7* | TSS1500 | NA | - |
| 1323 | cg27030854 | -0.0071 | 0.001014 | 12 | *0* | Body | NA | - |
| 1324 | cg04819580 | -0.0029 | 0.001014 | 7 | *-* | - | TRUE | - |
| 1325 | cg05884711 | 0.0041 | 0.001017 | 16 | *ATP6V0C* | TSS1500 | TRUE | N_Shore |
| 1326 | cg22423627 | -0.0063 | 0.001017 | 6 | *GFOD1* | Body | NA | - |
| 1327 | cg09509819 | -0.0076 | 0.001029 | 10 | *-* | - | NA | - |
| 1328 | cg08914310 | -0.0035 | 0.00103 | 13 | *IPO5* | 5'UTR | NA | - |
| 1329 | cg26746936 | 0.0024 | 0.00103 | 19 | *GRIK5* | Body | TRUE | Island |
| 1330 | cg27344140 | -0.0051 | 0.00103 | 3 | *-* | - | NA | - |
| 1331 | cg05324516 | -0.0043 | 0.001033 | 10 | *-* | - | TRUE | - |
| 1332 | cg11926456 | 0.004 | 0.001033 | 2 | *CPS1* | Body | TRUE | - |
| 1333 | cg23344436 | -0.0026 | 0.001033 | 22 | *PRR34AS1* | Body | NA | S_Shelf |
| 1334 | cg01445100 | 0.0032 | 0.001039 | 16 | *BANP* | Body | TRUE | S_Shore |
| 1335 | cg13001878 | -0.0035 | 0.001039 | 13 | *F10* | TSS200 | TRUE | - |
| 1336 | cg14556683 | 0.0043 | 0.001039 | 19 | *EPHX3* | 1stExon | TRUE | Island |
| 1337 | cg10886225 | -0.0059 | 0.001041 | 8 | *FUT10* | Body | TRUE | - |
| 1338 | cg15078085 | -0.0033 | 0.001043 | 7 | *-* | - | NA | - |
| 1339 | cg20018452 | -0.005 | 0.001041 | 5 | *SIL1* | Body | NA | - |
| 1340 | cg00062245 | 0.0036 | 0.001048 | 17 | *ITGA2B* | Body | TRUE | Island |
| 1341 | cg03208691 | -0.0035 | 0.001048 | 1 | *ADORA1* | Body | NA | - |
| 1342 | cg12436196 | -0.0035 | 0.001048 | 1 | *PRDM16* | Body | TRUE | N_Shore |
| 1343 | cg06093408 | -0.0041 | 0.001051 | 18 | *-* | - | NA | - |
| 1344 | cg12300603 | 0.0032 | 0.001048 | 15 | *-* | - | NA | - |
| 1345 | cg12727018 | -0.0035 | 0.00105 | 22 | *LOC100271722* | Body | TRUE | N_Shelf |
| 1346 | cg14798125 | -0.0081 | 0.001051 | 1 | *GPR52* | 1stExon | NA | - |
| 1347 | cg08951301 | -0.0051 | 0.001052 | 12 | *-* | - | NA | - |
| 1348 | cg11446602 | 0.0029 | 0.001052 | 3 | *BFSP2* | Body | NA | - |
| 1349 | cg19319125 | 0.0089 | 0.001052 | 16 | *CES1P1* | TSS1500 | NA | N_Shore |
| 1350 | cg00793342 | 0.0023 | 0.001058 | 11 | *ACY3* | Body | NA | - |
| 1351 | cg04413853 | 0.0046 | 0.001055 | 17 | *C17orf64* | TSS200 | TRUE | S_Shore |
| 1352 | cg06419432 | -0.0069 | 0.001055 | 17 | *-* | - | TRUE | - |
| 1353 | cg10066188 | -0.0032 | 0.001058 | 2 | *#NAME?* | 1stExon | TRUE | - |
| 1354 | cg08446924 | -0.004 | 0.001059 | 7 | *-* | - | NA | - |
| 1355 | cg22204103 | -0.007 | 0.001058 | 4 | *LIMCH1* | Body | TRUE | - |
| 1356 | cg20018782 | -0.0061 | 0.001063 | 14 | *-* | - | TRUE | S_Shelf |
| 1357 | cg01910639 | -0.0038 | 0.001068 | 1 | *S100A6* | Body | TRUE | N_Shore |
| 1358 | cg03864443 | -0.0062 | 0.001068 | 7 | *KCND2* | Body | NA | - |
| 1359 | cg06045838 | 0.0027 | 0.001068 | 17 | *TNFAIP1* | Body | NA | - |
| 1360 | cg07551820 | 0.0027 | 0.001068 | 1 | *-* | - | NA | - |
| 1361 | cg13215995 | -0.0031 | 0.001068 | 17 | *-* | - | TRUE | Island |
| 1362 | cg17646797 | -0.0033 | 0.001067 | 1 | *-* | - | NA | - |
| 1363 | cg27039866 | -0.004 | 0.001067 | 11 | *-* | - | NA | - |
| 1364 | cg03118800 | -0.0032 | 0.001069 | 17 | *EVPL* | Body | NA | Island |
| 1365 | cg17438696 | -0.0066 | 0.001069 | 1 | *-* | - | NA | - |
| 1366 | cg14188401 | -0.0033 | 0.001075 | 3 | *-* | - | TRUE | S_Shelf |
| 1367 | cg24690071 | 0.0022 | 0.001072 | 13 | *ZIC2* | 1stExon | TRUE | Island |
| 1368 | cg02946885 | -0.0042 | 0.001086 | 14 | *C14orf143* | Body | TRUE | - |
| 1369 | cg11667847 | 0.0031 | 0.001087 | 9 | *GRIN1* | 5'UTR | NA | Island |
| 1370 | cg01429333 | -0.0063 | 0.001088 | 7 | *C7orf50* | Body | TRUE | N_Shelf |
| 1371 | cg16097920 | -0.0042 | 0.001088 | 3 | *-* | - | NA | - |
| 1372 | cg18582010 | -0.0057 | 0.001089 | 8 | *ASAP1* | TSS1500 | TRUE | - |
| 1373 | cg13423729 | -0.005 | 0.001094 | 19 | *PAPL* | TSS1500 | NA | N_Shore |
| 1374 | cg05977193 | -0.0026 | 0.001098 | 20 | *LINC00176* | Body | NA | S_Shore |
| 1375 | cg24020235 | -0.0051 | 0.001096 | 8 | *LOC101929622* | TSS200 | NA | - |
| 1376 | cg24724428 | 0.0033 | 0.001097 | 6 | *ELOVL2* | TSS1500 | TRUE | Island |
| 1377 | cg08078742 | -0.0083 | 0.00112 | 12 | *LINC00507* | Body | NA | - |
| 1378 | cg18643262 | -0.0021 | 0.001118 | 20 | *-* | - | NA | - |
| 1379 | cg20774283 | -0.0034 | 0.001118 | 17 | *LINC01483* | Body | NA | - |
| 1380 | cg14293575 | 0.0047 | 0.001121 | 22 | *USP18* | 5'UTR | TRUE | S_Shelf |
| 1381 | cg01794929 | 0.008 | 0.001128 | 22 | *SELM* | 3'UTR | TRUE | Island |
| 1382 | cg07613178 | -0.0055 | 0.001129 | 4 | *RXFP1* | Body | NA | - |
| 1383 | cg12213064 | 0.0032 | 0.001132 | 2 | *BCL11A* | Body | NA | N_Shelf |
| 1384 | cg12419932 | 0.0042 | 0.001131 | 18 | *KIAA0427* | 5'UTR | TRUE | S_Shelf |
| 1385 | cg13524209 | -0.003 | 0.001133 | 12 | *LRRC23* | TSS1500 | TRUE | - |
| 1386 | cg15171452 | 0.0092 | 0.001146 | 5 | *SLC12A7* | Body | NA | N_Shore |
| 1387 | cg17403084 | -0.0034 | 0.001145 | 12 | *PXN* | TSS1500 | NA | S_Shore |
| 1388 | cg01634328 | -0.0044 | 0.001159 | 4 | *-* | - | NA | - |
| 1389 | cg11105339 | -0.0022 | 0.001155 | 9 | *-* | - | NA | - |
| 1390 | cg16432182 | -0.0055 | 0.001156 | 4 | *ABCG2* | TSS200 | NA | - |
| 1391 | cg01907945 | -0.0056 | 0.001159 | 3 | *CLRN1AS1* | Body | NA | - |
| 1392 | cg08632918 | -0.0036 | 0.001167 | 13 | *RAB20* | Body | NA | N_Shore |
| 1393 | cg10299585 | 0.0022 | 0.001167 | 12 | *-* | - | NA | Island |
| 1394 | cg13201644 | 0.0032 | 0.00117 | 15 | *STOML1* | Body | TRUE | - |
| 1395 | cg19673155 | -0.0035 | 0.00117 | 10 | *UNC5B* | Body | TRUE | - |
| 1396 | cg24774812 | -0.0025 | 0.00117 | 14 | *RHOJ* | Body | TRUE | - |
| 1397 | cg08705351 | -0.004 | 0.001179 | 1 | *EIF4G3* | 5'UTR | NA | - |
| 1398 | cg22042988 | -0.0024 | 0.00118 | 11 | *DSCAML1* | Body | NA | - |
| 1399 | cg25656315 | 0.0039 | 0.001179 | 1 | *-* | - | NA | - |
| 1400 | cg09430877 | 0.0026 | 0.001185 | 11 | *-* | - | NA | - |
| 1401 | cg04349839 | -0.0038 | 0.001186 | 4 | *SEL1L3* | Body | TRUE | - |
| 1402 | cg13021857 | -0.0052 | 0.001187 | 8 | *-* | - | TRUE | - |
| 1403 | cg03384000 | 0.0024 | 0.001201 | 17 | *#NAME?* | TSS200 | TRUE | N_Shore |
| 1404 | cg15055378 | -0.0033 | 0.001201 | 15 | *LCTL* | TSS1500 | NA | - |
| 1405 | cg04224661 | 0.0038 | 0.001206 | 11 | *-* | - | NA | - |
| 1406 | cg09954232 | -0.0021 | 0.001206 | 17 | *-* | - | NA | S_Shore |
| 1407 | cg13498289 | -0.0021 | 0.001206 | 6 | *BMP6* | Body | TRUE | Island |
| 1408 | cg20816447 | -0.0062 | 0.001206 | 4 | *CC2D2A* | Body | TRUE | - |
| 1409 | cg22809078 | 0.0031 | 0.001207 | 19 | *SMARCA4* | 5'UTR | NA | - |
| 1410 | cg16394551 | -0.0033 | 0.001211 | 1 | *PRDM16* | Body | TRUE | - |
| 1411 | cg25813319 | -0.0026 | 0.001222 | 4 | *FRYL* | Body | NA | - |
| 1412 | cg00403246 | -0.0029 | 0.001222 | 10 | *-* | - | NA | S_Shelf |
| 1413 | cg07402003 | -0.004 | 0.001225 | 16 | *TOX3* | Body | TRUE | - |
| 1414 | cg09834794 | 0.0015 | 0.001226 | 3 | *-* | - | TRUE | - |
| 1415 | cg14584292 | -0.0098 | 0.001234 | 11 | *MIR100HG* | Body | NA | - |
| 1416 | cg17142950 | -0.0051 | 0.001231 | 1 | *SAMD13* | TSS1500 | TRUE | S_Shore |
| 1417 | cg11361983 | -0.0022 | 0.001236 | 19 | *ALDH16A1* | TSS1500 | NA | N_Shore |
| 1418 | cg18902238 | -0.0049 | 0.00124 | 2 | *-* | - | NA | - |
| 1419 | cg02970086 | 0.0038 | 0.001247 | 20 | *SLA2* | 5'UTR | NA | - |
| 1420 | cg07415507 | 0.0022 | 0.001253 | 11 | *TMEM134* | TSS1500 | NA | S_Shore |
| 1421 | cg14129356 | -0.0037 | 0.001249 | 6 | *-* | - | NA | - |
| 1422 | cg10001287 | -0.004 | 0.001255 | 7 | *RELN* | Body | NA | - |
| 1423 | cg11731671 | -0.0067 | 0.001255 | 1 | *PRDM16* | Body | TRUE | N_Shore |
| 1424 | cg23769143 | -0.0072 | 0.001256 | 3 | *SYN2* | Body | TRUE | S_Shore |
| 1425 | cg04183873 | -0.0032 | 0.001258 | 20 | *-* | - | NA | - |
| 1426 | cg05405914 | -0.0036 | 0.001259 | 16 | *-* | - | TRUE | - |
| 1427 | cg21025494 | -0.0031 | 0.001258 | 7 | *IGF2BP3* | Body | TRUE | - |
| 1428 | cg02506717 | -0.0042 | 0.00126 | 7 | *CREB5* | TSS200 | TRUE | - |
| 1429 | cg26120073 | 0.0066 | 0.00126 | 14 | *CCDC88C* | Body | TRUE | - |
| 1430 | cg05689209 | -0.0022 | 0.00127 | 14 | *LRP10* | Body | NA | - |
| 1431 | cg12467090 | 0.0037 | 0.001273 | 1 | *PIK3C2B* | 5'UTR | TRUE | - |
| 1432 | cg01969701 | -0.0074 | 0.001275 | 11 | *-* | - | TRUE | Island |
| 1433 | cg03277268 | -0.0055 | 0.001274 | 7 | *-* | - | NA | - |
| 1434 | cg09978533 | -0.0036 | 0.001276 | 22 | *-* | - | TRUE | N_Shore |
| 1435 | cg11419186 | -0.0025 | 0.001276 | 16 | *CMIP* | TSS1500 | TRUE | N_Shore |
| 1436 | cg18635614 | -0.0053 | 0.001276 | 1 | *-* | - | NA | - |
| 1437 | cg04165428 | 0.0017 | 0.001284 | 19 | *RHPN2* | TSS1500 | NA | Island |
| 1438 | cg04255201 | 0.0026 | 0.001287 | 19 | *PNMAL2* | TSS200 | TRUE | S_Shore |
| 1439 | cg25190347 | 0.0024 | 0.001285 | 16 | *-* | - | NA | - |
| 1440 | cg27603605 | -0.0033 | 0.001287 | 12 | *TMEM132B* | Body | NA | - |
| 1441 | cg22566897 | -0.006 | 0.001295 | 10 | *-* | - | NA | - |
| 1442 | cg01792230 | -0.0095 | 0.001303 | 9 | *CDC14B* | Body | NA | - |
| 1443 | cg05646492 | -0.0037 | 0.001298 | 15 | *S-P23* | TSS1500 | NA | S_Shelf |
| 1444 | cg00906812 | -0.0025 | 0.001311 | 3 | *CCDC12* | TSS1500 | TRUE | S_Shore |
| 1445 | cg10625544 | 0.0025 | 0.001309 | 7 | *IKZF1* | Body | NA | - |
| 1446 | cg24068289 | -0.0034 | 0.001316 | 12 | *-* | - | NA | - |
| 1447 | cg13613450 | 0.0034 | 0.001325 | X | *-* | - | TRUE | S_Shore |
| 1448 | cg18813365 | -0.0043 | 0.001325 | 17 | *-* | - | NA | - |
| 1449 | cg20591472 | 0.0023 | 0.001328 | 1 | *SYPL2* | TSS200 | TRUE | Island |
| 1450 | cg23791229 | -0.0043 | 0.001328 | 7 | *CDK6* | Body | NA | - |
| 1451 | cg14345226 | -0.0043 | 0.001339 | 1 | *-* | - | NA | N_Shelf |
| 1452 | cg22947000 | -0.0059 | 0.001339 | 16 | *BCMO1* | TSS200 | TRUE | - |
| 1453 | cg14331923 | -0.0022 | 0.001344 | 11 | *-* | - | NA | - |
| 1454 | cg03761599 | 0.0032 | 0.001351 | 2 | *-* | - | NA | - |
| 1455 | cg14510445 | 0.0032 | 0.001366 | 11 | *-* | - | NA | - |
| 1456 | cg04745384 | 0.0032 | 0.001377 | 12 | *-* | - | TRUE | Island |
| 1457 | cg24069172 | -0.003 | 0.001375 | 11 | *PRSS23* | Body | NA | - |
| 1458 | cg25268605 | -0.0042 | 0.001378 | 1 | *TAL1* | TSS1500 | NA | S_Shore |
| 1459 | cg00956907 | -0.0061 | 0.001378 | 2 | *-* | - | NA | - |
| 1460 | cg06627827 | -0.0055 | 0.00138 | 1 | *-* | - | NA | - |
| 1461 | cg07400692 | -0.0101 | 0.001378 | 20 | *LOC101929608* | Body | NA | - |
| 1462 | cg20205586 | 0.004 | 0.001384 | 2 | *SRSF7* | TSS1500 | NA | S_Shore |
| 1463 | cg07662935 | -0.0023 | 0.001389 | 1 | *CAMTA1* | Body | NA | - |
| 1464 | cg02364122 | 0.0027 | 0.001398 | 11 | *PHLDB1* | Body | NA | - |
| 1465 | cg06991974 | 0.0037 | 0.001396 | 1 | *FLJ42875* | Body | TRUE | Island |
| 1466 | cg07755896 | -0.0014 | 0.001402 | 2 | *-* | - | NA | - |
| 1467 | cg23287894 | -0.0059 | 0.001402 | 4 | *-* | - | NA | - |
| 1468 | cg23656415 | -0.0093 | 0.001402 | 3 | *-* | - | NA | - |
| 1469 | cg02685435 | -0.0034 | 0.001411 | 7 | *IQCE* | TSS1500 | NA | N_Shore |
| 1470 | cg10163536 | -0.011 | 0.001411 | 4 | *-* | - | NA | - |
| 1471 | cg16604233 | -0.0058 | 0.001411 | 6 | *-* | - | TRUE | N_Shore |
| 1472 | cg05127193 | -0.0031 | 0.001411 | 7 | *-* | - | NA | - |
| 1473 | cg12950368 | 0.0043 | 0.001411 | 6 | *PACSIN1* | 5'UTR | NA | - |
| 1474 | cg15324190 | -0.0046 | 0.001411 | 5 | *-* | - | NA | - |
| 1475 | cg22426570 | 0.0033 | 0.001411 | 7 | *CHRM2* | 5'UTR | TRUE | S_Shore |
| 1476 | cg27625479 | 0.0043 | 0.001411 | 7 | *FEZF1* | Body | NA | N_Shore |
| 1477 | cg09080576 | 0.004 | 0.001411 | 9 | *RAPGEF1* | Body | NA | - |
| 1478 | cg12203247 | -0.0043 | 0.001414 | 11 | *-* | - | NA | - |
| 1479 | cg04710700 | -0.0097 | 0.00142 | 15 | *ADAMTSL3* | Body | NA | - |
| 1480 | cg06072257 | -0.0033 | 0.001421 | 1 | *-* | - | NA | - |
| 1481 | cg01279933 | 0.0021 | 0.001426 | 19 | *ATG4D* | 3'UTR | TRUE | - |
| 1482 | cg10803903 | -0.005 | 0.001428 | 1 | *PLOD1* | Body | TRUE | - |
| 1483 | cg18645241 | -0.0079 | 0.001428 | 21 | *-* | - | NA | - |
| 1484 | cg08935887 | -0.0039 | 0.001434 | 2 | *-* | - | NA | - |
| 1485 | cg20801278 | -0.0063 | 0.001434 | 2 | *-* | - | NA | - |
| 1486 | cg21756451 | -0.0074 | 0.00144 | 4 | *C4orf45* | Body | NA | - |
| 1487 | cg01228357 | -0.0038 | 0.001443 | 2 | *-* | - | NA | - |
| 1488 | cg04260591 | -0.0087 | 0.001442 | 9 | *-* | - | NA | - |
| 1489 | cg00697290 | -0.0086 | 0.001446 | 3 | *-* | - | TRUE | N_Shelf |
| 1490 | cg22556485 | -0.0031 | 0.001444 | 5 | *ADGRV1* | Body | NA | - |
| 1491 | cg03947688 | 0.0022 | 0.001448 | 5 | *ANKRD34B* | TSS200 | NA | Island |
| 1492 | cg16273343 | 0.002 | 0.001448 | 5 | *PAM* | Body | NA | S_Shore |
| 1493 | cg20287562 | -0.0052 | 0.001448 | 10 | *-* | - | NA | - |
| 1494 | cg23395688 | -0.0053 | 0.001451 | 12 | *-* | - | TRUE | - |
| 1495 | cg09879253 | 0.0047 | 0.001461 | 17 | *-* | - | TRUE | - |
| 1496 | cg01211283 | 0.004 | 0.001466 | 8 | *PEBP4* | Body | TRUE | - |
| 1497 | cg05159099 | 0.0042 | 0.001465 | 2 | *MAL* | Body | NA | - |
| 1498 | cg21174512 | 0.0028 | 0.001466 | 11 | *HTR3A* | TSS1500 | NA | - |
| 1499 | cg09416908 | -0.0027 | 0.00147 | 11 | *ME3* | TSS1500 | TRUE | S_Shore |
| 1500 | cg23718736 | -0.0059 | 0.00147 | 18 | *L3MBTL4* | 5'UTR | TRUE | N_Shore |
| 1501 | cg12024104 | -0.0025 | 0.001477 | 12 | *IFFO1* | Body | TRUE | N_Shore |
| 1502 | cg20816917 | -0.0041 | 0.001477 | 4 | *FGB* | Body | NA | - |
| 1503 | cg19896563 | -0.0055 | 0.001485 | 3 | *-* | - | NA | - |
| 1504 | cg04090392 | 0.0017 | 0.001495 | 15 | *BNC1* | Body | TRUE | Island |
| 1505 | cg08511485 | -0.0033 | 0.001495 | 3 | *MECOM* | Body | TRUE | - |
| 1506 | cg17796461 | 0.003 | 0.001495 | 15 | *-* | - | TRUE | Island |
| 1507 | cg19356022 | -0.0029 | 0.001508 | 1 | *SHC1* | 5'UTR | TRUE | N_Shelf |
| 1508 | cg23824239 | -0.005 | 0.001513 | 18 | *DT-* | 5'UTR | NA | - |
| 1509 | cg24093698 | -0.0086 | 0.001516 | 11 | *NTM* | Body | NA | - |
| 1510 | cg09291026 | -0.0025 | 0.001522 | 14 | *C14orf145* | Body | TRUE | - |
| 1511 | cg11619216 | -0.0027 | 0.001522 | 17 | *LOC100130933* | TSS1500 | TRUE | - |
| 1512 | cg22788465 | -0.0029 | 0.001525 | 5 | *IL3* | 3'UTR | TRUE | - |
| 1513 | cg10203610 | -0.0029 | 0.001529 | 4 | *LRAT* | TSS1500 | TRUE | Island |
| 1514 | cg15086543 | -0.0065 | 0.001535 | 1 | *-* | - | NA | - |
| 1515 | cg18302606 | 0.0033 | 0.001535 | 19 | *PLEKHG2* | 5'UTR | TRUE | Island |
| 1516 | cg20210410 | 0.0036 | 0.001535 | 2 | *C2orf80* | Body | NA | - |
| 1517 | cg24775076 | -0.0029 | 0.001539 | 10 | *OR13A1* | 5'UTR | NA | - |
| 1518 | cg06537454 | 0.0026 | 0.001548 | 9 | *-* | - | NA | - |
| 1519 | cg07324393 | -0.0028 | 0.001548 | 4 | *-* | - | NA | - |
| 1520 | cg13679679 | -0.0044 | 0.00155 | 3 | *CMC1* | TSS1500 | TRUE | N_Shore |
| 1521 | cg14665825 | 0.0022 | 0.001548 | 17 | *SSH2* | Body | TRUE | - |
| 1522 | cg15546227 | -0.0029 | 0.001549 | 1 | *NFIA* | Body | TRUE | N_Shelf |
| 1523 | cg24674215 | -0.0031 | 0.001548 | 13 | *-* | - | TRUE | - |
| 1524 | cg03451833 | 0.0031 | 0.001554 | 5 | *LOC100287592* | Body | NA | S_Shore |
| 1525 | cg12892303 | 0.0024 | 0.001556 | 17 | *C17orf104* | TSS200 | TRUE | Island |
| 1526 | cg17740900 | -0.0049 | 0.001556 | 14 | *-* | - | NA | - |
| 1527 | cg03054432 | -0.0044 | 0.00156 | 1 | *-* | - | NA | S_Shore |
| 1528 | cg12032603 | -0.005 | 0.00156 | 12 | *SH2B3* | TSS1500 | NA | - |
| 1529 | cg07382588 | -0.003 | 0.001563 | 15 | *CTDSPL2* | 5'UTR | NA | - |
| 1530 | cg08634797 | 0.0022 | 0.001565 | 11 | *YPEL4* | 5'UTR | NA | S_Shore |
| 1531 | cg27443377 | -0.0051 | 0.001565 | 3 | *TMEM108* | 1stExon | NA | - |
| 1532 | cg27653724 | -0.0037 | 0.001567 | 13 | *PCDH9* | Body | NA | - |
| 1533 | cg02079985 | -0.004 | 0.001567 | 3 | *ST3GAL6* | TSS1500 | NA | N_Shore |
| 1534 | cg02512168 | -0.0065 | 0.001569 | 16 | *ZNF205* | 5'UTR | TRUE | - |
| 1535 | cg07633256 | -0.0065 | 0.001569 | 5 | *TENM2* | Body | NA | - |
| 1536 | cg25537245 | -0.0029 | 0.001572 | 17 | *-* | - | TRUE | - |
| 1537 | cg27470213 | -0.0041 | 0.001573 | 17 | *LGALS3BP* | Body | TRUE | - |
| 1538 | cg03759346 | -0.0062 | 0.001579 | 3 | *-* | - | TRUE | - |
| 1539 | cg18805621 | -0.0035 | 0.001578 | 4 | *CLNK* | Body | NA | - |
| 1540 | cg07790318 | -0.0015 | 0.001584 | 20 | *-* | - | NA | - |
| 1541 | cg17309085 | -0.0034 | 0.001584 | 11 | *CNTN5* | Body | NA | - |
| 1542 | cg19467605 | -0.0077 | 0.001584 | 11 | *PIWIL4* | Body | NA | - |
| 1543 | cg11078736 | -0.0044 | 0.001586 | 7 | *-* | - | NA | - |
| 1544 | cg25129960 | -0.0108 | 0.001587 | 12 | *-* | - | NA | - |
| 1545 | cg02529485 | 0.0042 | 0.001589 | 2 | *AGAP1* | Body | NA | - |
| 1546 | cg05216984 | 0.0017 | 0.001602 | 2 | *PPP1R7* | Body | NA | - |
| 1547 | cg03569876 | 0.0035 | 0.001608 | 5 | *CSNK1A1* | Body | TRUE | - |
| 1548 | cg15530026 | -0.0033 | 0.001608 | 18 | *LOXHD1* | TSS1500 | NA | S_Shore |
| 1549 | cg12065779 | -0.0029 | 0.001621 | 10 | *-* | - | TRUE | N_Shore |
| 1550 | cg18443579 | -0.0022 | 0.001626 | 9 | *LRRC8A* | Body | NA | S_Shelf |
| 1551 | cg22414262 | -0.0047 | 0.001626 | 21 | *MIR802* | TSS200 | TRUE | - |
| 1552 | cg07504763 | -0.0036 | 0.001628 | 1 | *-* | - | TRUE | - |
| 1553 | cg12990614 | 0.0044 | 0.001633 | 22 | *SOX10* | Body | TRUE | Island |
| 1554 | cg10278475 | -0.0035 | 0.001637 | 15 | *RORA* | Body | NA | - |
| 1555 | cg27363529 | 0.0034 | 0.001637 | 11 | *SLC35C1* | TSS200 | TRUE | N_Shore |
| 1556 | cg07776980 | -0.0036 | 0.001644 | 13 | *FRY* | Body | NA | - |
| 1557 | cg09624551 | -0.0063 | 0.001649 | 3 | *-* | - | TRUE | - |
| 1558 | cg27278639 | -0.0059 | 0.001655 | 5 | *CDH18* | Body | NA | - |
| 1559 | cg05944249 | 0.0051 | 0.001658 | 8 | *SCARA3* | Body | NA | S_Shelf |
| 1560 | cg16636772 | 0.0024 | 0.001659 | 2 | *-* | - | NA | Island |
| 1561 | cg04999637 | -0.0054 | 0.001661 | 17 | *-* | - | TRUE | - |
| 1562 | cg14888636 | 0.0021 | 0.001661 | 1 | *MAN1C1* | Body | NA | S_Shore |
| 1563 | cg19509778 | 0.0111 | 0.001665 | 10 | *FOXI2* | 3'UTR | TRUE | Island |
| 1564 | cg27090964 | -0.0065 | 0.001663 | 18 | *-* | - | NA | - |
| 1565 | cg04959747 | -0.0026 | 0.001674 | 3 | *-* | - | NA | - |
| 1566 | cg14391437 | -0.0082 | 0.001678 | 9 | *TRPM3* | Body | NA | - |
| 1567 | cg24418447 | -0.0033 | 0.00168 | 17 | *-* | - | NA | - |
| 1568 | cg14134497 | 0.002 | 0.001684 | 18 | *DT-* | 5'UTR | TRUE | Island |
| 1569 | cg18853017 | -0.0027 | 0.001686 | 17 | *-* | - | NA | - |
| 1570 | cg10107473 | -0.0043 | 0.001686 | 2 | *EPAS1* | Body | TRUE | - |
| 1571 | cg13466600 | -0.0032 | 0.001686 | X | *-* | - | NA | - |
| 1572 | cg15705175 | -0.0021 | 0.001686 | 10 | *-* | - | TRUE | N_Shore |
| 1573 | cg09006543 | -0.0039 | 0.001688 | 13 | *FRY* | Body | NA | - |
| 1574 | cg18177875 | 0.0017 | 0.001688 | 22 | *MGC16703* | Body | TRUE | N_Shelf |
| 1575 | cg22906032 | -0.0034 | 0.001688 | 1 | *LPGAT1* | TSS1500 | NA | S_Shore |
| 1576 | cg02238388 | 0.0021 | 0.001698 | 1 | *SYPL2* | TSS200 | NA | Island |
| 1577 | cg06297012 | -0.0052 | 0.001702 | 7 | *-* | - | NA | - |
| 1578 | cg14901671 | 0.0013 | 0.001701 | 17 | *-* | - | TRUE | Island |
| 1579 | cg10531975 | -0.0027 | 0.001703 | 5 | *-* | - | NA | - |
| 1580 | cg00210007 | -0.0065 | 0.001706 | 1 | *ESRRG* | TSS1500 | NA | - |
| 1581 | cg05954062 | -0.0026 | 0.001706 | 19 | *-* | - | NA | S_Shelf |
| 1582 | cg17113707 | -0.0035 | 0.001708 | 13 | *SKA3* | TSS1500 | NA | S_Shore |
| 1583 | cg21962791 | -0.0047 | 0.001716 | 12 | *PYROXD1* | TSS1500 | TRUE | N_Shore |
| 1584 | cg04386130 | -0.0047 | 0.001718 | 3 | *KC-B1* | Body | NA | - |
| 1585 | cg21515349 | 0.0013 | 0.001719 | 17 | *#NAME?* | TSS200 | TRUE | Island |
| 1586 | cg00676294 | -0.0092 | 0.001724 | 8 | *-* | - | NA | - |
| 1587 | cg13066481 | -0.0028 | 0.001724 | 3 | *MYLK* | Body | TRUE | - |
| 1588 | cg11851792 | 0.0037 | 0.001725 | 6 | *-* | - | TRUE | - |
| 1589 | cg21218370 | -0.0027 | 0.001731 | 4 | *GABRB1* | Body | NA | - |
| 1590 | cg25405211 | 0.0026 | 0.001737 | 4 | *LINC01093* | Body | NA | - |
| 1591 | cg02441694 | 0.0047 | 0.001744 | 2 | *-* | - | NA | - |
| 1592 | cg05823563 | -0.0047 | 0.001741 | 9 | *C9orf125* | TSS1500 | TRUE | S_Shore |
| 1593 | cg13424363 | -0.0031 | 0.001744 | 4 | *-* | - | NA | - |
| 1594 | cg26962579 | -0.0055 | 0.001744 | 7 | *-* | - | NA | - |
| 1595 | cg02570063 | -0.0031 | 0.001746 | 1 | *-* | - | NA | - |
| 1596 | cg11299964 | -0.0021 | 0.001746 | 9 | *MAPKAP1* | TSS1500 | TRUE | S_Shore |
| 1597 | cg04826825 | -0.0025 | 0.001754 | 22 | *MGAT3* | 5'UTR | NA | - |
| 1598 | cg07443704 | -0.0064 | 0.001754 | 1 | *-* | - | NA | - |
| 1599 | cg10819350 | -0.0037 | 0.001754 | 19 | *ATG4D* | Body | TRUE | S_Shore |
| 1600 | cg16598508 | -0.0038 | 0.001761 | 16 | *-* | - | TRUE | - |
| 1601 | cg00034101 | 0.0041 | 0.001765 | 1 | *HEYL* | Body | TRUE | - |
| 1602 | cg14854355 | 0.0045 | 0.001765 | 17 | *SARM1* | Body | TRUE | Island |
| 1603 | cg01438090 | -0.0066 | 0.001769 | 11 | *MPPED2* | Body | TRUE | - |
| 1604 | cg02992887 | -0.0037 | 0.001774 | 1 | *C1orf21* | 5'UTR | NA | - |
| 1605 | cg24845165 | 0.0043 | 0.001775 | 20 | *LPIN3* | TSS1500 | TRUE | N_Shore |
| 1606 | cg20529923 | -0.0022 | 0.001785 | 11 | *-* | - | TRUE | N_Shelf |
| 1607 | cg01802062 | -0.0052 | 0.001789 | 16 | *PAGR1* | 3'UTR | NA | - |
| 1608 | cg21024922 | -0.0049 | 0.001792 | 18 | *-* | - | NA | - |
| 1609 | cg01021271 | -0.0062 | 0.001792 | 11 | *-* | - | NA | - |
| 1610 | cg26232308 | 0.0031 | 0.001794 | 1 | *-* | - | TRUE | - |
| 1611 | cg17478888 | 0.0031 | 0.001795 | 16 | *MBTPS1* | Body | NA | - |
| 1612 | cg18150280 | -0.0045 | 0.001799 | 1 | *RGS2* | TSS1500 | TRUE | N_Shore |
| 1613 | cg15480367 | 0.0018 | 0.001803 | 14 | *CHGA* | 5'UTR | TRUE | Island |
| 1614 | cg16146033 | -0.0028 | 0.001803 | 11 | *SLC22A8* | Body | TRUE | - |
| 1615 | cg20449670 | -0.0038 | 0.001803 | 1 | *-* | - | TRUE | - |
| 1616 | cg01749651 | 0.0031 | 0.001804 | 8 | *NCR-00051* | TSS200 | TRUE | - |
| 1617 | cg18014251 | -0.0025 | 0.001804 | 6 | *-* | - | NA | - |
| 1618 | cg05444038 | -0.0058 | 0.001811 | 4 | *-* | - | NA | - |
| 1619 | cg21145523 | -0.0045 | 0.001817 | 8 | *FDFT1* | Body | NA | - |
| 1620 | cg21783228 | -0.0035 | 0.001817 | 3 | *TPRG1* | Body | NA | - |
| 1621 | cg23114828 | 0.0043 | 0.001835 | 18 | *-* | - | NA | - |
| 1622 | cg26742280 | -0.0049 | 0.001838 | 1 | *EDARADD* | TSS1500 | NA | N_Shore |
| 1623 | cg03696346 | -0.0015 | 0.001848 | 10 | *ZNF485* | TSS200 | TRUE | N_Shore |
| 1624 | cg02227986 | -0.0041 | 0.001853 | 2 | *-* | - | NA | - |
| 1625 | cg00379415 | -0.0044 | 0.001861 | 1 | *KCNT2* | Body | NA | - |
| 1626 | cg00808170 | 0.005 | 0.001861 | 5 | *PCDHGA4* | Body | TRUE | Island |
| 1627 | cg08025812 | -0.0021 | 0.001871 | 6 | *-* | - | NA | - |
| 1628 | cg11017186 | -0.0066 | 0.001871 | 13 | *-* | - | NA | - |
| 1629 | cg14186558 | -0.0027 | 0.001872 | 9 | *RNF183* | TSS1500 | TRUE | - |
| 1630 | cg03771840 | 0.0036 | 0.00188 | 6 | *TRIM15* | 3'UTR | TRUE | Island |
| 1631 | cg11939713 | -0.0043 | 0.00188 | 21 | *-* | - | NA | - |
| 1632 | cg05235590 | 0.0026 | 0.001883 | 3 | *SMARCC1* | TSS1500 | NA | S_Shore |
| 1633 | cg08473326 | -0.0022 | 0.001882 | 2 | *-* | - | NA | - |
| 1634 | cg10924811 | 0.0024 | 0.001883 | 6 | *-* | - | NA | - |
| 1635 | cg11529819 | -0.0025 | 0.001883 | 1 | *FCN3* | 3'UTR | TRUE | S_Shelf |
| 1636 | cg23766254 | 0.0096 | 0.00189 | 17 | *FAM171A2* | Body | TRUE | Island |
| 1637 | cg01836835 | -0.0037 | 0.001894 | 9 | *-* | - | NA | - |
| 1638 | cg03443986 | -0.0029 | 0.001893 | 2 | *-* | - | TRUE | - |
| 1639 | cg01154508 | 0.0025 | 0.001906 | 14 | *-* | - | NA | S_Shore |
| 1640 | cg16119613 | -0.004 | 0.001908 | 12 | *-* | - | TRUE | N_Shelf |
| 1641 | cg05279738 | -0.0053 | 0.001921 | 3 | *-* | - | TRUE | - |
| 1642 | cg11508999 | -0.0029 | 0.001922 | 4 | *C4orf45* | Body | NA | - |
| 1643 | cg12143126 | 0.006 | 0.001922 | 22 | *SGSM1* | Body | NA | - |
| 1644 | cg12938127 | 0.0037 | 0.001922 | 13 | *-* | - | NA | - |
| 1645 | cg01369725 | 0.0021 | 0.001925 | 19 | *UHRF1* | 5'UTR | NA | Island |
| 1646 | cg23244545 | -0.0076 | 0.001928 | 18 | *CDH2* | Body | NA | - |
| 1647 | cg24218885 | -0.0024 | 0.001943 | 3 | *ECE2* | 1stExon | NA | - |
| 1648 | cg02340851 | -0.002 | 0.001947 | 1 | *ANP32E* | Body | TRUE | N_Shore |
| 1649 | cg17288471 | -0.0024 | 0.001947 | 2 | *-* | - | TRUE | - |
| 1650 | cg24778195 | -0.0033 | 0.001947 | 14 | *-* | - | NA | - |
| 1651 | cg22512670 | -0.0038 | 0.001948 | 1 | *RPS6KA1* | TSS1500 | TRUE | N_Shore |
| 1652 | cg24707573 | 0.008 | 0.001948 | 7 | *SH2B2* | Body | TRUE | Island |
| 1653 | cg11155434 | 0.0043 | 0.001952 | 11 | *ETS1* | Body | NA | - |
| 1654 | cg22982767 | -0.0024 | 0.001951 | 22 | *LOC150381* | Body | TRUE | N_Shelf |
| 1655 | cg21126595 | -0.0034 | 0.001955 | 3 | *-* | - | NA | - |
| 1656 | cg21748401 | 0.0017 | 0.001956 | 3 | *CASR* | 1stExon | NA | Island |
| 1657 | cg09983216 | 0.0101 | 0.001957 | 17 | *LOC404266* | Body | TRUE | - |
| 1658 | cg13214605 | -0.0035 | 0.001957 | 13 | *-* | - | NA | - |
| 1659 | cg05463027 | -0.0042 | 0.001967 | 6 | *KIF13A* | TSS1500 | TRUE | S_Shore |
| 1660 | cg09224452 | 0.0028 | 0.001981 | 20 | *-* | - | NA | - |
| 1661 | cg10112270 | -0.0063 | 0.001981 | 1 | *-* | - | TRUE | - |
| 1662 | cg13776906 | -0.0053 | 0.001987 | 20 | *APMAP* | TSS1500 | NA | S_Shore |
| 1663 | cg21452942 | -0.0031 | 0.001987 | 17 | *CCDC40* | Body | TRUE | - |
| 1664 | cg26209441 | -0.0058 | 0.001986 | 1 | *VPS13D* | Body | NA | - |
| 1665 | cg00755846 | -0.0018 | 0.001988 | 22 | *-* | - | NA | - |
| 1666 | cg03132729 | -0.0046 | 0.001988 | 1 | *RAP1GAP* | Body | TRUE | N_Shore |
| 1667 | cg09824749 | -0.0038 | 0.001988 | 2 | *-* | - | NA | - |
| 1668 | cg15377518 | -0.0031 | 0.001988 | 2 | *ZEB2* | TSS200 | TRUE | N_Shelf |
| 1669 | cg06302240 | -0.0029 | 0.001995 | 10 | *-* | - | NA | - |
| 1670 | cg24445466 | 0.003 | 0.001997 | 8 | *-* | - | NA | - |
| 1671 | cg07187827 | -0.0033 | 0.002003 | 9 | *NRARP* | TSS1500 | NA | S_Shore |
| 1672 | cg19222574 | -0.0044 | 0.002021 | 3 | *PLX-1* | TSS1500 | TRUE | N_Shore |
| 1673 | cg20665157 | 0.006 | 0.002029 | 7 | *CADPS2* | Body | TRUE | - |
| 1674 | cg00156995 | 0.0035 | 0.00203 | 13 | *LOC646982* | TSS1500 | TRUE | - |
| 1675 | cg13411656 | -0.0019 | 0.002029 | 3 | *ZBTB38* | 5'UTR | TRUE | Island |
| 1676 | cg14180029 | -0.0034 | 0.002029 | 11 | *LDLRAD3* | Body | TRUE | - |
| 1677 | cg19567415 | -0.0053 | 0.002039 | 17 | *TANC2* | Body | NA | - |
| 1678 | cg20489179 | 0.0034 | 0.002044 | 20 | *CDC25B* | TSS1500 | NA | S_Shelf |
| 1679 | cg13984563 | 0.0029 | 0.002045 | 14 | *SERPI-5* | TSS200 | TRUE | - |
| 1680 | cg04923375 | 0.0027 | 0.002048 | 20 | *ZNF337* | 1stExon | NA | - |
| 1681 | cg06735626 | -0.0069 | 0.002062 | 20 | *FAM65C* | Body | NA | - |
| 1682 | cg12277313 | 0.003 | 0.002062 | 3 | *NME6* | Body | NA | N_Shore |
| 1683 | cg01998039 | 0.0045 | 0.002069 | 20 | *DOK5* | 5'UTR | NA | - |
| 1684 | cg11330941 | 0.0028 | 0.002069 | 2 | *GEN1* | TSS1500 | TRUE | Island |
| 1685 | cg01901101 | 0.0045 | 0.002072 | 1 | *-* | - | TRUE | N_Shore |
| 1686 | cg18203859 | -0.0028 | 0.002069 | 1 | *CYB5R1* | Body | NA | N_Shore |
| 1687 | cg01244663 | -0.0076 | 0.002073 | 13 | *-* | - | NA | - |
| 1688 | cg16945428 | 0.0032 | 0.002073 | 20 | *-* | - | NA | - |
| 1689 | cg01531262 | -0.0036 | 0.002087 | 3 | *MRPS22* | TSS1500 | NA | N_Shore |
| 1690 | cg20535646 | -0.0016 | 0.002087 | 2 | *CNGA3* | Body | TRUE | - |
| 1691 | cg20918505 | 0.0045 | 0.00209 | 9 | *GTF3C4* | Body | NA | S_Shelf |
| 1692 | cg04803208 | 0.0025 | 0.002091 | 8 | *LOC100130298* | Body | NA | - |
| 1693 | cg15949714 | -0.004 | 0.002091 | 10 | *-* | - | NA | - |
| 1694 | cg00634475 | -0.003 | 0.002094 | 11 | *MAML2* | Body | NA | - |
| 1695 | cg24748262 | -0.0019 | 0.002094 | 12 | *-* | - | TRUE | - |
| 1696 | cg12108113 | -0.0027 | 0.002096 | 1 | *-* | - | NA | - |
| 1697 | cg04600297 | -0.0039 | 0.002106 | 19 | *-* | - | TRUE | S_Shore |
| 1698 | cg27130665 | -0.0029 | 0.002104 | 11 | *LRRC4C* | TSS1500 | TRUE | - |
| 1699 | cg16449837 | -0.0039 | 0.002122 | 12 | *-* | - | NA | - |
| 1700 | cg14955982 | -0.0089 | 0.002152 | 7 | *CALN1* | 5'UTR | NA | - |
| 1701 | cg04925748 | 0.0031 | 0.00216 | 2 | *ABI2* | 5'UTR | NA | S_Shore |
| 1702 | cg19151852 | 0.0087 | 0.00216 | 17 | *ASPSCR1* | Body | NA | S_Shelf |
| 1703 | cg16989340 | -0.0022 | 0.002171 | 1 | *-* | - | TRUE | - |
| 1704 | cg07018856 | -0.0069 | 0.002173 | 18 | *CDH2* | Body | NA | - |
| 1705 | cg00752121 | -0.003 | 0.002175 | 2 | *-* | - | NA | - |
| 1706 | cg03047995 | 0.0032 | 0.002177 | 14 | *FITM1* | Body | TRUE | Island |
| 1707 | cg12499963 | -0.003 | 0.002178 | 19 | *-* | - | NA | S_Shelf |
| 1708 | cg25746849 | -0.0042 | 0.002178 | 3 | *-* | - | NA | - |
| 1709 | cg09292826 | -0.0044 | 0.002178 | 1 | *TCTEX1D4* | TSS1500 | NA | S_Shore |
| 1710 | cg15491247 | -0.0068 | 0.002178 | 1 | *PRDM16* | Body | TRUE | - |
| 1711 | cg17990814 | -0.0049 | 0.002178 | 2 | *SLC25A12* | Body | NA | - |
| 1712 | cg04525074 | -0.0024 | 0.002187 | 15 | *NOX5* | Body | NA | - |
| 1713 | cg00280235 | -0.0046 | 0.002209 | 7 | *NRCAM* | 1stExon | TRUE | - |
| 1714 | cg06097236 | 0.0015 | 0.002209 | 5 | *ARHGAP26* | 3'UTR | NA | - |
| 1715 | cg10469774 | 0.0048 | 0.002213 | 11 | *-* | - | TRUE | - |
| 1716 | cg26922780 | -0.0039 | 0.002214 | 16 | *RNF166* | Body | TRUE | N_Shelf |
| 1717 | cg04326808 | -0.0044 | 0.002216 | 15 | *C15orf54* | 1stExon | TRUE | - |
| 1718 | cg17554862 | -0.0022 | 0.002217 | 2 | *CFLAR* | 5'UTR | NA | - |
| 1719 | cg10576216 | -0.0032 | 0.002218 | 1 | *ZNF697* | TSS1500 | NA | S_Shore |
| 1720 | cg10728994 | 0.0036 | 0.002219 | 14 | *RIN3* | Body | NA | - |
| 1721 | cg07456172 | 0.0026 | 0.002221 | 22 | *SGSM1* | Body | NA | - |
| 1722 | cg08578119 | -0.0188 | 0.002233 | 1 | *-* | - | NA | - |
| 1723 | cg19951006 | 0.0048 | 0.002235 | 12 | *IL23A* | TSS200 | TRUE | - |
| 1724 | cg14772651 | -0.0096 | 0.002253 | 2 | *ZNF804A* | Body | NA | - |
| 1725 | cg08355702 | -0.0034 | 0.00226 | 2 | *-* | - | TRUE | - |
| 1726 | cg02566861 | 0.0085 | 0.002268 | 17 | *LOC404266* | Body | TRUE | S_Shelf |
| 1727 | cg08957484 | 0.0054 | 0.002273 | 5 | *CCNI2* | 1stExon | TRUE | Island |
| 1728 | cg08864105 | -0.0039 | 0.002274 | 1 | *DENND2D* | 1stExon | TRUE | N_Shelf |
| 1729 | cg23656533 | -0.0028 | 0.002274 | 14 | *KHNYN* | 1stExon | NA | S_Shelf |
| 1730 | cg03553587 | 0.0025 | 0.002279 | 3 | *-* | - | TRUE | Island |
| 1731 | cg15361162 | -0.0069 | 0.002279 | 6 | *PLAGL1* | 5'UTR | NA | - |
| 1732 | cg23004527 | 0.0025 | 0.002279 | 19 | *NFIC* | Body | TRUE | S_Shelf |
| 1733 | cg24261673 | 0.0068 | 0.002279 | 16 | *SLC6A2* | TSS200 | NA | N_Shore |
| 1734 | cg06237102 | -0.0116 | 0.002282 | 12 | *-* | - | NA | - |
| 1735 | cg24185062 | -0.0028 | 0.002284 | 7 | *CTTNBP2* | Body | NA | - |
| 1736 | cg04959790 | -0.0033 | 0.002293 | 11 | *NR1H3* | 5'UTR | TRUE | - |
| 1737 | cg23289235 | -0.0037 | 0.002293 | 5 | *CDC42SE2* | Body | NA | - |
| 1738 | cg00186909 | -0.0037 | 0.002299 | 12 | *PLEKHG7* | TSS1500 | TRUE | - |
| 1739 | cg05921699 | 0.003 | 0.002299 | 19 | *CD79A* | TSS1500 | TRUE | - |
| 1740 | cg27172413 | -0.0026 | 0.002299 | 6 | *SYNE1* | Body | NA | - |
| 1741 | cg02157398 | -0.0044 | 0.002312 | 21 | *LINC00313* | TSS200 | NA | - |
| 1742 | cg01034993 | -0.0021 | 0.002317 | 15 | *CORO2B* | Body | TRUE | - |
| 1743 | cg19308985 | -0.0034 | 0.002315 | 16 | *IRF8* | Body | NA | - |
| 1744 | cg05746957 | -0.0031 | 0.002328 | 18 | *-* | - | NA | - |
| 1745 | cg19344626 | -0.0041 | 0.002328 | 19 | *NWD1* | TSS200 | TRUE | - |
| 1746 | cg19513876 | -0.003 | 0.002338 | 17 | *-* | - | TRUE | N_Shelf |
| 1747 | cg06963130 | -0.004 | 0.002355 | 2 | *-* | - | NA | - |
| 1748 | cg12140144 | 0.0043 | 0.002365 | 1 | *FLJ42875* | Body | TRUE | Island |
| 1749 | cg19818923 | -0.0022 | 0.002365 | 19 | *-* | - | NA | - |
| 1750 | cg20547770 | -0.0066 | 0.002365 | 4 | *-* | - | NA | - |
| 1751 | cg10000705 | -0.0025 | 0.002376 | 1 | *-* | - | NA | - |
| 1752 | cg01577707 | -0.0062 | 0.002376 | 11 | *MIR100HG* | Body | NA | - |
| 1753 | cg10797387 | -0.0024 | 0.002376 | 14 | *INF2* | TSS1500 | TRUE | N_Shore |
| 1754 | cg08665685 | 0.0042 | 0.00238 | 22 | *SMTN* | Body | TRUE | Island |
| 1755 | cg19799745 | -0.0039 | 0.00239 | 2 | *GCC2* | Body | NA | - |
| 1756 | cg08140019 | 0.0043 | 0.002395 | 15 | *AGBL1* | Body | NA | - |
| 1757 | cg13683374 | -0.0034 | 0.002397 | 17 | *GPR142* | Body | TRUE | N_Shelf |
| 1758 | cg03242507 | -0.0057 | 0.002398 | 15 | *-* | - | NA | - |
| 1759 | cg10004888 | -0.0032 | 0.002398 | 8 | *STK3* | Body | NA | - |
| 1760 | cg07430714 | -0.004 | 0.002399 | 13 | *FAM124A* | Body | NA | - |
| 1761 | cg16540658 | -0.0034 | 0.002399 | 5 | *GFRA3* | TSS1500 | NA | S_Shore |
| 1762 | cg04771042 | 0.0037 | 0.002405 | 8 | *SORBS3* | 5'UTR | NA | S_Shelf |
| 1763 | cg13696706 | 0.0035 | 0.002405 | 9 | *DAB2IP* | Body | TRUE | - |
| 1764 | cg25527554 | -0.0025 | 0.002405 | 20 | *-* | - | NA | - |
| 1765 | cg26685941 | -0.0066 | 0.002405 | 13 | *ABCC4* | Body | TRUE | N_Shore |
| 1766 | cg08794478 | -0.0049 | 0.002406 | 7 | *ITGB8* | Body | NA | - |
| 1767 | cg21332930 | -0.0024 | 0.002413 | 1 | *PPFIA4* | 3'UTR | NA | S_Shore |
| 1768 | cg24016523 | -0.0039 | 0.002413 | 12 | *LOC100506551* | Body | NA | - |
| 1769 | cg08507334 | -0.0021 | 0.002415 | 13 | *-* | - | NA | - |
| 1770 | cg15963913 | -0.0029 | 0.002418 | 14 | *ZFYVE21* | Body | TRUE | S_Shore |
| 1771 | cg01199321 | -0.0038 | 0.002436 | 5 | *LCP2* | Body | NA | - |
| 1772 | cg22087119 | -0.0029 | 0.00244 | 3 | *ADGRG7* | TSS1500 | NA | - |
| 1773 | cg00766416 | -0.003 | 0.00244 | 13 | *-* | - | NA | - |
| 1774 | cg20620484 | -0.0044 | 0.002456 | 1 | *MTHFR* | Body | NA | - |
| 1775 | cg10727474 | -0.002 | 0.002458 | 11 | *LSP1* | TSS1500 | NA | - |
| 1776 | cg02587602 | 0.0023 | 0.002462 | 11 | *-* | - | TRUE | Island |
| 1777 | cg27646205 | -0.0031 | 0.002464 | 19 | *CCDC151* | Body | TRUE | N_Shore |
| 1778 | cg13226308 | -0.0047 | 0.002473 | 11 | *-* | - | TRUE | - |
| 1779 | cg22652747 | -0.0048 | 0.002473 | 15 | *MEIS2* | Body | NA | - |
| 1780 | cg09685196 | -0.003 | 0.002474 | 15 | *-* | - | NA | - |
| 1781 | cg24065451 | -0.0076 | 0.002475 | 11 | *ETS1* | Body | TRUE | Island |
| 1782 | cg24578623 | -0.0028 | 0.002499 | 2 | *PNKD* | Body | NA | - |
| 1783 | cg19715771 | 0.0017 | 0.002509 | 17 | *CBX4* | Body | TRUE | N_Shore |
| 1784 | cg20509525 | -0.0037 | 0.002512 | 22 | *FBLN1* | Body | NA | - |
| 1785 | cg14454381 | 0.0024 | 0.002512 | 10 | *ABLIM1* | Body | NA | - |
| 1786 | cg23722274 | 0.0029 | 0.002513 | 10 | *DNTT* | TSS200 | TRUE | - |
| 1787 | cg16203865 | -0.0034 | 0.002519 | 19 | *LOC105372441* | Body | NA | N_Shelf |
| 1788 | cg24496375 | -0.004 | 0.00252 | 2 | *-* | - | NA | - |
| 1789 | cg08191690 | 0.0027 | 0.002521 | 7 | *GTF2IRD1* | 5'UTR | NA | - |
| 1790 | cg07690222 | -0.0157 | 0.002526 | 12 | *TMTC2* | Body | NA | - |
| 1791 | cg11026820 | -0.008 | 0.002533 | 9 | *ENG* | Body | NA | - |
| 1792 | cg23918953 | -0.0031 | 0.002533 | 12 | *-* | - | TRUE | - |
| 1793 | cg04195684 | 0.0024 | 0.002545 | 22 | *MLC1* | Body | TRUE | Island |
| 1794 | cg00940560 | -0.006 | 0.00255 | 8 | *MBOAT4* | 1stExon | TRUE | - |
| 1795 | cg22584802 | -0.0025 | 0.00256 | 7 | *AHCYL2* | Body | TRUE | - |
| 1796 | cg01856384 | -0.0089 | 0.002563 | 6 | *-* | - | TRUE | Island |
| 1797 | cg07151445 | -8.00E-04 | 0.002569 | 8 | *-* | - | TRUE | Island |
| 1798 | cg11366363 | -0.0024 | 0.002572 | 13 | *GUCY1B2* | TSS1500 | TRUE | - |
| 1799 | cg10551832 | -0.0033 | 0.002573 | 14 | *-* | - | NA | - |
| 1800 | cg25694915 | -0.0032 | 0.002573 | 2 | *NOSTRIN* | 5'UTR | TRUE | - |
| 1801 | cg09391697 | -0.0047 | 0.002575 | 12 | *-* | - | NA | - |
| 1802 | cg05584950 | -0.0042 | 0.002581 | 2 | *ATOH8* | Body | TRUE | - |
| 1803 | cg07337598 | 0.0029 | 0.002581 | 1 | *ANXA9* | TSS1500 | TRUE | - |
| 1804 | cg09619064 | -0.0038 | 0.002585 | 2 | *GAD1* | Body | NA | - |
| 1805 | cg20012537 | -0.0036 | 0.002589 | 10 | *GPR123* | Body | TRUE | S_Shore |
| 1806 | cg21283720 | -0.0034 | 0.002588 | 17 | *MIR497HG* | Body | NA | N_Shelf |
| 1807 | cg02970255 | -0.0023 | 0.002593 | 2 | *-* | - | NA | S_Shelf |
| 1808 | cg10553109 | -0.0035 | 0.002593 | 12 | *-* | - | NA | - |
| 1809 | cg13942826 | -0.0034 | 0.002593 | 12 | *PRR4* | TSS1500 | TRUE | - |
| 1810 | cg16811453 | -0.0028 | 0.002593 | 4 | *ARHGEF38* | Body | NA | - |
| 1811 | cg00449997 | -0.0045 | 0.002599 | 12 | *UBE3B* | Body | NA | - |
| 1812 | cg06813554 | -0.0031 | 0.002597 | 14 | *ABHD4* | TSS1500 | TRUE | N_Shore |
| 1813 | cg07517675 | -0.003 | 0.002609 | 14 | *-* | - | NA | - |
| 1814 | cg14642193 | 0.0049 | 0.002618 | 9 | *-* | - | NA | - |
| 1815 | cg22079827 | -0.0029 | 0.002622 | 1 | *SYT6* | Body | TRUE | - |
| 1816 | cg09478021 | 0.0014 | 0.002622 | 19 | *CACNG7* | Body | NA | - |
| 1817 | cg24759016 | -0.0029 | 0.002622 | 4 | *LOC101929095* | Body | NA | - |
| 1818 | cg04069273 | -0.0033 | 0.002629 | 20 | *-* | - | NA | - |
| 1819 | cg14317608 | -0.0036 | 0.002629 | 1 | *NME7* | Body | NA | - |
| 1820 | cg19345895 | -0.0024 | 0.002643 | 1 | *MYBPHL* | Body | NA | - |
| 1821 | cg22929506 | -0.0044 | 0.002643 | 2 | *PNKD* | 3'UTR | TRUE | S_Shelf |
| 1822 | cg26624021 | -0.0104 | 0.002643 | 16 | *CETP* | TSS200 | TRUE | - |
| 1823 | cg25938039 | -0.0026 | 0.002644 | 2 | *-* | - | NA | - |
| 1824 | cg05306305 | -0.0045 | 0.002658 | 3 | *-* | - | NA | - |
| 1825 | cg14676592 | 0.002 | 0.002658 | 16 | *-* | - | TRUE | Island |
| 1826 | cg10103850 | -0.0029 | 0.002668 | 2 | *SLC8A1* | TSS1500 | TRUE | - |
| 1827 | cg02129236 | -0.007 | 0.002668 | 14 | *-* | - | NA | - |
| 1828 | cg21567504 | 0.0018 | 0.002668 | 15 | *OCA2* | TSS200 | TRUE | Island |
| 1829 | cg21781784 | -0.0043 | 0.002668 | 1 | *-* | - | NA | - |
| 1830 | cg19631762 | -0.0025 | 0.002668 | 1 | *-* | - | TRUE | - |
| 1831 | cg05848656 | -0.0026 | 0.002676 | 11 | *CREB3L1* | TSS1500 | NA | N_Shore |
| 1832 | cg14069287 | -0.0043 | 0.002676 | 4 | *PALLD* | Body | TRUE | - |
| 1833 | cg01807026 | -0.0036 | 0.002676 | 9 | *MEGF9* | Body | TRUE | N_Shore |
| 1834 | cg25360297 | 0.0025 | 0.002676 | 10 | *CCAR1* | Body | NA | - |
| 1835 | cg13837071 | 0.0025 | 0.002685 | 8 | *-* | - | NA | - |
| 1836 | cg23248055 | -0.0055 | 0.002689 | 4 | *ZNF718* | TSS1500 | NA | S_Shelf |
| 1837 | cg08086996 | -0.0038 | 0.00269 | 19 | *CCDC114* | TSS1500 | NA | N_Shore |
| 1838 | cg08358620 | -0.0097 | 0.002691 | 12 | *RASSF9* | TSS200 | NA | - |
| 1839 | cg08419476 | 0.0038 | 0.002704 | 11 | *TEAD1* | 5'UTR | NA | - |
| 1840 | cg06949673 | -0.0062 | 0.002714 | 1 | *-* | - | TRUE | - |
| 1841 | cg03963837 | -0.0043 | 0.002721 | 12 | *BCL2L14* | 3'UTR | NA | - |
| 1842 | cg08368885 | 0.0024 | 0.002728 | 9 | *ZBTB43* | Body | NA | S_Shore |
| 1843 | cg03848406 | -0.0055 | 0.002732 | 13 | *-* | - | NA | - |
| 1844 | cg19958586 | 0.0019 | 0.002737 | 3 | *CCRL2* | 5'UTR | NA | - |
| 1845 | cg27423177 | -0.0044 | 0.002739 | 12 | *C3AR1* | TSS1500 | TRUE | - |
| 1846 | cg06159782 | 0.0025 | 0.002749 | 9 | *-* | - | NA | - |
| 1847 | cg00776917 | 0.0046 | 0.00275 | 14 | *-* | - | TRUE | - |
| 1848 | cg16199747 | -0.0032 | 0.00275 | 6 | *-* | - | TRUE | - |
| 1849 | cg17504742 | -0.0034 | 0.00275 | 22 | *TTC28* | Body | NA | - |
| 1850 | cg18589742 | -0.004 | 0.002755 | 19 | *NFIX* | TSS1500 | NA | N_Shore |
| 1851 | cg03670162 | -0.0029 | 0.002762 | 12 | *-* | - | TRUE | N_Shore |
| 1852 | cg18899220 | 0.0029 | 0.002762 | 13 | *-* | - | TRUE | Island |
| 1853 | cg18964582 | -0.0042 | 0.002762 | 1 | *EDARADD* | TSS1500 | TRUE | N_Shore |
| 1854 | cg19878363 | -0.004 | 0.002769 | 13 | *-* | - | NA | - |
| 1855 | cg20239639 | 0.0035 | 0.00277 | 1 | *LCK* | 5'UTR | TRUE | N_Shore |
| 1856 | cg06655861 | -0.0028 | 0.002774 | 16 | *JPH3* | Body | TRUE | - |
| 1857 | cg19693191 | -0.0021 | 0.002776 | 1 | *ERI3* | Body | NA | - |
| 1858 | cg05251389 | 0.0051 | 0.002795 | 22 | *BIK* | 3'UTR | TRUE | - |
| 1859 | cg15071067 | 0.0035 | 0.002794 | 2 | *DGUOKAS1* | Body | NA | - |
| 1860 | cg07442943 | -0.004 | 0.002796 | 4 | *CLNK* | Body | NA | - |
| 1861 | cg13716020 | -0.005 | 0.002801 | 4 | *-* | - | NA | - |
| 1862 | cg18608055 | 0.0038 | 0.002824 | 19 | *SBNO2* | Body | TRUE | - |
| 1863 | cg13723336 | 0.0026 | 0.002831 | 8 | *-* | - | NA | - |
| 1864 | cg08027708 | -0.003 | 0.00285 | 1 | *-* | - | NA | - |
| 1865 | cg01763740 | -0.0023 | 0.002856 | 9 | *-* | - | NA | - |
| 1866 | cg06879746 | -0.0043 | 0.002862 | 6 | *VARS2* | Body | TRUE | S_Shore |
| 1867 | cg18709349 | 0.0038 | 0.002862 | 19 | *PNMAL2* | TSS200 | TRUE | Island |
| 1868 | cg02699829 | -0.0043 | 0.002865 | 19 | *TRMT1* | Body | TRUE | S_Shore |
| 1869 | cg09201783 | 0.0024 | 0.002865 | 7 | *SAP25* | TSS1500 | TRUE | Island |
| 1870 | cg10930366 | -0.0052 | 0.002873 | 14 | *MPP5* | Body | TRUE | - |
| 1871 | cg23181172 | -0.005 | 0.002873 | 11 | *LSP1* | 3'UTR | NA | - |
| 1872 | cg20732703 | -0.0039 | 0.002873 | 7 | *GRB10* | 5'UTR | TRUE | - |
| 1873 | cg27666108 | -0.0037 | 0.002873 | 3 | *-* | - | NA | - |
| 1874 | cg12872535 | -0.0029 | 0.002874 | 1 | *-* | - | NA | - |
| 1875 | cg19332649 | -0.0029 | 0.002874 | 9 | *-* | - | NA | - |
| 1876 | cg22250781 | -0.0054 | 0.00288 | 16 | *NHLRC4* | TSS1500 | TRUE | Island |
| 1877 | cg18054943 | -0.0044 | 0.002924 | 17 | *TNK1* | TSS1500 | TRUE | N_Shore |
| 1878 | cg22335882 | -0.0031 | 0.002922 | 8 | *SLC45A4* | Body | TRUE | S_Shore |
| 1879 | cg19929902 | -0.0048 | 0.002949 | 6 | *ENPP1* | Body | NA | - |
| 1880 | cg21519787 | 0.006 | 0.002951 | 19 | *SPHK2* | 3'UTR | TRUE | Island |
| 1881 | cg14629910 | -0.0035 | 0.002952 | 10 | *CELF2* | Body | NA | - |
| 1882 | cg20315326 | -0.0033 | 0.002951 | 17 | *-* | - | NA | - |
| 1883 | cg04960579 | -0.005 | 0.002953 | 3 | *LINC01209* | Body | NA | - |
| 1884 | cg22989283 | -8.00E-04 | 0.002956 | 17 | *MAPT* | Body | NA | - |
| 1885 | cg16117881 | -0.0043 | 0.002959 | 12 | *-* | - | NA | - |
| 1886 | cg01849164 | -0.0029 | 0.002961 | 10 | *-* | - | NA | - |
| 1887 | cg00863306 | -0.0021 | 0.002966 | 19 | *0* | 3'UTR | TRUE | S_Shore |
| 1888 | cg07941506 | 0.0064 | 0.00298 | X | *-* | - | NA | - |
| 1889 | cg18539332 | -0.0031 | 0.00298 | 10 | *C10orf116* | TSS1500 | TRUE | N_Shore |
| 1890 | cg13000597 | -0.0028 | 0.002982 | 11 | *SYT13* | Body | NA | - |
| 1891 | cg09090090 | -0.0047 | 0.002984 | 8 | *-* | - | NA | - |
| 1892 | cg18000076 | -0.0076 | 0.002984 | 11 | *CD151* | 5'UTR | NA | S_Shore |
| 1893 | cg13806740 | -0.0022 | 0.002984 | 17 | *LRRC48* | TSS1500 | NA | N_Shore |
| 1894 | cg21469505 | -0.0037 | 0.002995 | 18 | *-* | - | TRUE | N_Shore |
| 1895 | cg20579173 | 0.0044 | 0.002999 | 12 | *-* | - | NA | - |
| 1896 | cg17451677 | -0.009 | 0.003004 | 10 | *-* | - | TRUE | - |
| 1897 | cg11015305 | 0.0027 | 0.003007 | 4 | *ACSL1* | TSS200 | NA | - |
| 1898 | cg22651852 | -0.0049 | 0.003011 | 3 | *-* | - | NA | - |
| 1899 | cg24221044 | 0.004 | 0.003011 | 4 | *LOC728175* | TSS200 | NA | - |
| 1900 | cg03318573 | 0.0025 | 0.003014 | 14 | *-29* | TSS1500 | TRUE | N_Shore |
| 1901 | cg20115925 | -0.0079 | 0.003022 | 1 | *CASQ2* | Body | NA | - |
| 1902 | cg01578875 | 0.0034 | 0.003022 | 4 | *ZNF827* | Body | TRUE | - |
| 1903 | cg14282721 | -0.0037 | 0.003026 | 20 | *EBF4* | Body | NA | - |
| 1904 | cg14257583 | 0.005 | 0.003026 | 17 | *RBFOX3* | 5'UTR | NA | - |
| 1905 | cg18622950 | -0.0044 | 0.003026 | 2 | *IL1R1* | TSS1500 | NA | N_Shore |
| 1906 | cg25865108 | -0.0035 | 0.003028 | 6 | *-* | - | TRUE | - |
| 1907 | cg18086853 | -0.0045 | 0.003033 | 19 | *-* | - | TRUE | S_Shelf |
| 1908 | cg12324970 | 0.0048 | 0.003034 | 10 | *PITX3* | 5'UTR | TRUE | Island |
| 1909 | cg07022048 | -0.0028 | 0.003038 | 12 | *KRT7* | Body | TRUE | N_Shore |
| 1910 | cg12776916 | -0.0035 | 0.00304 | 7 | *-* | - | NA | - |
| 1911 | cg09563216 | 0.0043 | 0.003044 | 1 | *C1orf51* | 1stExon | TRUE | S_Shore |
| 1912 | cg13224583 | 0.0086 | 0.003045 | 1 | *PEAR1* | Body | TRUE | - |
| 1913 | cg07753717 | 0.0028 | 0.003052 | 22 | *PPARA* | 3'UTR | NA | - |
| 1914 | cg01899253 | -0.0052 | 0.003061 | 13 | *FLT1* | TSS1500 | TRUE | S_Shore |
| 1915 | cg20793144 | 0.0037 | 0.003062 | 2 | *TTC7A* | Body | NA | - |
| 1916 | cg13523510 | -0.0055 | 0.003063 | X | *-* | - | NA | - |
| 1917 | cg09681043 | -0.0025 | 0.003068 | 2 | *MYO7B* | TSS200 | TRUE | - |
| 1918 | cg20331218 | -0.0054 | 0.003073 | 9 | *ASTN2* | Body | NA | - |
| 1919 | cg01644640 | 0.0033 | 0.003079 | 2 | *-* | - | TRUE | - |
| 1920 | cg00042654 | -0.0029 | 0.0031 | 2 | *LRP2* | Body | NA | - |
| 1921 | cg03419051 | -0.0035 | 0.003103 | 17 | *RAB11FIP4* | Body | TRUE | N_Shore |
| 1922 | cg16387850 | 0.0048 | 0.00311 | 1 | *-* | - | TRUE | - |
| 1923 | cg05150077 | 0.0054 | 0.003131 | 12 | *-* | - | TRUE | - |
| 1924 | cg00059225 | 0.0031 | 0.003133 | 5 | *GLRA1* | 1stExon | TRUE | Island |
| 1925 | cg20737242 | -0.0062 | 0.003132 | 4 | *FAT4* | Body | NA | - |
| 1926 | cg08637691 | 0.0024 | 0.003143 | 9 | *-* | - | NA | - |
| 1927 | cg14632941 | -0.0029 | 0.003148 | 4 | *BEND4* | TSS1500 | TRUE | S_Shore |
| 1928 | cg22595188 | 0.0034 | 0.003152 | 11 | *-* | - | NA | - |
| 1929 | cg01059449 | 0.0022 | 0.003168 | 18 | *ST8SIA5* | TSS1500 | TRUE | Island |
| 1930 | cg01994049 | -0.0075 | 0.003181 | 11 | *UEVLD* | TSS200 | NA | - |
| 1931 | cg25005894 | 0.0016 | 0.003182 | 3 | *ERC2* | 5'UTR | TRUE | N_Shore |
| 1932 | cg03352185 | -0.0038 | 0.003183 | 7 | *-* | - | NA | - |
| 1933 | cg16285956 | -0.0031 | 0.003186 | 15 | *-* | - | NA | - |
| 1934 | cg06702607 | 0.0029 | 0.003187 | 3 | *CMTM7* | Body | NA | - |
| 1935 | cg12078492 | -0.0046 | 0.003187 | 3 | *-* | - | NA | S_Shelf |
| 1936 | cg24851859 | -0.004 | 0.003187 | 5 | *-* | - | TRUE | - |
| 1937 | cg00550955 | 0.0041 | 0.003203 | 22 | *-* | - | TRUE | S_Shore |
| 1938 | cg14391148 | 0.0036 | 0.00321 | 9 | *C9orf167* | 3'UTR | TRUE | Island |
| 1939 | cg25453047 | -0.0027 | 0.003211 | 20 | *-* | - | NA | N_Shelf |
| 1940 | cg27529460 | -0.0046 | 0.003226 | 11 | *-* | - | NA | - |
| 1941 | cg03459510 | 0.0028 | 0.00323 | 12 | *RBM19* | Body | NA | - |
| 1942 | cg07508006 | 0.0028 | 0.003236 | 2 | *REV1* | 5'UTR | NA | - |
| 1943 | cg26150489 | -0.0056 | 0.003236 | 11 | *AH-K* | Body | NA | - |
| 1944 | cg27187555 | -0.0062 | 0.003236 | 1 | *PRDM16* | Body | TRUE | - |
| 1945 | cg04155862 | -0.0024 | 0.003236 | 3 | *MGLL* | 5'UTR | TRUE | N_Shore |
| 1946 | cg12650265 | 0.0029 | 0.00326 | 12 | *-* | - | NA | - |
| 1947 | cg00384707 | 0.0035 | 0.003297 | 19 | *SYT3* | Body | TRUE | Island |
| 1948 | cg20360406 | -0.0027 | 0.003298 | 6 | *ZKSCAN8* | TSS1500 | NA | N_Shore |
| 1949 | cg00729699 | -0.0036 | 0.003299 | 8 | *EPB49* | 5'UTR | TRUE | S_Shelf |
| 1950 | cg03748951 | -0.0035 | 0.003299 | 9 | *REXO4* | TSS1500 | NA | S_Shore |
| 1951 | cg19816837 | -0.0029 | 0.003311 | 2 | *LOC644838* | TSS200 | NA | - |
| 1952 | cg13885437 | 0.0039 | 0.003312 | 2 | *PECR* | Body | NA | - |
| 1953 | cg15572436 | -0.0015 | 0.003328 | 11 | *SLC22A8* | Body | TRUE | - |
| 1954 | cg24976744 | -0.0077 | 0.003328 | 5 | *-* | - | NA | - |
| 1955 | cg04651137 | -0.0024 | 0.003347 | 7 | *GPNMB* | Body | TRUE | Island |
| 1956 | cg02355112 | -0.0031 | 0.003349 | 1 | *-* | - | NA | - |
| 1957 | cg06426114 | -0.0083 | 0.003349 | 17 | *PITPNM3* | 3'UTR | TRUE | N_Shelf |
| 1958 | cg23722790 | -0.003 | 0.00336 | 1 | *SLC35D1* | 3'UTR | TRUE | - |
| 1959 | cg26064307 | -0.0026 | 0.003364 | 5 | *TENM2* | Body | NA | - |
| 1960 | cg10449680 | 0.0036 | 0.003366 | 5 | *MCTP1* | Body | NA | - |
| 1961 | cg25893275 | -9.00E-04 | 0.003372 | 1 | *F3* | TSS1500 | TRUE | Island |
| 1962 | cg16138024 | -0.004 | 0.003376 | 12 | *FMNL3* | Body | NA | - |
| 1963 | cg11215901 | -0.0093 | 0.003387 | 4 | *RXFP1* | Body | NA | - |
| 1964 | cg06320917 | -0.0018 | 0.003394 | 12 | *MLF2* | TSS1500 | NA | S_Shore |
| 1965 | cg01692968 | -0.0053 | 0.003397 | 9 | *-* | - | TRUE | N_Shore |
| 1966 | cg07299295 | 0.0017 | 0.003397 | 7 | *-* | - | NA | - |
| 1967 | cg04494335 | 0.0033 | 0.003397 | 15 | *-* | - | NA | - |
| 1968 | cg15723956 | -0.0019 | 0.003397 | 2 | *-* | - | NA | - |
| 1969 | cg20647153 | 0.0026 | 0.003398 | 16 | *-* | - | NA | - |
| 1970 | cg25226285 | 0.0053 | 0.0034 | 13 | *MEDAG* | TSS200 | NA | N_Shore |
| 1971 | cg07102001 | 0.0031 | 0.003404 | 16 | *LOC100129637* | Body | TRUE | N_Shore |
| 1972 | cg18099189 | 0.0021 | 0.003417 | 4 | *-* | - | TRUE | S_Shore |
| 1973 | cg05554536 | -0.0029 | 0.003421 | 15 | *S-P23* | TSS200 | NA | S_Shelf |
| 1974 | cg07433723 | -0.0096 | 0.003425 | 17 | *-* | - | NA | - |
| 1975 | cg21415060 | -0.0038 | 0.003425 | 1 | *FCER1A* | Body | TRUE | - |
| 1976 | cg17355299 | -0.0042 | 0.003432 | 13 | *-* | - | NA | - |
| 1977 | cg24598141 | -0.0043 | 0.003432 | 7 | *-* | - | NA | - |
| 1978 | cg04635543 | -0.0067 | 0.003432 | 3 | *-* | - | NA | - |
| 1979 | cg04986004 | -0.0046 | 0.003432 | 5 | *MIR874* | TSS1500 | TRUE | - |
| 1980 | cg06559756 | 0.0029 | 0.003432 | 17 | *PITPNC1* | Body | TRUE | - |
| 1981 | cg09158878 | -0.0052 | 0.003434 | 22 | *-* | - | NA | N_Shore |
| 1982 | cg15743533 | -0.0026 | 0.003439 | 20 | *FAM110A* | TSS1500 | TRUE | S_Shore |
| 1983 | cg26725604 | -0.0027 | 0.003439 | 18 | *PCAT18* | Body | NA | - |
| 1984 | cg17356498 | -0.0029 | 0.003439 | 8 | *-* | - | NA | - |
| 1985 | cg06689039 | -0.0033 | 0.003476 | 18 | *SERPINB5* | 3'UTR | TRUE | - |
| 1986 | cg07597099 | -0.0028 | 0.00349 | 17 | *PRCD* | Body | NA | - |
| 1987 | cg13071763 | -0.003 | 0.003495 | 3 | *-* | - | NA | - |
| 1988 | cg26649194 | -0.0035 | 0.003497 | 4 | *-* | - | NA | - |
| 1989 | cg24479690 | 0.0038 | 0.003506 | 10 | *PARD3* | Body | NA | - |
| 1990 | cg01469409 | -0.0032 | 0.003507 | 19 | *-* | - | NA | - |
| 1991 | cg27394111 | -0.0046 | 0.003507 | 10 | *-* | - | NA | - |
| 1992 | cg00230207 | -0.002 | 0.003512 | 3 | *-* | - | NA | - |
| 1993 | cg13649581 | 0.0037 | 0.003517 | 7 | *CADPS2* | 1stExon | NA | - |
| 1994 | cg25861229 | -0.0078 | 0.003517 | 14 | *FUT8* | Body | NA | - |
| 1995 | cg17832704 | -0.004 | 0.003517 | 17 | *SHBG* | TSS1500 | TRUE | N_Shore |
| 1996 | cg20830848 | -0.0025 | 0.003517 | 7 | *C7orf49* | Body | TRUE | N_Shore |
| 1997 | cg25431484 | -0.0042 | 0.003517 | 11 | *-* | - | NA | - |
| 1998 | cg05490029 | -0.0059 | 0.003517 | 8 | *IL7* | TSS1500 | TRUE | S_Shore |
| 1999 | cg25045735 | -0.0027 | 0.003517 | 9 | *-* | - | NA | - |
| 2000 | cg16762684 | -0.0034 | 0.003519 | 18 | *MBP* | 5'UTR | TRUE | N_Shelf |
| 2001 | cg04761231 | 0.002 | 0.003524 | 9 | *RPL35* | Body | TRUE | Island |
| 2002 | cg07161822 | -0.0054 | 0.00354 | 7 | *COL26A1* | Body | NA | - |
| 2003 | cg05410362 | -0.0026 | 0.003545 | 7 | *-* | - | NA | - |
| 2004 | cg13128440 | -0.0033 | 0.003545 | 5 | *CPEB4* | Body | NA | - |
| 2005 | cg00592949 | 0.0052 | 0.003554 | 9 | *PALM2* | Body | NA | - |
| 2006 | cg26962618 | -0.0049 | 0.003563 | 15 | *-* | - | TRUE | - |
| 2007 | cg04484995 | -0.0055 | 0.003573 | 18 | *CDH7* | 5'UTR | NA | S_Shore |
| 2008 | cg20779663 | -0.0052 | 0.003593 | 10 | *-* | - | NA | N_Shore |
| 2009 | cg11289601 | -0.0064 | 0.003605 | 5 | *PRR16* | Body | NA | - |
| 2010 | cg20038477 | -0.0026 | 0.003605 | 3 | *CCDC48* | Body | TRUE | - |
| 2011 | cg26543436 | -0.0072 | 0.003608 | 1 | *DNM3* | Body | NA | - |
| 2012 | cg14660461 | -0.0041 | 0.003612 | 4 | *SLC10A7* | Body | NA | - |
| 2013 | cg24622122 | -0.0055 | 0.003618 | 8 | *TMEM67* | Body | NA | - |
| 2014 | cg22679812 | -0.0032 | 0.003622 | 16 | *IL34* | 5'UTR | NA | - |
| 2015 | cg01847889 | -0.0021 | 0.003631 | 22 | *-* | - | TRUE | S_Shelf |
| 2016 | cg07870585 | -0.0034 | 0.003634 | 14 | *ESRRB* | 5'UTR | NA | - |
| 2017 | cg05156137 | -0.009 | 0.003634 | 21 | *RCAN1* | 5'UTR | TRUE | - |
| 2018 | cg05923226 | 0.0046 | 0.003634 | 19 | *CCDC105* | TSS200 | TRUE | N_Shore |
| 2019 | cg11415498 | -0.0056 | 0.003639 | 1 | *-* | - | NA | - |
| 2020 | cg15948836 | -0.0053 | 0.003639 | 11 | *LDLRAD3* | Body | TRUE | - |
| 2021 | cg19671293 | -0.0049 | 0.003639 | 6 | *RMND1* | Body | NA | - |
| 2022 | cg16152924 | -0.0025 | 0.003639 | 11 | *-* | - | NA | - |
| 2023 | cg18609061 | 0.0032 | 0.003639 | 11 | *FXYD2* | TSS1500 | NA | - |
| 2024 | cg27047406 | 0.0036 | 0.003645 | 16 | *SLC6A2* | TSS1500 | TRUE | N_Shore |
| 2025 | cg06924548 | -0.0047 | 0.003654 | 1 | *SLC44A5* | Body | NA | - |
| 2026 | cg16977700 | -0.0039 | 0.003654 | 4 | *-* | - | NA | - |
| 2027 | cg20102280 | -0.007 | 0.003664 | 13 | *HTR2A* | 5'UTR | TRUE | - |
| 2028 | cg11970349 | 0.0019 | 0.003664 | 4 | *GPR78* | TSS200 | TRUE | Island |
| 2029 | cg20828207 | -0.0062 | 0.003664 | 6 | *PLAGL1* | 5'UTR | NA | - |
| 2030 | cg21480966 | -0.0035 | 0.003664 | 3 | *MYH15* | Body | TRUE | - |
| 2031 | cg03487430 | 0.0022 | 0.003666 | 9 | *CD72* | Body | TRUE | N_Shore |
| 2032 | cg27364650 | -0.0039 | 0.003664 | 3 | *-* | - | TRUE | - |
| 2033 | cg00277334 | -0.0044 | 0.003675 | 10 | *-* | - | TRUE | - |
| 2034 | cg00750917 | -0.0034 | 0.003675 | 8 | *FAM135B* | 5'UTR | TRUE | N_Shore |
| 2035 | cg05655457 | -0.0045 | 0.003675 | 11 | *OR5B17* | TSS1500 | TRUE | - |
| 2036 | cg24628298 | -0.0038 | 0.003675 | 17 | *SLC35B1* | TSS1500 | NA | - |
| 2037 | cg03338751 | 0.0031 | 0.003683 | 4 | *-* | - | NA | N_Shore |
| 2038 | cg26811832 | -0.0037 | 0.003683 | 13 | *PCDH9* | 5'UTR | NA | N_Shore |
| 2039 | cg06711175 | -0.0043 | 0.003683 | 5 | *-* | - | TRUE | - |
| 2040 | cg17144735 | -0.0017 | 0.003683 | 10 | *-* | - | NA | - |
| 2041 | cg20868997 | 0.0032 | 0.003683 | 2 | *-* | - | NA | - |
| 2042 | cg24480012 | -0.0051 | 0.003685 | 12 | *FAM19A2* | 5'UTR | TRUE | N_Shore |
| 2043 | cg11165563 | -0.0026 | 0.003699 | 1 | *LGR6* | Body | NA | - |
| 2044 | cg09785387 | 0.0028 | 0.003707 | 20 | *PPP1R16B* | Body | NA | - |
| 2045 | cg11242602 | 0.0041 | 0.003727 | 1 | *HEYL* | Body | NA | - |
| 2046 | cg27038676 | -0.0027 | 0.003732 | 16 | *-* | - | TRUE | - |
| 2047 | cg14176099 | -0.003 | 0.003737 | 1 | *TDRD10* | TSS1500 | NA | N_Shore |
| 2048 | cg06964027 | -0.0023 | 0.00374 | 11 | *MUC2* | TSS1500 | TRUE | - |
| 2049 | cg09983348 | -0.0026 | 0.003755 | 2 | *GRB14* | Body | NA | - |
| 2050 | cg04278580 | 0.0028 | 0.003756 | 7 | *RBM33* | Body | NA | - |
| 2051 | cg11059800 | -0.0028 | 0.003756 | 16 | *EEF2K* | TSS1500 | TRUE | N_Shore |
| 2052 | cg19540617 | -0.0027 | 0.003764 | 19 | *-* | - | NA | - |
| 2053 | cg24436906 | 0.0035 | 0.003764 | 2 | *BOK* | TSS200 | TRUE | Island |
| 2054 | cg25530908 | -0.0015 | 0.003764 | 6 | *-* | - | TRUE | - |
| 2055 | cg05350315 | 0.0024 | 0.003803 | 1 | *LCK* | 5'UTR | TRUE | S_Shelf |
| 2056 | cg08951931 | -0.0037 | 0.003803 | 16 | *PAGR1* | 3'UTR | NA | - |
| 2057 | cg09751812 | -0.0024 | 0.003809 | 3 | *EIF4G1* | 5'UTR | NA | S_Shelf |
| 2058 | cg05009902 | 0.0038 | 0.003812 | 17 | *-* | - | NA | - |
| 2059 | cg22901840 | 0.0034 | 0.003813 | 1 | *DIRAS3* | Body | TRUE | Island |
| 2060 | cg19670290 | -0.0025 | 0.00382 | 15 | *HDDC3* | TSS1500 | TRUE | N_Shore |
| 2061 | cg26837399 | -0.0029 | 0.00382 | 1 | *NID1* | Body | TRUE | - |
| 2062 | cg17695841 | 0.0028 | 0.003824 | 11 | *SLC35C1* | TSS200 | TRUE | N_Shore |
| 2063 | cg26331343 | -0.0044 | 0.003825 | 3 | *-* | - | TRUE | S_Shore |
| 2064 | cg11284797 | 0.0032 | 0.003827 | 11 | *-* | - | TRUE | Island |
| 2065 | cg15809217 | -0.0062 | 0.00383 | 6 | *BAT3* | Body | TRUE | - |
| 2066 | cg02120968 | -0.0033 | 0.003838 | 12 | *BTBD11* | Body | NA | - |
| 2067 | cg26019523 | 0.004 | 0.003841 | 4 | *ACSL1* | 5'UTR | NA | - |
| 2068 | cg08531508 | 0.004 | 0.003841 | 14 | *GTF2A1* | TSS1500 | NA | S_Shore |
| 2069 | cg23587449 | -0.0015 | 0.003843 | 4 | *LRAT* | TSS1500 | TRUE | N_Shore |
| 2070 | cg27560040 | -0.0037 | 0.003846 | 6 | *-* | - | NA | - |
| 2071 | cg10686304 | 0.0029 | 0.003848 | 8 | *-* | - | TRUE | Island |
| 2072 | cg11715966 | -0.0035 | 0.003854 | 19 | *CCDC8* | TSS1500 | TRUE | S_Shore |
| 2073 | cg17011173 | 0.0021 | 0.003854 | 5 | *ZNF608* | Body | NA | - |
| 2074 | cg21620690 | -0.0042 | 0.003854 | 17 | *-* | - | NA | - |
| 2075 | cg12170787 | 0.0041 | 0.003856 | 19 | *SBNO2* | Body | TRUE | - |
| 2076 | cg04084157 | 0.0012 | 0.003858 | 7 | *VGF* | TSS200 | TRUE | Island |
| 2077 | cg02172058 | 0.0037 | 0.003864 | 17 | *C17orf64* | 1stExon | TRUE | S_Shore |
| 2078 | cg12615982 | 0.0041 | 0.003868 | 3 | *TERC* | TSS1500 | TRUE | S_Shore |
| 2079 | cg17176619 | 0.0055 | 0.003868 | 13 | *STK24* | Body | TRUE | - |
| 2080 | cg02281167 | 0.0022 | 0.003873 | 6 | *TRIM15* | Body | TRUE | Island |
| 2081 | cg03316752 | -0.0064 | 0.003876 | 15 | *FSIP1* | 5'UTR | TRUE | - |
| 2082 | cg18023598 | 0.0026 | 0.003876 | 1 | *HTR6* | 1stExon | TRUE | Island |
| 2083 | cg01663988 | -0.0023 | 0.00388 | 1 | *WNT4* | Body | NA | S_Shelf |
| 2084 | cg01695994 | 0.004 | 0.00388 | 17 | *-* | - | TRUE | N_Shelf |
| 2085 | cg03314889 | -0.0036 | 0.00388 | 9 | *PALM2* | Body | NA | - |
| 2086 | cg21579239 | -0.0037 | 0.00388 | 15 | *TTBK2* | 5'UTR | TRUE | N_Shore |
| 2087 | cg17802213 | 0.0047 | 0.003883 | 3 | *DGKG* | Body | TRUE | N_Shore |
| 2088 | cg17936236 | -0.0031 | 0.003889 | 1 | *CREG1* | Body | TRUE | N_Shelf |
| 2089 | cg22169866 | -0.004 | 0.003899 | 1 | *-* | - | TRUE | - |
| 2090 | cg00792799 | -0.0044 | 0.003909 | 4 | *-* | - | NA | - |
| 2091 | cg04998379 | -0.0026 | 0.003904 | 4 | *-* | - | TRUE | - |
| 2092 | cg15845821 | -0.004 | 0.00391 | 19 | *NWD1* | TSS200 | TRUE | - |
| 2093 | cg25102594 | 0.0029 | 0.003909 | 8 | *PTK2* | Body | NA | - |
| 2094 | cg06214925 | 0.0015 | 0.003918 | 4 | *LOC641518* | TSS1500 | TRUE | Island |
| 2095 | cg17224536 | -0.0054 | 0.003934 | 2 | *KIF3C* | Body | NA | - |
| 2096 | cg20195763 | -0.0048 | 0.003944 | 2 | *ACKR3* | TSS1500 | NA | S_Shore |
| 2097 | cg21167201 | -0.0025 | 0.003934 | 3 | *KALRN* | Body | NA | - |
| 2098 | cg22180675 | -0.0027 | 0.003927 | 8 | *MIR4861* | TSS1500 | NA | - |
| 2099 | cg07769588 | -0.0033 | 0.003958 | 19 | *ATG4D* | Body | TRUE | S_Shore |
| 2100 | cg08531746 | 0.0029 | 0.003967 | 13 | *ATP11A* | Body | TRUE | - |
| 2101 | cg11126134 | 0.0036 | 0.003975 | 13 | *C13orf33* | TSS200 | TRUE | Island |
| 2102 | cg05493528 | -0.0022 | 0.003994 | 1 | *AK4* | Body | NA | - |
| 2103 | cg06823795 | -0.0025 | 0.003989 | 9 | *-* | - | NA | - |
| 2104 | cg19753867 | -0.0022 | 0.003978 | 20 | *DSN1* | Body | TRUE | - |
| 2105 | cg23044186 | -0.0033 | 0.003982 | 5 | *-* | - | TRUE | N_Shore |
| 2106 | cg24334507 | -0.0033 | 0.003982 | 20 | *WISP2* | TSS1500 | NA | - |
| 2107 | cg27285103 | -0.0025 | 0.003989 | 12 | *-* | - | NA | - |
| 2108 | cg10860819 | -0.004 | 0.004022 | 12 | *LRRK2* | Body | TRUE | S_Shore |
| 2109 | cg14137282 | 0.0027 | 0.004011 | 19 | *-* | - | NA | N_Shelf |
| 2110 | cg15297650 | -0.0036 | 0.004034 | 2 | *TMEM163* | TSS1500 | TRUE | S_Shore |
| 2111 | cg23597186 | -0.0047 | 0.004014 | 7 | *-* | - | NA | - |
| 2112 | cg01107130 | -0.003 | 0.004063 | 17 | *-* | - | NA | - |
| 2113 | cg04043623 | -0.0028 | 0.004051 | 20 | *AHCY* | 5'UTR | TRUE | S_Shore |
| 2114 | cg08018499 | -0.0025 | 0.004044 | 17 | *SAMD14* | Body | NA | N_Shore |
| 2115 | cg26708596 | 0.0023 | 0.004066 | 8 | *LOC101929268* | Body | NA | - |
| 2116 | cg11047325 | 0.0081 | 0.004071 | 17 | *SOCS3* | Body | NA | Island |
| 2117 | cg23983607 | -0.0026 | 0.004071 | 3 | *-* | - | NA | S_Shore |
| 2118 | cg24807394 | -0.003 | 0.004075 | 1 | *-* | - | NA | - |
| 2119 | cg24845774 | 0.0095 | 0.004082 | 8 | *-* | - | NA | - |
| 2120 | cg03732728 | -0.004 | 0.004118 | 10 | *PSD* | Body | NA | S_Shore |
| 2121 | cg04238302 | -0.0016 | 0.004113 | 1 | *-* | - | NA | - |
| 2122 | cg05593628 | 0.0024 | 0.004119 | 8 | *KCNQ3* | Body | NA | - |
| 2123 | cg16665765 | -0.0025 | 0.00412 | 3 | *CTDSPL* | Body | TRUE | - |
| 2124 | cg00203698 | 0.0021 | 0.004138 | 12 | *GALNT6* | 5'UTR | NA | - |
| 2125 | cg18552219 | -0.0041 | 0.004135 | 18 | *LOC284294* | Body | NA | - |
| 2126 | cg11153400 | -0.0075 | 0.004168 | 22 | *C22orf26* | Body | TRUE | N_Shore |
| 2127 | cg11425788 | -0.0046 | 0.004168 | 1 | *FGGY* | Body | TRUE | - |
| 2128 | cg25194937 | -0.0032 | 0.00417 | 4 | *PPBP* | TSS1500 | NA | - |
| 2129 | cg27555041 | -0.0042 | 0.004169 | 3 | *RARB* | 5'UTR | NA | - |
| 2130 | cg02256616 | 0.0041 | 0.004213 | 16 | *IFT140* | Body | NA | - |
| 2131 | cg08043020 | 0.0039 | 0.004199 | 11 | *-* | - | TRUE | N_Shelf |
| 2132 | cg11319232 | -0.0036 | 0.004201 | 12 | *PPM1H* | Body | NA | - |
| 2133 | cg12521566 | -0.0021 | 0.004215 | 1 | *-* | - | NA | - |
| 2134 | cg20997268 | -0.0034 | 0.004199 | 7 | *CREB5* | TSS1500 | TRUE | - |
| 2135 | cg23368715 | -0.003 | 0.004197 | 12 | *C1R* | TSS1500 | TRUE | - |
| 2136 | cg05383255 | -0.0057 | 0.004233 | 6 | *FYN* | TSS1500 | NA | - |
| 2137 | cg14127927 | -0.0036 | 0.004233 | 14 | *-* | - | TRUE | - |
| 2138 | cg18788940 | -0.0042 | 0.004237 | 11 | *HTATIP2* | TSS1500 | TRUE | N_Shore |
| 2139 | cg21309222 | -0.003 | 0.004246 | 3 | *FRMD4B* | Body | NA | - |
| 2140 | cg01778935 | -0.0048 | 0.004266 | 7 | *-* | - | NA | - |
| 2141 | cg03586713 | -0.0029 | 0.004261 | 11 | *CCDC86* | TSS1500 | NA | N_Shore |
| 2142 | cg13305373 | -0.0034 | 0.004261 | 17 | *RGS9* | Body | NA | - |
| 2143 | cg15490801 | 0.0055 | 0.004261 | 14 | *PRKD1* | Body | TRUE | - |
| 2144 | cg18758922 | 0.0023 | 0.004264 | 22 | *CACNG2* | TSS1500 | NA | S_Shore |
| 2145 | cg19381811 | -0.0035 | 0.004261 | 3 | *UBA7* | TSS1500 | TRUE | - |
| 2146 | cg21985590 | 0.002 | 0.004256 | 6 | *RIPPLY2* | Body | TRUE | S_Shore |
| 2147 | cg22477590 | 0.0031 | 0.004271 | 1 | *EFCAB14AS1* | Body | NA | - |
| 2148 | cg04431742 | -0.0041 | 0.004294 | 4 | *RGS12* | Body | NA | - |
| 2149 | cg09789543 | -0.0024 | 0.004284 | 3 | *ITGB5* | 1stExon | NA | - |
| 2150 | cg16498975 | 0.0022 | 0.004274 | 5 | *-* | - | NA | - |
| 2151 | cg16907824 | -0.0019 | 0.004289 | 16 | *TMEM114* | TSS200 | NA | S_Shelf |
| 2152 | cg18419716 | -0.0055 | 0.004274 | 5 | *CRHBP* | Body | NA | S_Shelf |
| 2153 | cg21397399 | 0.0026 | 0.004291 | 21 | *LINC01426* | Body | NA | - |
| 2154 | cg22365313 | -0.0027 | 0.004284 | 4 | *PAPSS1* | Body | TRUE | - |
| 2155 | cg05207048 | -0.0061 | 0.004302 | 5 | *ODZ2* | Body | TRUE | - |
| 2156 | cg05697274 | 0.0085 | 0.004298 | 1 | *GALNT2* | 3'UTR | TRUE | Island |
| 2157 | cg14674720 | 0.0015 | 0.004313 | 2 | *-* | - | TRUE | Island |
| 2158 | cg20972877 | -0.0028 | 0.004304 | 14 | *PLEKHH1* | TSS1500 | NA | N_Shore |
| 2159 | cg22581137 | 0.0023 | 0.004308 | 17 | *KAT2A* | Body | NA | N_Shore |
| 2160 | cg25167618 | -0.0038 | 0.004308 | 3 | *SLC12A8* | Body | NA | - |
| 2161 | cg02224002 | -0.0032 | 0.00434 | 16 | *RNF166* | Body | TRUE | N_Shelf |
| 2162 | cg02482690 | -0.004 | 0.004332 | 22 | *KCTD17* | Body | TRUE | S_Shore |
| 2163 | cg14614643 | -0.0067 | 0.004344 | 1 | *-* | - | TRUE | - |
| 2164 | cg21327055 | -0.0053 | 0.00434 | 2 | *-* | - | NA | - |
| 2165 | cg21817059 | -0.0032 | 0.004325 | 1 | *-* | - | NA | - |
| 2166 | cg02646470 | -0.0024 | 0.00435 | 1 | *GBAP1* | Body | NA | - |
| 2167 | cg04730882 | 0.0029 | 0.00437 | 2 | *HOXD9* | TSS1500 | TRUE | Island |
| 2168 | cg10750730 | -0.005 | 0.004375 | 14 | *FOXN3* | 5'UTR | NA | - |
| 2169 | cg11332163 | 0.002 | 0.004374 | 4 | *-* | - | TRUE | Island |
| 2170 | cg00897409 | -0.0027 | 0.004386 | 10 | *GLUD1* | Body | NA | - |
| 2171 | cg02586450 | 0.006 | 0.004395 | 3 | *LINC01471* | Body | NA | - |
| 2172 | cg03643998 | -0.0025 | 0.004386 | 17 | *C1QTNF1* | 5'UTR | TRUE | N_Shore |
| 2173 | cg08189998 | -0.0026 | 0.004391 | 6 | *KIF6* | Body | NA | - |
| 2174 | cg08815515 | -0.008 | 0.004391 | 2 | *CFAP221* | Body | NA | - |
| 2175 | cg14010720 | -0.0041 | 0.004386 | 20 | *PTK6* | TSS200 | TRUE | S_Shore |
| 2176 | cg14190522 | -0.0056 | 0.004391 | 9 | *DAB2IP* | Body | TRUE | - |
| 2177 | cg14825251 | 0.0027 | 0.004395 | 11 | *RAG1* | TSS1500 | NA | - |
| 2178 | cg22178367 | 0.003 | 0.004384 | 15 | *CEMIP* | 5'UTR | NA | - |
| 2179 | cg25788793 | -0.0029 | 0.004386 | 4 | *SLC2A9* | 5'UTR | TRUE | - |
| 2180 | cg01559644 | -0.0045 | 0.004404 | 1 | *RASAL2* | Body | NA | - |
| 2181 | cg05389014 | -0.0024 | 0.004407 | 6 | *-* | - | NA | - |
| 2182 | cg06976025 | 0.0016 | 0.0044 | 17 | *CBX4* | Body | TRUE | N_Shore |
| 2183 | cg11782279 | 0.0034 | 0.004406 | 10 | *-* | - | NA | - |
| 2184 | cg16638920 | 0.0019 | 0.004417 | 5 | *LOC645323* | Body | TRUE | Island |
| 2185 | cg17959307 | -0.0033 | 0.004408 | 22 | *PRR34AS1* | Body | NA | S_Shore |
| 2186 | cg22516725 | 0.0024 | 0.004406 | 16 | *WWOX* | Body | NA | - |
| 2187 | cg24907511 | -0.0022 | 0.004408 | 17 | *-* | - | NA | - |
| 2188 | cg00094998 | 0.0014 | 0.004418 | 22 | *SGSM3* | 5'UTR | NA | - |
| 2189 | cg00985388 | -0.0038 | 0.004433 | 3 | *VWA5B2* | Body | TRUE | S_Shelf |
| 2190 | cg04427498 | 0.0033 | 0.004418 | 7 | *-* | - | TRUE | Island |
| 2191 | cg07204969 | -0.0029 | 0.00443 | 1 | *ZBTB48* | Body | NA | - |
| 2192 | cg09110771 | 0.0022 | 0.004429 | 20 | *-* | - | NA | - |
| 2193 | cg14200127 | -0.0066 | 0.004435 | 1 | *DPYD* | Body | TRUE | - |
| 2194 | cg22496437 | -0.0046 | 0.004417 | 1 | *ARHGEF10L* | 5'UTR | TRUE | - |
| 2195 | cg02772754 | 0.0064 | 0.004445 | 22 | *MED15* | Body | NA | - |
| 2196 | cg08357286 | -0.0022 | 0.004454 | 12 | *C12orf10* | TSS1500 | NA | N_Shore |
| 2197 | cg11027822 | 0.0021 | 0.004445 | 2 | *ITGA6* | Body | TRUE | - |
| 2198 | cg11457007 | -0.0027 | 0.004445 | 1 | *GNG12* | 5'UTR | NA | - |
| 2199 | cg14890331 | 0.0025 | 0.004445 | 17 | *PHF12* | Body | NA | N_Shelf |
| 2200 | cg17327848 | -0.002 | 0.004445 | 12 | *-* | - | NA | - |
| 2201 | cg20075319 | -0.0035 | 0.004445 | 1 | *KCND3* | Body | NA | - |
| 2202 | cg21860689 | -0.0049 | 0.004445 | 17 | *TNK1* | TSS1500 | NA | N_Shore |
| 2203 | cg21946545 | -0.0027 | 0.004445 | 2 | *-* | - | NA | - |
| 2204 | cg11206131 | 0.002 | 0.00447 | 19 | *KDM4B* | 5'UTR | NA | - |
| 2205 | cg18884834 | -0.0022 | 0.004463 | 20 | *-* | - | NA | - |
| 2206 | cg25365109 | -0.0027 | 0.004477 | 16 | *-* | - | NA | - |
| 2207 | cg04662594 | -0.0043 | 0.004497 | 8 | *EPB49* | 5'UTR | TRUE | S_Shelf |
| 2208 | cg05519821 | 0.0026 | 0.00449 | 19 | *CERS4* | 5'UTR | NA | - |
| 2209 | cg11410682 | 0.0051 | 0.004491 | 19 | *PNMAL2* | TSS200 | TRUE | Island |
| 2210 | cg12404281 | 0.0041 | 0.004491 | 1 | *RXRG* | TSS1500 | TRUE | - |
| 2211 | cg18290871 | 0.0052 | 0.004497 | 13 | *GPC5* | Body | NA | - |
| 2212 | cg22925712 | 0.0064 | 0.004491 | 1 | *FMO1* | 5'UTR | NA | - |
| 2213 | cg24557546 | -0.0042 | 0.004491 | 9 | *G-14* | Body | NA | - |
| 2214 | cg00033202 | -0.0061 | 0.004539 | 4 | *SPATA5* | Body | NA | - |
| 2215 | cg13447308 | 0.0023 | 0.004538 | 15 | *-* | - | NA | - |
| 2216 | cg13943460 | -0.0049 | 0.004538 | 6 | *QKI* | Body | NA | - |
| 2217 | cg26577241 | 0.0028 | 0.004538 | 1 | *PCNXL2* | Body | NA | - |
| 2218 | cg07822010 | 0.004 | 0.004539 | 11 | *SLC22A20* | TSS1500 | NA | - |
| 2219 | cg18635670 | -0.0034 | 0.004539 | 6 | *HMGN4* | 5'UTR | TRUE | S_Shore |
| 2220 | cg19355120 | 0.0017 | 0.004552 | 7 | *EVX1AS* | Body | NA | Island |
| 2221 | cg19441947 | -0.0028 | 0.004554 | 16 | *-* | - | NA | - |
| 2222 | cg23595222 | -0.0079 | 0.004539 | 22 | *C22orf26* | Body | TRUE | N_Shore |
| 2223 | cg01091029 | 0.0031 | 0.004582 | 7 | *-* | - | TRUE | N_Shore |
| 2224 | cg01538166 | -0.0052 | 0.004581 | 17 | *-* | - | TRUE | Island |
| 2225 | cg04329478 | -0.0039 | 0.004581 | 19 | *LOC400696* | TSS200 | TRUE | - |
| 2226 | cg08781584 | -0.0027 | 0.004579 | 21 | *NCR-00113* | Body | TRUE | - |
| 2227 | cg09470832 | -0.0041 | 0.004579 | 22 | *CACNG2* | Body | NA | - |
| 2228 | cg14874993 | -0.0028 | 0.004579 | 6 | *RPS6KA2* | Body | NA | - |
| 2229 | cg18929842 | -0.0023 | 0.004568 | 17 | *-* | - | TRUE | N_Shore |
| 2230 | cg19222775 | -0.0034 | 0.004579 | 12 | *-* | - | NA | - |
| 2231 | cg03977395 | -0.0041 | 0.004615 | 12 | *CAC-2D4* | Body | NA | - |
| 2232 | cg01895164 | -0.0018 | 0.004642 | 1 | *-* | - | NA | - |
| 2233 | cg19307112 | 0.0025 | 0.004648 | 16 | *-* | - | NA | - |
| 2234 | cg24642844 | 0.0042 | 0.004648 | 7 | *C7orf50* | Body | TRUE | S_Shore |
| 2235 | cg04842552 | -0.004 | 0.004683 | 4 | *STOX2* | Body | NA | - |
| 2236 | cg07797660 | -0.0027 | 0.004683 | 1 | *PRDM16* | Body | TRUE | N_Shore |
| 2237 | cg02379533 | -0.002 | 0.004692 | 5 | *C5orf4* | Body | TRUE | - |
| 2238 | cg03922748 | -0.0026 | 0.004697 | 2 | *D-JB2* | TSS1500 | TRUE | N_Shore |
| 2239 | cg15296238 | -0.0026 | 0.004686 | 7 | *LAMB4* | Body | NA | - |
| 2240 | cg18645861 | -0.0044 | 0.004687 | 1 | *-* | - | NA | - |
| 2241 | cg23669081 | 0.017 | 0.004697 | 17 | *HOXB7* | Body | TRUE | Island |
| 2242 | cg26618737 | -0.0043 | 0.004686 | 8 | *C8orf86* | Body | NA | - |
| 2243 | cg00817490 | -0.0036 | 0.004709 | 16 | *-* | - | NA | - |
| 2244 | cg01076874 | 0.0059 | 0.004721 | 19 | *LRFN1* | 3'UTR | TRUE | Island |
| 2245 | cg05507065 | -0.0047 | 0.004721 | X | *ARHGAP6* | Body | NA | - |
| 2246 | cg07262842 | -0.0043 | 0.004723 | 1 | *LOC101927876* | TSS1500 | NA | - |
| 2247 | cg26306437 | -0.0026 | 0.00471 | 20 | *CST7* | 1stExon | TRUE | - |
| 2248 | cg00554833 | -0.0037 | 0.004754 | 10 | *C10orf71* | 5'UTR | NA | - |
| 2249 | cg07425963 | -0.0047 | 0.004758 | 2 | *MLPH* | Body | NA | - |
| 2250 | cg13752418 | 0.0035 | 0.004765 | 7 | *SRRM3* | Body | NA | - |
| 2251 | cg05198378 | -0.0066 | 0.004788 | 5 | *-* | - | NA | - |
| 2252 | cg06487288 | -0.0032 | 0.004788 | 3 | *-* | - | NA | - |
| 2253 | cg06779591 | -0.003 | 0.004788 | 3 | *-* | - | NA | - |
| 2254 | cg07403981 | -0.0048 | 0.004775 | 1 | *-* | - | NA | - |
| 2255 | cg16038636 | -0.0018 | 0.004788 | 17 | *FOXJ1* | TSS1500 | TRUE | S_Shore |
| 2256 | cg16693741 | 0.003 | 0.004782 | 4 | *-* | - | NA | - |
| 2257 | cg19777067 | -0.0036 | 0.004775 | 4 | *-* | - | TRUE | Island |
| 2258 | cg04874129 | 0.0023 | 0.004789 | 16 | *SLC6A2* | 1stExon | TRUE | Island |
| 2259 | cg05137895 | -0.0094 | 0.004789 | 8 | *-* | - | NA | - |
| 2260 | cg07465899 | -0.0027 | 0.004789 | 4 | *-* | - | TRUE | N_Shore |
| 2261 | cg11027221 | 0.0035 | 0.004795 | 11 | *UNC93B1* | Body | TRUE | Island |
| 2262 | cg13033938 | -0.0025 | 0.004795 | 3 | *IP6K1* | TSS1500 | TRUE | S_Shore |
| 2263 | cg13812705 | -0.0025 | 0.004789 | 16 | *-* | - | NA | - |
| 2264 | cg14989226 | -0.0046 | 0.004801 | 7 | *SRRM3* | Body | TRUE | S_Shore |
| 2265 | cg16463941 | -0.0032 | 0.004789 | 1 | *LOC101928404* | TSS1500 | NA | - |
| 2266 | cg26480713 | -0.0054 | 0.004789 | 6 | *-* | - | NA | - |
| 2267 | cg09122442 | -0.0027 | 0.004817 | 4 | *-* | - | TRUE | - |
| 2268 | cg16786284 | 0.0075 | 0.00482 | 1 | *-* | - | NA | - |
| 2269 | cg24119717 | -0.0033 | 0.004811 | 5 | *-* | - | TRUE | S_Shore |
| 2270 | cg27007872 | -0.0025 | 0.004822 | 13 | *THSD1* | Body | NA | - |
| 2271 | cg00655875 | -0.0038 | 0.00485 | 7 | *AQP1* | Body | NA | - |
| 2272 | cg05062618 | -0.0053 | 0.00485 | 12 | *MYBPC1* | 1stExon | NA | - |
| 2273 | cg05596756 | -0.0016 | 0.004849 | 12 | *FAM113B* | 5'UTR | TRUE | - |
| 2274 | cg18113734 | -0.0032 | 0.004857 | 7 | *-* | - | TRUE | S_Shelf |
| 2275 | cg27098268 | 0.0028 | 0.004857 | 14 | *IPO4* | Body | NA | N_Shore |
| 2276 | cg01832712 | -0.0019 | 0.004864 | 3 | *-* | - | TRUE | S_Shore |
| 2277 | cg03306998 | -0.0045 | 0.004882 | 11 | *-* | - | NA | - |
| 2278 | cg14938587 | -0.0048 | 0.004877 | 7 | *-* | - | TRUE | - |
| 2279 | cg15548613 | 0.0077 | 0.004872 | 22 | *MAFF* | Body | TRUE | Island |
| 2280 | cg07533559 | 0.0025 | 0.004894 | 13 | *ARHGEF7* | 5'UTR | NA | - |
| 2281 | cg19546057 | -0.003 | 0.004893 | 11 | *C11orf45* | TSS1500 | TRUE | - |
| 2282 | cg00740914 | -0.0027 | 0.004926 | 2 | *-* | - | TRUE | N_Shore |
| 2283 | cg03655701 | -0.0035 | 0.004923 | 1 | *C1orf130* | 5'UTR | TRUE | - |
| 2284 | cg15773296 | -0.0076 | 0.004931 | 14 | *SYNE2* | Body | NA | - |
| 2285 | cg03905718 | -0.0035 | 0.004949 | 16 | *SRL* | Body | TRUE | - |
| 2286 | cg04074321 | 0.0016 | 0.004949 | 10 | *-* | - | TRUE | Island |
| 2287 | cg18031708 | -0.0022 | 0.004949 | 5 | *-* | - | TRUE | - |
| 2288 | cg04567724 | -0.002 | 0.004988 | 8 | *COL22A1* | Body | NA | - |
| 2289 | cg17547033 | -0.0041 | 0.004994 | 2 | *FMNL2* | Body | NA | - |
| 2290 | cg22020059 | -0.003 | 0.004983 | 3 | *QARS* | Body | NA | - |
| 2291 | cg25485376 | -0.0044 | 0.004975 | 4 | *-* | - | NA | - |
| 2292 | cg08175831 | -0.0023 | 0.005034 | X | *EDA* | Body | NA | - |
| 2293 | cg01735324 | -0.0049 | 0.00506 | 18 | *PHLPP1* | Body | NA | - |
| 2294 | cg10577534 | -0.0037 | 0.005039 | 15 | *-* | - | TRUE | N_Shelf |
| 2295 | cg10979567 | -0.0026 | 0.005037 | 2 | *COL6A3* | Body | TRUE | - |
| 2296 | cg12735586 | -0.0049 | 0.005037 | 6 | *-* | - | NA | - |
| 2297 | cg00764612 | 0.0032 | 0.005075 | 1 | *C1orf51* | TSS200 | TRUE | S_Shore |
| 2298 | cg01364222 | 0.0018 | 0.005074 | 2 | *EPCAM* | 5'UTR | NA | N_Shore |
| 2299 | cg02230426 | -0.002 | 0.005087 | 17 | *-* | - | NA | - |
| 2300 | cg21016438 | -0.0021 | 0.005075 | 1 | *MYBPHL* | Body | NA | - |
| 2301 | cg14900807 | 0.0032 | 0.005111 | 22 | *ARFGAP3* | Body | NA | - |
| 2302 | cg14963553 | -0.0041 | 0.005115 | 2 | *-* | - | NA | - |
| 2303 | cg05794482 | -0.003 | 0.005129 | 17 | *-* | - | NA | - |
| 2304 | cg10807450 | -0.0065 | 0.005131 | 6 | *-* | - | NA | S_Shore |
| 2305 | cg17200283 | -0.0029 | 0.005136 | 8 | *-* | - | NA | - |
| 2306 | cg15811468 | -0.0024 | 0.005174 | 19 | *-* | - | NA | - |
| 2307 | cg18083248 | 0.0029 | 0.005152 | 15 | *ATP10A* | TSS1500 | TRUE | S_Shore |
| 2308 | cg23810564 | 0.0043 | 0.005174 | 7 | *CDK14* | TSS200 | NA | - |
| 2309 | cg01987202 | -0.0024 | 0.005199 | 15 | *C15orf52* | TSS200 | TRUE | - |
| 2310 | cg09180564 | 0.0066 | 0.005199 | 1 | *OLFM3* | Body | TRUE | - |
| 2311 | cg12980774 | -0.0061 | 0.005199 | 3 | *GSK3B* | Body | NA | - |
| 2312 | cg16689225 | -0.0032 | 0.005202 | 12 | *LOC101928162* | Body | NA | - |
| 2313 | cg00433563 | 0.003 | 0.00526 | 2 | *ARHGAP25* | Body | NA | - |
| 2314 | cg02736016 | -0.0035 | 0.005245 | 12 | *WNK1* | Body | TRUE | - |
| 2315 | cg06264633 | -0.0016 | 0.005238 | 11 | *SLC39A13* | 1stExon | NA | S_Shelf |
| 2316 | cg12801027 | -0.0081 | 0.005238 | 15 | *DPH6AS1* | Body | NA | - |
| 2317 | cg17777077 | -0.0032 | 0.005251 | 18 | *DSG2* | Body | NA | - |
| 2318 | cg15999702 | 0.0038 | 0.005281 | 4 | *LOC728175* | TSS200 | NA | - |
| 2319 | cg22478240 | -0.002 | 0.005278 | 10 | *-* | - | NA | - |
| 2320 | cg08178756 | -0.0049 | 0.005302 | 22 | *-* | - | NA | N_Shore |
| 2321 | cg08638989 | -0.0034 | 0.005317 | 11 | *-* | - | NA | - |
| 2322 | cg08936253 | 0.0033 | 0.005313 | 18 | *ST8SIA5* | Body | NA | N_Shore |
| 2323 | cg15538427 | -0.0022 | 0.005342 | 11 | *LRRN4CL* | 5'UTR | TRUE | S_Shore |
| 2324 | cg17307655 | 0.0035 | 0.005341 | 9 | *DFNB31* | 5'UTR | NA | - |
| 2325 | cg17897688 | -0.0047 | 0.00532 | 2 | *-* | - | NA | - |
| 2326 | cg19118660 | -0.0026 | 0.005324 | 9 | *XPA* | TSS1500 | NA | S_Shore |
| 2327 | cg24121503 | -0.0036 | 0.00532 | 6 | *AKAP12* | Body | TRUE | S_Shelf |
| 2328 | cg24643543 | -0.0034 | 0.005334 | 20 | *-* | - | NA | - |
| 2329 | cg02385661 | -0.0023 | 0.005342 | 19 | *TRMT1* | 3'UTR | TRUE | S_Shore |
| 2330 | cg07580762 | -0.0036 | 0.005342 | 1 | *NDUFS2* | 5'UTR | TRUE | Island |
| 2331 | cg08441327 | 0.002 | 0.005353 | 15 | *RASGRF1* | Body | NA | - |
| 2332 | cg10084993 | -0.0029 | 0.005343 | 16 | *SLC9A3R2* | Body | TRUE | S_Shore |
| 2333 | cg16617301 | 0.0038 | 0.005343 | 19 | *PNMAL2* | 1stExon | TRUE | Island |
| 2334 | cg19370065 | -0.0037 | 0.005355 | 15 | *FMN1* | Body | NA | - |
| 2335 | cg25730803 | -0.0054 | 0.005343 | 22 | *-* | - | NA | - |
| 2336 | cg00144480 | -0.0035 | 0.005361 | 2 | *-* | - | NA | - |
| 2337 | cg03018028 | -0.0035 | 0.005367 | 15 | *S-P23* | TSS1500 | NA | S_Shelf |
| 2338 | cg15031678 | -0.0024 | 0.00538 | 16 | *MKL2* | Body | NA | - |
| 2339 | cg17100176 | 0.0033 | 0.005362 | 1 | *FAIM3* | 5'UTR | TRUE | - |
| 2340 | cg19908374 | 0.0038 | 0.005372 | 2 | *-* | - | NA | - |
| 2341 | cg27369013 | 0.0042 | 0.005382 | 2 | *PLCD4* | 1stExon | TRUE | - |
| 2342 | cg01791648 | -0.0047 | 0.005434 | 3 | *SENP7* | TSS1500 | TRUE | S_Shore |
| 2343 | cg04471143 | -0.004 | 0.005426 | 6 | *-* | - | NA | - |
| 2344 | cg04579453 | -0.002 | 0.005426 | 2 | *-* | - | NA | N_Shore |
| 2345 | cg10864298 | -0.0035 | 0.005426 | 2 | *NOSTRIN* | 5'UTR | NA | - |
| 2346 | cg13318543 | 0.0039 | 0.005425 | 12 | *TMTC1* | 5'UTR | TRUE | N_Shelf |
| 2347 | cg17461878 | 0.0035 | 0.005434 | 6 | *UBE2J1* | TSS1500 | NA | S_Shore |
| 2348 | cg26246840 | 0.002 | 0.005434 | 18 | *CD226* | TSS1500 | TRUE | - |
| 2349 | cg23078123 | -0.0035 | 0.005458 | 1 | *GPR177* | Body | TRUE | - |
| 2350 | cg27296793 | -0.002 | 0.005443 | 1 | *-* | - | NA | - |
| 2351 | cg03223072 | -0.003 | 0.005468 | 10 | *ABLIM1* | Body | TRUE | - |
| 2352 | cg05446322 | 0.0048 | 0.005462 | 13 | *MIR548F5* | Body | TRUE | S_Shore |
| 2353 | cg13004877 | 0.0024 | 0.005462 | 20 | *PTPRA* | Body | NA | - |
| 2354 | cg21658656 | -0.0024 | 0.005462 | 2 | *CFAP221* | Body | NA | - |
| 2355 | cg24882673 | -0.0054 | 0.00547 | 8 | *ZMAT4* | Body | TRUE | - |
| 2356 | cg17117814 | 0.0029 | 0.005503 | 17 | *9-Sep* | Body | NA | - |
| 2357 | cg16086570 | 0.0021 | 0.005545 | 5 | *-* | - | TRUE | - |
| 2358 | cg27032492 | -0.0053 | 0.005541 | 14 | *NRXN3* | Body | NA | - |
| 2359 | cg01933515 | -0.0039 | 0.005563 | 14 | *C14orf132* | Body | NA | - |
| 2360 | cg03873281 | -0.0074 | 0.005563 | 5 | *PDLIM4* | 3'UTR | TRUE | S_Shore |
| 2361 | cg18570331 | -0.0039 | 0.005563 | 11 | *MS4A6E* | TSS1500 | TRUE | - |
| 2362 | cg22571654 | -0.0062 | 0.005563 | 3 | *MED12L* | Body | TRUE | S_Shore |
| 2363 | cg24718465 | 0.0042 | 0.005566 | 19 | *PNMAL2* | 1stExon | TRUE | Island |
| 2364 | cg08088161 | 0.0028 | 0.005601 | 19 | *HSPBP1* | TSS200 | TRUE | S_Shore |
| 2365 | cg24597159 | 0.0038 | 0.005578 | 19 | *-* | - | NA | - |
| 2366 | cg07209567 | -0.0028 | 0.005624 | 3 | *-* | - | NA | - |
| 2367 | cg09400475 | -0.0031 | 0.005617 | 1 | *TCTEX1D4* | TSS1500 | NA | S_Shore |
| 2368 | cg20388165 | 0.0066 | 0.005625 | 15 | *CHSY1* | Body | TRUE | - |
| 2369 | cg22332722 | 0.0092 | 0.005617 | 18 | *CDH2* | Body | TRUE | N_Shore |
| 2370 | cg07492033 | -0.0021 | 0.005645 | 18 | *-* | - | NA | - |
| 2371 | cg07529654 | -0.0039 | 0.005629 | 18 | *TGIF1* | 5'UTR | TRUE | N_Shore |
| 2372 | cg13983920 | -0.0069 | 0.00564 | 9 | *-* | - | NA | - |
| 2373 | cg19412295 | 0.003 | 0.005629 | 7 | *-* | - | TRUE | N_Shore |
| 2374 | cg00602880 | -0.0031 | 0.005657 | 1 | *-* | - | NA | - |
| 2375 | cg04249817 | -0.0023 | 0.005674 | 10 | *FUOM* | TSS1500 | NA | S_Shore |
| 2376 | cg04709124 | -0.0022 | 0.005674 | 17 | *STARD3* | 5'UTR | NA | - |
| 2377 | cg09872250 | -0.0031 | 0.005674 | 5 | *MAST4* | Body | NA | - |
| 2378 | cg17102325 | -0.0018 | 0.005666 | 1 | *GJB5* | Body | TRUE | N_Shelf |
| 2379 | cg08001199 | -0.0033 | 0.005694 | 2 | *-* | - | NA | - |
| 2380 | cg22620627 | -0.0029 | 0.005701 | 20 | *CPXM1* | TSS1500 | NA | S_Shore |
| 2381 | cg08761208 | -0.0023 | 0.005706 | 15 | *IGDCC4* | Body | TRUE | S_Shelf |
| 2382 | cg09172850 | -0.004 | 0.005723 | 1 | *-* | - | TRUE | - |
| 2383 | cg01439383 | 0.0033 | 0.005744 | 7 | *AMZ1* | 5'UTR | TRUE | S_Shore |
| 2384 | cg06576867 | -0.0057 | 0.005733 | 22 | *-* | - | NA | N_Shore |
| 2385 | cg13793503 | -0.0037 | 0.005744 | 2 | *SLC4A10* | 1stExon | NA | - |
| 2386 | cg19134288 | 0.0028 | 0.005733 | 4 | *-* | - | NA | - |
| 2387 | cg20633070 | 0.0078 | 0.005733 | 22 | *MED15* | Body | NA | - |
| 2388 | cg25045526 | 0.0015 | 0.005754 | 7 | *FEZF1* | 1stExon | NA | Island |
| 2389 | cg16503933 | -0.0051 | 0.005756 | 6 | *-* | - | TRUE | - |
| 2390 | cg21569104 | 0.0038 | 0.005761 | 22 | *ARFGAP3* | Body | NA | - |
| 2391 | cg26682900 | -0.0018 | 0.005759 | 12 | *HIP1R* | Body | TRUE | - |
| 2392 | cg00987808 | -0.0036 | 0.005794 | 12 | *PDE3A* | Body | NA | S_Shore |
| 2393 | cg03877600 | -0.0024 | 0.005781 | 9 | *-* | - | NA | - |
| 2394 | cg07980026 | -0.0052 | 0.005792 | 1 | *MTHFR* | Body | NA | - |
| 2395 | cg20351187 | -0.0033 | 0.005798 | 6 | *PDE7B* | TSS1500 | NA | - |
| 2396 | cg04211115 | 0.0015 | 0.005825 | 17 | *HS3ST3A1* | 1stExon | TRUE | Island |
| 2397 | cg08469939 | -0.0036 | 0.005804 | 10 | *-* | - | TRUE | S_Shore |
| 2398 | cg21130532 | -0.003 | 0.005831 | 19 | *ISY-1* | TSS1500 | NA | S_Shore |
| 2399 | cg06034194 | -0.006 | 0.00585 | 9 | *LURAP1LAS1* | TSS1500 | NA | - |
| 2400 | cg06657240 | 0.002 | 0.005853 | 18 | *-* | - | TRUE | S_Shelf |
| 2401 | cg07582229 | -0.0044 | 0.005851 | 12 | *-* | - | NA | - |
| 2402 | cg11795824 | -0.004 | 0.005844 | 20 | *-* | - | NA | - |
| 2403 | cg17084492 | -0.0042 | 0.005844 | 3 | *SEMA5B* | Body | NA | - |
| 2404 | cg04163847 | -0.0037 | 0.005861 | 1 | *-* | - | TRUE | - |
| 2405 | cg10580045 | -0.0056 | 0.005862 | 2 | *NYAP2* | Body | NA | - |
| 2406 | cg24007673 | -0.0031 | 0.005861 | 1 | *-* | - | NA | - |
| 2407 | cg14507845 | -0.004 | 0.005882 | 9 | *-* | - | TRUE | - |
| 2408 | cg15457998 | -0.0028 | 0.005892 | 1 | *-* | - | NA | - |
| 2409 | cg03496350 | -0.0024 | 0.005925 | 1 | *ZBTB48* | Body | NA | - |
| 2410 | cg08830157 | 0.0034 | 0.005928 | 5 | *SLC12A7* | Body | TRUE | N_Shore |
| 2411 | cg20772795 | -0.0035 | 0.005923 | 15 | *THSD4* | Body | NA | - |
| 2412 | cg23406643 | -0.0027 | 0.005915 | 19 | *-* | - | NA | - |
| 2413 | cg23664174 | -0.0048 | 0.005927 | 4 | *LNX1* | Body | TRUE | - |
| 2414 | cg27276712 | 0.0042 | 0.005914 | 11 | *FOLH1* | TSS1500 | NA | S_Shore |
| 2415 | cg04991447 | 0.0013 | 0.005933 | 5 | *SEMA6A* | 1stExon | NA | Island |
| 2416 | cg10997284 | 0.0033 | 0.005937 | 11 | *LOC103611081* | Body | NA | - |
| 2417 | cg04024688 | -0.0065 | 0.005958 | 18 | *-* | - | NA | - |
| 2418 | cg24175713 | 0.0034 | 0.005975 | 9 | *C9orf167* | 3'UTR | TRUE | Island |
| 2419 | cg09617088 | -0.0037 | 0.005996 | 4 | *ARHGAP24* | Body | NA | - |
| 2420 | cg20560283 | -0.0043 | 0.006013 | 15 | *-* | - | TRUE | - |
| 2421 | cg00978831 | -0.0041 | 0.006032 | 6 | *-* | - | NA | - |
| 2422 | cg01302656 | 0.0016 | 0.006015 | 14 | *C14orf23* | Body | TRUE | Island |
| 2423 | cg08100069 | -0.0034 | 0.006013 | 9 | *-* | - | TRUE | Island |
| 2424 | cg15357334 | -0.0023 | 0.006039 | 17 | *GAS7* | 1stExon | NA | - |
| 2425 | cg15829826 | -0.0019 | 0.006048 | 11 | *FRMD8* | TSS200 | TRUE | Island |
| 2426 | cg14858407 | 0.0028 | 0.006095 | 4 | *-* | - | NA | - |
| 2427 | cg16504933 | 0.002 | 0.00607 | 11 | *PRDM11* | Body | NA | - |
| 2428 | cg21730334 | -0.0035 | 0.00607 | 12 | *-* | - | NA | - |
| 2429 | cg00266535 | -0.0022 | 0.006124 | 1 | *FCN3* | Body | NA | S_Shelf |
| 2430 | cg05764839 | 0.0049 | 0.006102 | 6 | *PRRT1* | Body | TRUE | Island |
| 2431 | cg09164377 | 0.0032 | 0.006134 | 8 | *TOX* | Body | NA | - |
| 2432 | cg10856032 | -0.0026 | 0.006125 | 1 | *NM-T2* | Body | NA | - |
| 2433 | cg13483447 | 0.0038 | 0.006135 | 1 | *-* | - | TRUE | S_Shore |
| 2434 | cg17233022 | -0.0027 | 0.006128 | 1 | *SESN2* | TSS1500 | NA | N_Shore |
| 2435 | cg20276630 | 0.0041 | 0.006135 | 10 | *-* | - | TRUE | Island |
| 2436 | cg21361322 | 0.0068 | 0.006128 | 22 | *SELM* | 3'UTR | TRUE | Island |
| 2437 | cg26068725 | -0.0019 | 0.006135 | 20 | *-* | - | NA | - |
| 2438 | cg04168145 | 0.0023 | 0.006141 | 20 | *-* | - | NA | - |
| 2439 | cg09606526 | 0.0028 | 0.006152 | 10 | *CELF2* | Body | NA | - |
| 2440 | cg01745411 | -0.0028 | 0.006167 | 5 | *-* | - | NA | - |
| 2441 | cg03131054 | -0.0027 | 0.00617 | 7 | *IQCE* | TSS1500 | NA | N_Shore |
| 2442 | cg03326609 | -0.004 | 0.006177 | 4 | *SPATA5* | Body | NA | - |
| 2443 | cg10404112 | -0.0109 | 0.006167 | 9 | *KANK1* | 5'UTR | NA | - |
| 2444 | cg15197609 | 0.0015 | 0.006167 | 13 | *KLHL1* | 1stExon | NA | Island |
| 2445 | cg15822414 | -0.0035 | 0.006177 | 3 | *-* | - | NA | - |
| 2446 | cg23922083 | -0.0025 | 0.006168 | 19 | *LIPEAS1* | Body | NA | - |
| 2447 | cg02558362 | 0.0021 | 0.006189 | 13 | *MIR548F5* | Body | TRUE | Island |
| 2448 | cg07440147 | -0.0032 | 0.006196 | 7 | *LINC01510* | Body | NA | - |
| 2449 | cg10734892 | -0.0022 | 0.006206 | 8 | *CLU* | 5'UTR | NA | N_Shelf |
| 2450 | cg26789284 | 0.0024 | 0.006189 | 18 | *LINC00907* | Body | NA | - |
| 2451 | cg02921623 | 0.0037 | 0.006216 | 10 | *-* | - | TRUE | N_Shelf |
| 2452 | cg08621843 | 0.0023 | 0.006235 | 2 | *PROC* | Body | TRUE | Island |
| 2453 | cg04940570 | 0.0016 | 0.006245 | 11 | *TEAD1* | 5'UTR | TRUE | Island |
| 2454 | cg08187512 | -0.0026 | 0.006256 | 1 | *ATP8B2* | Body | NA | - |
| 2455 | cg11595624 | -0.0035 | 0.006256 | 9 | *RECK* | Body | NA | - |
| 2456 | cg15985561 | -0.0036 | 0.006242 | 2 | *-* | - | NA | - |
| 2457 | cg26916780 | -0.0022 | 0.006242 | 15 | *ZNF609* | Body | TRUE | - |
| 2458 | cg11649376 | -0.0022 | 0.006257 | 12 | *ACSS3* | Body | TRUE | S_Shore |
| 2459 | cg15894389 | -0.0069 | 0.006257 | 13 | *HTR2A* | 1stExon | TRUE | - |
| 2460 | cg16786644 | 0.0045 | 0.006267 | 1 | *OLFM3* | TSS1500 | NA | - |
| 2461 | cg19322743 | -0.0033 | 0.00626 | 18 | *PSTPIP2* | Body | NA | - |
| 2462 | cg19708554 | -0.0048 | 0.006272 | 1 | *FAM78B* | TSS1500 | TRUE | S_Shore |
| 2463 | cg20549620 | -0.0021 | 0.006257 | 2 | *-* | - | TRUE | - |
| 2464 | cg03084103 | -0.0024 | 0.006283 | 4 | *MIR574* | Body | TRUE | Island |
| 2465 | cg04027040 | -0.0057 | 0.006286 | 6 | *SUPT3H* | 5'UTR | NA | - |
| 2466 | cg12789532 | 0.0034 | 0.006291 | 5 | *MAP3K1* | Body | NA | - |
| 2467 | cg14378798 | -0.0026 | 0.006291 | 9 | *LMX1B* | Body | NA | - |
| 2468 | cg17326555 | 0.0032 | 0.006283 | 1 | *-* | - | TRUE | Island |
| 2469 | cg02687799 | -0.0025 | 0.006297 | 9 | *CTN-L1* | Body | NA | - |
| 2470 | cg04027548 | 0.0077 | 0.006299 | 12 | *KCNJ8* | Body | TRUE | - |
| 2471 | cg13470352 | -0.0038 | 0.006299 | 14 | *-* | - | NA | N_Shore |
| 2472 | cg23170988 | -0.0033 | 0.006299 | 10 | *SNCG* | Body | TRUE | - |
| 2473 | cg27343616 | 0.0014 | 0.006304 | 20 | *PCSK2* | 5'UTR | TRUE | N_Shore |
| 2474 | cg01176433 | -0.0039 | 0.006328 | 15 | *-* | - | TRUE | - |
| 2475 | cg17313245 | -0.0059 | 0.006313 | 18 | *GAREM* | 3'UTR | NA | - |
| 2476 | cg06708720 | -0.0028 | 0.006366 | 12 | *ERC1* | TSS1500 | TRUE | N_Shore |
| 2477 | cg09575238 | -0.0063 | 0.006352 | 9 | *-* | - | NA | - |
| 2478 | cg26300795 | 0.0026 | 0.006358 | 7 | *SUN1* | Body | NA | - |
| 2479 | cg00076653 | -0.0035 | 0.006379 | 4 | *C1QTNF7* | Body | TRUE | - |
| 2480 | cg03778809 | 0.0046 | 0.006384 | 8 | *CLN8* | Body | NA | - |
| 2481 | cg10003526 | -0.0079 | 0.006384 | 5 | *OSMRAS1* | Body | NA | - |
| 2482 | cg11384088 | -0.0039 | 0.006366 | 9 | *-* | - | NA | - |
| 2483 | cg24087280 | -0.004 | 0.006379 | 17 | *SAMD14* | Body | NA | N_Shore |
| 2484 | cg27605701 | -0.0018 | 0.006379 | 16 | *-* | - | TRUE | - |
| 2485 | cg11036041 | -0.0085 | 0.006386 | 4 | *LIMCH1* | Body | TRUE | S_Shore |
| 2486 | cg20838039 | -0.0026 | 0.006385 | 7 | *CUX1* | Body | NA | - |
| 2487 | cg05101643 | -0.0066 | 0.006415 | 5 | *-* | - | NA | - |
| 2488 | cg18704597 | 0.0022 | 0.006417 | 4 | *TRIM2* | 5'UTR | NA | - |
| 2489 | cg26615224 | 0.0015 | 0.006422 | 19 | *TCF3* | Body | TRUE | N_Shore |
| 2490 | cg01318665 | -0.0055 | 0.006441 | 16 | *-* | - | NA | - |
| 2491 | cg09507934 | 0.0027 | 0.006439 | 7 | *ORAI2* | TSS1500 | TRUE | N_Shore |
| 2492 | cg11549945 | 0.0018 | 0.006441 | 1 | *-* | - | NA | Island |
| 2493 | cg12556616 | -0.0037 | 0.006441 | 17 | *WNT3* | Body | NA | - |
| 2494 | cg19511235 | -0.0023 | 0.006446 | 19 | *CRLF1* | Body | TRUE | S_Shelf |
| 2495 | cg21644578 | -0.0014 | 0.006441 | 1 | *ESPNP* | Body | TRUE | S_Shore |
| 2496 | cg27416204 | 0.0032 | 0.006448 | 17 | *PRKCA* | Body | NA | - |
| 2497 | cg08672314 | -0.0042 | 0.006448 | 21 | *-* | - | NA | - |
| 2498 | cg09750469 | -0.0034 | 0.006448 | X | *TCEANC* | TSS1500 | NA | N_Shore |
| 2499 | cg10250072 | -0.0033 | 0.006457 | 5 | *ZNF366* | 5'UTR | NA | - |
| 2500 | cg12294310 | -0.0039 | 0.006467 | 18 | *LOC643542* | Body | NA | - |
| 2501 | cg17435610 | -0.0035 | 0.006448 | 22 | *LOC400927CSNK1E* | 5'UTR | NA | - |
| 2502 | cg20470514 | -0.0051 | 0.006483 | 17 | *VEZF1* | 3'UTR | NA | - |
| 2503 | cg21523751 | -0.0044 | 0.006483 | 1 | *-* | - | TRUE | N_Shelf |
| 2504 | cg25453225 | -0.0041 | 0.006489 | 7 | *MPP6* | TSS1500 | NA | - |
| 2505 | cg01201472 | -0.0035 | 0.006508 | 12 | *CREBL2* | TSS1500 | NA | N_Shore |
| 2506 | cg04232095 | 0.0058 | 0.006499 | 11 | *PPFIBP2* | Body | NA | - |
| 2507 | cg06974546 | -0.0034 | 0.006508 | 14 | *-* | - | NA | - |
| 2508 | cg08564831 | 0.002 | 0.006498 | 21 | *RIPK4* | Body | NA | Island |
| 2509 | cg17183905 | -0.0027 | 0.006509 | 12 | *TRPV4* | 5'UTR | TRUE | - |
| 2510 | cg17303796 | -0.0038 | 0.006509 | 13 | *-* | - | NA | - |
| 2511 | cg18717076 | 0.0028 | 0.006497 | 2 | *-* | - | NA | - |
| 2512 | cg23627828 | -0.0025 | 0.006498 | 1 | *-* | - | TRUE | - |
| 2513 | cg00346883 | 0.0037 | 0.006528 | 16 | *PLA2G15* | Body | TRUE | - |
| 2514 | cg16959033 | 0.0049 | 0.006515 | 6 | *-* | - | NA | - |
| 2515 | cg22933714 | -0.0048 | 0.006521 | 14 | *L3HYPDH* | Body | NA | N_Shore |
| 2516 | cg05509458 | 0.0025 | 0.006545 | 19 | *LAIR1* | TSS200 | NA | - |
| 2517 | cg09704401 | -0.0027 | 0.006572 | 2 | *-* | - | TRUE | S_Shore |
| 2518 | cg19164246 | -0.0068 | 0.006609 | 6 | *RUNX2* | 1stExon | NA | - |
| 2519 | cg24617176 | -0.0038 | 0.006601 | 8 | *SLC10A5* | TSS1500 | NA | - |
| 2520 | cg19449917 | 0.007 | 0.006639 | 3 | *-* | - | TRUE | - |
| 2521 | cg01463225 | 0.0024 | 0.006665 | 9 | *SARDH* | Body | NA | - |
| 2522 | cg17972352 | 0.002 | 0.006672 | 10 | *ALOX5* | Body | TRUE | Island |
| 2523 | cg27346545 | -0.0041 | 0.006665 | 20 | *RAD21L1* | TSS1500 | TRUE | N_Shore |
| 2524 | cg01443755 | 0.0078 | 0.006692 | 12 | *CNTN1* | 5'UTR | TRUE | - |
| 2525 | cg15502698 | -0.004 | 0.006674 | 5 | *SH3TC2* | TSS1500 | NA | - |
| 2526 | cg20657872 | -0.006 | 0.006692 | 16 | *-* | - | NA | - |
| 2527 | cg16661866 | -0.0017 | 0.006723 | 14 | *CLMN* | Body | NA | - |
| 2528 | cg00384847 | -0.0032 | 0.006727 | 1 | *VPS13D* | 5'UTR | TRUE | S_Shore |
| 2529 | cg05673882 | -0.0047 | 0.006748 | 5 | *POLK* | Body | TRUE | - |
| 2530 | cg08349497 | -0.0056 | 0.00673 | 3 | *-* | - | TRUE | - |
| 2531 | cg18190310 | 0.0079 | 0.006727 | 1 | *PLD5* | Body | TRUE | - |
| 2532 | cg19871903 | -0.002 | 0.006746 | 4 | *-* | - | TRUE | - |
| 2533 | cg13900758 | 0.0086 | 0.006758 | 11 | *IGSF9B* | Body | NA | - |
| 2534 | cg07536018 | -0.0033 | 0.006781 | 5 | *SIL1* | 5'UTR | TRUE | - |
| 2535 | cg07779119 | -0.0094 | 0.0068 | 4 | *-* | - | NA | - |
| 2536 | cg14663914 | -0.002 | 0.0068 | 19 | *AZU1* | TSS200 | TRUE | - |
| 2537 | cg20178893 | -0.0029 | 0.0068 | 1 | *PBX1* | Body | NA | - |
| 2538 | cg22006852 | 0.0023 | 0.006828 | 1 | *-* | - | NA | - |
| 2539 | cg16332047 | -0.0045 | 0.006842 | 4 | *-* | - | NA | - |
| 2540 | cg19567836 | -0.0022 | 0.006833 | 19 | *-* | - | NA | - |
| 2541 | cg07704786 | -0.0024 | 0.006867 | 7 | *CDK14* | 3'UTR | NA | - |
| 2542 | cg07738234 | -0.0054 | 0.006857 | 6 | *VGLL2* | 3'UTR | NA | S_Shore |
| 2543 | cg10908299 | -0.0032 | 0.006861 | 5 | *ATP6V0E1* | 3'UTR | NA | - |
| 2544 | cg02219001 | -0.0029 | 0.006884 | 11 | *-* | - | NA | - |
| 2545 | cg09395552 | -0.0036 | 0.006887 | 20 | *CHD6* | Body | NA | - |
| 2546 | cg12871938 | -0.0042 | 0.006884 | 5 | *-* | - | NA | - |
| 2547 | cg13026370 | -0.003 | 0.00689 | 18 | *LOC100192426* | TSS200 | TRUE | N_Shore |
| 2548 | cg14209583 | -0.0054 | 0.006893 | 17 | *ARHGEF15* | 5'UTR | NA | - |
| 2549 | cg07065166 | 0.003 | 0.006899 | 2 | *LOC101929231* | Body | NA | - |
| 2550 | cg19393302 | 0.0022 | 0.006901 | 22 | *ADORA2A* | 5'UTR | NA | - |
| 2551 | cg08423424 | -0.0039 | 0.006939 | 2 | *SH3BP4* | 5'UTR | NA | - |
| 2552 | cg10348486 | -0.0064 | 0.006942 | 11 | *-* | - | NA | - |
| 2553 | cg24842528 | 0.0044 | 0.006942 | 13 | *-* | - | NA | - |
| 2554 | cg07413917 | -0.0027 | 0.006958 | 3 | *-* | - | NA | - |
| 2555 | cg07530264 | 0.002 | 0.006959 | 8 | *-* | - | TRUE | N_Shore |
| 2556 | cg05955675 | -0.0066 | 0.006971 | 16 | *-* | - | TRUE | S_Shore |
| 2557 | cg06645033 | 0.003 | 0.006971 | 1 | *-* | - | TRUE | Island |
| 2558 | cg09808774 | 0.0021 | 0.006977 | 17 | *OTOP3* | Body | TRUE | S_Shore |
| 2559 | cg12534390 | 0.0057 | 0.006971 | 19 | *HOOK2* | Body | TRUE | N_Shore |
| 2560 | cg16530881 | 0.0023 | 0.006971 | 17 | *-* | - | TRUE | - |
| 2561 | cg19137417 | -0.0022 | 0.006971 | 17 | *-* | - | TRUE | S_Shore |
| 2562 | cg19518666 | -0.0062 | 0.006971 | 4 | *PDGFC* | Body | TRUE | - |
| 2563 | cg22399868 | -0.0072 | 0.006977 | 11 | *0* | Body | NA | - |
| 2564 | cg02458141 | -0.002 | 0.00701 | 2 | *HS1BP3* | 3'UTR | TRUE | - |
| 2565 | cg02602957 | 0.005 | 0.007016 | 3 | *RNF123* | Body | NA | - |
| 2566 | cg07230652 | -0.0025 | 0.00701 | 8 | *LINC00536* | Body | NA | - |
| 2567 | cg09458615 | -0.0086 | 0.007023 | 16 | *-* | - | NA | - |
| 2568 | cg14466794 | -0.0034 | 0.00701 | 5 | *-* | - | NA | - |
| 2569 | cg03531326 | -0.0028 | 0.007042 | 19 | *FBXO17* | 5'UTR | TRUE | S_Shelf |
| 2570 | cg15925801 | -0.0046 | 0.007044 | 21 | *HLCS* | Body | NA | - |
| 2571 | cg06067723 | -0.0041 | 0.007067 | 11 | *-* | - | NA | S_Shelf |
| 2572 | cg13716144 | 0.0035 | 0.007061 | 2 | *-* | - | NA | - |
| 2573 | cg14320320 | -0.0042 | 0.007062 | 3 | *-* | - | TRUE | - |
| 2574 | cg02891046 | -0.0054 | 0.007103 | 13 | *LRCH1* | Body | NA | - |
| 2575 | cg11135072 | -0.004 | 0.007105 | 12 | *-* | - | NA | - |
| 2576 | cg12773508 | -0.0069 | 0.007092 | 21 | *-* | - | NA | S_Shore |
| 2577 | cg11836767 | -0.0035 | 0.007112 | 19 | *CARD8* | TSS1500 | NA | - |
| 2578 | cg14114546 | -0.0049 | 0.007112 | 5 | *LHFPL2* | 5'UTR | TRUE | - |
| 2579 | cg15985712 | -0.0047 | 0.007112 | 9 | *RGS3* | Body | NA | - |
| 2580 | cg20717459 | -0.0048 | 0.007112 | 17 | *ARSG* | Body | NA | - |
| 2581 | cg24049468 | -0.0051 | 0.007112 | 1 | *AK3L1* | Body | TRUE | S_Shore |
| 2582 | cg26622895 | -0.0042 | 0.007112 | 12 | *SLC5A8* | TSS1500 | TRUE | S_Shore |
| 2583 | cg20989225 | 0.002 | 0.007146 | 2 | *-* | - | NA | - |
| 2584 | cg25857569 | 0.004 | 0.007126 | 3 | *PPM1L* | Body | TRUE | - |
| 2585 | cg10172884 | 0.0022 | 0.007155 | 9 | *DFNB31* | TSS200 | NA | N_Shore |
| 2586 | cg20544966 | 0.0027 | 0.00715 | 9 | *-* | - | NA | - |
| 2587 | cg00043673 | -0.0027 | 0.007173 | 18 | *-* | - | NA | - |
| 2588 | cg10127367 | -0.0041 | 0.007177 | X | *AFF2* | Body | NA | - |
| 2589 | cg16652349 | -0.0035 | 0.007174 | 2 | *CERKL* | Body | NA | - |
| 2590 | cg17871918 | 0.0019 | 0.007177 | 15 | *SH3GL3* | 5'UTR | NA | - |
| 2591 | cg25773262 | 0.0052 | 0.007175 | 1 | *BCAN* | Body | TRUE | Island |
| 2592 | cg03365491 | -0.0043 | 0.007199 | 8 | *-* | - | NA | - |
| 2593 | cg19878428 | 0.0023 | 0.007193 | 8 | *UBE2V2* | Body | NA | - |
| 2594 | cg23651872 | 0.0075 | 0.007202 | 17 | *TBC1D16* | Body | TRUE | S_Shore |
| 2595 | cg23977964 | 0.0024 | 0.007193 | 5 | *TCF7* | Body | NA | - |
| 2596 | cg11367633 | -0.0027 | 0.007219 | 20 | *CHD6* | TSS1500 | TRUE | S_Shore |
| 2597 | cg03025830 | 0.0066 | 0.00726 | 8 | *FGF17* | Body | TRUE | Island |
| 2598 | cg19748455 | 0.0049 | 0.007257 | 17 | *LOC100996291* | TSS1500 | NA | - |
| 2599 | cg20357873 | -0.0043 | 0.00726 | 5 | *-* | - | NA | - |
| 2600 | cg26547898 | -0.0023 | 0.00726 | 12 | *C12orf36* | 3'UTR | TRUE | - |
| 2601 | cg26596307 | 0.0022 | 0.007267 | 16 | *CX3CL1* | Body | TRUE | - |
| 2602 | cg00089966 | -0.0033 | 0.007293 | 5 | *-* | - | TRUE | N_Shelf |
| 2603 | cg08642787 | 0.0022 | 0.007313 | X | *RPS6KA6* | TSS200 | TRUE | Island |
| 2604 | cg00109764 | -0.0037 | 0.007331 | 3 | *-* | - | TRUE | - |
| 2605 | cg07552130 | 0.0029 | 0.007315 | 18 | *LDLRAD4* | 5'UTR | NA | S_Shore |
| 2606 | cg13723989 | -0.0035 | 0.007331 | 8 | *IKBKB* | TSS1500 | NA | N_Shore |
| 2607 | cg02845345 | -0.005 | 0.007363 | 17 | *ASIC2* | Body | NA | - |
| 2608 | cg13074526 | 0.0064 | 0.007344 | 17 | *LOC100996291* | TSS200 | NA | - |
| 2609 | cg03755698 | 0.0028 | 0.007363 | 2 | *WDFY1* | Body | NA | - |
| 2610 | cg13723853 | -0.002 | 0.007363 | 14 | *-* | - | TRUE | - |
| 2611 | cg16280624 | 0.0024 | 0.007381 | 3 | *-* | - | NA | - |
| 2612 | cg04084354 | -0.0017 | 0.007392 | 1 | *-* | - | TRUE | - |
| 2613 | cg06446412 | -0.0035 | 0.007396 | 22 | *PRR34AS1* | Body | NA | S_Shore |
| 2614 | cg07110801 | 0.004 | 0.007408 | 16 | *SPG7* | 3'UTR | TRUE | N_Shelf |
| 2615 | cg05753553 | 0.0051 | 0.007426 | 17 | *-* | - | NA | - |
| 2616 | cg19723436 | -0.0054 | 0.007426 | 17 | *C17orf62* | Body | NA | S_Shore |
| 2617 | cg11629459 | -0.0041 | 0.007434 | 14 | *-* | - | NA | - |
| 2618 | cg27504007 | -0.002 | 0.007451 | 2 | *-* | - | NA | - |
| 2619 | cg00737840 | 0.0011 | 0.007461 | 11 | *PKNOX2* | 5'UTR | TRUE | Island |
| 2620 | cg02340116 | -0.0065 | 0.007462 | 8 | *-* | - | NA | - |
| 2621 | cg03030858 | -0.0042 | 0.007463 | 12 | *-* | - | NA | - |
| 2622 | cg15676677 | -0.0056 | 0.007461 | 5 | *MCC* | Body | TRUE | - |
| 2623 | cg17552710 | 0.0029 | 0.007462 | 5 | *-* | - | NA | S_Shelf |
| 2624 | cg18974246 | 0.0035 | 0.007461 | 2 | *RANBP2* | Body | NA | - |
| 2625 | cg01278041 | 0.0057 | 0.007479 | 4 | *C4orf38* | Body | TRUE | N_Shore |
| 2626 | cg03935116 | 0.0021 | 0.00748 | 12 | *FAM60A* | 5'UTR | TRUE | N_Shore |
| 2627 | cg01640945 | -0.004 | 0.007502 | 7 | *-* | - | TRUE | - |
| 2628 | cg12935478 | -0.0048 | 0.007512 | 21 | *C21orf84* | TSS200 | TRUE | - |
| 2629 | cg17429075 | -0.0035 | 0.007505 | 10 | *-* | - | TRUE | - |
| 2630 | cg17515347 | 0.007 | 0.007505 | 1 | *AIM2* | TSS1500 | TRUE | - |
| 2631 | cg00830215 | -0.0023 | 0.007526 | 5 | *PCYOX1L* | Body | NA | S_Shelf |
| 2632 | cg08405005 | -0.0032 | 0.007528 | 7 | *LOC441204* | Body | NA | - |
| 2633 | cg26152950 | 0.0025 | 0.007527 | 5 | *-* | - | NA | - |
| 2634 | cg12531236 | 0.0026 | 0.00755 | 15 | *-* | - | NA | - |
| 2635 | cg13690543 | 0.0024 | 0.00754 | 6 | *BAT2* | TSS1500 | TRUE | N_Shore |
| 2636 | cg26199540 | -0.0037 | 0.00754 | 17 | *CD300LB* | Body | NA | - |
| 2637 | cg01447828 | -0.0034 | 0.007569 | 19 | *PRX* | TSS200 | TRUE | - |
| 2638 | cg06117039 | 0.0028 | 0.007565 | 13 | *STK24* | Body | NA | - |
| 2639 | cg07279842 | -0.0024 | 0.007569 | 3 | *-* | - | NA | - |
| 2640 | cg18791929 | -0.0034 | 0.007569 | 3 | *MYH15* | Body | TRUE | - |
| 2641 | cg22361181 | -0.0021 | 0.007569 | 17 | *NKIRAS2* | TSS1500 | TRUE | N_Shore |
| 2642 | cg23309303 | -0.004 | 0.007632 | 3 | *ROBO1* | Body | NA | - |
| 2643 | cg09503838 | 0.0025 | 0.007646 | 15 | *VPS18* | Body | NA | - |
| 2644 | cg12710546 | 0.0027 | 0.00766 | 7 | *FOXK1* | Body | NA | - |
| 2645 | cg20606007 | -0.0043 | 0.007653 | 7 | *-* | - | NA | - |
| 2646 | cg07313155 | -0.0013 | 0.007666 | 17 | *THRA* | TSS1500 | TRUE | N_Shore |
| 2647 | cg18382690 | 0.0017 | 0.007666 | 21 | *ABCG1* | Body | NA | - |
| 2648 | cg22622477 | 0.0053 | 0.007661 | 17 | *HOXB7* | Body | TRUE | Island |
| 2649 | cg25632577 | 0.0025 | 0.007704 | 8 | *-* | - | TRUE | - |
| 2650 | cg04324917 | 0.0035 | 0.007715 | 16 | *LOC100129637* | Body | TRUE | N_Shore |
| 2651 | cg26393275 | -0.0043 | 0.00775 | 2 | *GPD2* | Body | NA | - |
| 2652 | cg26403171 | -0.003 | 0.00775 | 5 | *-* | - | TRUE | - |
| 2653 | cg12597822 | -0.0025 | 0.007789 | 16 | *-* | - | NA | - |
| 2654 | cg14836313 | -0.0024 | 0.007789 | 11 | *MRPL23* | Body | TRUE | N_Shore |
| 2655 | cg04188862 | 0.0034 | 0.0078 | 15 | *-* | - | TRUE | S_Shore |
| 2656 | cg13264840 | 0.0094 | 0.007803 | 7 | *-* | - | TRUE | N_Shore |
| 2657 | cg14519664 | -0.0028 | 0.007803 | 9 | *MLLT3* | Body | TRUE | - |
| 2658 | cg05889085 | 0.0096 | 0.007813 | 8 | *-* | - | NA | - |
| 2659 | cg15716705 | -0.003 | 0.00782 | 18 | *-* | - | NA | - |
| 2660 | cg04429328 | -0.0019 | 0.007858 | 5 | *-* | - | NA | - |
| 2661 | cg00189689 | -0.0067 | 0.007873 | 11 | *SIGIRR* | 5'UTR | NA | N_Shelf |
| 2662 | cg01892620 | 0.0037 | 0.007865 | 7 | *-* | - | TRUE | - |
| 2663 | cg02461690 | 0.0025 | 0.007876 | 7 | *EIF4H* | Body | NA | - |
| 2664 | cg13748469 | 0.0011 | 0.007865 | 21 | *TIAM1* | TSS1500 | NA | S_Shore |
| 2665 | cg14672304 | -0.0037 | 0.007877 | 20 | *STK4* | Body | NA | - |
| 2666 | cg23480021 | -0.0127 | 0.007891 | 3 | *-* | - | TRUE | N_Shore |
| 2667 | cg24403479 | -0.0025 | 0.0079 | 1 | *-* | - | NA | - |
| 2668 | cg16616600 | -0.0077 | 0.007931 | 2 | *-* | - | NA | - |
| 2669 | cg00361201 | -0.0043 | 0.007931 | 1 | *MMACHC* | Body | NA | - |
| 2670 | cg06473177 | -0.0044 | 0.007931 | 7 | *AASS* | 5'UTR | NA | - |
| 2671 | cg06602847 | 0.0012 | 0.007931 | 9 | *DBC1* | 5'UTR | TRUE | Island |
| 2672 | cg14199723 | 0.0034 | 0.007944 | 8 | *PLEC1* | TSS1500 | TRUE | Island |
| 2673 | cg16477091 | 0.0014 | 0.007944 | 17 | *PPM1E* | TSS1500 | TRUE | Island |
| 2674 | cg11741201 | -0.0029 | 0.007954 | 11 | *FJX1* | TSS1500 | TRUE | N_Shore |
| 2675 | cg07843120 | -0.0016 | 0.007976 | 19 | *GPI* | Body | TRUE | S_Shore |
| 2676 | cg08397633 | -0.0038 | 0.00798 | 15 | *S-P23* | TSS1500 | NA | S_Shelf |
| 2677 | cg26337070 | -0.006 | 0.00798 | 2 | *ATOH8* | Body | TRUE | - |
| 2678 | cg15050614 | 0.0029 | 0.008004 | 17 | *TRIM37* | Body | NA | - |
| 2679 | cg25289697 | 0.0015 | 0.008004 | 1 | *LHX8* | TSS200 | NA | N_Shore |
| 2680 | cg14160221 | -0.0024 | 0.008021 | 21 | *LINC00160* | Body | NA | - |
| 2681 | cg09741855 | -0.0024 | 0.008047 | 17 | *TLCD2* | 3'UTR | NA | - |
| 2682 | cg14746276 | -0.0048 | 0.008058 | 5 | *-* | - | TRUE | S_Shore |
| 2683 | cg22587186 | -0.0027 | 0.008059 | 6 | *LOC101927640* | TSS1500 | NA | - |
| 2684 | cg03725309 | -0.0024 | 0.008085 | 1 | *SARS* | Body | TRUE | S_Shore |
| 2685 | cg00015801 | -0.0033 | 0.008111 | 4 | *SH3RF1* | Body | NA | - |
| 2686 | cg15512469 | -0.0027 | 0.00811 | 11 | *CORO1B* | Body | NA | - |
| 2687 | cg16032635 | -0.0045 | 0.008106 | 8 | *C8orf34* | Body | NA | - |
| 2688 | cg16174681 | -0.0031 | 0.008104 | 5 | *FER* | Body | TRUE | - |
| 2689 | cg09487370 | -0.0026 | 0.008116 | 1 | *-* | - | NA | - |
| 2690 | cg14544125 | -0.0033 | 0.008124 | 4 | *-* | - | NA | - |
| 2691 | cg26303577 | -0.0048 | 0.008124 | 20 | *-* | - | NA | - |
| 2692 | cg03356288 | 0.0025 | 0.008141 | 13 | *KLF12* | 5'UTR | NA | - |
| 2693 | cg09268854 | 0.0027 | 0.008152 | 7 | *TPK1* | Body | NA | - |
| 2694 | cg04232704 | 0.0035 | 0.008175 | 11 | *MRGPRX3* | 5'UTR | NA | - |
| 2695 | cg04724477 | -0.0025 | 0.00816 | 8 | *FAM91A1* | TSS1500 | TRUE | N_Shore |
| 2696 | cg14251509 | -0.005 | 0.008175 | 7 | *HECW1* | 5'UTR | NA | - |
| 2697 | cg25102652 | -0.0051 | 0.00816 | 9 | *-* | - | NA | - |
| 2698 | cg20633146 | -0.0031 | 0.008195 | 1 | *TNR* | 5'UTR | NA | - |
| 2699 | cg03051146 | -0.0047 | 0.008208 | 2 | *-* | - | NA | - |
| 2700 | cg08231710 | 0.0041 | 0.008208 | 1 | *MMP23A* | TSS1500 | TRUE | Island |
| 2701 | cg11399254 | -0.0056 | 0.00822 | 1 | *TAL1* | 5'UTR | TRUE | N_Shore |
| 2702 | cg15853125 | 0.0012 | 0.008215 | 21 | *TIAM1* | TSS1500 | TRUE | S_Shore |
| 2703 | cg17084529 | -0.0031 | 0.008251 | 2 | *-* | - | NA | - |
| 2704 | cg05786809 | 0.0012 | 0.008267 | 14 | *CKB* | TSS1500 | TRUE | Island |
| 2705 | cg21340628 | -0.004 | 0.008258 | 3 | *-* | - | NA | - |
| 2706 | cg21716560 | -0.0027 | 0.008251 | 19 | *RAVER1* | 3'UTR | NA | S_Shore |
| 2707 | cg22181129 | -0.0038 | 0.00826 | 4 | *C4orf45* | Body | NA | - |
| 2708 | cg02172773 | -0.0023 | 0.008291 | 6 | *KIF25* | Body | TRUE | N_Shelf |
| 2709 | cg05078438 | -0.0068 | 0.008286 | 16 | *CDH13* | Body | NA | - |
| 2710 | cg12158483 | 0.0017 | 0.008291 | 22 | *BAIAP2L2* | Body | NA | Island |
| 2711 | cg23650171 | -0.0043 | 0.008305 | 1 | *-* | - | NA | - |
| 2712 | cg27072333 | -0.0071 | 0.008291 | 3 | *ABI3BP* | Body | NA | - |
| 2713 | cg02560069 | -0.0051 | 0.008331 | 15 | *-* | - | NA | - |
| 2714 | cg17952114 | -0.0028 | 0.008381 | 19 | *-* | - | TRUE | S_Shore |
| 2715 | cg18234296 | 0.0033 | 0.008378 | 1 | *C1orf133* | TSS1500 | TRUE | S_Shore |
| 2716 | cg19706697 | -0.0046 | 0.008381 | 10 | *KIAA1217* | 5'UTR | NA | - |
| 2717 | cg23891909 | 0.0038 | 0.008388 | 15 | *ATP10A* | Body | NA | N_Shore |
| 2718 | cg00669776 | 0.0026 | 0.008388 | 6 | *HIVEP1* | Body | NA | - |
| 2719 | cg11324953 | -0.0047 | 0.008395 | 4 | *HTT* | Body | TRUE | S_Shore |
| 2720 | cg12371991 | -0.0063 | 0.008388 | 15 | *-* | - | TRUE | - |
| 2721 | cg14557845 | 0.0021 | 0.008395 | 12 | *ADGRD1* | Body | NA | - |
| 2722 | cg00934609 | -0.0031 | 0.008415 | 1 | *-* | - | NA | - |
| 2723 | cg01719405 | -0.0033 | 0.008441 | 14 | *-* | - | TRUE | - |
| 2724 | cg07173688 | 0.0047 | 0.008443 | 2 | *-* | - | TRUE | - |
| 2725 | cg03822645 | -0.0031 | 0.008477 | 7 | *SLC25A13* | Body | NA | - |
| 2726 | cg10411850 | -0.0109 | 0.008476 | 1 | *TDRD10* | TSS1500 | NA | N_Shore |
| 2727 | cg05797674 | -0.0059 | 0.00848 | 9 | *-* | - | NA | - |
| 2728 | cg09213452 | -0.0048 | 0.008492 | 15 | *-* | - | NA | - |
| 2729 | cg09312999 | -0.0049 | 0.00848 | 1 | *EDARADD* | TSS1500 | NA | N_Shore |
| 2730 | cg15247832 | -0.0017 | 0.008492 | 8 | *GRHL2* | Body | NA | - |
| 2731 | cg22303418 | 0.0019 | 0.008485 | 2 | *-* | - | TRUE | N_Shore |
| 2732 | cg07534516 | -0.0018 | 0.008499 | 10 | *PLXDC2* | TSS1500 | NA | N_Shore |
| 2733 | cg21403806 | -0.0028 | 0.008508 | 8 | *EXT1* | Body | NA | - |
| 2734 | cg01008602 | -0.0031 | 0.008528 | 8 | *TRIM35* | 3'UTR | TRUE | N_Shore |
| 2735 | cg01992590 | 0.0027 | 0.008528 | 17 | *COL1A1* | Body | TRUE | Island |
| 2736 | cg10244503 | -0.0036 | 0.008528 | 1 | *-* | - | TRUE | - |
| 2737 | cg06419300 | -0.0043 | 0.008544 | 5 | *C5orf66* | 5'UTR | NA | - |
| 2738 | cg07886756 | -0.0014 | 0.008542 | 3 | *-* | - | NA | - |
| 2739 | cg12635966 | -0.0026 | 0.008544 | 16 | *RPL3L* | TSS200 | NA | - |
| 2740 | cg20704823 | -0.003 | 0.008544 | 10 | *BICC1* | Body | NA | - |
| 2741 | cg26448394 | -0.0035 | 0.008544 | 4 | *-* | - | TRUE | - |
| 2742 | cg27189533 | -0.0022 | 0.008544 | 16 | *-* | - | TRUE | - |
| 2743 | cg07699901 | -0.0024 | 0.008559 | 20 | *-* | - | NA | - |
| 2744 | cg13007187 | -0.0024 | 0.008571 | 11 | *-* | - | NA | - |
| 2745 | cg01480833 | 0.0026 | 0.008575 | 3 | *ZBTB20* | Body | NA | - |
| 2746 | cg17335281 | -0.0033 | 0.00858 | 5 | *-* | - | NA | - |
| 2747 | cg09512696 | -0.0015 | 0.008605 | 9 | *-* | - | NA | - |
| 2748 | cg11121826 | -0.0031 | 0.008605 | 17 | *SEC14L1* | 5'UTR | NA | - |
| 2749 | cg18432528 | -0.0032 | 0.008594 | 6 | *GMDS* | Body | NA | - |
| 2750 | cg25805115 | -0.0042 | 0.008594 | 1 | *-* | - | NA | - |
| 2751 | cg27315996 | 0.0032 | 0.008596 | 8 | *EPB49* | Body | TRUE | S_Shore |
| 2752 | cg24815891 | 0.0041 | 0.008618 | 8 | *-* | - | NA | - |
| 2753 | cg10280038 | -0.0025 | 0.008647 | 16 | *-* | - | NA | - |
| 2754 | cg12890526 | -0.0052 | 0.008652 | 4 | *-* | - | NA | - |
| 2755 | cg13251145 | -0.0056 | 0.008651 | 19 | *MAP3K10* | Body | NA | S_Shelf |
| 2756 | cg04306089 | 0.0043 | 0.008662 | X | *-* | - | NA | - |
| 2757 | cg11540855 | 0.0041 | 0.008664 | 3 | *VGLL4* | 5'UTR | NA | - |
| 2758 | cg11596239 | -0.0039 | 0.008657 | 1 | *-* | - | TRUE | S_Shelf |
| 2759 | cg20143092 | -0.0013 | 0.00867 | 11 | *CD44* | TSS200 | TRUE | N_Shore |
| 2760 | cg23460663 | -0.0027 | 0.008667 | 13 | *-* | - | NA | - |
| 2761 | cg04562589 | 0.0018 | 0.008704 | 4 | *-* | - | TRUE | - |
| 2762 | cg07396047 | 0.005 | 0.008714 | 16 | *LOC100129637* | Body | TRUE | N_Shore |
| 2763 | cg08415973 | -0.0022 | 0.008714 | 6 | *TDRG1* | TSS200 | TRUE | - |
| 2764 | cg01633902 | 0.005 | 0.008733 | 1 | *VANGL1* | Body | NA | - |
| 2765 | cg06001419 | -0.0036 | 0.008727 | 7 | *-* | - | TRUE | N_Shelf |
| 2766 | cg19759064 | -0.0045 | 0.008725 | 7 | *PHKG1* | 1stExon | TRUE | - |
| 2767 | cg21091503 | -0.0036 | 0.008727 | 1 | *NDUFS2* | TSS200 | NA | N_Shore |
| 2768 | cg05129265 | -0.0032 | 0.008785 | 8 | *SPIDR* | Body | NA | - |
| 2769 | cg14182810 | -0.0037 | 0.008771 | 11 | *-* | - | NA | - |
| 2770 | cg17111166 | -0.0071 | 0.008785 | 11 | *-* | - | TRUE | - |
| 2771 | cg17237086 | 0.0024 | 0.008769 | 22 | *MKL1* | Body | TRUE | Island |
| 2772 | cg02956806 | 0.0025 | 0.008793 | 9 | *C9orf167* | 3'UTR | TRUE | Island |
| 2773 | cg27430293 | -0.0064 | 0.008792 | 16 | *-* | - | TRUE | N_Shore |
| 2774 | cg27634195 | 0.0029 | 0.008792 | 16 | *-* | - | TRUE | N_Shore |
| 2775 | cg01663970 | 0.0035 | 0.008817 | 12 | *SCARB1* | Body | TRUE | Island |
| 2776 | cg05231308 | 0.0057 | 0.008853 | 2 | *UCN* | TSS200 | TRUE | Island |
| 2777 | cg06837596 | -0.0024 | 0.008853 | 14 | *MDGA2* | Body | NA | - |
| 2778 | cg10758229 | 0.0022 | 0.008859 | 21 | *-* | - | NA | - |
| 2779 | cg15193638 | -0.0051 | 0.008859 | 2 | *COBLL1* | Body | NA | - |
| 2780 | cg15922801 | -0.0027 | 0.008859 | 2 | *-* | - | NA | - |
| 2781 | cg25352208 | 0.0034 | 0.008853 | 1 | *CLSTN1* | Body | NA | - |
| 2782 | cg27460717 | -0.0035 | 0.008853 | 4 | *-* | - | NA | - |
| 2783 | cg18801806 | -0.0017 | 0.008863 | 11 | *CCDC84* | Body | TRUE | S_Shore |
| 2784 | cg27528351 | 0.003 | 0.008863 | 7 | *DDC* | 5'UTR | TRUE | - |
| 2785 | cg13931228 | -0.0028 | 0.008898 | 7 | *MPP6* | TSS1500 | TRUE | - |
| 2786 | cg15256783 | 0.0034 | 0.008893 | 22 | *PIWIL3* | Body | NA | - |
| 2787 | cg16657173 | -0.0022 | 0.008889 | 1 | *KCNN3* | Body | NA | - |
| 2788 | cg21527370 | -0.0033 | 0.008905 | 6 | *PPIL6* | Body | TRUE | - |
| 2789 | cg03497059 | -0.0022 | 0.00891 | 11 | *OSBPL5* | Body | NA | - |
| 2790 | cg06102300 | -0.0051 | 0.008912 | X | *KLHL13* | 5'UTR | NA | - |
| 2791 | cg18401778 | -0.0053 | 0.008912 | 3 | *TMEM108* | 5'UTR | TRUE | - |
| 2792 | cg08238472 | -0.0039 | 0.008937 | 14 | *LOC102724890* | Body | NA | - |
| 2793 | cg17180284 | -0.0042 | 0.008932 | 13 | *B3GALTL* | Body | TRUE | - |
| 2794 | cg21968765 | -0.0043 | 0.008932 | 3 | *ABI3BP* | TSS200 | TRUE | - |
| 2795 | cg21995960 | -0.0037 | 0.008931 | 8 | *-* | - | NA | - |
| 2796 | cg24690437 | 0.0034 | 0.008964 | 13 | *C13orf33* | TSS200 | TRUE | Island |
| 2797 | cg24773560 | 0.0035 | 0.008964 | 12 | *IL23A* | 5'UTR | TRUE | - |
| 2798 | cg11752275 | -0.0036 | 0.00897 | 2 | *GNLY* | TSS1500 | TRUE | - |
| 2799 | cg22151881 | 0.0024 | 0.008974 | 11 | *LRP5* | Body | TRUE | S_Shore |
| 2800 | cg24382141 | 0.0037 | 0.008964 | 16 | *PSKH1* | Body | NA | S_Shore |
| 2801 | cg19963224 | -0.004 | 0.00901 | 17 | *NLK* | Body | NA | - |
| 2802 | cg02253924 | -0.0033 | 0.00901 | 6 | *-* | - | NA | - |
| 2803 | cg06530089 | -0.0023 | 0.009025 | 17 | *ANKFN1* | Body | NA | - |
| 2804 | cg04654716 | -0.0034 | 0.009034 | 5 | *FAM169A* | TSS1500 | TRUE | S_Shore |
| 2805 | cg22380139 | -0.0025 | 0.00905 | 8 | *-* | - | TRUE | - |
| 2806 | cg27497667 | -0.0021 | 0.00905 | 1 | *-* | - | NA | - |
| 2807 | cg01195628 | -0.0052 | 0.009075 | 1 | *OBSCN* | Body | TRUE | N_Shore |
| 2808 | cg04348265 | 0.0059 | 0.009081 | 4 | *SORBS2* | 5'UTR | TRUE | - |
| 2809 | cg13305823 | -0.0033 | 0.009081 | 6 | *MAS1L* | 1stExon | TRUE | - |
| 2810 | cg17833746 | 0.0044 | 0.009081 | 17 | *STAT3* | Body | TRUE | - |
| 2811 | cg23766561 | 0.0026 | 0.00909 | 3 | *GXYLT2* | Body | NA | - |
| 2812 | cg26259865 | -0.0023 | 0.009081 | 16 | *ZG16B* | Body | TRUE | - |
| 2813 | cg03418218 | -0.0021 | 0.009091 | 3 | *-* | - | NA | - |
| 2814 | cg09330888 | -0.0032 | 0.009091 | 1 | *PHGDH* | Body | NA | - |
| 2815 | cg14030719 | 0.003 | 0.00909 | 20 | *E2F1* | Body | TRUE | S_Shore |
| 2816 | cg14292870 | 0.0058 | 0.009105 | 12 | *LAG3* | Body | TRUE | - |
| 2817 | cg25811395 | -0.003 | 0.009105 | 9 | *ZNF462* | 5'UTR | NA | - |
| 2818 | cg07184401 | -0.002 | 0.009119 | 10 | *-* | - | NA | - |
| 2819 | cg04955835 | 0.002 | 0.009148 | 17 | *GAS7* | 5'UTR | NA | - |
| 2820 | cg14904725 | -0.0046 | 0.009133 | 13 | *F10* | TSS200 | TRUE | - |
| 2821 | cg26277237 | 0.0041 | 0.009135 | 9 | *KANK1* | 5'UTR | NA | - |
| 2822 | cg02632200 | -0.0023 | 0.009151 | 10 | *SYT15* | TSS1500 | TRUE | S_Shore |
| 2823 | cg11553560 | 0.0021 | 0.009151 | 6 | *REV3L* | Body | NA | - |
| 2824 | cg12103197 | 0.0046 | 0.009151 | 14 | *ANKRD9* | 5'UTR | TRUE | Island |
| 2825 | cg12122356 | -0.0026 | 0.009161 | 21 | *IF-R2* | Body | NA | - |
| 2826 | cg13866747 | -0.0067 | 0.009154 | 13 | *-* | - | NA | - |
| 2827 | cg04358214 | -0.006 | 0.009172 | 16 | *C16orf70* | TSS1500 | TRUE | Island |
| 2828 | cg05082527 | 0.0033 | 0.009172 | 11 | *MIR130A* | TSS1500 | TRUE | - |
| 2829 | cg12912663 | 0.0014 | 0.009172 | 11 | *RIN1* | Body | TRUE | Island |
| 2830 | cg13734871 | -0.0014 | 0.009177 | 17 | *ALYREF* | Body | NA | N_Shore |
| 2831 | cg09093142 | -0.0057 | 0.00919 | 5 | *-* | - | NA | - |
| 2832 | cg16450893 | 0.0019 | 0.009177 | 20 | *-* | - | NA | S_Shore |
| 2833 | cg19787650 | 0.0023 | 0.009177 | 8 | *-* | - | TRUE | S_Shelf |
| 2834 | cg20201031 | -0.0028 | 0.009188 | 12 | *-* | - | NA | - |
| 2835 | cg13862013 | -0.0029 | 0.009199 | 11 | *CPT1A* | Body | NA | N_Shore |
| 2836 | cg14769059 | -0.0023 | 0.009196 | 3 | *MECOM* | 5'UTR | NA | - |
| 2837 | cg18236477 | 0.002 | 0.009197 | 13 | *ATP8A2* | Body | TRUE | Island |
| 2838 | cg08456236 | -0.0026 | 0.009233 | 1 | *-* | - | NA | - |
| 2839 | cg18739031 | -0.0033 | 0.009223 | 12 | *MYRFL* | Body | NA | - |
| 2840 | cg02631686 | 0.0094 | 0.00924 | 6 | *PARK2* | Body | NA | - |
| 2841 | cg03365437 | 0.0014 | 0.00924 | 15 | *ALDH1A2* | 5'UTR | TRUE | Island |
| 2842 | cg07757281 | 0.0027 | 0.00924 | 3 | *-* | - | TRUE | - |
| 2843 | cg14758256 | -0.0042 | 0.00924 | 8 | *-* | - | NA | - |
| 2844 | cg08836984 | -0.0017 | 0.009266 | 1 | *SERTAD4* | 5'UTR | NA | S_Shelf |
| 2845 | cg15503235 | -0.0037 | 0.009266 | 1 | *CA14* | TSS1500 | NA | - |
| 2846 | cg16341707 | -0.0025 | 0.009266 | 2 | *-* | - | NA | - |
| 2847 | cg00498401 | -0.0025 | 0.009269 | 17 | *EVPL* | TSS1500 | TRUE | - |
| 2848 | cg05773708 | -0.003 | 0.00931 | 2 | *LOC284950* | TSS1500 | NA | S_Shelf |
| 2849 | cg12882189 | -0.0035 | 0.009309 | 17 | *C17orf62* | Body | TRUE | S_Shore |
| 2850 | cg27083484 | -0.0045 | 0.00931 | 5 | *PRDM6* | Body | TRUE | - |
| 2851 | cg00331334 | -0.0042 | 0.009322 | 4 | *-* | - | NA | - |
| 2852 | cg03880355 | -0.0034 | 0.00932 | 5 | *FAM169A* | TSS1500 | TRUE | S_Shore |
| 2853 | cg08244745 | -0.0026 | 0.009317 | 17 | *NTN1* | TSS1500 | NA | N_Shore |
| 2854 | cg12076203 | -0.0025 | 0.00932 | 2 | *DPYSL5* | Body | NA | - |
| 2855 | cg26897909 | -0.0026 | 0.00932 | 1 | *SRGAP2* | Body | TRUE | - |
| 2856 | cg27442162 | -0.0021 | 0.009336 | 8 | *PEBP4* | Body | NA | - |
| 2857 | cg00793895 | -0.003 | 0.009364 | 8 | *-* | - | NA | - |
| 2858 | cg27144670 | -0.0038 | 0.009368 | 15 | *TMOD3* | Body | TRUE | - |
| 2859 | cg27635485 | 0.005 | 0.009359 | 4 | *VEGFC* | Body | TRUE | - |
| 2860 | cg01506917 | -0.006 | 0.009377 | 6 | *TRERF1* | 5'UTR | TRUE | N_Shelf |
| 2861 | cg09110744 | -0.0027 | 0.009376 | 6 | *-* | - | NA | - |
| 2862 | cg14381448 | 0.0033 | 0.00938 | 17 | *SLC4A1* | Body | TRUE | - |
| 2863 | cg04092800 | -0.0036 | 0.009395 | 5 | *PTGER4* | Body | TRUE | Island |
| 2864 | cg08344099 | 0.005 | 0.009395 | 3 | *-* | - | NA | - |
| 2865 | cg10732921 | -0.0028 | 0.009395 | 4 | *JADE1* | TSS1500 | NA | N_Shore |
| 2866 | cg23068081 | -0.0043 | 0.009395 | 2 | *LIMS1* | 5'UTR | NA | - |
| 2867 | cg09764150 | -0.0109 | 0.009432 | 14 | *ESR2* | Body | NA | - |
| 2868 | cg22385463 | -0.0027 | 0.009432 | 11 | *TBX10* | TSS1500 | TRUE | - |
| 2869 | cg23104165 | -0.0053 | 0.009432 | 3 | *MECOM* | 5'UTR | NA | - |
| 2870 | cg02269477 | 0.0028 | 0.009457 | 9 | *-* | - | NA | - |
| 2871 | cg07872945 | -0.0033 | 0.009453 | 9 | *-* | - | NA | N_Shore |
| 2872 | cg13514175 | 0.0038 | 0.009455 | 17 | *SERPINF1* | Body | NA | - |
| 2873 | cg15662381 | -0.0054 | 0.009463 | 2 | *ABI2* | 5'UTR | NA | - |
| 2874 | cg23146153 | -0.0032 | 0.009459 | 20 | *LINC00489* | TSS1500 | NA | - |
| 2875 | cg23719650 | -0.0022 | 0.009496 | 3 | *-* | - | NA | S_Shore |
| 2876 | cg03708443 | -0.0022 | 0.009523 | 3 | *-* | - | NA | - |
| 2877 | cg13428418 | -0.0027 | 0.009539 | 5 | *-* | - | NA | - |
| 2878 | cg12353098 | 0.0029 | 0.009554 | 9 | *HIATL2* | Body | NA | - |
| 2879 | cg14221766 | -0.0052 | 0.009544 | 9 | *-* | - | TRUE | - |
| 2880 | cg23980659 | 0.0041 | 0.009544 | X | *-* | - | NA | - |
| 2881 | cg27553776 | -0.0038 | 0.009552 | 6 | *VNN1* | Body | NA | - |
| 2882 | cg08241785 | -0.0025 | 0.009575 | 5 | *F2RL2* | TSS200 | TRUE | - |
| 2883 | cg24865086 | -0.0039 | 0.009579 | 17 | *ARHGEF15* | 5'UTR | NA | - |
| 2884 | cg06930067 | 0.0027 | 0.009605 | 14 | *RAD51B* | Body | NA | - |
| 2885 | cg27160886 | 0.0035 | 0.009608 | 21 | *APP* | 5'UTR | NA | - |
| 2886 | cg07207090 | -0.0069 | 0.009622 | 18 | *RAB27B* | TSS1500 | NA | N_Shore |
| 2887 | cg09168222 | -0.003 | 0.009622 | 4 | *HERC6* | TSS200 | TRUE | N_Shore |
| 2888 | cg26908868 | 0.0014 | 0.009625 | 6 | *-* | - | NA | - |
| 2889 | cg01310332 | 0.0022 | 0.00965 | 12 | *-* | - | NA | - |
| 2890 | cg07069368 | -0.0052 | 0.00965 | 6 | *RUNX2* | TSS1500 | NA | - |
| 2891 | cg11078090 | 0.0029 | 0.009654 | 1 | *-* | - | TRUE | N_Shelf |
| 2892 | cg03593823 | -0.0056 | 0.009654 | 1 | *GREM2* | 5'UTR | TRUE | - |
| 2893 | cg03925395 | 0.0028 | 0.009659 | 8 | *TRAPPC9* | Body | NA | - |
| 2894 | cg04812351 | -0.0034 | 0.009657 | 4 | *ADAMTS3* | Body | TRUE | - |
| 2895 | cg08589600 | -0.0073 | 0.009654 | 5 | *MEGF10* | 5'UTR | NA | - |
| 2896 | cg08718518 | 0.0029 | 0.009659 | 10 | *STAMBPL1* | Body | NA | - |
| 2897 | cg25969661 | -0.0035 | 0.009657 | 19 | *ZNF823* | TSS1500 | NA | S_Shore |
| 2898 | cg27376282 | -0.0064 | 0.009654 | 4 | *SPOCK3* | 5'UTR | NA | - |
| 2899 | cg00217953 | 0.004 | 0.009668 | 2 | *SEMA4C* | Body | TRUE | Island |
| 2900 | cg02503376 | -0.0063 | 0.009668 | 17 | *TNK1* | TSS1500 | TRUE | N_Shore |
| 2901 | cg10247466 | -0.0025 | 0.009687 | 12 | *KRT80* | Body | NA | - |
| 2902 | cg20257087 | -0.0022 | 0.009695 | 3 | *SCHIP1* | Body | NA | - |
| 2903 | cg04931216 | 0.0016 | 0.009726 | 1 | *DMRTA2* | Body | TRUE | Island |
| 2904 | cg10903575 | -0.0027 | 0.009711 | 17 | *NXN* | TSS1500 | NA | - |
| 2905 | cg20741479 | 0.0032 | 0.009713 | 11 | *TEAD1* | 5'UTR | NA | - |
| 2906 | cg04644869 | -0.0031 | 0.009748 | 11 | *ROBO4* | 5'UTR | NA | - |
| 2907 | cg05227865 | -0.0024 | 0.00974 | 5 | *-* | - | NA | - |
| 2908 | cg03326252 | 0.0089 | 0.009777 | 11 | *IGSF9B* | Body | TRUE | - |
| 2909 | cg13529849 | -0.0052 | 0.009777 | 4 | *CCDC158* | TSS1500 | NA | - |
| 2910 | cg20865082 | 0.0044 | 0.009777 | 3 | *-* | - | TRUE | - |
| 2911 | cg24853956 | -0.0038 | 0.009777 | 12 | *IFFO1* | Body | TRUE | N_Shore |
| 2912 | cg26640594 | -0.0035 | 0.009777 | 17 | *LINC00974* | TSS1500 | NA | - |
| 2913 | cg00430037 | 0.0026 | 0.009798 | 7 | *ZSCAN25* | Body | NA | - |
| 2914 | cg02867145 | -0.0027 | 0.009789 | 3 | *RFT1* | Body | TRUE | N_Shore |
| 2915 | cg03996090 | 0.0029 | 0.009789 | 1 | *HSD11B1* | TSS200 | NA | - |
| 2916 | cg08548748 | -0.0018 | 0.009792 | 1 | *DUSP23* | TSS1500 | TRUE | N_Shore |
| 2917 | cg11900294 | -0.0025 | 0.009789 | 12 | *-* | - | NA | - |
| 2918 | cg03860054 | 0.0036 | 0.009844 | 16 | *SLC6A2* | Body | TRUE | S_Shore |
| 2919 | cg04184143 | 0.002 | 0.009841 | 5 | *-* | - | TRUE | N_Shore |
| 2920 | cg23471274 | -0.0194 | 0.00983 | 19 | *-* | - | NA | - |
| 2921 | cg10992481 | -0.0054 | 0.009887 | 6 | *SLC22A23* | 5'UTR | NA | - |
| 2922 | cg12794567 | -0.0029 | 0.009888 | 10 | *-* | - | TRUE | Island |
| 2923 | cg24437221 | -0.0027 | 0.009887 | 7 | *-* | - | NA | - |
| 2924 | cg16969368 | 0.0029 | 0.009899 | 17 | *DHX40* | TSS200 | TRUE | Island |
| 2925 | cg05740045 | 0.0017 | 0.009924 | 8 | *-* | - | TRUE | Island |
| 2926 | cg18008766 | 0.0031 | 0.009933 | 2 | *SFRS7* | TSS1500 | TRUE | S_Shore |
| 2927 | cg24655222 | 0.0015 | 0.009928 | 2 | *ZNF513* | Body | TRUE | Island |
| 2928 | cg06695691 | -0.0042 | 0.009941 | 4 | *SPATA5* | Body | TRUE | - |
| 2929 | cg11271492 | -0.0015 | 0.009948 | 5 | *SLC4A9* | Body | NA | S_Shore |
| 2930 | cg15736994 | -0.0038 | 0.009975 | 4 | *ACCN5* | Body | TRUE | - |
| 2931 | cg27191499 | -0.0042 | 0.009958 | 1 | *PAPPA2* | Body | NA | - |
| 2932 | cg06332058 | -0.0063 | 0.009995 | 9 | *-* | - | NA | - |
| 2933 | cg06649567 | -0.003 | 0.009995 | 8 | *RNF19A* | TSS1500 | NA | S_Shore |
| 2934 | cg00566858 | -0.0016 | 0.009998 | 20 | *-* | - | NA | - |
| 2935 | cg00059874 | -0.0032 | 0.010041 | 9 | *PGM5P2* | Body | NA | N_Shore |
| 2936 | cg04745278 | 0.0028 | 0.010021 | 3 | *OSTN* | Body | NA | - |
| 2937 | cg00187686 | -0.0034 | 0.010068 | 11 | *TCN1* | TSS200 | TRUE | - |
| 2938 | cg01111041 | -0.0033 | 0.010117 | 6 | *PPT2* | TSS1500 | TRUE | N_Shore |
| 2939 | cg06090837 | -0.0045 | 0.010117 | 4 | *-* | - | NA | - |
| 2940 | cg08955995 | 0.0035 | 0.010117 | 19 | *GRIK5* | Body | TRUE | Island |
| 2941 | cg06850054 | -0.0025 | 0.010142 | 4 | *-* | - | NA | - |
| 2942 | cg09363892 | -0.0035 | 0.010142 | 1 | *AGRN* | TSS1500 | TRUE | N_Shore |
| 2943 | cg16799223 | 0.0016 | 0.010142 | 11 | *SPI1* | TSS200 | NA | S_Shore |
| 2944 | cg24221515 | 0.0027 | 0.010142 | 10 | *-* | - | NA | - |
| 2945 | cg27057509 | -0.0046 | 0.010142 | 6 | *VARS2* | Body | TRUE | S_Shore |
| 2946 | cg06376426 | 0.0025 | 0.010169 | 1 | *GRHL3* | TSS1500 | TRUE | Island |
| 2947 | cg07201475 | -0.0068 | 0.010152 | 2 | *-* | - | TRUE | - |
| 2948 | cg03578689 | -0.0027 | 0.010183 | 17 | *ITGA2B* | Body | TRUE | Island |
| 2949 | cg11879741 | -0.0051 | 0.010176 | 13 | *-* | - | TRUE | - |
| 2950 | cg19889669 | 0.0025 | 0.010169 | 12 | *FMNL3* | Body | NA | - |
| 2951 | cg12168054 | 0.0033 | 0.010206 | 8 | *-* | - | TRUE | N_Shore |
| 2952 | cg25268718 | -0.003 | 0.010219 | 14 | *PSME1* | TSS1500 | TRUE | N_Shore |
| 2953 | cg26157232 | -0.003 | 0.010209 | 16 | *CMTM4* | Body | NA | - |
| 2954 | cg01806928 | 0.004 | 0.010249 | 11 | *SYT9* | TSS1500 | TRUE | Island |
| 2955 | cg02679542 | -0.0031 | 0.010257 | 17 | *KIAA0753* | Body | NA | - |
| 2956 | cg03538236 | -0.0031 | 0.010253 | 16 | *LOC100128770* | TSS200 | NA | S_Shelf |
| 2957 | cg07291109 | -0.0057 | 0.010254 | 5 | *-* | - | NA | - |
| 2958 | cg16734615 | -0.0053 | 0.010257 | 12 | *SRGAP1* | Body | NA | - |
| 2959 | cg22518157 | 0.0026 | 0.010253 | 4 | *ARAP2* | Body | TRUE | - |
| 2960 | cg19084096 | 0.0028 | 0.010277 | 3 | *HTR3E* | 5'UTR | NA | - |
| 2961 | cg10334416 | -0.0051 | 0.010291 | 17 | *-* | - | TRUE | - |
| 2962 | cg18818075 | -0.004 | 0.010291 | 6 | *-* | - | TRUE | - |
| 2963 | cg18886109 | -0.0029 | 0.010287 | 10 | *ADAMTS14* | Body | TRUE | - |
| 2964 | cg25287357 | -0.0036 | 0.010287 | 13 | *-* | - | NA | - |
| 2965 | cg06272282 | -8.00E-04 | 0.010347 | 1 | *MTF1* | TSS1500 | NA | S_Shore |
| 2966 | cg12696808 | -0.0023 | 0.010344 | 1 | *RPS6KA1* | Body | NA | S_Shore |
| 2967 | cg13523587 | -0.0017 | 0.010333 | 3 | *NISCH* | TSS1500 | NA | N_Shore |
| 2968 | cg00013684 | -0.0028 | 0.010357 | 20 | *BCL2L1* | Body | NA | - |
| 2969 | cg12424164 | 0.003 | 0.010347 | 8 | *LINC00051* | Body | NA | - |
| 2970 | cg15646614 | 0.0022 | 0.010364 | 10 | *-* | - | NA | - |
| 2971 | cg03996822 | -0.0042 | 0.010364 | 4 | *RASSF6* | TSS1500 | TRUE | S_Shore |
| 2972 | cg07870237 | 0.0025 | 0.010366 | 5 | *ACSL6* | TSS1500 | TRUE | S_Shore |
| 2973 | cg10908953 | -0.0022 | 0.010366 | 11 | *SORL1* | TSS1500 | TRUE | N_Shore |
| 2974 | cg16831114 | -0.0043 | 0.010377 | 7 | *CLDN15* | 5'UTR | NA | - |
| 2975 | cg26969150 | -0.0044 | 0.010389 | 8 | *POTEA* | TSS200 | TRUE | - |
| 2976 | cg10893268 | 0.0022 | 0.010418 | 1 | *BCAR3* | 5'UTR | NA | - |
| 2977 | cg21052869 | 0.0019 | 0.010455 | 5 | *ADAM19* | Body | NA | - |
| 2978 | cg09098195 | -0.0045 | 0.010475 | 4 | *BEND4* | TSS1500 | TRUE | S_Shore |
| 2979 | cg22697108 | -0.0035 | 0.010497 | 5 | *-* | - | TRUE | - |
| 2980 | cg08873673 | 0.0029 | 0.01051 | 19 | *FUT2* | TSS200 | TRUE | N_Shore |
| 2981 | cg16120485 | -0.0039 | 0.010539 | 16 | *-* | - | NA | - |
| 2982 | cg00515023 | -0.0033 | 0.010547 | 15 | *-* | - | NA | - |
| 2983 | cg07794938 | -0.0043 | 0.01056 | 4 | *SLIT2* | Body | NA | - |
| 2984 | cg14333555 | -0.0036 | 0.01056 | X | *-* | - | NA | - |
| 2985 | cg03233896 | -0.0049 | 0.010564 | 3 | *MYLK* | Body | NA | - |
| 2986 | cg08159120 | -0.0031 | 0.010564 | 9 | *TMC1* | 5'UTR | NA | - |
| 2987 | cg13072943 | -0.0056 | 0.01056 | 6 | *RPS6KA2* | Body | TRUE | - |
| 2988 | cg16411103 | -0.0032 | 0.010564 | 13 | *STARD13* | 5'UTR | TRUE | - |
| 2989 | cg26557890 | -0.0038 | 0.010564 | 1 | *FAM78B* | Body | NA | - |
| 2990 | cg04060751 | -0.0025 | 0.010578 | 4 | *-* | - | NA | - |
| 2991 | cg05615966 | -0.0025 | 0.010578 | 20 | *-* | - | NA | - |
| 2992 | cg06909254 | 0.002 | 0.010578 | 2 | *ST3GAL5* | Body | TRUE | - |
| 2993 | cg13798384 | -0.0026 | 0.010578 | 13 | *PCDH9* | Body | TRUE | - |
| 2994 | cg26147845 | -0.0047 | 0.010581 | 12 | *EP400* | TSS1500 | TRUE | N_Shore |
| 2995 | cg15186576 | -0.005 | 0.010605 | 14 | *EGLN3* | Body | NA | - |
| 2996 | cg07402322 | -0.0029 | 0.010605 | 11 | *-* | - | NA | - |
| 2997 | cg22971191 | 0.0064 | 0.010628 | 13 | *SLC10A2* | TSS1500 | TRUE | - |
| 2998 | cg05683544 | -0.0033 | 0.010653 | 11 | *CLPB* | Body | NA | N_Shelf |
| 2999 | cg06646572 | -0.002 | 0.010662 | 4 | *-* | - | NA | - |
| 3000 | cg08119655 | 0.008 | 0.010653 | 11 | *PPFIBP2* | Body | TRUE | - |
| 3001 | cg13273374 | -0.0022 | 0.010662 | 6 | *-* | - | NA | - |
| 3002 | cg17836612 | 0.0042 | 0.010653 | 17 | *LGALS3BP* | TSS1500 | TRUE | - |
| 3003 | cg06626750 | -0.0026 | 0.010664 | 17 | *SFRS1* | 3'UTR | TRUE | N_Shore |
| 3004 | cg02100997 | 0.0012 | 0.010686 | 1 | *PTPRU* | Body | TRUE | N_Shore |
| 3005 | cg06492796 | -0.0027 | 0.010681 | 12 | *-* | - | TRUE | N_Shore |
| 3006 | cg06661266 | -0.0037 | 0.010687 | 3 | *-* | - | TRUE | N_Shelf |
| 3007 | cg09014329 | 0.0058 | 0.010682 | 17 | *FAM171A2* | Body | TRUE | Island |
| 3008 | cg11275153 | -0.0027 | 0.010681 | 2 | *LINC01122* | Body | NA | - |
| 3009 | cg11942629 | -0.003 | 0.010681 | 18 | *-* | - | NA | - |
| 3010 | cg18408264 | -0.0055 | 0.010687 | 1 | *VPS13D* | Body | NA | - |
| 3011 | cg04916802 | 0.0027 | 0.010687 | 13 | *MIR548F5* | Body | TRUE | Island |
| 3012 | cg13929106 | 0.005 | 0.010708 | 12 | *TMTC1* | Body | TRUE | - |
| 3013 | cg00589617 | 0.0088 | 0.010741 | 1 | *GALNT2* | 3'UTR | TRUE | Island |
| 3014 | cg06737494 | 0.0017 | 0.010751 | 3 | *GHSR* | TSS1500 | TRUE | Island |
| 3015 | cg14899624 | 0.002 | 0.010738 | 16 | *-* | - | NA | N_Shore |
| 3016 | cg19932169 | 0.0026 | 0.010789 | 15 | *KLF13* | Body | NA | - |
| 3017 | cg04839576 | -0.0024 | 0.010804 | 9 | *-* | - | NA | - |
| 3018 | cg11808699 | -0.0088 | 0.010805 | 15 | *IL16* | Body | TRUE | - |
| 3019 | cg08404702 | 0.0037 | 0.010824 | 16 | *TSC2* | Body | TRUE | N_Shore |
| 3020 | cg18990030 | 0.0081 | 0.010824 | 19 | *GRIN2D* | Body | NA | S_Shore |
| 3021 | cg09717923 | 0.0028 | 0.010839 | 16 | *-* | - | TRUE | Island |
| 3022 | cg12137273 | -0.0024 | 0.010839 | 2 | *EPC2* | TSS1500 | NA | N_Shore |
| 3023 | cg01715299 | -0.0027 | 0.010875 | 1 | *-* | - | NA | - |
| 3024 | cg02821871 | 0.0032 | 0.010863 | 19 | *SPHK2* | 3'UTR | TRUE | Island |
| 3025 | cg16728604 | -0.0023 | 0.010863 | 1 | *KIAA1522* | TSS1500 | TRUE | N_Shore |
| 3026 | cg17037638 | 0.002 | 0.010878 | 11 | *ALKBH3* | Body | NA | - |
| 3027 | cg20697791 | 0.0053 | 0.01088 | 4 | *-* | - | NA | - |
| 3028 | cg21179705 | 0.0024 | 0.010886 | 5 | *-* | - | TRUE | - |
| 3029 | cg05684714 | -0.0034 | 0.01089 | 16 | *COG4* | Body | NA | - |
| 3030 | cg07049996 | -0.0033 | 0.01089 | 18 | *-* | - | NA | - |
| 3031 | cg14479354 | -0.0036 | 0.01089 | 1 | *-* | - | NA | - |
| 3032 | cg23737927 | -0.004 | 0.010921 | 12 | *-* | - | TRUE | N_Shelf |
| 3033 | cg22041417 | -0.004 | 0.010948 | 19 | *TPM4* | TSS200 | TRUE | N_Shore |
| 3034 | cg04625862 | 0.0024 | 0.010955 | 3 | *NME6* | Body | TRUE | N_Shore |
| 3035 | cg05061804 | -0.003 | 0.010951 | 9 | *TTF1* | TSS200 | TRUE | S_Shore |
| 3036 | cg11565377 | -0.0024 | 0.010951 | 3 | *MIR711* | TSS1500 | TRUE | - |
| 3037 | cg16180657 | -0.0037 | 0.010989 | 20 | *-* | - | NA | - |
| 3038 | cg27129754 | 0.0027 | 0.010981 | 19 | *PPP1R37* | Body | NA | - |
| 3039 | cg00430484 | 0.0021 | 0.011022 | 22 | *BAIAP2L2* | Body | TRUE | Island |
| 3040 | cg25843936 | -0.0031 | 0.011038 | 7 | *RSBN1L* | Body | NA | - |
| 3041 | cg02596819 | 0.0022 | 0.011044 | 16 | *USP7* | Body | TRUE | N_Shore |
| 3042 | cg02774128 | 0.0066 | 0.011039 | 11 | *-* | - | NA | N_Shelf |
| 3043 | cg12634236 | 0.0011 | 0.011049 | 2 | *TBR1* | Body | NA | Island |
| 3044 | cg01431244 | -0.0025 | 0.011068 | 17 | *PTRF* | TSS1500 | NA | S_Shore |
| 3045 | cg11405123 | -0.0035 | 0.011056 | 9 | *DAPK1* | Body | NA | - |
| 3046 | cg12640662 | -0.0031 | 0.011058 | 18 | *DSG2* | Body | NA | - |
| 3047 | cg02930866 | -0.0028 | 0.011081 | 3 | *EAF1* | 3'UTR | TRUE | - |
| 3048 | cg08922729 | -0.0036 | 0.011074 | 1 | *-* | - | TRUE | - |
| 3049 | cg23275644 | -0.003 | 0.011081 | 5 | *ROPN1L* | Body | TRUE | - |
| 3050 | cg09589646 | -0.004 | 0.011082 | 3 | *-* | - | NA | - |
| 3051 | cg11147940 | -0.0031 | 0.011098 | 1 | *-* | - | NA | - |
| 3052 | cg11622592 | -0.0021 | 0.011098 | 2 | *EFHD1* | Body | NA | - |
| 3053 | cg21824733 | 0.0033 | 0.011082 | 15 | *EHD4* | Body | TRUE | S_Shelf |
| 3054 | cg10341152 | 0.0018 | 0.011139 | 13 | *RFXAP* | TSS1500 | TRUE | N_Shore |
| 3055 | cg16508098 | -0.0071 | 0.011131 | 5 | *SMAD5AS1* | Body | NA | N_Shore |
| 3056 | cg17168836 | -0.0041 | 0.011126 | 1 | *GNG12* | 5'UTR | TRUE | - |
| 3057 | cg12142278 | -0.004 | 0.011151 | X | *-* | - | NA | - |
| 3058 | cg27494383 | 7.00E-04 | 0.011139 | 15 | *LTK* | 1stExon | TRUE | S_Shore |
| 3059 | cg13732420 | -0.003 | 0.01117 | 5 | *FGF1* | 5'UTR | NA | - |
| 3060 | cg25386151 | -0.0017 | 0.011163 | 1 | *ATF3* | 5'UTR | TRUE | N_Shore |
| 3061 | cg08787832 | 0.0045 | 0.011195 | 6 | *PACSIN1* | 5'UTR | NA | - |
| 3062 | cg17291136 | -0.0025 | 0.011217 | 8 | *-* | - | TRUE | - |
| 3063 | cg00582231 | -0.0028 | 0.011221 | 8 | *RSPO2* | Body | NA | - |
| 3064 | cg03677924 | -0.0024 | 0.011226 | 12 | *MIP* | TSS1500 | TRUE | - |
| 3065 | cg06075311 | -0.0025 | 0.011229 | 10 | *CALHM2* | 5'UTR | TRUE | N_Shore |
| 3066 | cg07291349 | -0.0042 | 0.011226 | 4 | *APBB2* | Body | TRUE | - |
| 3067 | cg09001440 | -0.0099 | 0.011226 | 16 | *CA5A* | Body | TRUE | - |
| 3068 | cg12238465 | -0.0026 | 0.011221 | 16 | *-* | - | NA | N_Shelf |
| 3069 | cg01165142 | -0.0043 | 0.011238 | 16 | *IL4R* | Body | TRUE | - |
| 3070 | cg18986925 | -0.0043 | 0.011238 | 2 | *C2orf39* | Body | TRUE | - |
| 3071 | cg21531416 | 0.0036 | 0.011238 | 11 | *DEAF1* | Body | TRUE | S_Shelf |
| 3072 | cg05919454 | 0.0029 | 0.011252 | 9 | *-* | - | NA | - |
| 3073 | cg20062413 | -0.0044 | 0.011252 | 2 | *CD302* | TSS1500 | NA | S_Shore |
| 3074 | cg17733594 | 0.0021 | 0.011273 | 1 | *SIPA1L2* | Body | TRUE | - |
| 3075 | cg05011554 | -0.0043 | 0.011298 | 10 | *SH3PXD2A* | Body | NA | - |
| 3076 | cg09612304 | -0.0088 | 0.011294 | 6 | *-* | - | NA | - |
| 3077 | cg12531498 | 0.0032 | 0.011294 | 12 | *-* | - | TRUE | N_Shore |
| 3078 | cg09005399 | -0.005 | 0.011314 | 11 | *GAL3ST3* | Body | TRUE | Island |
| 3079 | cg21002456 | 0.0028 | 0.011312 | 17 | *CEP295NL* | TSS1500 | NA | S_Shelf |
| 3080 | cg27056819 | -0.0034 | 0.011312 | 6 | *DSE* | 5'UTR | NA | - |
| 3081 | cg27426436 | 0.0054 | 0.011314 | 19 | *KLHL26* | Body | TRUE | N_Shelf |
| 3082 | cg11532433 | -0.0077 | 0.011323 | 2 | *-* | - | TRUE | - |
| 3083 | cg22880122 | -0.0066 | 0.011323 | 6 | *HCG22* | Body | TRUE | - |
| 3084 | cg25866895 | -0.0079 | 0.011322 | 11 | *GSTP1* | TSS1500 | TRUE | N_Shore |
| 3085 | cg25934997 | -0.0073 | 0.011317 | 5 | *SYNPO* | 5'UTR | NA | - |
| 3086 | cg06553312 | 0.0016 | 0.011328 | 19 | *MYH14* | TSS200 | TRUE | Island |
| 3087 | cg14305711 | -0.0035 | 0.011329 | 2 | *COMMD1* | TSS1500 | TRUE | - |
| 3088 | cg14841103 | 0.0024 | 0.011328 | 16 | *E4F1* | 1stExon | NA | S_Shore |
| 3089 | cg18580117 | -0.0037 | 0.011328 | 15 | *S-P23* | TSS1500 | TRUE | S_Shelf |
| 3090 | cg26332061 | 0.0015 | 0.011328 | 20 | *EBF4* | Body | TRUE | Island |
| 3091 | cg04398950 | 0.0014 | 0.011375 | 14 | *AMN* | Body | TRUE | Island |
| 3092 | cg23579199 | 0.0031 | 0.01137 | 2 | *FBXO41* | TSS1500 | NA | S_Shore |
| 3093 | cg01946401 | -0.0076 | 0.011391 | 6 | *SUPT3H* | 5'UTR | TRUE | - |
| 3094 | cg13721246 | 0.0044 | 0.011378 | 12 | *D-H10* | Body | NA | - |
| 3095 | cg17267171 | -0.0047 | 0.011396 | 3 | *-* | - | NA | - |
| 3096 | cg07787996 | -0.0036 | 0.011409 | 1 | *RERE* | 5'UTR | NA | - |
| 3097 | cg00000776 | -0.0037 | 0.011426 | 4 | *-* | - | NA | - |
| 3098 | cg03415494 | -0.0027 | 0.01142 | 5 | *SIL1* | Body | NA | - |
| 3099 | cg16169166 | -0.0035 | 0.01142 | 3 | *-* | - | NA | - |
| 3100 | cg06319762 | 0.0025 | 0.011447 | 16 | *RPS2* | TSS1500 | TRUE | S_Shore |
| 3101 | cg21847909 | -0.0041 | 0.011433 | 6 | *-* | - | NA | - |
| 3102 | cg22345205 | 0.0024 | 0.011439 | 1 | *TNFRSF18* | Body | NA | N_Shore |
| 3103 | cg13409704 | 0.0036 | 0.011453 | 22 | *-* | - | TRUE | Island |
| 3104 | cg22543892 | -0.0031 | 0.011451 | 7 | *-* | - | TRUE | - |
| 3105 | cg21757387 | 0.0066 | 0.011466 | 6 | *-* | - | NA | - |
| 3106 | cg05107303 | -0.0031 | 0.011509 | 9 | *MAMDC2* | Body | NA | - |
| 3107 | cg01231075 | -0.0031 | 0.01151 | 1 | *RAP1A* | 5'UTR | NA | - |
| 3108 | cg05237543 | 0.0011 | 0.01151 | 8 | *MTDH* | TSS1500 | TRUE | N_Shore |
| 3109 | cg19662109 | 0.0023 | 0.01151 | 21 | *-* | - | NA | - |
| 3110 | cg21207019 | -0.0038 | 0.011512 | 22 | *TRIOBP* | Body | NA | - |
| 3111 | cg24250902 | 0.0083 | 0.01151 | 1 | *GALNT2* | 3'UTR | TRUE | Island |
| 3112 | cg05517697 | 0.003 | 0.011534 | 5 | *RNF44* | 3'UTR | TRUE | - |
| 3113 | cg08823864 | -0.004 | 0.011555 | 11 | *LPXN* | TSS1500 | NA | N_Shore |
| 3114 | cg09727097 | -0.0014 | 0.011555 | 10 | *-* | - | NA | N_Shelf |
| 3115 | cg11743675 | 0.0104 | 0.011555 | 12 | *CNTN1* | 5'UTR | TRUE | - |
| 3116 | cg20149168 | 0.0033 | 0.011543 | 1 | *DIRAS3* | Body | TRUE | Island |
| 3117 | cg16148960 | -0.0028 | 0.011573 | 3 | *NLGN1* | Body | NA | - |
| 3118 | cg09957492 | -0.0033 | 0.01159 | 3 | *-* | - | NA | - |
| 3119 | cg05246110 | 0.0025 | 0.0116 | 11 | *SAPS3* | 5'UTR | TRUE | - |
| 3120 | cg14598480 | -0.0031 | 0.0116 | 11 | *RASSF7* | TSS1500 | NA | N_Shore |
| 3121 | cg16810969 | 0.0025 | 0.0116 | 1 | *LINC01135* | Body | NA | - |
| 3122 | cg24085171 | -0.0087 | 0.0116 | 5 | *CTD3080P12.3* | Body | NA | - |
| 3123 | cg13668490 | -0.0043 | 0.011648 | 17 | *ITGA2B* | Body | TRUE | S_Shore |
| 3124 | cg25147026 | -0.005 | 0.011668 | 1 | *PTGS2* | TSS1500 | TRUE | S_Shore |
| 3125 | cg03737975 | 0.0026 | 0.011685 | 2 | *LOC101927156* | Body | NA | - |
| 3126 | cg04091927 | 0.0039 | 0.011676 | 8 | *-* | - | TRUE | N_Shore |
| 3127 | cg04361926 | -0.0036 | 0.011676 | 16 | *-* | - | TRUE | N_Shore |
| 3128 | cg11126410 | 0.0078 | 0.011679 | 3 | *RAB43* | TSS1500 | NA | S_Shore |
| 3129 | cg22688447 | -0.005 | 0.011676 | 5 | *PPARGC1B* | TSS1500 | NA | N_Shore |
| 3130 | cg03095055 | 0.0039 | 0.011705 | 19 | *SPHK2* | 3'UTR | NA | Island |
| 3131 | cg12706938 | 0.002 | 0.011696 | 19 | *MEX3D* | Body | TRUE | Island |
| 3132 | cg00407231 | 0.0047 | 0.011722 | 1 | *KLHDC8A* | Body | TRUE | N_Shore |
| 3133 | cg00806461 | 0.0029 | 0.011721 | 3 | *FAM43A* | 1stExon | TRUE | Island |
| 3134 | cg01682111 | 0.0058 | 0.011722 | 16 | *UNKL* | TSS1500 | TRUE | Island |
| 3135 | cg12164614 | 0.0023 | 0.011736 | 17 | *DHX58* | Body | TRUE | S_Shelf |
| 3136 | cg01140244 | 0.0017 | 0.011741 | 10 | *INPP5A* | Body | TRUE | - |
| 3137 | cg11557257 | -0.0043 | 0.011736 | 3 | *-* | - | NA | - |
| 3138 | cg11931531 | -0.0023 | 0.011736 | 2 | *-* | - | NA | - |
| 3139 | cg12284566 | 0.0027 | 0.011736 | 1 | *SCMH1* | 5'UTR | NA | - |
| 3140 | cg19904126 | 0.0029 | 0.011741 | 22 | *MED15* | Body | NA | - |
| 3141 | cg21867846 | -0.0028 | 0.011736 | 1 | *-* | - | NA | - |
| 3142 | cg02330078 | -0.0031 | 0.01179 | 5 | *-* | - | NA | - |
| 3143 | cg03212133 | -0.0042 | 0.011814 | X | *MMGT1* | TSS1500 | TRUE | S_Shore |
| 3144 | cg08428985 | -0.0044 | 0.011834 | 6 | *-* | - | TRUE | N_Shore |
| 3145 | cg08247564 | -0.0113 | 0.011839 | 12 | *SLC4A8* | 5'UTR | NA | - |
| 3146 | cg08443014 | -0.0027 | 0.01186 | 2 | *LYPD6* | 5'UTR | NA | - |
| 3147 | cg12903966 | 0.0023 | 0.01186 | 5 | *SCAMP1* | Body | NA | - |
| 3148 | cg19589317 | 0.0034 | 0.01186 | 15 | *CHSY1* | Body | TRUE | - |
| 3149 | cg06998507 | -0.002 | 0.01189 | 16 | *KCNG4* | TSS1500 | TRUE | S_Shelf |
| 3150 | cg12473916 | -0.0029 | 0.011919 | 1 | *SHC1* | 5'UTR | TRUE | N_Shelf |
| 3151 | cg10492559 | 0.0019 | 0.011924 | 1 | *ESRRG* | TSS1500 | NA | - |
| 3152 | cg16088822 | -0.0022 | 0.011924 | 19 | *-* | - | NA | N_Shelf |
| 3153 | cg16996682 | 0.0037 | 0.011924 | 1 | *-* | - | TRUE | - |
| 3154 | cg20846768 | -0.0066 | 0.011921 | 20 | *-* | - | NA | - |
| 3155 | cg24055029 | 0.0072 | 0.011943 | 6 | *TNXB* | Body | TRUE | Island |
| 3156 | cg01209168 | 0.0021 | 0.01196 | 3 | *ARL8B* | Body | NA | - |
| 3157 | cg07226239 | -0.011 | 0.01196 | 6 | *PARK2* | Body | NA | - |
| 3158 | cg24601412 | -0.0013 | 0.012005 | 7 | *-* | - | NA | S_Shore |
| 3159 | cg24912415 | 0.0051 | 0.012001 | 20 | *MYH7B* | Body | TRUE | Island |
| 3160 | cg19925780 | -0.004 | 0.012052 | 1 | *-* | - | TRUE | - |
| 3161 | cg00068745 | -0.0047 | 0.012067 | 19 | *TPM4* | TSS200 | NA | N_Shore |
| 3162 | cg03668323 | 0.0024 | 0.012067 | 10 | *ABLIM1* | 5'UTR | NA | - |
| 3163 | cg14632165 | -0.0029 | 0.012067 | 14 | *-* | - | NA | - |
| 3164 | cg18460186 | -0.0023 | 0.012067 | 18 | *DLGAP1* | Body | NA | - |
| 3165 | cg24905290 | -0.0023 | 0.012067 | X | *-* | - | NA | - |
| 3166 | cg06374595 | -0.0029 | 0.012071 | 2 | *MEIS1* | Body | NA | - |
| 3167 | cg18327056 | 0.0029 | 0.012112 | 2 | *KCNK3* | TSS1500 | TRUE | N_Shore |
| 3168 | cg14878128 | -0.0037 | 0.01212 | 7 | *ABCB5* | TSS200 | TRUE | - |
| 3169 | cg20293653 | -0.0048 | 0.012134 | 11 | *-* | - | NA | - |
| 3170 | cg00307819 | 0.0024 | 0.012148 | 17 | *FAM117A* | Body | NA | - |
| 3171 | cg02593612 | 0.0025 | 0.012139 | 1 | *HIVEP3* | 5'UTR | NA | - |
| 3172 | cg13861180 | -0.0032 | 0.012165 | 9 | *GRIN1* | Body | TRUE | N_Shore |
| 3173 | cg20237595 | -0.0044 | 0.012162 | 3 | *KALRN* | Body | TRUE | N_Shelf |
| 3174 | cg15768146 | 0.0046 | 0.01217 | 14 | *ZFHX2* | 5'UTR | NA | - |
| 3175 | cg24555528 | 0.0024 | 0.012191 | 12 | *FMNL3* | Body | NA | - |
| 3176 | cg24736933 | -0.0033 | 0.012191 | 15 | *C15orf52* | TSS200 | TRUE | - |
| 3177 | cg00055747 | 0.0035 | 0.012218 | 6 | *-* | - | NA | - |
| 3178 | cg22005677 | 0.0027 | 0.01222 | 3 | *LPP* | Body | NA | - |
| 3179 | cg26251952 | 0.0038 | 0.01221 | 3 | *DGKG* | Body | NA | - |
| 3180 | cg01647632 | 0.0018 | 0.012228 | 15 | *HAPLN3* | TSS200 | TRUE | S_Shore |
| 3181 | cg17453778 | 0.0024 | 0.012222 | 3 | *CASR* | 5'UTR | TRUE | N_Shore |
| 3182 | cg22705959 | 0.0031 | 0.012234 | 2 | *-* | - | TRUE | S_Shore |
| 3183 | cg12250581 | -0.0043 | 0.012249 | 12 | *-* | - | TRUE | - |
| 3184 | cg01675440 | -0.0023 | 0.012249 | 17 | *-* | - | NA | - |
| 3185 | cg04527918 | 0.0044 | 0.012253 | 2 | *UCN* | TSS200 | TRUE | Island |
| 3186 | cg05346619 | -0.0113 | 0.01225 | 7 | *-* | - | NA | - |
| 3187 | cg07815522 | 0.0072 | 0.012249 | 3 | *PARP9* | 5'UTR | NA | N_Shore |
| 3188 | cg09939947 | -0.0031 | 0.012249 | 16 | *-* | - | NA | - |
| 3189 | cg11491420 | 0.0024 | 0.01225 | 2 | *THAP4* | Body | NA | - |
| 3190 | cg18685879 | -0.0039 | 0.012249 | 3 | *ACPL2* | 3'UTR | TRUE | - |
| 3191 | cg25782440 | -0.0022 | 0.012249 | 19 | *MAP2K7* | 3'UTR | TRUE | N_Shore |
| 3192 | cg03979714 | -0.0032 | 0.01226 | 1 | *FAM78B* | Body | NA | - |
| 3193 | cg05904155 | -0.0023 | 0.012262 | 18 | *DT-* | 5'UTR | NA | - |
| 3194 | cg14502172 | -0.0026 | 0.01226 | 10 | *NET1* | TSS1500 | TRUE | N_Shore |
| 3195 | cg22156842 | -0.0067 | 0.012259 | 3 | *TMEM22* | TSS1500 | TRUE | N_Shore |
| 3196 | cg00311984 | 0.0038 | 0.01227 | 8 | *PLEC1* | TSS1500 | TRUE | Island |
| 3197 | cg04098052 | -0.0054 | 0.01227 | 7 | *-* | - | TRUE | - |
| 3198 | cg07986469 | -0.002 | 0.01227 | 10 | *PTPRE* | 5'UTR | TRUE | - |
| 3199 | cg09880765 | -0.0039 | 0.01227 | 15 | *-* | - | NA | - |
| 3200 | cg13889684 | -0.0106 | 0.01227 | X | *RHOXF1AS1* | Body | NA | - |
| 3201 | cg04184571 | -0.0034 | 0.012303 | 5 | *-* | - | NA | - |
| 3202 | cg06869761 | -0.0068 | 0.012303 | 2 | *OLA1* | Body | NA | - |
| 3203 | cg12524338 | -0.0068 | 0.012303 | 4 | *-* | - | TRUE | Island |
| 3204 | cg25129541 | -0.0036 | 0.012297 | 12 | *-* | - | TRUE | N_Shore |
| 3205 | cg00879302 | 0.0032 | 0.012307 | 17 | *ABR* | Body | TRUE | N_Shore |
| 3206 | cg20805155 | 0.0022 | 0.01232 | 17 | *TBX2* | Body | NA | Island |
| 3207 | cg26682566 | -0.0073 | 0.012341 | 4 | *-* | - | NA | - |
| 3208 | cg15106656 | 0.002 | 0.012362 | 13 | *GGACT* | 5'UTR | NA | - |
| 3209 | cg20964505 | 0.0127 | 0.012363 | 2 | *-* | - | NA | - |
| 3210 | cg01987393 | -0.0063 | 0.012365 | 7 | *-* | - | TRUE | - |
| 3211 | cg26088163 | -0.0037 | 0.012402 | 6 | *ANKRD6* | 5'UTR | NA | S_Shelf |
| 3212 | cg01844757 | -0.002 | 0.01242 | 5 | *MCC* | Body | NA | - |
| 3213 | cg15284655 | 0.0018 | 0.01242 | 2 | *KCNG3* | 1stExon | NA | Island |
| 3214 | cg01154445 | -0.0035 | 0.01243 | 2 | *NRP2* | Body | TRUE | N_Shore |
| 3215 | cg17461972 | -0.0018 | 0.012446 | 11 | *MADD* | TSS1500 | NA | N_Shore |
| 3216 | cg04004810 | 0.0022 | 0.012469 | 6 | *-* | - | NA | - |
| 3217 | cg27330283 | -0.0031 | 0.012497 | 1 | *-* | - | NA | - |
| 3218 | cg03735592 | -0.0038 | 0.012506 | 6 | *NHSL1* | TSS1500 | TRUE | - |
| 3219 | cg19627034 | -0.0026 | 0.012506 | 1 | *-* | - | TRUE | - |
| 3220 | cg25769469 | -0.0031 | 0.012506 | 5 | *PTCD2* | Body | TRUE | - |
| 3221 | cg06231995 | 0.0014 | 0.012528 | 9 | *-* | - | TRUE | Island |
| 3222 | cg12190504 | -0.0021 | 0.012528 | 4 | *RNF150* | Body | NA | - |
| 3223 | cg12103569 | 0.0036 | 0.01255 | 12 | *SFRS8* | Body | TRUE | - |
| 3224 | cg04239194 | -0.0087 | 0.012573 | 6 | *HBS1L* | Body | NA | - |
| 3225 | cg02981828 | -0.0047 | 0.012584 | 12 | *GLIPR1L2* | TSS1500 | NA | N_Shore |
| 3226 | cg21229608 | 0.0025 | 0.012601 | 5 | *-* | - | NA | - |
| 3227 | cg01235057 | -0.0025 | 0.012603 | 9 | *0* | TSS1500 | NA | S_Shore |
| 3228 | cg11485154 | -0.0031 | 0.01261 | 11 | *-* | - | TRUE | S_Shore |
| 3229 | cg05980592 | 0.0045 | 0.01263 | 3 | *-* | - | TRUE | - |
| 3230 | cg07822980 | -0.0029 | 0.01262 | 6 | *-* | - | NA | - |
| 3231 | cg11299837 | -0.0053 | 0.01263 | 15 | *LOC101928134* | Body | NA | N_Shore |
| 3232 | cg15773678 | 0.002 | 0.01263 | 7 | *CREB5* | Body | NA | - |
| 3233 | cg05245822 | -0.0049 | 0.012692 | 18 | *-* | - | NA | - |
| 3234 | cg08052292 | 0.0022 | 0.012692 | 3 | *ARHGEF3* | Body | TRUE | - |
| 3235 | cg21002528 | -0.0023 | 0.012682 | 11 | *CRY2* | 3'UTR | TRUE | N_Shelf |
| 3236 | cg16034393 | 0.005 | 0.012703 | 20 | *NCOA3* | Body | NA | - |
| 3237 | cg21922478 | -0.0102 | 0.012695 | 5 | *ITGA1* | Body | NA | - |
| 3238 | cg22158648 | -0.0036 | 0.012705 | 18 | *CABLES1* | TSS1500 | TRUE | N_Shore |
| 3239 | cg03638795 | -0.0035 | 0.012707 | 11 | *SIGIRR* | 5'UTR | TRUE | Island |
| 3240 | cg25014419 | -0.0027 | 0.012717 | 2 | *-* | - | NA | - |
| 3241 | cg03748473 | 0.0025 | 0.012738 | 11 | *UBASH3B* | Body | NA | - |
| 3242 | cg23481102 | -0.003 | 0.012728 | 7 | *-* | - | NA | - |
| 3243 | cg21986665 | -0.003 | 0.012759 | 1 | *-* | - | NA | - |
| 3244 | cg03086022 | -0.0021 | 0.012766 | 22 | *PPM1F* | Body | NA | N_Shore |
| 3245 | cg17642041 | 0.0028 | 0.012765 | 3 | *CCR9* | TSS200 | TRUE | - |
| 3246 | cg01101556 | 0.0021 | 0.01278 | 6 | *CASC15* | Body | NA | - |
| 3247 | cg10176717 | -0.0058 | 0.012782 | 10 | *-* | - | NA | - |
| 3248 | cg12019755 | -0.0018 | 0.012782 | 17 | *DLG4* | Body | NA | S_Shore |
| 3249 | cg23512157 | -0.0044 | 0.012782 | 11 | *DLG2* | Body | NA | - |
| 3250 | cg05713859 | -0.0024 | 0.012792 | 5 | *PCBD2* | TSS1500 | TRUE | N_Shore |
| 3251 | cg10874644 | -0.0056 | 0.012794 | 5 | *-* | - | TRUE | - |
| 3252 | cg16540258 | -0.0011 | 0.012792 | 12 | *-* | - | NA | - |
| 3253 | cg21407059 | 0.0024 | 0.012792 | 16 | *-* | - | NA | S_Shore |
| 3254 | cg06121784 | -0.0038 | 0.012796 | 15 | *-* | - | NA | - |
| 3255 | cg08972190 | 0.0024 | 0.012807 | 7 | *MAD1L1* | Body | TRUE | - |
| 3256 | cg14368276 | -0.0047 | 0.012796 | 3 | *P2RY12* | TSS200 | NA | - |
| 3257 | cg24248329 | 0.0022 | 0.012796 | 1 | *NFYC* | 1stExon | NA | - |
| 3258 | cg16195752 | 0.0033 | 0.012859 | 1 | *FMO1* | 5'UTR | NA | - |
| 3259 | cg09399610 | -0.0034 | 0.012875 | 5 | *LOC285696* | Body | NA | - |
| 3260 | cg18493884 | -0.0034 | 0.012875 | 4 | *HTT* | Body | NA | - |
| 3261 | cg03090301 | -0.0041 | 0.012897 | 4 | *SPARCL1* | Body | NA | - |
| 3262 | cg04766061 | -0.003 | 0.012909 | 17 | *-* | - | TRUE | N_Shelf |
| 3263 | cg12145509 | -0.0025 | 0.012897 | 15 | *DPH6* | Body | NA | - |
| 3264 | cg03288304 | -0.0037 | 0.012916 | 2 | *SLC9A4* | Body | NA | - |
| 3265 | cg15415882 | -0.0042 | 0.012924 | 3 | *XXYLT1* | Body | NA | - |
| 3266 | cg10663081 | -0.0021 | 0.012945 | 20 | *-* | - | NA | - |
| 3267 | cg18462381 | 0.003 | 0.012947 | 10 | *FOXI2* | Body | TRUE | Island |
| 3268 | cg19281310 | -0.0016 | 0.012954 | 3 | *IL12AAS1* | Body | NA | - |
| 3269 | cg17809377 | 0.0046 | 0.013001 | 5 | *HK3* | TSS1500 | TRUE | - |
| 3270 | cg06459293 | -0.0019 | 0.013017 | 22 | *CELSR1* | Body | TRUE | Island |
| 3271 | cg06981309 | 0.0061 | 0.013017 | 3 | *PLSCR1* | 5'UTR | TRUE | N_Shore |
| 3272 | cg11101542 | 0.0021 | 0.013009 | 2 | *HAGLR* | TSS1500 | NA | Island |
| 3273 | cg21029357 | 0.0026 | 0.013009 | 11 | *FADS2* | Body | NA | - |
| 3274 | cg01414567 | -0.0066 | 0.013029 | 12 | *-* | - | NA | - |
| 3275 | cg06235469 | 0.0049 | 0.013019 | 10 | *-* | - | NA | - |
| 3276 | cg21513437 | -0.0014 | 0.013019 | 17 | *-* | - | TRUE | - |
| 3277 | cg10887181 | -0.0028 | 0.013031 | 2 | *TUBA4B* | Body | NA | - |
| 3278 | cg00888249 | -0.0045 | 0.013059 | 4 | *GALNTL6* | Body | NA | - |
| 3279 | cg07628784 | -0.0036 | 0.013059 | 7 | *UMAD1* | 5'UTR | NA | - |
| 3280 | cg15599875 | -0.0045 | 0.013059 | 11 | *-* | - | TRUE | - |
| 3281 | cg18486150 | 0.0025 | 0.013059 | 1 | *KIF17* | TSS1500 | TRUE | S_Shore |
| 3282 | cg00117066 | -0.0033 | 0.013059 | X | *GRPR* | TSS1500 | TRUE | - |
| 3283 | cg02286081 | -0.006 | 0.013062 | 6 | *HLADPB1* | 1stExon | TRUE | - |
| 3284 | cg04524040 | 0.003 | 0.013059 | 19 | *CREB3L3* | TSS1500 | TRUE | - |
| 3285 | cg13835114 | -0.0028 | 0.013062 | 9 | *ASS1* | 5'UTR | TRUE | - |
| 3286 | cg01125058 | -0.0017 | 0.013083 | 8 | *ZNF704* | Body | NA | - |
| 3287 | cg08798307 | 0.0026 | 0.013083 | 7 | *-* | - | TRUE | - |
| 3288 | cg20103150 | -0.0048 | 0.013072 | 4 | *-* | - | TRUE | - |
| 3289 | cg13431515 | 0.0045 | 0.013084 | 12 | *TMTC1* | Body | NA | - |
| 3290 | cg04726374 | 0.0042 | 0.013104 | 17 | *PTRF* | 3'UTR | TRUE | N_Shelf |
| 3291 | cg07001914 | 0.0021 | 0.013104 | 22 | *MAFF* | Body | NA | N_Shore |
| 3292 | cg16281776 | -0.005 | 0.013104 | 1 | *PRELP* | TSS1500 | NA | - |
| 3293 | cg19655569 | -0.0024 | 0.013105 | 13 | *FAM155A* | Body | NA | - |
| 3294 | cg27493344 | -0.002 | 0.013104 | 4 | *GPRIN3* | 5'UTR | NA | - |
| 3295 | cg07647164 | 0.0017 | 0.01311 | 1 | *-* | - | TRUE | Island |
| 3296 | cg18055007 | 0.0036 | 0.013119 | 6 | *DDAH2* | TSS200 | TRUE | Island |
| 3297 | cg24259244 | 0.0015 | 0.013133 | 13 | *ZIC5* | TSS200 | TRUE | Island |
| 3298 | cg25371036 | -0.0028 | 0.013133 | 11 | *AMOTL1* | TSS1500 | TRUE | N_Shore |
| 3299 | cg27570326 | 0.0018 | 0.01313 | 11 | *TNNT3* | TSS200 | NA | - |
| 3300 | cg10403394 | -0.007 | 0.013155 | 15 | *TPM1* | Body | TRUE | - |
| 3301 | cg03350491 | -0.0021 | 0.013166 | 20 | *SLC35C2* | Body | NA | - |
| 3302 | cg19644580 | -0.0037 | 0.013166 | 21 | *C21orf91* | Body | NA | - |
| 3303 | cg17107239 | -0.0068 | 0.013188 | 4 | *RXFP1* | Body | NA | - |
| 3304 | cg10057309 | -0.0051 | 0.013195 | 11 | *-* | - | NA | - |
| 3305 | cg09690989 | -0.0033 | 0.013226 | 17 | *C1QTNF1* | 3'UTR | TRUE | S_Shore |
| 3306 | cg05634637 | -0.0074 | 0.013243 | 5 | *CDH6* | 5'UTR | TRUE | S_Shelf |
| 3307 | cg09043230 | -0.0023 | 0.013241 | 16 | *AP1G1* | TSS1500 | NA | S_Shore |
| 3308 | cg19075787 | -0.0056 | 0.013241 | 2 | *-* | - | TRUE | - |
| 3309 | cg23070738 | -0.0049 | 0.013243 | 6 | *-* | - | NA | - |
| 3310 | cg10337377 | 0.0016 | 0.013245 | 16 | *-* | - | NA | - |
| 3311 | cg10891482 | -0.0025 | 0.013245 | 11 | *MS4A8B* | 1stExon | TRUE | - |
| 3312 | cg26197136 | -0.0039 | 0.013245 | 6 | *CNKSR3* | Body | NA | - |
| 3313 | cg01047555 | 0.0026 | 0.013276 | 6 | *C6orf174* | Body | TRUE | Island |
| 3314 | cg19663246 | -0.0054 | 0.013257 | 7 | *-* | - | TRUE | S_Shore |
| 3315 | cg02222791 | -0.0048 | 0.013278 | 14 | *-* | - | TRUE | - |
| 3316 | cg22013747 | -0.0034 | 0.013278 | 12 | *-* | - | NA | - |
| 3317 | cg07177395 | 0.0049 | 0.013296 | 14 | *NRXN3* | Body | TRUE | - |
| 3318 | cg09452027 | 0.0033 | 0.013298 | 11 | *SLC35C1* | TSS200 | TRUE | N_Shore |
| 3319 | cg12164925 | -0.0028 | 0.013295 | 17 | *-* | - | NA | - |
| 3320 | cg18686270 | 0.0046 | 0.013298 | 3 | *PLSCR1* | 5'UTR | TRUE | N_Shelf |
| 3321 | cg11287725 | -0.0045 | 0.013304 | 9 | *-* | - | NA | N_Shore |
| 3322 | cg01582261 | 0.0027 | 0.013335 | 18 | *-* | - | NA | - |
| 3323 | cg03078743 | -0.0034 | 0.013338 | 11 | *-* | - | NA | - |
| 3324 | cg01738198 | 0.0018 | 0.013349 | 14 | *-* | - | NA | - |
| 3325 | cg04058080 | -0.0022 | 0.013349 | 3 | *SH3BP5* | Body | NA | - |
| 3326 | cg17187735 | -0.0021 | 0.013349 | 20 | *RIN2* | Body | NA | - |
| 3327 | cg05092387 | 0.0055 | 0.013373 | 15 | *MEIS2* | Body | NA | - |
| 3328 | cg05306109 | 0.0022 | 0.013377 | 1 | *ITPKB* | Body | TRUE | N_Shore |
| 3329 | cg13934924 | -0.0032 | 0.013377 | 15 | *PCSK6* | Body | TRUE | N_Shore |
| 3330 | cg16876859 | -0.0025 | 0.013377 | 10 | *-* | - | TRUE | N_Shore |
| 3331 | cg05303901 | -0.0032 | 0.013386 | 7 | *CREB5* | TSS1500 | TRUE | - |
| 3332 | cg07081054 | -0.0028 | 0.013386 | 17 | *TOM1L1* | Body | TRUE | S_Shore |
| 3333 | cg20866375 | 0.0069 | 0.013386 | 17 | *TAX1BP3* | TSS1500 | TRUE | Island |
| 3334 | cg18091083 | 0.0109 | 0.013426 | 17 | *RPTOR* | Body | TRUE | S_Shore |
| 3335 | cg11643740 | -0.0036 | 0.013443 | 20 | *-* | - | NA | - |
| 3336 | cg16732650 | -0.0054 | 0.013444 | 1 | *-* | - | NA | - |
| 3337 | cg22663791 | -0.0036 | 0.013468 | 2 | *RBMS1* | Body | NA | - |
| 3338 | cg25711003 | -0.008 | 0.013468 | 3 | *PHLDB2* | 5'UTR | TRUE | - |
| 3339 | cg03562625 | -0.0027 | 0.013473 | 1 | *UCK2* | TSS1500 | NA | N_Shore |
| 3340 | cg04344127 | -0.0026 | 0.013473 | 9 | *C9orf139* | TSS200 | NA | N_Shore |
| 3341 | cg12754671 | -0.0031 | 0.01351 | 1 | *NDUFS2* | 5'UTR | TRUE | Island |
| 3342 | cg09516808 | -0.0033 | 0.013517 | X | *-* | - | NA | - |
| 3343 | cg16636334 | -0.0029 | 0.013518 | 12 | *TESC* | TSS1500 | NA | S_Shore |
| 3344 | cg21415530 | 0.0016 | 0.013518 | 8 | *KCNK9* | TSS1500 | TRUE | Island |
| 3345 | cg05317006 | -0.0031 | 0.01353 | 2 | *-* | - | NA | - |
| 3346 | cg05913590 | -0.0018 | 0.01353 | 6 | *JARID2* | Body | NA | - |
| 3347 | cg17193066 | -0.0016 | 0.01353 | 10 | *HERC4* | TSS1500 | TRUE | S_Shore |
| 3348 | cg17599983 | 0.0027 | 0.013532 | 17 | *PIGL* | 1stExon | NA | - |
| 3349 | cg00754408 | -0.0032 | 0.013558 | 9 | *PGM5* | Body | NA | - |
| 3350 | cg04932525 | 0.0025 | 0.01356 | 2 | *LIMS1* | TSS1500 | NA | - |
| 3351 | cg07327489 | 0.0033 | 0.01356 | 9 | *RAPGEF1* | Body | NA | - |
| 3352 | cg20063884 | -0.0035 | 0.01356 | 21 | *-* | - | NA | - |
| 3353 | cg25805864 | 0.0043 | 0.01356 | 8 | *MATN2* | Body | NA | - |
| 3354 | cg02772240 | -0.0021 | 0.013608 | 19 | *FBXO17* | TSS200 | NA | S_Shore |
| 3355 | cg25785236 | 0.002 | 0.013608 | 16 | *ZNF205AS1* | Body | NA | - |
| 3356 | cg03363743 | 0.0027 | 0.013628 | 17 | *SLC6A4* | 5'UTR | TRUE | Island |
| 3357 | cg07739179 | 0.0034 | 0.013628 | 17 | *#NAME?* | TSS1500 | TRUE | N_Shore |
| 3358 | cg10524346 | -0.0097 | 0.013628 | 6 | *PARK2* | Body | NA | - |
| 3359 | cg14370910 | -0.0033 | 0.013628 | 14 | *FLJ43390* | Body | TRUE | - |
| 3360 | cg23777387 | -0.0029 | 0.013628 | 20 | *-* | - | NA | - |
| 3361 | cg00497086 | 0.0023 | 0.013657 | 16 | *PRKCB* | Body | NA | - |
| 3362 | cg02170478 | -0.003 | 0.013652 | 4 | *PDGFRA* | 5'UTR | TRUE | S_Shore |
| 3363 | cg17716965 | 0.0014 | 0.013652 | 3 | *TLR9* | TSS200 | NA | - |
| 3364 | cg06483524 | -0.003 | 0.013672 | 17 | *LINC01563* | TSS1500 | NA | - |
| 3365 | cg15481583 | -0.0045 | 0.013689 | 1 | *ROR1* | Body | TRUE | - |
| 3366 | cg25633772 | -0.0033 | 0.013689 | 10 | *C10orf116* | TSS1500 | TRUE | N_Shore |
| 3367 | cg06883859 | -0.0027 | 0.013715 | 16 | *-* | - | NA | - |
| 3368 | cg20337160 | -0.0034 | 0.01372 | 3 | *-* | - | NA | - |
| 3369 | cg04414766 | -0.0127 | 0.013757 | 3 | *-* | - | NA | N_Shore |
| 3370 | cg23512275 | -0.0038 | 0.01374 | 21 | *LOC284837* | TSS200 | TRUE | - |
| 3371 | cg15452970 | 0.0034 | 0.013757 | 1 | *-* | - | TRUE | Island |
| 3372 | cg22114814 | 0.003 | 0.013757 | 11 | *-* | - | NA | - |
| 3373 | cg00352043 | -0.0025 | 0.013775 | 22 | *KDELR3* | TSS1500 | TRUE | N_Shore |
| 3374 | cg08194009 | -0.0047 | 0.013775 | 17 | *-* | - | TRUE | - |
| 3375 | cg20161212 | -0.0021 | 0.013775 | 2 | *-* | - | NA | - |
| 3376 | cg14731462 | -0.0051 | 0.013825 | 10 | *PTPRE* | 5'UTR | TRUE | - |
| 3377 | cg02846651 | -0.0028 | 0.013831 | 1 | *NFIA* | TSS1500 | NA | N_Shelf |
| 3378 | cg13725803 | -0.0026 | 0.01383 | 13 | *GUCY1B2* | TSS200 | TRUE | - |
| 3379 | cg15698548 | -0.003 | 0.01383 | 1 | *SYF2* | Body | NA | - |
| 3380 | cg26061048 | -0.0044 | 0.01383 | 6 | *-* | - | NA | - |
| 3381 | cg07807219 | -0.0032 | 0.013855 | 1 | *-* | - | TRUE | N_Shelf |
| 3382 | cg15081128 | -0.0024 | 0.013855 | 6 | *COL11A2* | Body | TRUE | - |
| 3383 | cg11438391 | -0.0021 | 0.013889 | 22 | *-* | - | NA | - |
| 3384 | cg16144395 | 0.0029 | 0.013891 | 18 | *-* | - | NA | - |
| 3385 | cg14821468 | 0.0022 | 0.013902 | 1 | *ADGRB2* | TSS1500 | NA | S_Shore |
| 3386 | cg26590588 | -0.0024 | 0.013901 | 14 | *PRKCH* | Body | NA | S_Shore |
| 3387 | cg09674468 | -0.005 | 0.013906 | 1 | *SLC35F3* | Body | TRUE | - |
| 3388 | cg05445320 | 0.0028 | 0.013933 | 20 | *ZMYND8* | TSS200 | NA | - |
| 3389 | cg18298966 | -0.0032 | 0.013933 | 1 | *-* | - | NA | - |
| 3390 | cg26189139 | 0.0035 | 0.013933 | 8 | *-* | - | TRUE | Island |
| 3391 | cg02659784 | -0.0029 | 0.013937 | 1 | *-* | - | NA | S_Shelf |
| 3392 | cg16431787 | -0.004 | 0.013936 | 6 | *LOC285768* | Body | TRUE | - |
| 3393 | cg19891728 | 0.0015 | 0.013937 | 8 | *ANK1* | TSS1500 | TRUE | Island |
| 3394 | cg22226832 | 0.0024 | 0.013936 | 8 | *SLA* | 1stExon | NA | - |
| 3395 | cg22687497 | -0.0031 | 0.013937 | 2 | *MIR7853* | Body | NA | - |
| 3396 | cg06443661 | -0.0042 | 0.013944 | 3 | *TNIK* | Body | NA | - |
| 3397 | cg11389953 | -0.0076 | 0.013944 | 8 | *-* | - | TRUE | S_Shore |
| 3398 | cg11459094 | 0.0031 | 0.013947 | 1 | *-* | - | NA | - |
| 3399 | cg12856183 | 0.0013 | 0.013973 | 15 | *-* | - | TRUE | Island |
| 3400 | cg14782372 | 0.002 | 0.013959 | 5 | *CXXC5* | 5'UTR | NA | S_Shore |
| 3401 | cg06740227 | -0.0079 | 0.013984 | 12 | *RASSF9* | Body | TRUE | - |
| 3402 | cg07242169 | 0.0018 | 0.014002 | 16 | *TMEM219* | 1stExon | NA | S_Shore |
| 3403 | cg10527525 | 0.003 | 0.014002 | 7 | *AUTS2* | Body | TRUE | S_Shore |
| 3404 | cg14378288 | -0.0051 | 0.013999 | 11 | *HRASLS2* | Body | NA | - |
| 3405 | cg07047501 | -0.003 | 0.014011 | 3 | *-* | - | NA | - |
| 3406 | cg24936095 | -0.003 | 0.014012 | 2 | *LIMS1* | TSS1500 | TRUE | - |
| 3407 | cg26969324 | 0.0028 | 0.014011 | 1 | *ACOT11* | Body | NA | - |
| 3408 | cg15877993 | 0.0018 | 0.01404 | 15 | *PAQR5* | Body | NA | - |
| 3409 | cg22171829 | -0.0069 | 0.014043 | 7 | *PDK4* | 1stExon | TRUE | Island |
| 3410 | cg23418219 | -0.0038 | 0.014043 | 7 | *CREB5* | TSS1500 | TRUE | - |
| 3411 | cg24116176 | 0.0024 | 0.014043 | 3 | *-* | - | NA | - |
| 3412 | cg25489477 | -0.0042 | 0.014043 | 2 | *-* | - | NA | - |
| 3413 | cg19404209 | 0.0036 | 0.014052 | 2 | *LIMS1* | Body | NA | - |
| 3414 | cg23213217 | 0.0027 | 0.014046 | 1 | *DEGS1* | TSS1500 | TRUE | N_Shore |
| 3415 | cg24231380 | 0.0035 | 0.01409 | 2 | *C2orf85* | Body | TRUE | N_Shore |
| 3416 | cg10745039 | -0.0041 | 0.014099 | 8 | *-* | - | NA | - |
| 3417 | cg01410876 | 0.0041 | 0.01412 | 19 | *LMTK3* | Body | TRUE | Island |
| 3418 | cg05006728 | -0.0019 | 0.01412 | 3 | *ST3GAL6* | TSS1500 | NA | N_Shore |
| 3419 | cg07465627 | -0.0045 | 0.01412 | 17 | *STXBP4* | Body | TRUE | - |
| 3420 | cg10955995 | -0.0059 | 0.01412 | 4 | *PPARGC1A* | Body | TRUE | - |
| 3421 | cg26937798 | -0.0018 | 0.01412 | 16 | *IL4R* | 5'UTR | TRUE | S_Shore |
| 3422 | cg27319536 | -0.009 | 0.014118 | 5 | *PPP2R2B* | 5'UTR | TRUE | S_Shelf |
| 3423 | cg00389857 | -0.0058 | 0.014121 | 11 | *ZBTB16* | Body | NA | - |
| 3424 | cg06183970 | -0.0036 | 0.014164 | 14 | *PPP1R13B* | Body | NA | - |
| 3425 | cg02693701 | 0.0036 | 0.014199 | 1 | *HIVEP3* | 5'UTR | NA | - |
| 3426 | cg09146183 | 0.0054 | 0.014195 | 22 | *MAFF* | Body | TRUE | Island |
| 3427 | cg10955910 | -0.0018 | 0.014215 | 7 | *D-JB6* | Body | NA | S_Shore |
| 3428 | cg13360866 | -0.002 | 0.014224 | 1 | *-* | - | TRUE | N_Shore |
| 3429 | cg20548935 | -0.0026 | 0.014219 | 18 | *-* | - | NA | - |
| 3430 | cg24371604 | -0.0042 | 0.014219 | 22 | *MCHR1* | 5'UTR | NA | - |
| 3431 | cg04956949 | -0.0025 | 0.014248 | 7 | *STX1A* | Body | TRUE | S_Shore |
| 3432 | cg19118077 | -0.0068 | 0.014262 | 10 | *AKR1C3* | Body | NA | - |
| 3433 | cg21820873 | 0.0049 | 0.014248 | 11 | *RPS6KA4* | Body | TRUE | S_Shore |
| 3434 | cg19763025 | -0.0058 | 0.014262 | 7 | *LAMB1* | Body | NA | - |
| 3435 | cg00308841 | -0.0053 | 0.014293 | 5 | *-* | - | TRUE | - |
| 3436 | cg05814654 | -0.0017 | 0.01429 | 16 | *IL21R* | TSS1500 | TRUE | - |
| 3437 | cg18484851 | -0.003 | 0.014293 | 12 | *-* | - | NA | - |
| 3438 | cg16382665 | -0.0021 | 0.014311 | 6 | *KCNQ5* | Body | NA | - |
| 3439 | cg19504184 | -0.0022 | 0.014329 | 17 | *-* | - | TRUE | N_Shore |
| 3440 | cg20317748 | -0.004 | 0.014329 | 15 | *C15orf52* | 1stExon | TRUE | - |
| 3441 | cg01260219 | -0.0024 | 0.014334 | 11 | *ADAMTS8* | TSS1500 | TRUE | S_Shore |
| 3442 | cg12426092 | -0.0026 | 0.014355 | 15 | *-* | - | TRUE | Island |
| 3443 | cg13230996 | 0.0032 | 0.014349 | 1 | *-* | - | NA | - |
| 3444 | cg02315732 | -0.0039 | 0.014391 | 11 | *FTH1* | Body | TRUE | N_Shelf |
| 3445 | cg11407226 | -0.0107 | 0.014391 | 7 | *-* | - | NA | - |
| 3446 | cg13175572 | -0.0027 | 0.014386 | 1 | *-* | - | NA | - |
| 3447 | cg17431739 | -0.0033 | 0.014408 | 10 | *MSRB2* | TSS1500 | TRUE | N_Shore |
| 3448 | cg24382070 | 0.0031 | 0.014413 | 8 | *LGI3* | Body | TRUE | N_Shore |
| 3449 | cg15202552 | -0.0026 | 0.014425 | 1 | *FCRL3* | TSS1500 | TRUE | - |
| 3450 | cg22726155 | 0.0021 | 0.014422 | 21 | *-* | - | TRUE | Island |
| 3451 | cg26992381 | -0.0082 | 0.01442 | 3 | *FOXP1* | 5'UTR | NA | - |
| 3452 | cg08607907 | 0.0015 | 0.014435 | 7 | *SGCE* | Body | TRUE | N_Shore |
| 3453 | cg09042411 | 0.0038 | 0.01443 | 7 | *SAP25* | TSS1500 | TRUE | Island |
| 3454 | cg14471330 | 0.0019 | 0.014429 | 9 | *ABL1* | Body | NA | - |
| 3455 | cg14951497 | 0.0035 | 0.014438 | 2 | *STAT1* | 5'UTR | TRUE | N_Shelf |
| 3456 | cg17837888 | 0.0039 | 0.014438 | 15 | *SLCO3A1* | Body | NA | - |
| 3457 | cg00617180 | 0.0028 | 0.014456 | 6 | *-* | - | NA | - |
| 3458 | cg04801602 | -0.0079 | 0.014456 | 9 | *-* | - | NA | - |
| 3459 | cg23647768 | -0.0024 | 0.014456 | 11 | *-* | - | TRUE | N_Shore |
| 3460 | cg22812275 | 0.0034 | 0.014487 | 6 | *EZR* | TSS1500 | TRUE | S_Shore |
| 3461 | cg25556035 | 0.0032 | 0.014497 | 19 | *NFIX* | Body | TRUE | S_Shelf |
| 3462 | cg12259070 | -0.003 | 0.014519 | 1 | *CSMD2* | Body | NA | S_Shore |
| 3463 | cg01435420 | -0.0025 | 0.01453 | 1 | *RAP1GAP* | Body | NA | - |
| 3464 | cg24006770 | 0.0023 | 0.014528 | 1 | *-* | - | TRUE | - |
| 3465 | cg27331006 | -0.0026 | 0.014528 | 1 | *-* | - | NA | - |
| 3466 | cg10752406 | -0.0021 | 0.014549 | 19 | *AZU1* | TSS200 | TRUE | - |
| 3467 | cg26534223 | 0.0022 | 0.014532 | 13 | *DIAPH3AS2* | TSS1500 | NA | - |
| 3468 | cg00002033 | 0.0124 | 0.014583 | 19 | *LRFN1* | Body | TRUE | Island |
| 3469 | cg03464692 | -0.0026 | 0.014583 | 4 | *-* | - | TRUE | S_Shelf |
| 3470 | cg26951018 | -0.0026 | 0.014582 | 7 | *-* | - | NA | - |
| 3471 | cg12569593 | -0.0028 | 0.014592 | 11 | *KIRREL3* | Body | NA | - |
| 3472 | cg23119026 | -0.0033 | 0.014592 | 2 | *-* | - | TRUE | - |
| 3473 | cg26182859 | 0.0035 | 0.014592 | 1 | *-* | - | TRUE | - |
| 3474 | cg13850871 | -0.0022 | 0.0146 | 9 | *-* | - | TRUE | S_Shore |
| 3475 | cg16517790 | -0.0045 | 0.014604 | 19 | *-* | - | NA | S_Shelf |
| 3476 | cg09100984 | 0.0024 | 0.014617 | 11 | *-* | - | NA | - |
| 3477 | cg09143206 | 0.005 | 0.014617 | 5 | *COL23A1* | Body | NA | - |
| 3478 | cg10935968 | -0.0051 | 0.014617 | 18 | *FHOD3* | Body | NA | - |
| 3479 | cg05763299 | -0.0027 | 0.014617 | 2 | *MAL* | Body | NA | - |
| 3480 | cg24939196 | -0.0038 | 0.014619 | 6 | *-* | - | TRUE | - |
| 3481 | cg00809398 | -0.009 | 0.014647 | 11 | *SIK3* | Body | NA | - |
| 3482 | cg24053108 | -0.0044 | 0.014639 | 11 | *CASP1* | Body | NA | - |
| 3483 | cg12371345 | 0.0029 | 0.014663 | 16 | *FBXL16* | 5'UTR | NA | S_Shore |
| 3484 | cg13588791 | -0.0034 | 0.014663 | 1 | *-* | - | NA | - |
| 3485 | cg01515659 | -0.0059 | 0.01467 | 1 | *SYPL2* | Body | NA | - |
| 3486 | cg04313338 | -0.0024 | 0.01467 | 16 | *SLC6A2* | Body | TRUE | - |
| 3487 | cg10592901 | -0.0028 | 0.014671 | 12 | *VDR* | Body | TRUE | - |
| 3488 | cg14283783 | -0.0038 | 0.014674 | 13 | *FLT1* | TSS1500 | TRUE | Island |
| 3489 | cg19905757 | 0.003 | 0.014674 | 15 | *CORO2B* | Body | TRUE | - |
| 3490 | cg25273039 | 0.0012 | 0.014674 | 7 | *NXPH1* | TSS200 | NA | Island |
| 3491 | cg05550391 | 0.0025 | 0.014726 | 10 | *C10orf76* | Body | NA | - |
| 3492 | cg09411739 | -0.0016 | 0.01474 | 11 | *RNH1* | 5'UTR | NA | N_Shore |
| 3493 | cg12637106 | -0.0047 | 0.01474 | 6 | *-* | - | TRUE | Island |
| 3494 | cg25881038 | 0.0039 | 0.014726 | 2 | *PTH2R* | TSS1500 | TRUE | N_Shore |
| 3495 | cg03659428 | 0.005 | 0.01478 | 4 | *SYNPO2* | TSS1500 | NA | - |
| 3496 | cg09179369 | 0.009 | 0.01478 | 22 | *-* | - | NA | - |
| 3497 | cg10775231 | -0.0062 | 0.01478 | 10 | *-* | - | TRUE | S_Shore |
| 3498 | cg11742207 | -0.0018 | 0.01478 | 15 | *NTRK3* | Body | TRUE | - |
| 3499 | cg15393490 | -0.0023 | 0.01478 | 1 | *-* | - | TRUE | - |
| 3500 | cg22730004 | -0.0069 | 0.01478 | 1 | *SPTA1* | TSS1500 | TRUE | - |
| 3501 | cg26768142 | -0.006 | 0.01478 | 2 | *-* | - | NA | - |
| 3502 | cg02293192 | -0.0035 | 0.014809 | 4 | *MMRN1* | Body | NA | - |
| 3503 | cg07242464 | -0.0015 | 0.014835 | 11 | *SLC25A22* | 5'UTR | NA | N_Shore |
| 3504 | cg07944953 | -0.0075 | 0.014821 | 8 | *-* | - | NA | - |
| 3505 | cg05955301 | -0.0023 | 0.014836 | 1 | *PRELP* | TSS1500 | TRUE | - |
| 3506 | cg13500196 | -0.0036 | 0.014873 | 18 | *TCF4* | Body | NA | - |
| 3507 | cg11700071 | 0.0016 | 0.014875 | 11 | *KCNQ1* | Body | NA | - |
| 3508 | cg21814996 | -0.0038 | 0.014875 | 13 | *-* | - | NA | - |
| 3509 | cg24740647 | -0.0034 | 0.014875 | 10 | *-* | - | TRUE | S_Shelf |
| 3510 | cg25024734 | -0.002 | 0.014875 | 10 | *-* | - | TRUE | - |
| 3511 | cg25306915 | -0.0028 | 0.014875 | 8 | *UNC5D* | 3'UTR | NA | - |
| 3512 | cg26976492 | -0.0025 | 0.014881 | 2 | *-* | - | NA | - |
| 3513 | cg03151640 | -0.0053 | 0.014939 | 4 | *CPE* | Body | NA | - |
| 3514 | cg07106169 | 0.003 | 0.014951 | 2 | *-* | - | TRUE | N_Shelf |
| 3515 | cg21632975 | 0.0021 | 0.014951 | 19 | *NOVA2* | Body | TRUE | Island |
| 3516 | cg23475921 | -0.0024 | 0.014967 | X | *MAGED1* | 5'UTR | TRUE | N_Shore |
| 3517 | cg27325224 | -0.0036 | 0.014964 | 16 | *CA5A* | Body | TRUE | N_Shore |
| 3518 | cg12493255 | 0.0036 | 0.014971 | 17 | *ABR* | Body | NA | - |
| 3519 | cg07345722 | -0.0029 | 0.015 | 4 | *-* | - | NA | - |
| 3520 | cg17205832 | -0.0033 | 0.015008 | 8 | *FAM135B* | Body | TRUE | - |
| 3521 | cg04755674 | -0.0043 | 0.015031 | 19 | *IL27RA* | TSS1500 | TRUE | N_Shore |
| 3522 | cg06669752 | -0.0037 | 0.015031 | 5 | *SIL1* | TSS1500 | TRUE | S_Shore |
| 3523 | cg06208270 | -0.0059 | 0.01504 | 1 | *MEX3A* | 3'UTR | TRUE | Island |
| 3524 | cg24125828 | 0.0047 | 0.01504 | 6 | *PRRT1* | Body | TRUE | Island |
| 3525 | cg10274022 | 0.0026 | 0.015068 | 19 | *PLD3* | 5'UTR | TRUE | Island |
| 3526 | cg03285845 | 0.0026 | 0.01507 | 12 | *ETV6* | Body | NA | - |
| 3527 | cg06370069 | -0.0026 | 0.015123 | 10 | *CHST3* | 5'UTR | TRUE | S_Shelf |
| 3528 | cg14833933 | 0.0026 | 0.015113 | 15 | *CAPN3* | 1stExon | TRUE | - |
| 3529 | cg10639411 | -0.0032 | 0.015129 | 3 | *-* | - | TRUE | - |
| 3530 | cg23330270 | 0.0023 | 0.015129 | 12 | *CAC-2D4* | 1stExon | NA | - |
| 3531 | cg03814390 | 9.00E-04 | 0.015143 | 17 | *-* | - | TRUE | Island |
| 3532 | cg06044191 | 0.0026 | 0.015148 | 8 | *PTK2B* | 5'UTR | NA | - |
| 3533 | cg00243364 | -0.0037 | 0.015184 | 8 | *-* | - | NA | - |
| 3534 | cg02490920 | -0.0022 | 0.015184 | 3 | *SEMA3B* | TSS1500 | TRUE | - |
| 3535 | cg11348478 | -0.0044 | 0.015184 | 18 | *RTTN* | Body | NA | - |
| 3536 | cg16389901 | 0.0014 | 0.01522 | 14 | *BMP4* | TSS1500 | TRUE | S_Shore |
| 3537 | cg05752943 | 0.0029 | 0.015234 | 3 | *HTR3D* | TSS1500 | NA | - |
| 3538 | cg02771489 | -0.0026 | 0.015255 | 5 | *-* | - | NA | - |
| 3539 | cg05354317 | -0.0059 | 0.015255 | 14 | *NPAS3* | Body | NA | - |
| 3540 | cg18666327 | -0.0044 | 0.015255 | 17 | *-* | - | NA | - |
| 3541 | cg26469215 | -0.0028 | 0.015255 | 4 | *-* | - | NA | - |
| 3542 | cg16202624 | -0.0027 | 0.015264 | 6 | *AIM1* | Body | TRUE | Island |
| 3543 | cg05618013 | 0.0034 | 0.015273 | 16 | *METTL9* | Body | NA | - |
| 3544 | cg07420027 | -0.0024 | 0.015279 | 3 | *IL12A* | TSS1500 | NA | N_Shore |
| 3545 | cg07828121 | -0.0018 | 0.015279 | 21 | *-* | - | NA | - |
| 3546 | cg23706819 | -0.0051 | 0.015273 | 4 | *-* | - | TRUE | - |
| 3547 | cg12524168 | -0.0025 | 0.015289 | 5 | *F2R* | Body | TRUE | - |
| 3548 | cg16581455 | -0.003 | 0.015281 | 8 | *-* | - | TRUE | - |
| 3549 | cg13582060 | -0.0044 | 0.015292 | 9 | *NEK6* | 5'UTR | TRUE | - |
| 3550 | cg20676788 | -0.0049 | 0.015292 | 13 | *-* | - | TRUE | - |
| 3551 | cg22551065 | -0.0021 | 0.015292 | 5 | *-* | - | TRUE | S_Shore |
| 3552 | cg06624032 | -0.0042 | 0.015309 | 7 | *CPED1* | Body | NA | - |
| 3553 | cg10742523 | -0.0052 | 0.015309 | 4 | *-* | - | TRUE | - |
| 3554 | cg25607383 | 0.0022 | 0.015309 | 6 | *DDR1* | 5'UTR | TRUE | S_Shore |
| 3555 | cg00540067 | 0.0026 | 0.015309 | 3 | *KLF15* | 5'UTR | TRUE | N_Shore |
| 3556 | cg17696044 | -0.0037 | 0.015311 | 11 | *SHANK2* | Body | TRUE | - |
| 3557 | cg17879376 | -0.0036 | 0.015309 | 1 | *BAI2* | Body | TRUE | S_Shelf |
| 3558 | cg18132479 | 0.002 | 0.015309 | 20 | *-* | - | NA | - |
| 3559 | cg25580656 | -0.0025 | 0.015311 | 14 | *ZFYVE21* | Body | TRUE | S_Shelf |
| 3560 | cg06762834 | 0.0039 | 0.015317 | 14 | *SPTB* | Body | NA | - |
| 3561 | cg08747189 | -0.0041 | 0.015326 | 9 | *-* | - | NA | - |
| 3562 | cg11764371 | -0.0027 | 0.015326 | 17 | *-* | - | NA | - |
| 3563 | cg22257107 | -0.003 | 0.015343 | 20 | *KIAA1755* | Body | NA | - |
| 3564 | cg07333314 | 0.0042 | 0.015349 | 19 | *SBNO2* | TSS1500 | NA | - |
| 3565 | cg12902039 | 0.0012 | 0.015358 | 15 | *OCA2* | 1stExon | TRUE | Island |
| 3566 | cg11476866 | 0.0042 | 0.015364 | 17 | *-* | - | NA | - |
| 3567 | cg13107648 | -0.0039 | 0.015372 | 3 | *-* | - | NA | - |
| 3568 | cg16063162 | 0.003 | 0.015383 | 12 | *-* | - | TRUE | - |
| 3569 | cg07446795 | -0.0018 | 0.01539 | 17 | *CCDC40* | Body | TRUE | Island |
| 3570 | cg12901451 | -0.0026 | 0.015392 | X | *GLOD5* | 1stExon | TRUE | - |
| 3571 | cg13490106 | -0.002 | 0.01539 | 8 | *-* | - | NA | - |
| 3572 | cg22796593 | -0.0072 | 0.01539 | 10 | *COL17A1* | Body | TRUE | - |
| 3573 | cg03422015 | 0.0019 | 0.015397 | 12 | *ERC1* | Body | NA | - |
| 3574 | cg04836378 | 0.0031 | 0.015395 | 7 | *NRCAM* | Body | NA | - |
| 3575 | cg06658698 | -0.0055 | 0.015397 | 12 | *EPS8* | Body | NA | - |
| 3576 | cg22836612 | -0.002 | 0.0154 | 1 | *BCAR3* | TSS200 | NA | - |
| 3577 | cg26038465 | 0.0036 | 0.015395 | 22 | *MAFF* | Body | TRUE | Island |
| 3578 | cg25663304 | 0.0027 | 0.0154 | 5 | *-* | - | TRUE | S_Shelf |
| 3579 | cg00829125 | 0.0034 | 0.015429 | 3 | *ATP13A5* | Body | NA | - |
| 3580 | cg18342119 | 0.0057 | 0.015424 | 4 | *SORBS2* | TSS1500 | NA | - |
| 3581 | cg12179661 | -0.0039 | 0.01544 | 9 | *ENTPD8* | 5'UTR | TRUE | N_Shore |
| 3582 | cg20979921 | -0.0035 | 0.01544 | 22 | *C1QTNF6* | Body | TRUE | - |
| 3583 | cg01292810 | -0.004 | 0.015449 | 3 | *-* | - | TRUE | N_Shore |
| 3584 | cg02054191 | 0.0028 | 0.01545 | 1 | *ABL2* | 3'UTR | NA | - |
| 3585 | cg15383054 | 0.0025 | 0.015449 | 17 | *LINC01152* | Body | NA | - |
| 3586 | cg15304674 | -0.0045 | 0.015515 | 7 | *ANKIB1* | 5'UTR | TRUE | - |
| 3587 | cg19757435 | -0.0122 | 0.015508 | 7 | *CALN1* | 1stExon | NA | - |
| 3588 | cg13882251 | -0.0036 | 0.015516 | 19 | *AKT2* | 5'UTR | NA | - |
| 3589 | cg17960517 | -0.0036 | 0.015516 | 18 | *-* | - | NA | - |
| 3590 | cg21807944 | -0.0061 | 0.015516 | 7 | *ARL4A* | 5'UTR | TRUE | S_Shore |
| 3591 | cg23382033 | -0.0052 | 0.015516 | 12 | *SH2B3* | TSS1500 | NA | - |
| 3592 | cg01844491 | -0.0047 | 0.015516 | 7 | *BMPER* | Body | NA | - |
| 3593 | cg04126866 | 0.0026 | 0.015516 | 10 | *C10orf99* | TSS1500 | TRUE | - |
| 3594 | cg10163998 | 0.0029 | 0.015516 | 14 | *-* | - | TRUE | - |
| 3595 | cg12918983 | -0.0038 | 0.015516 | 8 | *-* | - | NA | - |
| 3596 | cg20868633 | -0.0041 | 0.015516 | 9 | *HRCT1* | TSS200 | NA | - |
| 3597 | cg27516404 | -0.0027 | 0.015516 | 7 | *-* | - | TRUE | - |
| 3598 | cg16101346 | -0.0051 | 0.015518 | 1 | *PTGS2* | TSS1500 | TRUE | S_Shore |
| 3599 | cg16163419 | -0.0055 | 0.015518 | 3 | *ABI3BP* | 5'UTR | TRUE | - |
| 3600 | cg26166595 | -0.0032 | 0.015518 | 11 | *DKK3* | Body | TRUE | - |
| 3601 | cg06263344 | 0.0016 | 0.015601 | 11 | *-* | - | NA | - |
| 3602 | cg18096787 | -0.0032 | 0.015596 | 21 | *-* | - | NA | - |
| 3603 | cg23186333 | -0.0064 | 0.015601 | 11 | *CD44* | Body | TRUE | S_Shore |
| 3604 | cg24298026 | -0.0031 | 0.015601 | 6 | *-* | - | TRUE | - |
| 3605 | cg16578742 | -0.0069 | 0.015618 | 5 | *SPEF2* | TSS1500 | TRUE | N_Shore |
| 3606 | cg26499547 | -0.0025 | 0.015634 | 14 | *ACTN1* | Body | NA | - |
| 3607 | cg13622642 | -0.0031 | 0.015651 | 8 | *PPP1R42* | TSS1500 | NA | S_Shore |
| 3608 | cg24391636 | 0.0044 | 0.015639 | 11 | *#NAME?* | Body | NA | - |
| 3609 | cg09867667 | 0.0017 | 0.01568 | 10 | *COMMD3* | Body | TRUE | S_Shore |
| 3610 | cg12532667 | -0.0037 | 0.015689 | 4 | *EMCN* | TSS1500 | TRUE | - |
| 3611 | cg04620466 | -0.0047 | 0.015705 | 7 | *ELN* | TSS200 | NA | - |
| 3612 | cg07121900 | 0.0028 | 0.01571 | 13 | *-* | - | TRUE | N_Shore |
| 3613 | cg14885175 | 0.0028 | 0.015709 | 5 | *SLC23A1* | TSS1500 | NA | - |
| 3614 | cg26731998 | -0.0045 | 0.015705 | 10 | *LOC101927964* | Body | NA | - |
| 3615 | cg12525449 | -0.0037 | 0.015732 | 2 | *ZNF804A* | Body | NA | - |
| 3616 | cg25345365 | -0.0061 | 0.015737 | 11 | *ZBTB16* | Body | NA | - |
| 3617 | cg05677014 | 0.002 | 0.01575 | 14 | *RPS6KL1* | Body | NA | - |
| 3618 | cg07717903 | 0.0044 | 0.01575 | 5 | *PCDHGA4* | Body | TRUE | N_Shore |
| 3619 | cg17261675 | -0.0026 | 0.01575 | 21 | *C21orf29* | Body | TRUE | - |
| 3620 | cg00866476 | 0.003 | 0.015788 | 2 | *-* | - | TRUE | N_Shore |
| 3621 | cg01475805 | 0.0024 | 0.015782 | 12 | *TMEM116* | Body | NA | - |
| 3622 | cg20574434 | -0.0024 | 0.015788 | 12 | *BAZ2A* | TSS1500 | NA | S_Shore |
| 3623 | cg15033085 | -0.0043 | 0.015801 | 3 | *-* | - | NA | - |
| 3624 | cg27208133 | 0.0072 | 0.015791 | 3 | *ZNF385D* | TSS1500 | NA | - |
| 3625 | cg17060366 | 0.0025 | 0.015812 | 16 | *-* | - | NA | - |
| 3626 | cg00570618 | -0.0039 | 0.015824 | 8 | *-* | - | TRUE | - |
| 3627 | cg17436656 | -0.0056 | 0.015843 | 12 | *RARG* | TSS1500 | TRUE | S_Shore |
| 3628 | cg12610312 | -0.0024 | 0.015864 | 12 | *-* | - | TRUE | Island |
| 3629 | cg18501647 | 0.003 | 0.015862 | 6 | *PRRT1* | Body | TRUE | N_Shore |
| 3630 | cg20024850 | 0.0031 | 0.015861 | X | *MCF2* | 5'UTR | TRUE | Island |
| 3631 | cg08349391 | -0.0033 | 0.015901 | 18 | *G-L* | 1stExon | NA | - |
| 3632 | cg16735881 | 0.0021 | 0.015901 | 13 | *NBEA* | Body | TRUE | Island |
| 3633 | cg02683324 | 0.003 | 0.015944 | 11 | *-* | - | NA | - |
| 3634 | cg11177833 | 0.0037 | 0.015928 | 17 | *GLTPD2* | 1stExon | TRUE | Island |
| 3635 | cg03571711 | -0.0026 | 0.015959 | 4 | *NWD2* | Body | NA | - |
| 3636 | cg27656973 | -0.0031 | 0.015956 | 17 | *YBX2* | Body | TRUE | S_Shore |
| 3637 | cg10255761 | -0.0028 | 0.015986 | 3 | *KLHDC8B* | 5'UTR | TRUE | S_Shore |
| 3638 | cg24292594 | 0.0047 | 0.015981 | 19 | *C19orf81* | Body | NA | - |
| 3639 | cg05722944 | -0.003 | 0.015996 | 19 | *NOSIP* | TSS1500 | NA | S_Shore |
| 3640 | cg06738782 | -0.0039 | 0.015996 | 18 | *RBBP8* | Body | NA | - |
| 3641 | cg08255481 | 0.0029 | 0.015996 | 16 | *BANP* | Body | TRUE | S_Shore |
| 3642 | cg14190292 | -0.0019 | 0.016006 | 22 | *-* | - | NA | - |
| 3643 | cg03886752 | -0.0024 | 0.016059 | 16 | *TMEM219* | 5'UTR | NA | S_Shore |
| 3644 | cg00361689 | -0.0023 | 0.016072 | 19 | *CAC-1A* | 1stExon | NA | - |
| 3645 | cg27565185 | -0.0023 | 0.016087 | 3 | *CP* | Body | NA | - |
| 3646 | cg13048896 | 0.0031 | 0.016124 | 16 | *-* | - | NA | - |
| 3647 | cg15864310 | -0.0049 | 0.016144 | 2 | *ABCB11* | Body | NA | - |
| 3648 | cg00690153 | -0.0031 | 0.016158 | 10 | *P4HA1* | 5'UTR | NA | - |
| 3649 | cg01231611 | -0.0023 | 0.016158 | 1 | *REG4* | TSS200 | NA | - |
| 3650 | cg13605327 | -0.0022 | 0.016156 | 10 | *-* | - | TRUE | - |
| 3651 | cg15133043 | 0.0039 | 0.016179 | 17 | *-* | - | NA | - |
| 3652 | cg02957569 | -0.0029 | 0.016179 | 8 | *-* | - | NA | - |
| 3653 | cg05189804 | -0.0058 | 0.016183 | 11 | *ASAM* | Body | TRUE | N_Shore |
| 3654 | cg08709672 | -0.0035 | 0.016183 | 1 | *AVPR1B* | 5'UTR | TRUE | S_Shore |
| 3655 | cg10553154 | 0.0027 | 0.016179 | 10 | *C10orf76* | Body | NA | - |
| 3656 | cg15336210 | -0.0089 | 0.016183 | 2 | *TEX41* | Body | NA | - |
| 3657 | cg27140247 | -0.0042 | 0.016198 | 22 | *C22orf26* | TSS200 | TRUE | Island |
| 3658 | cg00133624 | -0.0028 | 0.016204 | 12 | *EFCAB4B* | Body | TRUE | - |
| 3659 | cg14903837 | -0.0015 | 0.016204 | 1 | *PBX1* | Body | NA | - |
| 3660 | cg26826927 | -0.004 | 0.016204 | 7 | *COG5* | Body | TRUE | - |
| 3661 | cg14953972 | -0.0021 | 0.016216 | 9 | *CYSRT1* | 3'UTR | NA | S_Shelf |
| 3662 | cg24705939 | 0.0022 | 0.016218 | 8 | *RBPMS* | Body | TRUE | - |
| 3663 | cg00235934 | 0.0017 | 0.016258 | 3 | *-* | - | NA | - |
| 3664 | cg17631924 | -0.0033 | 0.016258 | 17 | *-* | - | NA | - |
| 3665 | cg13796381 | -0.004 | 0.016268 | 9 | *PTGDS* | Body | TRUE | Island |
| 3666 | cg14428590 | -0.0053 | 0.01627 | 15 | *FSIP1* | TSS1500 | TRUE | S_Shore |
| 3667 | cg06529600 | -0.0052 | 0.016271 | 6 | *-* | - | TRUE | - |
| 3668 | cg06973778 | -0.0057 | 0.016299 | 9 | *SMARCA2* | TSS1500 | NA | - |
| 3669 | cg11204177 | -0.0051 | 0.016294 | 7 | *-* | - | NA | - |
| 3670 | cg19630629 | 0.0029 | 0.016322 | 7 | *CHRM2* | 5'UTR | TRUE | Island |
| 3671 | cg02193584 | -0.0027 | 0.016344 | 2 | *DPYSL5* | Body | NA | - |
| 3672 | cg06135765 | -0.0034 | 0.016344 | 3 | *GOLGA4* | TSS1500 | TRUE | N_Shore |
| 3673 | cg07347026 | -0.003 | 0.016344 | 20 | *-* | - | NA | - |
| 3674 | cg07910181 | -0.002 | 0.016344 | 1 | *C1orf123* | Body | NA | N_Shore |
| 3675 | cg13572071 | 0.0033 | 0.016352 | 1 | *TCEA3* | 3'UTR | TRUE | - |
| 3676 | cg16551670 | -0.0033 | 0.016349 | 5 | *-* | - | TRUE | - |
| 3677 | cg05178839 | -0.0026 | 0.016373 | 16 | *JPH3* | Body | TRUE | N_Shore |
| 3678 | cg06754224 | -0.0018 | 0.016373 | 1 | *SESN2* | TSS1500 | TRUE | N_Shore |
| 3679 | cg10784813 | 0.004 | 0.0164 | 16 | *SOCS1* | 3'UTR | TRUE | Island |
| 3680 | cg00350885 | -0.0012 | 0.016402 | 7 | *GRB10* | 5'UTR | TRUE | N_Shore |
| 3681 | cg02199947 | -0.0027 | 0.016402 | 3 | *-* | - | NA | - |
| 3682 | cg13854896 | -0.0022 | 0.016402 | 1 | *-* | - | NA | - |
| 3683 | cg25697538 | 0.0025 | 0.016402 | 2 | *-* | - | NA | - |
| 3684 | cg26050864 | 0.0024 | 0.016427 | 1 | *G0S2* | 3'UTR | TRUE | S_Shore |
| 3685 | cg01781529 | -0.0023 | 0.016431 | 1 | *WNT4* | 3'UTR | TRUE | N_Shelf |
| 3686 | cg16550106 | -0.0022 | 0.016431 | 3 | *-* | - | NA | - |
| 3687 | cg22975024 | -0.0046 | 0.016431 | 6 | *-* | - | NA | - |
| 3688 | cg27035169 | -0.0036 | 0.016431 | 14 | *SLC7A8* | TSS1500 | TRUE | - |
| 3689 | cg04537723 | -0.0039 | 0.016438 | 20 | *-* | - | NA | - |
| 3690 | cg13251913 | 0.0032 | 0.01644 | 16 | *ADGRG1* | 1stExon | NA | - |
| 3691 | cg15262253 | -0.0024 | 0.016471 | 10 | *FUT11* | TSS1500 | NA | N_Shore |
| 3692 | cg21481630 | -0.0024 | 0.01648 | 2 | *EXOC6B* | Body | NA | - |
| 3693 | cg12964697 | 0.0023 | 0.016507 | 7 | *MAD1L1* | Body | TRUE | - |
| 3694 | cg13269407 | -0.0065 | 0.016507 | 22 | *C22orf26* | TSS200 | TRUE | Island |
| 3695 | cg14430760 | -0.0021 | 0.016508 | 17 | *NXN* | TSS1500 | NA | - |
| 3696 | cg02734692 | -0.0039 | 0.016524 | 9 | *RGS3* | Body | NA | - |
| 3697 | cg03545227 | 0.0011 | 0.016516 | 2 | *PTPRN* | Body | TRUE | Island |
| 3698 | cg15421962 | -0.0032 | 0.016524 | 5 | *BNIP1* | TSS1500 | TRUE | - |
| 3699 | cg18747571 | 0.0042 | 0.016516 | 18 | *-* | - | NA | - |
| 3700 | cg14592376 | -0.004 | 0.016535 | 3 | *-* | - | TRUE | S_Shore |
| 3701 | cg24330553 | 0.0028 | 0.016537 | 13 | *-* | - | NA | - |
| 3702 | cg15775152 | 0.0016 | 0.016538 | 8 | *CCDC25* | Body | NA | - |
| 3703 | cg15826568 | -0.0042 | 0.016537 | 16 | *BCO1* | TSS200 | NA | - |
| 3704 | cg17969341 | -0.0024 | 0.016553 | 7 | *MICALL2* | TSS1500 | NA | S_Shore |
| 3705 | cg20789819 | -0.0031 | 0.016553 | 7 | *-* | - | NA | - |
| 3706 | cg21029505 | -0.0042 | 0.016553 | 16 | *CDH13* | Body | NA | - |
| 3707 | cg22380090 | -0.0048 | 0.016553 | 10 | *SLC16A9* | Body | NA | - |
| 3708 | cg05539750 | 0.0021 | 0.01657 | 16 | *CDH13* | 5'UTR | NA | - |
| 3709 | cg21768956 | -8.00E-04 | 0.016599 | 17 | *ABR* | Body | TRUE | S_Shore |
| 3710 | cg24084564 | 0.0029 | 0.016599 | 19 | *-* | - | TRUE | N_Shore |
| 3711 | cg15284208 | 0.0017 | 0.016611 | 2 | *RNF144A* | 5'UTR | NA | - |
| 3712 | cg17150365 | -0.0024 | 0.016611 | 8 | *SAMD12AS1* | Body | NA | N_Shore |
| 3713 | cg00094538 | 0.0033 | 0.016671 | 7 | *-* | - | NA | - |
| 3714 | cg24317086 | 0.0023 | 0.016665 | 15 | *-* | - | TRUE | N_Shelf |
| 3715 | cg26598777 | -0.0031 | 0.016671 | 14 | *-* | - | NA | - |
| 3716 | cg00436253 | -0.0044 | 0.016673 | 17 | *LRRC75AAS1* | Body | NA | - |
| 3717 | cg10503136 | -0.0026 | 0.016673 | 1 | *PCP4L1* | Body | NA | S_Shore |
| 3718 | cg15681261 | 0.0015 | 0.016671 | 1 | *WNT3A* | Body | NA | - |
| 3719 | cg19722847 | -0.0033 | 0.016671 | 12 | *IPO8* | TSS1500 | TRUE | S_Shore |
| 3720 | cg08073979 | -0.0019 | 0.016677 | X | *-* | - | NA | - |
| 3721 | cg14392283 | 0.0033 | 0.016677 | 8 | *LY6E* | 3'UTR | TRUE | N_Shelf |
| 3722 | cg14338345 | -0.0038 | 0.01672 | 9 | *DMRTA1* | 1stExon | TRUE | Island |
| 3723 | cg15851014 | -0.0074 | 0.01672 | 4 | *TLR3* | TSS1500 | NA | - |
| 3724 | cg21163717 | -0.003 | 0.01672 | 8 | *DOK2* | Body | TRUE | Island |
| 3725 | cg26795073 | 0.0018 | 0.016727 | 15 | *-* | - | NA | - |
| 3726 | cg10446433 | 0.0023 | 0.016736 | 2 | *BRE* | Body | TRUE | - |
| 3727 | cg26246101 | -0.0028 | 0.016746 | 3 | *MYRIP* | TSS200 | NA | - |
| 3728 | cg24807169 | -0.0031 | 0.01681 | 4 | *EMCN* | TSS1500 | TRUE | - |
| 3729 | cg03455534 | 0.0018 | 0.016812 | 1 | *-* | - | NA | - |
| 3730 | cg12606401 | 0.0023 | 0.016815 | 2 | *-* | - | NA | - |
| 3731 | cg15708154 | 0.0024 | 0.016812 | 1 | *-* | - | NA | - |
| 3732 | cg19092476 | 0.0024 | 0.016846 | 4 | *-* | - | NA | - |
| 3733 | cg24052470 | -0.0041 | 0.016845 | 2 | *-* | - | NA | - |
| 3734 | cg00881945 | 0.0019 | 0.016856 | 9 | *TTLL11* | Body | NA | - |
| 3735 | cg02229135 | 0.0025 | 0.016856 | 13 | *MIR548F5* | Body | TRUE | Island |
| 3736 | cg22254983 | 0.0025 | 0.016853 | 8 | *-* | - | TRUE | - |
| 3737 | cg01329519 | -0.0088 | 0.016861 | 5 | *-* | - | NA | - |
| 3738 | cg13049862 | -0.0026 | 0.016864 | 17 | *MYO1C* | Body | TRUE | S_Shelf |
| 3739 | cg01327347 | 0.0034 | 0.016894 | 9 | *CDK20* | Body | NA | N_Shore |
| 3740 | cg09118625 | 0.0036 | 0.016894 | 1 | *DIRAS3* | Body | TRUE | Island |
| 3741 | cg02699218 | 0.0028 | 0.01692 | 5 | *ANKRD43* | 1stExon | TRUE | Island |
| 3742 | cg26577523 | -0.0015 | 0.016918 | 2 | *PCBP1AS1* | Body | NA | - |
| 3743 | cg18369516 | 0.0091 | 0.01692 | 20 | *ZBTB46* | Body | TRUE | Island |
| 3744 | cg05615722 | -0.0024 | 0.016943 | 15 | *-* | - | NA | - |
| 3745 | cg06752595 | -0.0089 | 0.01694 | 6 | *ALDH5A1* | TSS1500 | NA | N_Shore |
| 3746 | cg03234980 | -0.0019 | 0.016963 | 5 | *FABP6* | 5'UTR | TRUE | - |
| 3747 | cg25195136 | 0.0035 | 0.016972 | 3 | *-* | - | NA | - |
| 3748 | cg25998745 | -0.0048 | 0.016986 | 8 | *-* | - | TRUE | - |
| 3749 | cg08289567 | -0.0039 | 0.017016 | 4 | *-* | - | TRUE | - |
| 3750 | cg03165378 | 0.0023 | 0.017021 | 1 | *S100A9* | TSS1500 | TRUE | - |
| 3751 | cg11294276 | -0.0033 | 0.017021 | 15 | *MYO1E* | Body | NA | - |
| 3752 | cg15424740 | -0.0031 | 0.017036 | 13 | *LRCH1* | 3'UTR | NA | - |
| 3753 | cg17398329 | -0.0027 | 0.017036 | 11 | *NFRKB* | Body | NA | - |
| 3754 | cg11761728 | 0.0016 | 0.017048 | 4 | *-* | - | TRUE | N_Shelf |
| 3755 | cg14349763 | -0.0026 | 0.017053 | 9 | *ADAMTS13* | Body | TRUE | S_Shelf |
| 3756 | cg20506944 | 0.0029 | 0.017053 | 1 | *D-JC6* | 5'UTR | NA | S_Shore |
| 3757 | cg00913442 | -0.0032 | 0.017077 | X | *GRIA3* | Body | NA | - |
| 3758 | cg03834767 | -0.0034 | 0.017076 | 7 | *CDK14* | 3'UTR | TRUE | - |
| 3759 | cg06708213 | -0.0031 | 0.017076 | 17 | *MGAT5B* | TSS1500 | TRUE | N_Shore |
| 3760 | cg12530994 | -0.0052 | 0.017077 | 10 | *AKR1C3* | Body | TRUE | - |
| 3761 | cg05723989 | 0.0027 | 0.017077 | 1 | *C1orf133* | TSS1500 | TRUE | S_Shore |
| 3762 | cg27263505 | -0.0043 | 0.017077 | 18 | *-* | - | NA | - |
| 3763 | cg09986153 | -0.0036 | 0.017115 | 8 | *HMBOX1* | Body | NA | - |
| 3764 | cg19789466 | -0.0025 | 0.017113 | 12 | *OAS1* | 1stExon | TRUE | - |
| 3765 | cg03635266 | -0.0033 | 0.017128 | 8 | *TRPS1* | 3'UTR | NA | - |
| 3766 | cg00224335 | -0.0023 | 0.017165 | 21 | *MIR4327* | TSS200 | NA | - |
| 3767 | cg04330849 | -0.0034 | 0.017181 | 3 | *LINC01014* | Body | NA | - |
| 3768 | cg00462271 | 0.0023 | 0.017203 | 2 | *SPRED2* | Body | NA | - |
| 3769 | cg21994794 | -0.002 | 0.017209 | 14 | *NDRG2* | 5'UTR | NA | N_Shore |
| 3770 | cg22156674 | -0.0024 | 0.017209 | 2 | *-* | - | TRUE | - |
| 3771 | cg17394230 | -0.0017 | 0.017218 | 6 | *PTCRA* | Body | NA | Island |
| 3772 | cg00499595 | -0.0029 | 0.017229 | 8 | *-* | - | NA | - |
| 3773 | cg09124532 | -0.004 | 0.017229 | 4 | *-* | - | NA | - |
| 3774 | cg11208719 | -0.0022 | 0.017228 | 12 | *-* | - | NA | - |
| 3775 | cg23254295 | -0.0027 | 0.017228 | 17 | *ABCC3* | Body | NA | - |
| 3776 | cg01462126 | -0.0038 | 0.017239 | 8 | *CCDC25* | Body | NA | N_Shore |
| 3777 | cg25330665 | 0.0024 | 0.017239 | 7 | *AUTS2* | Body | NA | - |
| 3778 | cg20473680 | 0.0022 | 0.017246 | 19 | *SMARCA4* | 5'UTR | NA | - |
| 3779 | cg19313311 | -0.0062 | 0.017285 | 7 | *-* | - | TRUE | - |
| 3780 | cg11633892 | -0.0028 | 0.017352 | 3 | *-* | - | NA | S_Shelf |
| 3781 | cg16260349 | 0.0033 | 0.017352 | 10 | *TSPAN14* | 5'UTR | TRUE | - |
| 3782 | cg18819262 | -0.0024 | 0.017352 | 4 | *-* | - | NA | - |
| 3783 | cg23387597 | 0.0035 | 0.017352 | 10 | *ITPRIP* | TSS200 | TRUE | N_Shelf |
| 3784 | cg13518852 | -0.0039 | 0.017366 | 1 | *-* | - | NA | - |
| 3785 | cg18960565 | 0.0017 | 0.017366 | 17 | *#NAME?* | TSS1500 | NA | N_Shore |
| 3786 | cg21762610 | -0.0066 | 0.017366 | 2 | *UNC80* | Body | NA | - |
| 3787 | cg07485518 | -0.005 | 0.017372 | 14 | *-* | - | NA | - |
| 3788 | cg13457345 | 0.004 | 0.017372 | 3 | *PCOLCE2* | TSS1500 | NA | S_Shore |
| 3789 | cg17078883 | -0.0021 | 0.017372 | 10 | *-* | - | TRUE | - |
| 3790 | cg21284015 | 0.0019 | 0.017372 | 17 | *BAHCC1* | Body | TRUE | Island |
| 3791 | cg24271593 | -0.0037 | 0.017375 | 1 | *-* | - | NA | - |
| 3792 | cg07560588 | -0.0026 | 0.017399 | 1 | *SERBP1* | Body | NA | - |
| 3793 | cg26944958 | -0.0065 | 0.017409 | 18 | *-* | - | NA | - |
| 3794 | cg03688488 | -0.0015 | 0.017439 | 19 | *-* | - | NA | - |
| 3795 | cg24629020 | -0.006 | 0.017439 | 12 | *-* | - | NA | - |
| 3796 | cg02387435 | -0.0042 | 0.017442 | 22 | *-* | - | TRUE | N_Shore |
| 3797 | cg05941376 | -0.0044 | 0.017442 | 5 | *WWC1* | Body | TRUE | - |
| 3798 | cg14467281 | 0.0023 | 0.017439 | 9 | *SMU1* | 3'UTR | TRUE | Island |
| 3799 | cg23573505 | -0.0042 | 0.017442 | 13 | *EFNB2* | Body | NA | - |
| 3800 | cg02920129 | 0.0109 | 0.017447 | 3 | *ZNF385D* | Body | NA | - |
| 3801 | cg18464726 | -0.0023 | 0.017447 | 12 | *-* | - | TRUE | N_Shelf |
| 3802 | cg04391133 | -0.0026 | 0.017453 | 1 | *-* | - | NA | - |
| 3803 | cg20726664 | -0.0058 | 0.017454 | 15 | *AKAP13* | Body | TRUE | - |
| 3804 | cg03640151 | 0.0037 | 0.017473 | 4 | *-* | - | TRUE | - |
| 3805 | cg20592714 | 0.0023 | 0.017475 | 3 | *-* | - | NA | - |
| 3806 | cg25920842 | -0.0047 | 0.017473 | 7 | *-* | - | NA | - |
| 3807 | cg21488876 | -0.0025 | 0.017513 | 19 | *PTPRS* | Body | TRUE | N_Shore |
| 3808 | cg09332353 | 0.003 | 0.017516 | 6 | *PRSS16* | TSS200 | TRUE | N_Shelf |
| 3809 | cg07837181 | -0.0023 | 0.017531 | 3 | *FXR1* | TSS1500 | NA | N_Shore |
| 3810 | cg14426911 | -0.0066 | 0.01753 | 5 | *SEMA5A* | Body | NA | - |
| 3811 | cg16126997 | -0.0039 | 0.01753 | 2 | *-* | - | NA | - |
| 3812 | cg03022317 | 0.0036 | 0.017578 | 9 | *RCL1* | Body | NA | - |
| 3813 | cg16312609 | 0.0031 | 0.017583 | 14 | *-* | - | TRUE | - |
| 3814 | cg03065175 | 0.0044 | 0.017588 | 4 | *-* | - | TRUE | Island |
| 3815 | cg12558059 | 0.0027 | 0.017588 | 10 | *LZTS2* | TSS1500 | NA | N_Shore |
| 3816 | cg13381089 | -0.0027 | 0.017588 | 10 | *AIFM2* | TSS1500 | NA | S_Shore |
| 3817 | cg02256455 | 0.004 | 0.017608 | 14 | *XRCC3* | Body | TRUE | S_Shore |
| 3818 | cg11264678 | 0.0028 | 0.017608 | 17 | *STAT3* | 3'UTR | NA | S_Shore |
| 3819 | cg12572059 | 0.0024 | 0.01766 | 10 | *-* | - | NA | - |
| 3820 | cg23821328 | -0.0023 | 0.01766 | 10 | *-* | - | NA | - |
| 3821 | cg06222012 | -0.0036 | 0.017676 | 2 | *-* | - | TRUE | - |
| 3822 | cg12373771 | 0.0021 | 0.017683 | 22 | *CECR6* | 1stExon | TRUE | Island |
| 3823 | cg14778169 | 0.0031 | 0.017681 | 7 | *CAC-2D1* | TSS1500 | TRUE | S_Shore |
| 3824 | cg01845277 | -0.0031 | 0.017706 | 8 | *ZNF704* | 3'UTR | NA | - |
| 3825 | cg06499565 | 0.0011 | 0.017706 | 20 | *NDRG3* | TSS1500 | TRUE | S_Shore |
| 3826 | cg16334524 | -0.0044 | 0.017715 | 21 | *C21orf84* | TSS200 | TRUE | - |
| 3827 | cg11362599 | -0.0032 | 0.01777 | 21 | *-* | - | NA | - |
| 3828 | cg14287557 | -0.0016 | 0.017771 | 15 | *C15orf52* | TSS200 | NA | - |
| 3829 | cg26754818 | -0.0022 | 0.017771 | 7 | *-* | - | NA | - |
| 3830 | cg06234378 | -0.004 | 0.017771 | 17 | *CARD14* | Body | TRUE | N_Shore |
| 3831 | cg23302438 | -0.0034 | 0.017779 | 1 | *MTX1* | TSS1500 | NA | N_Shore |
| 3832 | cg02269157 | -0.004 | 0.017783 | 12 | *ANKRD52* | TSS1500 | NA | S_Shore |
| 3833 | cg04730443 | -0.0033 | 0.017787 | X | *MAGEB6* | TSS200 | TRUE | - |
| 3834 | cg07389870 | 0.0028 | 0.0178 | 1 | *SLC16A4* | Body | TRUE | - |
| 3835 | cg12729838 | -0.0033 | 0.017827 | 8 | *CLU* | 1stExon | TRUE | N_Shelf |
| 3836 | cg14363563 | -0.0043 | 0.01785 | 2 | *SULT1C2P1* | Body | NA | - |
| 3837 | cg16499416 | 0.0023 | 0.017904 | 11 | *MACROD1* | 3'UTR | TRUE | Island |
| 3838 | cg19992167 | -0.0019 | 0.017907 | 8 | *LINC00534* | Body | NA | - |
| 3839 | cg06713813 | -0.0076 | 0.01792 | 1 | *WARS2* | Body | NA | - |
| 3840 | cg07241717 | 0.0018 | 0.01792 | 22 | *PACSIN2* | 5'UTR | NA | N_Shore |
| 3841 | cg14243481 | 0.002 | 0.01792 | 11 | *SYT9* | TSS1500 | TRUE | Island |
| 3842 | cg24119006 | 0.0027 | 0.01792 | 2 | *MIR4269* | TSS1500 | NA | - |
| 3843 | cg06804705 | -0.0035 | 0.017928 | 21 | *NCR-00114* | TSS1500 | TRUE | - |
| 3844 | cg18053607 | -0.0027 | 0.017923 | 22 | *INPP5J* | 5'UTR | TRUE | - |
| 3845 | cg07976733 | -0.0043 | 0.017936 | 17 | *-* | - | NA | - |
| 3846 | cg12807158 | 0.0023 | 0.017936 | 3 | *LINC01215* | Body | NA | - |
| 3847 | cg07255019 | -0.0019 | 0.018019 | 22 | *TPST2* | 5'UTR | TRUE | N_Shore |
| 3848 | cg02191320 | 0.0063 | 0.018026 | 2 | *EML6* | Body | NA | - |
| 3849 | cg09485579 | -0.0036 | 0.018026 | 8 | *LOC101927543* | Body | NA | - |
| 3850 | cg18464316 | -0.0028 | 0.018026 | 3 | *CP* | Body | NA | - |
| 3851 | cg22539632 | 0.0051 | 0.018027 | 4 | *TACR3* | Body | NA | - |
| 3852 | cg06044636 | 0.0029 | 0.018037 | 22 | *MICAL3* | Body | NA | - |
| 3853 | cg11495719 | -0.0047 | 0.018038 | 11 | *-* | - | NA | - |
| 3854 | cg22189991 | 0.0026 | 0.01803 | 16 | *ST3GAL2* | 5'UTR | NA | - |
| 3855 | cg13511777 | -0.0046 | 0.018046 | 11 | *0* | Body | TRUE | - |
| 3856 | cg12307069 | -0.0021 | 0.018057 | 11 | *SMCO4* | 5'UTR | NA | - |
| 3857 | cg15239695 | 0.0028 | 0.018057 | 11 | *ETS1* | Body | NA | - |
| 3858 | cg21418707 | -0.0029 | 0.018057 | 1 | *TP73* | Body | TRUE | S_Shore |
| 3859 | cg22143698 | 0.0019 | 0.018057 | 5 | *ANKRD33B* | Body | TRUE | - |
| 3860 | cg00791074 | -0.0043 | 0.018061 | 6 | *MTHFD1L* | TSS1500 | TRUE | N_Shore |
| 3861 | cg21082116 | -0.0061 | 0.018065 | 4 | *STPG2* | Body | NA | - |
| 3862 | cg09576358 | -0.0019 | 0.018068 | 16 | *ALDOA* | 1stExon | NA | S_Shore |
| 3863 | cg11975824 | -0.0028 | 0.018068 | 8 | *-* | - | NA | - |
| 3864 | cg21599943 | -0.0038 | 0.018096 | 17 | *-* | - | NA | - |
| 3865 | cg16083670 | -0.0028 | 0.018103 | 17 | *TMEM92* | TSS1500 | NA | N_Shelf |
| 3866 | cg21990786 | -0.003 | 0.018103 | 17 | *RABEP1* | Body | NA | - |
| 3867 | cg25197194 | 0.0045 | 0.018099 | 3 | *CCDC48* | 3'UTR | TRUE | - |
| 3868 | cg08189223 | -0.0047 | 0.018107 | 7 | *NOBOX* | TSS200 | NA | - |
| 3869 | cg21958074 | -0.0042 | 0.018107 | 2 | *-* | - | NA | - |
| 3870 | cg26524555 | 0.0031 | 0.018107 | 14 | *M-T1* | Body | NA | - |
| 3871 | cg05625314 | -0.0025 | 0.018129 | 4 | *-* | - | NA | - |
| 3872 | cg06877191 | -0.0033 | 0.018128 | 8 | *-* | - | NA | - |
| 3873 | cg17852032 | -0.005 | 0.018128 | 6 | *ZDHHC14* | Body | TRUE | - |
| 3874 | cg21676859 | -0.0109 | 0.018128 | 13 | *STK24* | Body | NA | - |
| 3875 | cg11785812 | 0.0019 | 0.018168 | 2 | *RAB11FIP5* | Body | NA | - |
| 3876 | cg21213075 | -0.0029 | 0.018161 | 10 | *-* | - | NA | - |
| 3877 | cg01892452 | -0.0037 | 0.018177 | 1 | *COL24A1* | Body | NA | - |
| 3878 | cg21145608 | 0.0057 | 0.018177 | 6 | *PLEKHG1* | 5'UTR | NA | - |
| 3879 | cg07606495 | -0.0022 | 0.018179 | 19 | *C19orf57* | 3'UTR | TRUE | S_Shelf |
| 3880 | cg25758314 | 0.0079 | 0.01818 | X | *RENBP* | Body | TRUE | S_Shore |
| 3881 | cg06235649 | 0.0022 | 0.01819 | 22 | *PISD* | 5'UTR | NA | - |
| 3882 | cg19109335 | -0.0067 | 0.018188 | 6 | *RUNX2* | TSS1500 | NA | - |
| 3883 | cg02903680 | -0.0051 | 0.018232 | 15 | *PDE8A* | Body | TRUE | S_Shore |
| 3884 | cg06859186 | -0.0035 | 0.01823 | 17 | *-* | - | NA | - |
| 3885 | cg08551218 | 0.0018 | 0.01823 | 6 | *LRRC73* | 5'UTR | NA | Island |
| 3886 | cg27017172 | -0.0012 | 0.018239 | 16 | *POLR2C* | Body | TRUE | S_Shore |
| 3887 | cg27427318 | 0.0053 | 0.018239 | 1 | *-* | - | TRUE | S_Shore |
| 3888 | cg02268417 | -0.0038 | 0.018241 | 1 | *-* | - | NA | - |
| 3889 | cg18028993 | -0.0053 | 0.018241 | 10 | *-* | - | NA | - |
| 3890 | cg21528155 | -0.0046 | 0.018241 | 1 | *NFIA* | Body | NA | - |
| 3891 | cg04185922 | -0.0051 | 0.01825 | 6 | *LOC285768* | Body | NA | - |
| 3892 | cg25757472 | 0.002 | 0.01825 | 7 | *CDK14* | TSS1500 | NA | N_Shore |
| 3893 | cg20966659 | -0.0023 | 0.018262 | 3 | *-* | - | TRUE | - |
| 3894 | cg03229590 | -0.0055 | 0.018279 | 7 | *CALN1* | 5'UTR | NA | - |
| 3895 | cg02669012 | -0.0041 | 0.018299 | 10 | *-* | - | NA | - |
| 3896 | cg08622043 | 0.0026 | 0.018294 | 19 | *CCDC155* | Body | NA | - |
| 3897 | cg15321693 | -0.0026 | 0.018324 | 22 | *SSTR3* | TSS1500 | NA | - |
| 3898 | cg17514495 | 0.0031 | 0.018323 | 1 | *RXRG* | TSS1500 | TRUE | - |
| 3899 | cg02024465 | -0.0027 | 0.018361 | 14 | *-* | - | NA | - |
| 3900 | cg11655407 | 0.003 | 0.018361 | 18 | *BCL2* | Body | NA | - |
| 3901 | cg08669249 | -0.0047 | 0.018374 | 4 | *PALLD* | Body | NA | - |
| 3902 | cg05501609 | -0.0027 | 0.018391 | 14 | *HEATR5A* | TSS1500 | NA | S_Shore |
| 3903 | cg05245284 | -0.0077 | 0.01843 | 11 | *-* | - | NA | - |
| 3904 | cg16899999 | -0.0055 | 0.018434 | 10 | *-* | - | NA | - |
| 3905 | cg17333291 | 0.0027 | 0.018477 | 7 | *GPER* | TSS200 | TRUE | N_Shore |
| 3906 | cg21109217 | -0.0021 | 0.018475 | 17 | *KCNH6* | 5'UTR | NA | - |
| 3907 | cg23494015 | -8.00E-04 | 0.018475 | 14 | *C14orf38* | TSS200 | TRUE | S_Shore |
| 3908 | cg24706980 | -0.0037 | 0.018477 | 1 | *RGL1* | 5'UTR | NA | - |
| 3909 | cg17837572 | -0.0094 | 0.018479 | 7 | *SDK1* | Body | TRUE | - |
| 3910 | cg16593845 | -0.0016 | 0.018504 | 22 | *-* | - | NA | - |
| 3911 | cg23772533 | 0.0027 | 0.01855 | 16 | *RPL13* | Body | NA | S_Shore |
| 3912 | cg00178249 | 0.0015 | 0.018565 | 8 | *LOC100192378* | Body | TRUE | N_Shore |
| 3913 | cg04648494 | 0.0022 | 0.018565 | 2 | *-* | - | TRUE | N_Shore |
| 3914 | cg24481303 | -0.004 | 0.018565 | 7 | *PTPRN2* | Body | TRUE | - |
| 3915 | cg17754500 | -0.0096 | 0.018602 | 3 | *-* | - | NA | - |
| 3916 | cg07991586 | -0.0024 | 0.018616 | 16 | *-* | - | TRUE | N_Shore |
| 3917 | cg22574675 | -0.0029 | 0.018677 | 10 | *-* | - | TRUE | N_Shore |
| 3918 | cg05622422 | 0.003 | 0.018692 | 17 | *FAM171A2* | Body | TRUE | Island |
| 3919 | cg07452347 | 0.0033 | 0.018694 | 3 | *CLCN2* | 1stExon | NA | - |
| 3920 | cg04618184 | -0.0048 | 0.018696 | 14 | *-* | - | NA | - |
| 3921 | cg07952254 | -0.0031 | 0.018698 | 21 | *-* | - | NA | - |
| 3922 | cg23691090 | -0.0079 | 0.0187 | 22 | *C22orf26* | 1stExon | TRUE | Island |
| 3923 | cg08121845 | -0.011 | 0.018706 | 3 | *-* | - | NA | - |
| 3924 | cg18685080 | 0.0025 | 0.018706 | 16 | *CMIP* | Body | NA | - |
| 3925 | cg21002674 | -0.0025 | 0.01871 | 4 | *CLNK* | Body | NA | - |
| 3926 | cg08532140 | 0.0033 | 0.018711 | 6 | *FAM26F* | TSS1500 | NA | N_Shore |
| 3927 | cg12801115 | -0.0036 | 0.018711 | 4 | *-* | - | NA | - |
| 3928 | cg16324745 | -0.004 | 0.018719 | 7 | *AUTS2* | Body | NA | - |
| 3929 | cg25944317 | -0.0042 | 0.018719 | 6 | *SUPT3H* | 5'UTR | NA | - |
| 3930 | cg27210390 | -0.0035 | 0.018722 | 17 | *TOM1L1* | Body | TRUE | S_Shore |
| 3931 | cg19833748 | -0.0022 | 0.018744 | 7 | *C7orf31* | TSS1500 | NA | S_Shore |
| 3932 | cg04657044 | -0.0028 | 0.018754 | 13 | *ITM2B* | TSS1500 | TRUE | N_Shore |
| 3933 | cg03067296 | 0.0054 | 0.018769 | 17 | *LOC100996291* | TSS200 | NA | - |
| 3934 | cg26470457 | 0.0023 | 0.018772 | 10 | *ZMIZ1* | Body | NA | - |
| 3935 | cg04837005 | -0.0016 | 0.018808 | 6 | *SYNE1* | Body | NA | - |
| 3936 | cg22277154 | -0.0096 | 0.018808 | 13 | *-* | - | TRUE | S_Shore |
| 3937 | cg27529143 | 0.0018 | 0.018867 | 12 | *PITPNM2* | 5'UTR | NA | - |
| 3938 | cg26122129 | -0.0031 | 0.01888 | 8 | *RBPMS* | TSS1500 | TRUE | N_Shore |
| 3939 | cg26754179 | 0.0023 | 0.01889 | 7 | *LOC349160* | Body | NA | - |
| 3940 | cg15379564 | -0.0019 | 0.01889 | 1 | *-* | - | NA | - |
| 3941 | cg20959829 | -0.0021 | 0.018901 | 5 | *EBF1* | Body | NA | - |
| 3942 | cg03556995 | -0.0027 | 0.018901 | 4 | *-* | - | NA | - |
| 3943 | cg26636279 | -0.0054 | 0.018907 | 3 | *CD200R1* | Body | NA | - |
| 3944 | cg10591467 | -0.002 | 0.018914 | 17 | *ALOX12B* | Body | NA | N_Shelf |
| 3945 | cg11004181 | -0.0021 | 0.018923 | 17 | *CCDC40* | Body | NA | N_Shore |
| 3946 | cg11051758 | 0.002 | 0.018923 | 14 | *FERMT2* | Body | NA | - |
| 3947 | cg19687957 | 0.0022 | 0.018923 | 1 | *KIF26B* | Body | NA | - |
| 3948 | cg16681914 | 0.0018 | 0.018948 | X | *MSL3* | Body | TRUE | S_Shore |
| 3949 | cg08867923 | -0.0041 | 0.01895 | 7 | *-* | - | NA | - |
| 3950 | cg20471413 | 0.0052 | 0.01895 | 6 | *TNXB* | Body | TRUE | Island |
| 3951 | cg21259978 | -0.0045 | 0.01895 | 4 | *-* | - | NA | - |
| 3952 | cg13246175 | -0.0048 | 0.018983 | 13 | *NUFIP1* | Body | NA | - |
| 3953 | cg21859562 | 0.0043 | 0.018989 | 1 | *-* | - | TRUE | - |
| 3954 | cg05883098 | -0.002 | 0.018995 | 1 | *-* | - | NA | S_Shelf |
| 3955 | cg14419781 | -0.0022 | 0.018995 | 6 | *SLC44A4* | Body | NA | - |
| 3956 | cg07070138 | -0.0023 | 0.01901 | 11 | *MUC6* | Body | TRUE | S_Shore |
| 3957 | cg04563718 | -0.0022 | 0.019018 | 1 | *MMEL1* | TSS1500 | TRUE | - |
| 3958 | cg07292612 | -0.0063 | 0.019018 | 4 | *ABCG2* | TSS200 | NA | - |
| 3959 | cg09763167 | -0.0023 | 0.019016 | 17 | *-* | - | NA | - |
| 3960 | cg15417244 | -0.0083 | 0.019026 | 12 | *PDZRN4* | 5'UTR | TRUE | - |
| 3961 | cg19356311 | -0.003 | 0.019028 | 1 | *-* | - | TRUE | N_Shelf |
| 3962 | cg25409223 | -0.0021 | 0.019026 | 16 | *CDH16* | TSS200 | NA | N_Shelf |
| 3963 | cg14132225 | 0.0052 | 0.01908 | 6 | *-* | - | NA | - |
| 3964 | cg12619254 | -0.0041 | 0.019099 | 6 | *LOC100131047* | Body | NA | - |
| 3965 | cg10185057 | -0.0034 | 0.019109 | 11 | *MARK2* | Body | NA | - |
| 3966 | cg14175823 | -0.0035 | 0.019099 | 16 | *D-JA2* | Body | NA | - |
| 3967 | cg01438868 | -0.0048 | 0.019119 | 3 | *-* | - | TRUE | - |
| 3968 | cg06281680 | -0.0027 | 0.019119 | 2 | *-* | - | TRUE | N_Shore |
| 3969 | cg24993238 | -0.0021 | 0.019119 | 17 | *BIRC5* | TSS1500 | NA | N_Shore |
| 3970 | cg16181196 | -0.0078 | 0.019131 | 17 | *STXBP4* | Body | NA | - |
| 3971 | cg01890712 | 0.0051 | 0.019142 | 11 | *OR9Q1* | TSS1500 | TRUE | - |
| 3972 | cg10800572 | -0.0037 | 0.019142 | 22 | *PRR34AS1* | Body | NA | S_Shore |
| 3973 | cg11383720 | 0.0017 | 0.019142 | 2 | *LOC101927438* | Body | NA | S_Shore |
| 3974 | cg24705329 | -0.004 | 0.019168 | 4 | *-* | - | NA | - |
| 3975 | cg13781721 | 0.0029 | 0.01919 | X | *MBNL3* | Body | TRUE | - |
| 3976 | cg06159340 | -0.0045 | 0.019199 | 16 | *CTF1* | TSS1500 | TRUE | S_Shore |
| 3977 | cg27307183 | -0.0021 | 0.019208 | 6 | *CDYL* | 5'UTR | TRUE | - |
| 3978 | cg08057010 | -0.0034 | 0.019237 | 1 | *LINC01350* | Body | NA | - |
| 3979 | cg04882213 | -0.0039 | 0.019244 | 8 | *-* | - | TRUE | - |
| 3980 | cg25143508 | -0.0024 | 0.01926 | 1 | *PRELP* | 5'UTR | TRUE | - |
| 3981 | cg05072629 | -0.0049 | 0.019269 | 7 | *-* | - | NA | - |
| 3982 | cg18113602 | -0.0023 | 0.019269 | 3 | *CMTM6* | TSS1500 | TRUE | S_Shore |
| 3983 | cg00176635 | -0.0027 | 0.019295 | 19 | *UHRF1* | Body | NA | - |
| 3984 | cg15542713 | 0.0094 | 0.019341 | 1 | *HIVEP3* | TSS1500 | TRUE | S_Shore |
| 3985 | cg24964047 | 0.0031 | 0.019339 | 17 | *DGKE* | Body | NA | - |
| 3986 | cg07717344 | 0.0021 | 0.019343 | 9 | *LOC101927502* | Body | NA | - |
| 3987 | cg11031774 | -0.0036 | 0.019343 | 12 | *PCED1BAS1* | Body | NA | - |
| 3988 | cg22825269 | -0.0039 | 0.019343 | 4 | *DCLK2* | TSS1500 | NA | N_Shore |
| 3989 | cg24077102 | 0.0023 | 0.019343 | 11 | *C11orf85* | TSS200 | NA | Island |
| 3990 | cg03430502 | 0.0011 | 0.019348 | 3 | *GAP43* | 1stExon | TRUE | - |
| 3991 | cg14525247 | 0.0048 | 0.019343 | 9 | *MIR2192* | Body | TRUE | Island |
| 3992 | cg17949057 | 0.0029 | 0.019343 | 2 | *CYTIP* | Body | NA | - |
| 3993 | cg27519145 | -0.0034 | 0.019343 | 1 | *FAM78B* | Body | TRUE | - |
| 3994 | cg06799152 | 0.0033 | 0.019356 | 12 | *LRP1* | Body | NA | - |
| 3995 | cg09657446 | 0.0027 | 0.01935 | 9 | *FAM102A* | Body | NA | S_Shelf |
| 3996 | cg12708124 | -0.0022 | 0.019431 | 10 | *-* | - | TRUE | - |
| 3997 | cg21586203 | -0.0019 | 0.019424 | 7 | *FLJ23834* | Body | TRUE | S_Shore |
| 3998 | cg01569875 | -0.0018 | 0.019438 | 16 | *SYT17* | Body | TRUE | N_Shore |
| 3999 | cg16045390 | -0.0025 | 0.019461 | 17 | *GRB7* | 5'UTR | TRUE | - |
| 4000 | cg27332104 | -0.0052 | 0.019461 | 15 | *-* | - | TRUE | - |
| 4001 | cg11092487 | 0.0074 | 0.019467 | 20 | *MYH7B* | Body | TRUE | Island |
| 4002 | cg20363083 | -0.0029 | 0.019473 | 6 | *-* | - | NA | - |
| 4003 | cg08062407 | 0.0051 | 0.019496 | 19 | *-* | - | NA | - |
| 4004 | cg15264878 | -0.0052 | 0.019514 | 13 | *-* | - | NA | - |
| 4005 | cg22996440 | 0.0029 | 0.019511 | 2 | *GALNT14* | TSS1500 | TRUE | Island |
| 4006 | cg09889997 | -0.0018 | 0.01954 | 1 | *CAMTA1* | Body | TRUE | - |
| 4007 | cg06688910 | -0.0028 | 0.019572 | 8 | *-* | - | TRUE | - |
| 4008 | cg03667292 | -0.0068 | 0.019595 | 9 | *VLDLRAS1* | Body | NA | - |
| 4009 | cg20382695 | 0.0014 | 0.019621 | 10 | *ATRNL1* | Body | TRUE | Island |
| 4010 | cg10253780 | -0.0037 | 0.019631 | 18 | *BCL2* | Body | NA | - |
| 4011 | cg23859086 | -0.0029 | 0.019626 | 1 | *-* | - | NA | N_Shelf |
| 4012 | cg18546989 | 0.0043 | 0.019659 | 6 | *ARID1B* | Body | NA | - |
| 4013 | cg00386408 | -0.0029 | 0.019675 | 5 | *TGFBI* | Body | TRUE | Island |
| 4014 | cg07415266 | -0.0038 | 0.019671 | 2 | *IL18RAP* | Body | NA | - |
| 4015 | cg08877374 | 0.0029 | 0.019696 | 1 | *FABP3* | 1stExon | TRUE | - |
| 4016 | cg08939418 | -0.0021 | 0.019717 | 7 | *IGF2BP3* | TSS1500 | TRUE | S_Shore |
| 4017 | cg18325389 | -0.0033 | 0.019717 | 7 | *-* | - | TRUE | - |
| 4018 | cg18634760 | -0.0029 | 0.019724 | 13 | *CPB2* | TSS200 | TRUE | - |
| 4019 | cg19833632 | 0.0013 | 0.019724 | 3 | *PFN2* | TSS1500 | NA | S_Shore |
| 4020 | cg07346187 | -0.0021 | 0.019758 | 6 | *ZC3H12D* | Body | TRUE | N_Shelf |
| 4021 | cg07912766 | 0.003 | 0.019795 | 18 | *SMAD2* | TSS1500 | TRUE | S_Shore |
| 4022 | cg06633081 | -0.0037 | 0.01981 | 14 | *SAMD4A* | Body | TRUE | - |
| 4023 | cg26422861 | -0.003 | 0.019807 | 7 | *PHKG1* | TSS200 | TRUE | - |
| 4024 | cg02180665 | -0.0029 | 0.019864 | 6 | *-* | - | NA | - |
| 4025 | cg07670251 | -0.0016 | 0.019864 | 2 | *-* | - | NA | - |
| 4026 | cg14143795 | -0.0032 | 0.019866 | 16 | *D-H3* | Body | NA | - |
| 4027 | cg14346895 | 0.0019 | 0.019864 | 9 | *FBXO10* | TSS1500 | TRUE | S_Shore |
| 4028 | cg15091333 | 0.0028 | 0.019905 | 7 | *-* | - | NA | - |
| 4029 | cg16478906 | 0.0035 | 0.019906 | 4 | *SH3TC1* | Body | NA | - |
| 4030 | cg09081994 | 0.0028 | 0.019909 | 3 | *CDGAP* | TSS1500 | TRUE | N_Shore |
| 4031 | cg02075432 | -0.0037 | 0.019924 | 6 | *-* | - | NA | - |
| 4032 | cg15965233 | -0.0036 | 0.019944 | 2 | *COBLL1* | Body | NA | - |
| 4033 | cg02031676 | 0.0058 | 0.019963 | 12 | *TMTC1* | Body | NA | - |
| 4034 | cg22050642 | -0.003 | 0.019959 | 16 | *ZNF778* | 5'UTR | NA | S_Shore |
| 4035 | cg23660404 | -0.0051 | 0.019972 | 8 | *-* | - | NA | - |
| 4036 | cg12131208 | 0.0048 | 0.020005 | 17 | *C17orf64* | TSS200 | TRUE | S_Shore |
| 4037 | cg00166216 | 0.005 | 0.020009 | 3 | *FAM43A* | 1stExon | TRUE | Island |
| 4038 | cg01401327 | -0.0049 | 0.020006 | 7 | *-* | - | TRUE | N_Shore |
| 4039 | cg01811355 | -0.0027 | 0.020009 | 8 | *-* | - | NA | - |
| 4040 | cg25083305 | -0.0046 | 0.020006 | 2 | *ALK* | Body | NA | - |
| 4041 | cg02991078 | 0.0031 | 0.020033 | 4 | *SH3TC1* | Body | NA | - |
| 4042 | cg08950945 | -0.0073 | 0.020033 | 9 | *MUSK* | Body | NA | - |
| 4043 | cg11984161 | 0.0028 | 0.02005 | 19 | *LSR* | Body | NA | N_Shore |
| 4044 | cg16774382 | -0.0034 | 0.020093 | 7 | *OR6V1* | TSS1500 | NA | - |
| 4045 | cg08106973 | -0.0019 | 0.020093 | 1 | *-* | - | TRUE | - |
| 4046 | cg23099476 | 0.0044 | 0.020094 | 14 | *FOS* | Body | NA | Island |
| 4047 | cg07165610 | -0.0032 | 0.020099 | 3 | *XIRP1* | 5'UTR | TRUE | - |
| 4048 | cg14407055 | -0.0024 | 0.020099 | 5 | *COL23A1* | Body | NA | - |
| 4049 | cg23983188 | -0.0026 | 0.020101 | 12 | *CCDC60* | Body | NA | - |
| 4050 | cg25609878 | -0.0031 | 0.020099 | 4 | *-* | - | TRUE | - |
| 4051 | cg01080924 | -0.0062 | 0.020105 | 22 | *C22orf26* | Body | TRUE | N_Shore |
| 4052 | cg02355607 | -0.004 | 0.020105 | 9 | *-* | - | NA | - |
| 4053 | cg02609911 | -0.0021 | 0.020105 | 8 | *-* | - | NA | - |
| 4054 | cg03972838 | -0.0019 | 0.020105 | 22 | *SMCR7L* | TSS1500 | TRUE | N_Shore |
| 4055 | cg26129669 | -0.0042 | 0.020118 | 6 | *GABBR1* | Body | TRUE | N_Shore |
| 4056 | cg22406102 | -0.002 | 0.020135 | 6 | *-* | - | TRUE | - |
| 4057 | cg20975682 | -0.0022 | 0.02014 | 12 | *PRPF40B* | 5'UTR | TRUE | S_Shelf |
| 4058 | cg20329210 | 0.0016 | 0.020156 | 1 | *OBSCN* | Body | TRUE | Island |
| 4059 | cg21141726 | -0.0038 | 0.020164 | 11 | *-* | - | NA | - |
| 4060 | cg03253303 | -0.0028 | 0.020168 | 17 | *RAB11FIP4* | Body | TRUE | - |
| 4061 | cg08664199 | -0.0023 | 0.020199 | 17 | *AATF* | Body | NA | - |
| 4062 | cg11924683 | -0.0022 | 0.020204 | 4 | *SORCS2* | Body | TRUE | - |
| 4063 | cg07712198 | -0.0043 | 0.02022 | 5 | *-* | - | TRUE | - |
| 4064 | cg10680133 | 0.0026 | 0.020228 | 8 | *-* | - | NA | - |
| 4065 | cg26394940 | -0.0059 | 0.020239 | 22 | *C22orf26* | Body | TRUE | N_Shore |
| 4066 | cg09918713 | -0.0056 | 0.020252 | 16 | *PAGR1* | 3'UTR | NA | - |
| 4067 | cg27617964 | -0.0025 | 0.020257 | 19 | *-* | - | NA | - |
| 4068 | cg00504629 | 0.0037 | 0.02026 | 19 | *PPP5D1* | Body | NA | - |
| 4069 | cg12700677 | 0.0019 | 0.020262 | 3 | *CCDC13* | TSS1500 | NA | S_Shore |
| 4070 | cg08695830 | -0.0022 | 0.020295 | 7 | *CLIP2* | TSS1500 | TRUE | Island |
| 4071 | cg16171137 | -0.0054 | 0.020312 | 6 | *BAT3* | Body | TRUE | - |
| 4072 | cg22363511 | -0.0048 | 0.020312 | 14 | *MIPOL1* | 3'UTR | NA | - |
| 4073 | cg23143632 | 0.0011 | 0.020312 | 19 | *LTBP4* | Body | NA | Island |
| 4074 | cg09420267 | -0.0028 | 0.020312 | 2 | *-* | - | NA | - |
| 4075 | cg22518946 | 0.0041 | 0.020312 | 1 | *SYT6* | Body | NA | - |
| 4076 | cg07247419 | 0.0021 | 0.020312 | 20 | *NKX24* | 3'UTR | TRUE | Island |
| 4077 | cg00431146 | -0.0021 | 0.020312 | 14 | *-* | - | NA | - |
| 4078 | cg04586023 | -0.0026 | 0.020312 | 22 | *HDAC10* | TSS1500 | TRUE | S_Shore |
| 4079 | cg12435707 | -0.0043 | 0.020347 | 15 | *SYNM* | Body | NA | - |
| 4080 | cg26663490 | -0.0024 | 0.020347 | 17 | *GPRC5C* | Body | TRUE | Island |
| 4081 | cg07316359 | -0.0034 | 0.020351 | 14 | *PCNX* | Body | NA | - |
| 4082 | cg01868263 | -0.0031 | 0.020358 | 18 | *PSTPIP2* | Body | NA | - |
| 4083 | cg27367871 | -0.0031 | 0.020396 | 2 | *CXCR7* | TSS1500 | TRUE | S_Shore |
| 4084 | cg20515823 | -0.0011 | 0.020416 | 12 | *PCED1BAS1* | TSS200 | NA | - |
| 4085 | cg03430537 | -0.0029 | 0.020416 | 15 | *-* | - | NA | - |
| 4086 | cg06841648 | 0.0028 | 0.020416 | 16 | *-* | - | TRUE | - |
| 4087 | cg07517370 | 0.0034 | 0.020445 | 10 | *TACC2* | TSS1500 | NA | N_Shore |
| 4088 | cg25593560 | -0.0035 | 0.020467 | 19 | *TPM4* | Body | TRUE | Island |
| 4089 | cg20793665 | 0.0034 | 0.020482 | 2 | *-* | - | TRUE | S_Shelf |
| 4090 | cg25902808 | -0.0026 | 0.020532 | 3 | *LINCR0002* | Body | NA | - |
| 4091 | cg05625006 | -0.0036 | 0.020575 | 22 | *-* | - | NA | - |
| 4092 | cg25520710 | 0.0048 | 0.020575 | 20 | *ZMYND8* | 5'UTR | NA | - |
| 4093 | cg04845871 | -0.0024 | 0.020575 | 1 | *-* | - | TRUE | - |
| 4094 | cg26464586 | -0.0024 | 0.020631 | 6 | *-* | - | TRUE | - |
| 4095 | cg00771218 | -0.0025 | 0.020659 | 8 | *TG* | Body | NA | - |
| 4096 | cg26290632 | 0.0017 | 0.020659 | 8 | *CALB1* | 1stExon | TRUE | - |
| 4097 | cg10581158 | 0.0024 | 0.02066 | 14 | *-* | - | NA | - |
| 4098 | cg16663147 | -0.0033 | 0.02066 | 7 | *-* | - | NA | - |
| 4099 | cg04811706 | -0.0117 | 0.020676 | 16 | *-* | - | TRUE | N_Shelf |
| 4100 | cg11005400 | 0.0019 | 0.020676 | 3 | *UTS2B* | 5'UTR | NA | - |
| 4101 | cg10859157 | 0.0047 | 0.020681 | 2 | *CSRNP3* | TSS200 | NA | - |
| 4102 | cg00073181 | 0.0039 | 0.020681 | 1 | *TLR5* | 5'UTR | NA | - |
| 4103 | cg02027349 | 0.0021 | 0.020687 | 9 | *ODF2* | 1stExon | NA | S_Shelf |
| 4104 | cg19298856 | 0.0027 | 0.02075 | 19 | *FLT3LG* | Body | TRUE | - |
| 4105 | cg19004007 | -0.0016 | 0.02076 | 10 | *INPP5F* | TSS1500 | TRUE | Island |
| 4106 | cg17825846 | -0.0039 | 0.02076 | 16 | *-* | - | NA | - |
| 4107 | cg16332003 | -0.0023 | 0.020784 | 12 | *-* | - | TRUE | S_Shelf |
| 4108 | cg27313482 | -0.0022 | 0.020796 | 2 | *MYO3B* | Body | NA | - |
| 4109 | cg17931883 | -0.0021 | 0.020808 | 2 | *LOC101927865* | Body | NA | - |
| 4110 | cg26369839 | -0.003 | 0.020813 | 19 | *ZSWIM4* | Body | NA | N_Shelf |
| 4111 | cg09822170 | -0.0058 | 0.020813 | 10 | *ACTA2* | 5'UTR | NA | - |
| 4112 | cg09873215 | -0.0048 | 0.020813 | 10 | *CNNM2* | Body | TRUE | - |
| 4113 | cg00952050 | 0.0052 | 0.020818 | 20 | *MYH7B* | Body | TRUE | Island |
| 4114 | cg09736073 | -0.0035 | 0.020822 | 5 | *-* | - | NA | - |
| 4115 | cg03563437 | -0.0061 | 0.020832 | 10 | *NSUN6* | Body | NA | - |
| 4116 | cg07491468 | 0.0023 | 0.020832 | 6 | *CDKAL1* | Body | NA | - |
| 4117 | cg13047724 | -0.0021 | 0.020844 | 2 | *FMNL2* | Body | NA | - |
| 4118 | cg27102378 | 0.0136 | 0.020844 | 22 | *-* | - | NA | - |
| 4119 | cg21114303 | 0.0028 | 0.020844 | X | *PHF8* | Body | TRUE | N_Shore |
| 4120 | cg20504084 | -0.0026 | 0.020856 | 8 | *FAM110B* | 5'UTR | NA | - |
| 4121 | cg02883072 | -0.0018 | 0.020872 | 22 | *FBLN1* | Body | NA | - |
| 4122 | cg04909958 | 0.0017 | 0.020921 | 5 | *FAM193B* | 1stExon | NA | S_Shore |
| 4123 | cg02686801 | 0.0023 | 0.020921 | 10 | *PIK3AP1* | Body | NA | - |
| 4124 | cg24939561 | -0.0035 | 0.020925 | 3 | *SEC62* | TSS1500 | NA | N_Shore |
| 4125 | cg23736055 | -0.0046 | 0.02094 | 6 | *C6orf145* | Body | TRUE | - |
| 4126 | cg17076667 | -0.003 | 0.020943 | 10 | *BAG3* | Body | TRUE | - |
| 4127 | cg03937945 | 0.0023 | 0.020955 | 21 | *-* | - | NA | - |
| 4128 | cg21683284 | 0.0027 | 0.020956 | 1 | *D-JC6* | Body | TRUE | N_Shore |
| 4129 | cg01486321 | 0.0027 | 0.020956 | 2 | *-* | - | NA | - |
| 4130 | cg27401724 | -0.0029 | 0.020998 | 17 | *ACBD4* | Body | TRUE | S_Shelf |
| 4131 | cg06631775 | -0.036 | 0.020999 | 12 | *-* | - | TRUE | Island |
| 4132 | cg16405432 | -0.0026 | 0.021003 | 14 | *-* | - | TRUE | - |
| 4133 | cg25275743 | -0.0039 | 0.021008 | 12 | *HECTD4* | 5'UTR | NA | - |
| 4134 | cg08608090 | -0.0056 | 0.021008 | 2 | *-* | - | NA | - |
| 4135 | cg01833991 | -0.0022 | 0.021048 | 20 | *LOC339593* | Body | NA | - |
| 4136 | cg10242160 | 0.0022 | 0.021057 | 4 | *ZAR1* | Body | TRUE | Island |
| 4137 | cg20431615 | -0.0029 | 0.021057 | 9 | *ST6GAL-C6* | 5'UTR | NA | N_Shore |
| 4138 | cg20941110 | 0.0011 | 0.021057 | 15 | *BNC1* | TSS1500 | TRUE | Island |
| 4139 | cg25556502 | -0.0022 | 0.021057 | 13 | *-* | - | NA | - |
| 4140 | cg18370151 | -0.0041 | 0.021057 | 22 | *-* | - | TRUE | Island |
| 4141 | cg17892169 | -0.0051 | 0.021065 | 17 | *TNFSF12* | Body | TRUE | Island |
| 4142 | cg25798632 | 0.0049 | 0.021065 | 17 | *TBC1D16* | TSS200 | NA | S_Shore |
| 4143 | cg15836427 | -0.0059 | 0.021065 | 7 | *-* | - | TRUE | - |
| 4144 | cg01747226 | -0.0039 | 0.021075 | 15 | *-* | - | NA | - |
| 4145 | cg21552014 | -0.0036 | 0.021095 | 3 | *-* | - | TRUE | - |
| 4146 | cg04193901 | -0.0042 | 0.021125 | 20 | *WISP2* | TSS200 | TRUE | - |
| 4147 | cg26854931 | 0.0022 | 0.021169 | 1 | *GUK1* | 5'UTR | NA | S_Shelf |
| 4148 | cg22359701 | -0.0041 | 0.021169 | 13 | *-* | - | NA | - |
| 4149 | cg21750800 | 0.0024 | 0.021178 | 16 | *CLEC16A* | Body | NA | - |
| 4150 | cg21642635 | 0.0033 | 0.02118 | 14 | *PPP2R5C* | Body | NA | - |
| 4151 | cg19248961 | -0.0027 | 0.021188 | 1 | *-* | - | NA | S_Shelf |
| 4152 | cg17320255 | -0.0023 | 0.021188 | 1 | *CHD5* | Body | NA | - |
| 4153 | cg26857408 | 0.0017 | 0.021267 | 17 | *UBTF* | Body | TRUE | S_Shore |
| 4154 | cg22673583 | -0.0023 | 0.021301 | 1 | *AADACL4* | Body | TRUE | - |
| 4155 | cg05540431 | 0.0019 | 0.021321 | 2 | *ACVR2A* | 5'UTR | NA | - |
| 4156 | cg00233028 | 0.0051 | 0.021334 | 11 | *-* | - | TRUE | - |
| 4157 | cg10771914 | -0.0021 | 0.021356 | 9 | *WNK2* | Body | NA | - |
| 4158 | cg25505563 | -0.007 | 0.021356 | 22 | *-* | - | NA | - |
| 4159 | cg14840468 | -0.0041 | 0.021378 | 14 | *-* | - | NA | - |
| 4160 | cg18032908 | -0.0021 | 0.021378 | 1 | *CNTN2* | Body | NA | - |
| 4161 | cg07904452 | -0.0028 | 0.021385 | 6 | *-* | - | TRUE | - |
| 4162 | cg06082318 | -0.0029 | 0.021385 | 13 | *-* | - | TRUE | - |
| 4163 | cg06861945 | -0.0033 | 0.021386 | 2 | *-* | - | NA | - |
| 4164 | cg10590376 | 0.002 | 0.021388 | 12 | *BTBD11* | Body | NA | - |
| 4165 | cg14209296 | -0.003 | 0.021399 | 7 | *IMMP2L* | Body | NA | - |
| 4166 | cg13025618 | 0.0021 | 0.021445 | 1 | *-* | - | NA | - |
| 4167 | cg20445053 | -0.002 | 0.021472 | 2 | *LASS6* | Body | TRUE | - |
| 4168 | cg24251283 | 0.0038 | 0.021499 | 16 | *-* | - | NA | - |
| 4169 | cg19177941 | 0.0018 | 0.021503 | 15 | *ALDH1A3* | 1stExon | TRUE | Island |
| 4170 | cg19076587 | -0.002 | 0.021544 | 11 | *PHF21A* | Body | TRUE | - |
| 4171 | cg01414357 | -0.0036 | 0.021563 | 19 | *SEPW1* | Body | TRUE | S_Shore |
| 4172 | cg22175552 | -0.0036 | 0.021581 | 1 | *RGL1* | Body | NA | - |
| 4173 | cg24547636 | -0.0023 | 0.021629 | 6 | *-* | - | NA | - |
| 4174 | cg17702583 | -0.002 | 0.021645 | 4 | *KDR* | 3'UTR | NA | - |
| 4175 | cg00986236 | 0.0045 | 0.021656 | 3 | *ADAMTS9* | Body | NA | - |
| 4176 | cg22448874 | 0.004 | 0.021659 | 4 | *-* | - | TRUE | N_Shelf |
| 4177 | cg16948145 | 0.0022 | 0.021659 | 11 | *ADRBK1* | Body | NA | - |
| 4178 | cg17591857 | 0.0025 | 0.021688 | 17 | *MYO18A* | Body | NA | - |
| 4179 | cg07879474 | 0.0028 | 0.02169 | 5 | *-* | - | TRUE | S_Shelf |
| 4180 | cg19726666 | -0.0031 | 0.021706 | 1 | *LOC101927876* | TSS1500 | NA | - |
| 4181 | cg05243865 | -0.0023 | 0.021732 | 6 | *LOC285768* | Body | NA | - |
| 4182 | cg04579707 | -0.0031 | 0.021738 | 18 | *-* | - | NA | - |
| 4183 | cg17945323 | -0.0027 | 0.021738 | 11 | *SLC22A8* | Body | TRUE | - |
| 4184 | cg03939424 | 0.0026 | 0.021776 | 11 | *-* | - | NA | - |
| 4185 | cg15019006 | 0.0016 | 0.021778 | 9 | *TRAF2* | Body | NA | - |
| 4186 | cg13532659 | 0.0025 | 0.021781 | 13 | *PCDH9* | Body | NA | - |
| 4187 | cg13567403 | -0.0035 | 0.021781 | 9 | *-* | - | TRUE | - |
| 4188 | cg07101980 | -0.0028 | 0.021803 | 11 | *NDUFC2* | 3'UTR | TRUE | - |
| 4189 | cg08873209 | 0.0017 | 0.021826 | 1 | *NFYC* | 1stExon | NA | - |
| 4190 | cg02968445 | -0.0056 | 0.021826 | 2 | *ARHGAP25* | Body | NA | - |
| 4191 | cg12590902 | 0.002 | 0.021855 | 1 | *ERI3* | Body | TRUE | - |
| 4192 | cg08151705 | 0.0019 | 0.021861 | 17 | *SLFN13* | TSS200 | TRUE | N_Shore |
| 4193 | cg19413066 | -0.0027 | 0.021877 | 17 | *-* | - | TRUE | - |
| 4194 | cg11376472 | -0.0019 | 0.021912 | 7 | *CREB3L2* | Body | NA | - |
| 4195 | cg26558412 | -0.0042 | 0.021936 | 4 | *-* | - | NA | - |
| 4196 | cg02036851 | -0.0048 | 0.021942 | 20 | *-* | - | NA | - |
| 4197 | cg26055210 | -0.0014 | 0.021974 | 11 | *AH-K* | 5'UTR | TRUE | N_Shelf |
| 4198 | cg22117723 | -0.0031 | 0.021982 | 11 | *MUC2* | Body | TRUE | Island |
| 4199 | cg21355059 | -0.0019 | 0.021986 | 4 | *FRYL* | 5'UTR | NA | - |
| 4200 | cg26931154 | -0.0039 | 0.021986 | 3 | *-* | - | NA | - |
| 4201 | cg04017413 | -0.0043 | 0.021986 | 15 | *-* | - | NA | S_Shelf |
| 4202 | cg26182780 | 0.003 | 0.021995 | 2 | *AGAP1* | Body | TRUE | N_Shore |
| 4203 | cg02822838 | -0.002 | 0.021995 | 6 | *TFEB* | TSS1500 | TRUE | S_Shore |
| 4204 | cg14040894 | -0.003 | 0.022026 | 4 | *-* | - | NA | - |
| 4205 | cg13022784 | -0.0043 | 0.022026 | 8 | *MAPK15* | Body | NA | S_Shore |
| 4206 | cg22051854 | -0.004 | 0.02204 | 1 | *MACF1* | Body | NA | - |
| 4207 | cg11447922 | -0.002 | 0.022055 | 17 | *CARD14* | Body | TRUE | N_Shelf |
| 4208 | cg26201109 | 0.0022 | 0.022057 | 3 | *ERC2* | Body | NA | - |
| 4209 | cg16106768 | -0.0036 | 0.022102 | 14 | *-* | - | NA | - |
| 4210 | cg07470207 | -0.0031 | 0.022102 | 8 | *-* | - | TRUE | - |
| 4211 | cg06204376 | 0.0011 | 0.02212 | 9 | *PAX5* | TSS200 | NA | Island |
| 4212 | cg04519462 | -0.0034 | 0.022157 | 17 | *HRNBP3* | 5'UTR | TRUE | - |
| 4213 | cg20180364 | -0.0019 | 0.022157 | 10 | *HHEX* | TSS1500 | TRUE | N_Shore |
| 4214 | cg05907177 | -0.0047 | 0.022157 | 1 | *-* | - | NA | - |
| 4215 | cg21235151 | 0.0023 | 0.022157 | 2 | *-* | - | TRUE | Island |
| 4216 | cg15360181 | -0.0038 | 0.022157 | 3 | *SLC9A9* | TSS200 | TRUE | - |
| 4217 | cg20273725 | 0.0016 | 0.022157 | 5 | *-* | - | NA | - |
| 4218 | cg01225248 | 0.0022 | 0.022157 | 3 | *-* | - | NA | - |
| 4219 | cg19370097 | -0.0058 | 0.022157 | 3 | *ZBTB20* | TSS1500 | NA | - |
| 4220 | cg12385825 | -0.0027 | 0.022158 | 14 | *AKAP6* | Body | NA | - |
| 4221 | cg25150715 | -0.003 | 0.022158 | 12 | *C12orf43* | TSS1500 | NA | S_Shore |
| 4222 | cg14699734 | 0.003 | 0.022158 | 19 | *-* | - | TRUE | S_Shore |
| 4223 | cg17430979 | -0.0057 | 0.022158 | 4 | *JAKMIP1* | Body | TRUE | - |
| 4224 | cg05338066 | -0.005 | 0.022158 | 1 | *CAMTA1* | Body | TRUE | - |
| 4225 | cg10590964 | -0.0068 | 0.022167 | 2 | *-* | - | TRUE | - |
| 4226 | cg02962508 | 0.0023 | 0.022171 | 8 | *-* | - | NA | N_Shore |
| 4227 | cg12149665 | -0.0021 | 0.022172 | 14 | *-* | - | NA | - |
| 4228 | cg20528917 | -0.0034 | 0.022238 | 1 | *REG4* | 5'UTR | NA | - |
| 4229 | cg10251538 | -0.0039 | 0.022238 | 3 | *MORC1* | Body | NA | - |
| 4230 | cg12580930 | 0.0031 | 0.022238 | 11 | *-* | - | TRUE | S_Shore |
| 4231 | cg21901395 | -0.0027 | 0.022262 | 17 | *-* | - | TRUE | N_Shore |
| 4232 | cg26033937 | -0.0021 | 0.022282 | 13 | *TM9SF2* | Body | NA | - |
| 4233 | cg06623698 | -0.0053 | 0.022289 | 6 | *PACSIN1* | 5'UTR | TRUE | Island |
| 4234 | cg22740110 | 0.0015 | 0.022289 | 1 | *-* | - | NA | N_Shore |
| 4235 | cg23573187 | 0.003 | 0.022289 | 2 | *-* | - | NA | - |
| 4236 | cg01725658 | 0.0026 | 0.022292 | 6 | *CDKAL1* | Body | NA | - |
| 4237 | cg15706574 | -0.0075 | 0.022302 | 6 | *RCAN2* | Body | NA | - |
| 4238 | cg27272447 | -0.0039 | 0.022328 | 12 | *PPFIBP1* | 5'UTR | NA | - |
| 4239 | cg16862893 | -0.0023 | 0.022331 | 7 | *-* | - | TRUE | N_Shore |
| 4240 | cg06544989 | -0.003 | 0.022378 | 22 | *UNC84B* | 3'UTR | TRUE | - |
| 4241 | cg13635516 | 0.0014 | 0.022389 | 20 | *-* | - | TRUE | N_Shore |
| 4242 | cg20170989 | -0.0034 | 0.022403 | 7 | *POM121L12* | TSS1500 | TRUE | N_Shore |
| 4243 | cg14630856 | -0.0047 | 0.022411 | 1 | *-* | - | NA | - |
| 4244 | cg20819878 | -0.004 | 0.022444 | 8 | *C8orf88* | TSS1500 | NA | S_Shore |
| 4245 | cg15131146 | 0.0024 | 0.022457 | 1 | *MAN1C1* | Body | TRUE | S_Shelf |
| 4246 | cg03560743 | 0.0015 | 0.022482 | 17 | *-* | - | NA | - |
| 4247 | cg18885584 | -0.0019 | 0.022493 | 13 | *-* | - | NA | - |
| 4248 | cg09576762 | -0.0051 | 0.022493 | 15 | *LOC145814* | 5'UTR | TRUE | - |
| 4249 | cg19659741 | 0.009 | 0.022501 | 8 | *ADAM5P* | TSS200 | TRUE | - |
| 4250 | cg18553399 | 0.0037 | 0.022502 | 16 | *UNKL* | Body | NA | S_Shore |
| 4251 | cg06562969 | 0.0042 | 0.022516 | 13 | *EPSTI1* | TSS1500 | NA | S_Shore |
| 4252 | cg05641859 | -0.0033 | 0.022576 | 18 | *NEDD4L* | Body | NA | - |
| 4253 | cg15848231 | -0.0028 | 0.022585 | 20 | *-* | - | NA | - |
| 4254 | cg15456476 | -0.0019 | 0.022586 | 16 | *FAM92B* | TSS200 | TRUE | - |
| 4255 | cg03433230 | 0.0035 | 0.022594 | 7 | *-* | - | NA | - |
| 4256 | cg18912541 | -0.0024 | 0.022595 | 14 | *DCAF5* | Body | TRUE | - |
| 4257 | cg04252852 | -0.0021 | 0.022621 | 1 | *ATXN7L2* | 1stExon | NA | N_Shelf |
| 4258 | cg01974375 | -0.0028 | 0.022674 | 1 | *PI4KB* | TSS1500 | TRUE | N_Shore |
| 4259 | cg23287816 | -0.0052 | 0.022674 | 14 | *-* | - | NA | - |
| 4260 | cg22003512 | 0.0037 | 0.022684 | 7 | *CDK14* | Body | NA | - |
| 4261 | cg18731398 | -0.004 | 0.022732 | 3 | *MYLK* | Body | TRUE | - |
| 4262 | cg16642281 | 0.0014 | 0.022818 | 4 | *LEF1* | Body | TRUE | - |
| 4263 | cg15001747 | 0.0017 | 0.022863 | 17 | *-* | - | TRUE | Island |
| 4264 | cg09985344 | 0.0033 | 0.022863 | 16 | *COTL1* | Body | TRUE | N_Shelf |
| 4265 | cg03432101 | -0.0044 | 0.022892 | 13 | *-* | - | NA | - |
| 4266 | cg24754626 | -0.0021 | 0.022892 | 2 | *-* | - | NA | - |
| 4267 | cg11519073 | 0.0046 | 0.022892 | 6 | *-* | - | NA | - |
| 4268 | cg22290972 | -0.0019 | 0.022908 | 8 | *-* | - | NA | - |
| 4269 | cg24332002 | -0.0027 | 0.022908 | 1 | *HSPG2* | Body | TRUE | - |
| 4270 | cg07606171 | -0.0032 | 0.022937 | 16 | *-* | - | NA | - |
| 4271 | cg26924646 | -0.0025 | 0.022937 | 11 | *C11orf49* | Body | NA | - |
| 4272 | cg09141931 | -0.0015 | 0.022946 | 17 | *RPTOR* | Body | TRUE | - |
| 4273 | cg21156263 | -0.0041 | 0.022983 | 9 | *-* | - | TRUE | - |
| 4274 | cg20534846 | -0.007 | 0.022983 | 9 | *-* | - | NA | - |
| 4275 | cg19906877 | 0.003 | 0.023004 | 6 | *-* | - | NA | - |
| 4276 | cg23396057 | -0.0022 | 0.023012 | 1 | *MYCBP* | TSS1500 | NA | S_Shore |
| 4277 | cg19757346 | -0.0019 | 0.023026 | 3 | *-* | - | NA | - |
| 4278 | cg02614932 | 0.0022 | 0.023036 | 10 | *DIP2C* | Body | TRUE | S_Shelf |
| 4279 | cg04583842 | 0.0049 | 0.023092 | 16 | *BANP* | Body | TRUE | S_Shore |
| 4280 | cg27102892 | 0.0021 | 0.023094 | 14 | *-* | - | NA | - |
| 4281 | cg17331032 | 0.0036 | 0.023177 | 9 | *FBP1* | Body | NA | - |
| 4282 | cg02070677 | -0.0038 | 0.02319 | 8 | *-* | - | NA | - |
| 4283 | cg27402362 | 0.0035 | 0.023219 | 1 | *-* | - | NA | - |
| 4284 | cg21837069 | -0.0025 | 0.023265 | 1 | *VWA1* | TSS1500 | TRUE | N_Shore |
| 4285 | cg14314729 | -0.0041 | 0.023274 | 5 | *-* | - | TRUE | N_Shore |
| 4286 | cg25607216 | -0.005 | 0.023274 | 11 | *-* | - | TRUE | - |
| 4287 | cg08692006 | -0.0019 | 0.023301 | 19 | *TNFAIP8L1* | TSS200 | TRUE | S_Shore |
| 4288 | cg05551937 | 0.0026 | 0.023301 | 4 | *REST* | 5'UTR | NA | S_Shore |
| 4289 | cg10812186 | 0.0062 | 0.023322 | 6 | *TNXB* | Body | TRUE | Island |
| 4290 | cg25494045 | 0.0042 | 0.02333 | 16 | *CHST4* | TSS1500 | NA | - |
| 4291 | cg23552977 | -0.0036 | 0.023331 | 14 | *NDRG2* | 5'UTR | TRUE | N_Shore |
| 4292 | cg04850714 | -0.0034 | 0.023348 | 9 | *FAM73B* | Body | NA | - |
| 4293 | cg20734611 | -0.0022 | 0.023363 | 22 | *PHF21B* | Body | NA | - |
| 4294 | cg13632168 | 0.0027 | 0.023364 | 4 | *-* | - | NA | - |
| 4295 | cg01887846 | -0.0033 | 0.023369 | 19 | *ATG4D* | Body | NA | S_Shore |
| 4296 | cg05036656 | 0.0015 | 0.023369 | 4 | *-* | - | TRUE | Island |
| 4297 | cg06460333 | -0.0034 | 0.023369 | 11 | *CCDC73* | 5'UTR | NA | - |
| 4298 | cg24671330 | 0.0031 | 0.023369 | 3 | *ATP13A5* | Body | TRUE | - |
| 4299 | cg16495504 | -0.0029 | 0.023387 | 9 | *-* | - | NA | - |
| 4300 | cg26745861 | -0.0041 | 0.023387 | 2 | *KIF1A* | Body | NA | - |
| 4301 | cg14294215 | 0.0055 | 0.023387 | 11 | *-* | - | NA | Island |
| 4302 | cg12603632 | -0.0049 | 0.023387 | 13 | *-* | - | TRUE | - |
| 4303 | cg05581731 | 0.0019 | 0.023391 | 17 | *-* | - | NA | - |
| 4304 | cg09658958 | 0.0016 | 0.023391 | 8 | *LYN* | Body | NA | - |
| 4305 | cg01249544 | -0.0022 | 0.023391 | 10 | *CTN-3* | Body | TRUE | - |
| 4306 | cg05113927 | 0.0054 | 0.023391 | 2 | *UCN* | TSS200 | TRUE | Island |
| 4307 | cg09886558 | -0.0025 | 0.023391 | 15 | *-* | - | TRUE | - |
| 4308 | cg08310088 | -0.0034 | 0.023391 | 16 | *-* | - | TRUE | - |
| 4309 | cg01438428 | 0.0025 | 0.023391 | 10 | *-* | - | NA | - |
| 4310 | cg01220564 | 0.0029 | 0.023396 | 7 | *DDC* | 5'UTR | TRUE | - |
| 4311 | cg25917590 | -0.0042 | 0.023408 | 20 | *-* | - | NA | - |
| 4312 | cg14266032 | -0.002 | 0.023425 | 15 | *THSD4* | Body | NA | - |
| 4313 | cg06987468 | 0.0016 | 0.023466 | 7 | *WNT2* | TSS200 | TRUE | Island |
| 4314 | cg06089049 | -0.0028 | 0.023476 | 20 | *APCDD1LAS1* | Body | NA | - |
| 4315 | cg05811250 | 0.0013 | 0.023501 | 9 | *STKLD1* | Body | NA | - |
| 4316 | cg01267185 | -0.0024 | 0.023501 | 1 | *NM-T2* | TSS1500 | NA | - |
| 4317 | cg16226866 | -0.0039 | 0.023531 | 6 | *-* | - | TRUE | - |
| 4318 | cg24446969 | 0.003 | 0.023623 | 14 | *NRXN3* | Body | NA | - |
| 4319 | cg16474118 | -0.0025 | 0.02364 | 2 | *-* | - | TRUE | - |
| 4320 | cg11835347 | -0.003 | 0.023654 | 1 | *RHOC* | 5'UTR | TRUE | N_Shore |
| 4321 | cg18788040 | 0.0016 | 0.02366 | 3 | *-* | - | NA | - |
| 4322 | cg26404919 | -0.0033 | 0.02366 | 19 | *SMARCA4* | Body | NA | - |
| 4323 | cg12006375 | -0.0033 | 0.02366 | 13 | *GUCY1B2* | TSS200 | TRUE | - |
| 4324 | cg20286560 | -0.0026 | 0.023702 | 22 | *-* | - | NA | - |
| 4325 | cg23360388 | -0.0023 | 0.023706 | 5 | *FAM81B* | TSS1500 | TRUE | - |
| 4326 | cg10901368 | 0.0029 | 0.023706 | 3 | *SOX2OT* | Body | TRUE | N_Shelf |
| 4327 | cg24245594 | -0.0021 | 0.02373 | 11 | *ATG2A* | 3'UTR | NA | N_Shore |
| 4328 | cg26194477 | 0.0019 | 0.023744 | 14 | *FOXG1* | TSS1500 | TRUE | Island |
| 4329 | cg08917810 | -0.0034 | 0.023767 | 2 | *-* | - | NA | - |
| 4330 | cg25460807 | 0.0065 | 0.02382 | 8 | *-* | - | TRUE | S_Shelf |
| 4331 | cg16737749 | -0.0038 | 0.02382 | 4 | *MAPK10* | 5'UTR | TRUE | - |
| 4332 | cg11368302 | -0.003 | 0.02382 | 3 | *LMCD1AS1* | Body | NA | - |
| 4333 | cg11280790 | -0.012 | 0.023885 | 9 | *-* | - | NA | N_Shore |
| 4334 | cg21280392 | -0.0033 | 0.023901 | 17 | *PHOSPHO1* | 5'UTR | TRUE | S_Shore |
| 4335 | cg26951577 | -0.0059 | 0.023922 | 3 | *PTPRG* | Body | NA | - |
| 4336 | cg26161329 | 0.002 | 0.023989 | 17 | *PPM1E* | TSS1500 | TRUE | Island |
| 4337 | cg02927995 | -0.0028 | 0.02399 | 1 | *GLIS1* | 5'UTR | NA | - |
| 4338 | cg08020495 | -0.0031 | 0.024 | 4 | *LRBA* | Body | NA | - |
| 4339 | cg19837174 | 0.0044 | 0.024001 | 10 | *-* | - | TRUE | - |
| 4340 | cg13289177 | -0.0033 | 0.024005 | 2 | *LIMS1* | TSS1500 | NA | - |
| 4341 | cg07549924 | 0.0062 | 0.02401 | 4 | *ZNF595* | Body | TRUE | S_Shore |
| 4342 | cg13761615 | -0.0037 | 0.02401 | 5 | *MCC* | Body | NA | - |
| 4343 | cg15796437 | -0.0027 | 0.024021 | 21 | *-* | - | NA | - |
| 4344 | cg06661904 | -0.0021 | 0.024038 | 12 | *-* | - | NA | - |
| 4345 | cg27571448 | -0.0028 | 0.024054 | 9 | *-* | - | NA | - |
| 4346 | cg06651549 | -0.0027 | 0.024065 | 5 | *-* | - | NA | - |
| 4347 | cg04670451 | 0.0027 | 0.024065 | 16 | *LOC100129637* | Body | TRUE | N_Shore |
| 4348 | cg08700993 | 0.0047 | 0.024115 | 14 | *PELI2* | Body | NA | - |
| 4349 | cg21485970 | -0.0022 | 0.024115 | 8 | *C8orf34* | Body | NA | - |
| 4350 | cg13221767 | 0.0042 | 0.024122 | 11 | *UCP3* | TSS1500 | TRUE | - |
| 4351 | cg23461714 | -0.0025 | 0.024122 | 11 | *TTC12* | TSS1500 | TRUE | N_Shore |
| 4352 | cg14030146 | -0.0031 | 0.024123 | 7 | *-* | - | NA | N_Shore |
| 4353 | cg16750801 | -0.006 | 0.024149 | 10 | *PTPRE* | 5'UTR | TRUE | S_Shore |
| 4354 | cg11982045 | -0.0023 | 0.02415 | 17 | *-* | - | NA | N_Shelf |
| 4355 | cg04626879 | 0.0022 | 0.024159 | 15 | *FES* | Body | NA | - |
| 4356 | cg08618219 | -0.0028 | 0.024159 | 15 | *SEMA6D* | 5'UTR | NA | - |
| 4357 | cg02893604 | -0.0017 | 0.02419 | 14 | *PSMB11* | 3'UTR | TRUE | - |
| 4358 | cg16384189 | -0.003 | 0.024206 | 10 | *-* | - | NA | - |
| 4359 | cg12948278 | -0.0018 | 0.024264 | 17 | *NTN1* | Body | NA | - |
| 4360 | cg00347198 | 0.0021 | 0.024264 | 17 | *GALR2* | Body | TRUE | Island |
| 4361 | cg00606952 | -0.0019 | 0.024264 | 12 | *AQP5* | Body | NA | S_Shore |
| 4362 | cg03839074 | -0.0046 | 0.024282 | 5 | *-* | - | TRUE | N_Shore |
| 4363 | cg20268658 | -0.0042 | 0.024325 | 10 | *-* | - | NA | - |
| 4364 | cg25246416 | -0.0019 | 0.024325 | 11 | *DDX25* | Body | NA | - |
| 4365 | cg13429998 | 0.0048 | 0.024325 | 1 | *HIVEP3* | TSS1500 | NA | S_Shore |
| 4366 | cg12967137 | 0.0021 | 0.024325 | 14 | *C14orf23* | Body | TRUE | Island |
| 4367 | cg19652829 | 0.0017 | 0.024325 | 5 | *PIK3R1* | Body | NA | - |
| 4368 | cg11314684 | -0.0039 | 0.024325 | 1 | *AKT3* | Body | TRUE | - |
| 4369 | cg24473481 | -0.0022 | 0.024325 | 17 | *-* | - | NA | - |
| 4370 | cg02345747 | 0.0021 | 0.024325 | 3 | *LAMP3* | Body | TRUE | N_Shore |
| 4371 | cg14373646 | -0.0015 | 0.024332 | 7 | *ABCB1* | 5'UTR | NA | S_Shore |
| 4372 | cg24365969 | -0.0018 | 0.024332 | 1 | *ACTN2* | Body | NA | - |
| 4373 | cg05564087 | -0.0038 | 0.024332 | 10 | *-* | - | NA | - |
| 4374 | cg23725321 | 0.0038 | 0.024332 | 10 | *SEC31B* | TSS200 | TRUE | Island |
| 4375 | cg11048520 | -0.0091 | 0.024342 | 1 | *-* | - | NA | - |
| 4376 | cg20615795 | -0.0028 | 0.024342 | 4 | *ADGRL3* | Body | NA | - |
| 4377 | cg07104137 | -0.0021 | 0.024343 | 8 | *PREX2* | Body | NA | - |
| 4378 | cg16083236 | 0.0023 | 0.024358 | 14 | *PRKCH* | Body | NA | - |
| 4379 | cg19532942 | 0.0017 | 0.024421 | 14 | *-* | - | TRUE | - |
| 4380 | cg10778249 | -0.0019 | 0.024433 | 19 | *LIG1* | TSS1500 | TRUE | N_Shore |
| 4381 | cg15483426 | -0.0039 | 0.024439 | 7 | *TPK1* | Body | NA | - |
| 4382 | cg23052254 | -0.0015 | 0.024457 | 11 | *-* | - | NA | - |
| 4383 | cg18568570 | 0.0023 | 0.024461 | 7 | *TBXAS1* | Body | TRUE | - |
| 4384 | cg20517911 | -0.0029 | 0.024461 | 22 | *RBFOX2* | Body | NA | - |
| 4385 | cg08461746 | -0.0022 | 0.024461 | 2 | *KCNIP3* | Body | NA | S_Shore |
| 4386 | cg11757040 | -0.0044 | 0.024461 | 14 | *DPF3* | Body | TRUE | - |
| 4387 | cg03180426 | 0.0027 | 0.024461 | 9 | *C9orf140* | Body | TRUE | Island |
| 4388 | cg04382897 | 0.0015 | 0.024474 | 16 | *SNX29* | Body | NA | - |
| 4389 | cg17295489 | -0.0031 | 0.024474 | 12 | *DTX3* | TSS1500 | TRUE | - |
| 4390 | cg20224991 | -0.0024 | 0.024486 | 7 | *-* | - | TRUE | S_Shore |
| 4391 | cg12412575 | -0.0039 | 0.024486 | 15 | *AKAP13* | Body | TRUE | - |
| 4392 | cg24891133 | 0.0016 | 0.024494 | 13 | *C13orf33* | 1stExon | TRUE | Island |
| 4393 | cg08935541 | -0.0021 | 0.024552 | 22 | *LINC00899* | Body | NA | N_Shelf |
| 4394 | cg06650861 | 0.0036 | 0.024561 | 4 | *DDX60* | 5'UTR | NA | N_Shore |
| 4395 | cg11337097 | 0.0024 | 0.024562 | 2 | *AFF3* | Body | NA | - |
| 4396 | cg25355213 | 0.0023 | 0.024562 | 17 | *UNK* | TSS1500 | TRUE | N_Shore |
| 4397 | cg06708107 | -0.0036 | 0.024577 | 7 | *CHRM2* | 5'UTR | NA | - |
| 4398 | cg21697360 | -0.0032 | 0.024587 | 20 | *-* | - | NA | - |
| 4399 | cg11900923 | -0.0028 | 0.024587 | 3 | *-* | - | NA | N_Shore |
| 4400 | cg08878177 | -0.0028 | 0.024587 | 20 | *NKAIN4* | Body | TRUE | N_Shore |
| 4401 | cg05338961 | -0.003 | 0.024624 | 6 | *-* | - | NA | - |
| 4402 | cg19394169 | 0.0051 | 0.024686 | 17 | *RPTOR* | Body | TRUE | N_Shelf |
| 4403 | cg19130511 | -0.0031 | 0.024686 | 22 | *PRR34AS1* | Body | NA | S_Shore |
| 4404 | cg05369308 | -0.0035 | 0.024703 | 8 | *-* | - | NA | - |
| 4405 | cg04955914 | -0.0024 | 0.024703 | 2 | *C2orf24* | Body | TRUE | N_Shore |
| 4406 | cg14455466 | -0.0024 | 0.02474 | 5 | *-* | - | NA | - |
| 4407 | cg26645401 | 0.0017 | 0.02476 | 11 | *SWAP70* | Body | TRUE | S_Shore |
| 4408 | cg08050216 | -0.0026 | 0.024771 | 7 | *CDK14* | Body | NA | - |
| 4409 | cg11134246 | 0.0023 | 0.024771 | 1 | *CCDC24* | Body | TRUE | S_Shore |
| 4410 | cg21236182 | -0.0025 | 0.024803 | 2 | *-* | - | TRUE | - |
| 4411 | cg23117180 | -0.0046 | 0.024815 | 1 | *KIRREL* | Body | NA | - |
| 4412 | cg07421518 | 0.0035 | 0.024815 | 14 | *-* | - | NA | S_Shore |
| 4413 | cg23727813 | -0.002 | 0.024815 | 12 | *C12orf53* | 3'UTR | TRUE | - |
| 4414 | cg09131500 | -0.003 | 0.024815 | 13 | *LRCH1* | Body | NA | - |
| 4415 | cg27167546 | 0.003 | 0.024815 | 22 | *-* | - | NA | - |
| 4416 | cg03574656 | 0.0046 | 0.024815 | 2 | *FAM228B* | 5'UTR | NA | - |
| 4417 | cg19538026 | 0.0024 | 0.024815 | 17 | *TBCD* | Body | NA | S_Shore |
| 4418 | cg25194841 | -0.0023 | 0.024835 | 9 | *WDR38* | TSS1500 | NA | N_Shore |
| 4419 | cg15398801 | -0.003 | 0.024872 | 4 | *-* | - | NA | - |
| 4420 | cg12364755 | 0.0023 | 0.024874 | 17 | *PAFAH1B1* | 5'UTR | TRUE | S_Shore |
| 4421 | cg09789721 | 0.0025 | 0.024874 | 4 | *SORCS2* | Body | TRUE | - |
| 4422 | cg08472142 | -0.0029 | 0.024925 | 1 | *DCST1* | Body | TRUE | - |
| 4423 | cg11742595 | -0.0033 | 0.024946 | 16 | *NDRG4* | 5'UTR | NA | - |
| 4424 | cg23788167 | -9.00E-04 | 0.024946 | 9 | *ZFAND5* | 5'UTR | TRUE | Island |
| 4425 | cg07276449 | 0.0029 | 0.024946 | 17 | *TBC1D16* | Body | NA | S_Shore |
| 4426 | cg08360197 | -0.0036 | 0.024951 | 2 | *-* | - | NA | - |
| 4427 | cg16550651 | 0.0018 | 0.02496 | 17 | *KAT2A* | Body | TRUE | N_Shore |
| 4428 | cg01739327 | -0.002 | 0.024976 | 11 | *EED* | TSS1500 | TRUE | N_Shore |
| 4429 | cg00861100 | -0.0038 | 0.024976 | 13 | *HS6ST3* | Body | NA | - |
| 4430 | cg23903782 | 0.0015 | 0.024976 | 10 | *CAMK1D* | Body | NA | - |
| 4431 | cg26910511 | 0.0086 | 0.024982 | 19 | *LRFN1* | Body | TRUE | Island |
| 4432 | cg21524899 | -0.0019 | 0.025006 | 1 | *-* | - | TRUE | N_Shore |
| 4433 | cg10794874 | -0.0017 | 0.025011 | 1 | *-* | - | NA | - |
| 4434 | cg07710843 | 0.002 | 0.025011 | 22 | *MIR3909* | TSS1500 | NA | - |
| 4435 | cg13343932 | 0.0067 | 0.025011 | 17 | *SOCS3* | Body | NA | Island |
| 4436 | cg14572845 | -0.0018 | 0.025031 | 9 | *COL27A1* | Body | TRUE | - |
| 4437 | cg23972551 | -0.0014 | 0.025034 | 11 | *LRP5* | Body | TRUE | N_Shelf |
| 4438 | cg02495743 | -0.0032 | 0.025048 | 11 | *-* | - | TRUE | - |
| 4439 | cg10912634 | 0.003 | 0.025063 | 5 | *SRA1* | 3'UTR | TRUE | S_Shelf |
| 4440 | cg08616061 | 0.003 | 0.025063 | 5 | *PCDHGA4* | Body | TRUE | N_Shore |
| 4441 | cg14621448 | -0.0021 | 0.025063 | 1 | *-* | - | NA | S_Shelf |
| 4442 | cg10656654 | 0.0019 | 0.025063 | 16 | *-* | - | NA | - |
| 4443 | cg02343604 | 0.0033 | 0.025064 | 1 | *ID3* | 3'UTR | TRUE | N_Shore |
| 4444 | cg07180832 | -0.0019 | 0.025081 | 19 | *FBXO17* | TSS200 | NA | S_Shore |
| 4445 | cg00008500 | 0.0019 | 0.025117 | 14 | *-* | - | NA | - |
| 4446 | cg13456999 | -0.0025 | 0.025133 | 3 | *-* | - | NA | - |
| 4447 | cg18714560 | 0.0024 | 0.025183 | 11 | *SCT* | TSS1500 | TRUE | S_Shore |
| 4448 | cg11512544 | -0.0054 | 0.025185 | 13 | *LINC01309* | TSS1500 | NA | - |
| 4449 | cg19782686 | -0.0026 | 0.025185 | 4 | *SPATA5* | Body | TRUE | - |
| 4450 | cg02780919 | 0.0036 | 0.025185 | 11 | *NRIP3* | TSS1500 | TRUE | Island |
| 4451 | cg04239247 | -0.0026 | 0.025185 | 1 | *FNBP1L* | Body | TRUE | - |
| 4452 | cg01185345 | 0.0037 | 0.025185 | 13 | *STK24* | Body | TRUE | - |
| 4453 | cg16775095 | -0.0307 | 0.02521 | 15 | *-* | - | TRUE | N_Shore |
| 4454 | cg01438174 | -0.0026 | 0.025224 | 9 | *AK1* | TSS1500 | NA | S_Shore |
| 4455 | cg23064951 | -0.0015 | 0.025232 | 11 | *B3GAT3* | Body | TRUE | N_Shore |
| 4456 | cg24222580 | 0.005 | 0.025264 | 13 | *MCF2L* | Body | TRUE | S_Shelf |
| 4457 | cg08090640 | -0.0025 | 0.025293 | 17 | *IFI35* | Body | TRUE | - |
| 4458 | cg00481951 | 0.0017 | 0.025325 | 3 | *SST* | Body | TRUE | N_Shore |
| 4459 | cg11399975 | -0.0023 | 0.025325 | 5 | *C5orf64* | Body | NA | - |
| 4460 | cg24388008 | -0.002 | 0.025325 | 12 | *-* | - | NA | - |
| 4461 | cg26739849 | -0.002 | 0.025325 | 13 | *-* | - | TRUE | S_Shore |
| 4462 | cg03008244 | -0.002 | 0.025325 | 9 | *DPH7* | 1stExon | NA | - |
| 4463 | cg25311470 | -0.0026 | 0.025325 | 7 | *NRCAM* | 5'UTR | TRUE | - |
| 4464 | cg09959150 | -0.0029 | 0.025325 | 8 | *-* | - | TRUE | - |
| 4465 | cg25514148 | 0.0025 | 0.025325 | 11 | *-* | - | TRUE | - |
| 4466 | cg07008193 | -0.0032 | 0.025325 | 11 | *CREB3L1* | Body | TRUE | S_Shore |
| 4467 | cg08004178 | 0.0021 | 0.02533 | 11 | *-* | - | NA | - |
| 4468 | cg12785694 | 0.0027 | 0.02533 | 3 | *SMC4* | Body | TRUE | S_Shelf |
| 4469 | cg04812498 | 0.004 | 0.025348 | 11 | *-* | - | NA | - |
| 4470 | cg24430782 | 0.0032 | 0.025349 | 12 | *CAC-1C* | Body | NA | - |
| 4471 | cg00929286 | -0.0278 | 0.02537 | 14 | *ESRRB* | 5'UTR | NA | - |
| 4472 | cg04001714 | -0.0021 | 0.025378 | 2 | *KCNIP3* | Body | TRUE | S_Shelf |
| 4473 | cg15028548 | -0.0055 | 0.025378 | 3 | *ABI3BP* | 5'UTR | TRUE | - |
| 4474 | cg22104164 | 0.0016 | 0.025378 | 15 | *LOC283710* | TSS1500 | NA | - |
| 4475 | cg09290723 | -0.0058 | 0.025427 | 12 | *-* | - | NA | - |
| 4476 | cg07027457 | 0.0018 | 0.025428 | 16 | *CBFA2T3* | Body | TRUE | - |
| 4477 | cg03206681 | -0.0033 | 0.025428 | 3 | *-* | - | TRUE | - |
| 4478 | cg10812247 | -0.0033 | 0.025428 | 12 | *SLC16A7* | 3'UTR | TRUE | - |
| 4479 | cg13921444 | 0.0062 | 0.025445 | 4 | *SORBS2* | 5'UTR | TRUE | - |
| 4480 | cg06178299 | 0.0019 | 0.025452 | 1 | *PAX7* | Body | TRUE | Island |
| 4481 | cg05114676 | -0.0021 | 0.025452 | 14 | *DPF3* | Body | TRUE | - |
| 4482 | cg01514867 | -0.0025 | 0.025517 | 8 | *DPYS* | Body | NA | - |
| 4483 | cg21232161 | 0.0024 | 0.025532 | 2 | *MGAT5* | TSS1500 | TRUE | - |
| 4484 | cg08162803 | -0.0016 | 0.025536 | 11 | *TCIRG1* | TSS200 | TRUE | Island |
| 4485 | cg05750856 | -0.0027 | 0.025536 | 3 | *-* | - | NA | - |
| 4486 | cg02027609 | -0.0035 | 0.025564 | 2 | *-* | - | NA | - |
| 4487 | cg02476619 | -0.0019 | 0.025564 | 17 | *SLC47A1* | Body | NA | - |
| 4488 | cg15574588 | -0.0041 | 0.025693 | 5 | *-* | - | NA | - |
| 4489 | cg12105770 | 0.0024 | 0.02571 | 16 | *PPL* | Body | NA | - |
| 4490 | cg21393163 | -0.0033 | 0.025743 | 1 | *-* | - | TRUE | - |
| 4491 | cg20009354 | -0.0023 | 0.025756 | 14 | *-* | - | TRUE | - |
| 4492 | cg00990587 | 0.0022 | 0.025759 | 22 | *-* | - | TRUE | N_Shore |
| 4493 | cg15724607 | 0.0021 | 0.025759 | 5 | *EBF1* | Body | NA | - |
| 4494 | cg24010336 | -0.0046 | 0.025782 | 19 | *FBXO17* | TSS1500 | TRUE | S_Shore |
| 4495 | cg19426827 | -0.0024 | 0.025802 | 14 | *ADSSL1* | Body | TRUE | S_Shore |
| 4496 | cg21919729 | 0.0027 | 0.025802 | 8 | *CTSB* | 5'UTR | TRUE | - |
| 4497 | cg01105819 | -0.002 | 0.025809 | 16 | *-* | - | NA | - |
| 4498 | cg14202825 | 9.00E-04 | 0.025809 | 13 | *DGKH* | TSS1500 | TRUE | Island |
| 4499 | cg23615483 | 0.0027 | 0.025817 | 14 | *CCDC88C* | Body | TRUE | - |
| 4500 | cg27326514 | 0.0025 | 0.025839 | 8 | *STMN2* | Body | TRUE | S_Shore |
| 4501 | cg23600284 | -0.0013 | 0.025841 | 19 | *SLC8A2* | Body | NA | S_Shore |
| 4502 | cg24232083 | -0.0054 | 0.025842 | 7 | *-* | - | TRUE | - |
| 4503 | cg05568492 | -0.0047 | 0.025866 | 2 | *-* | - | NA | - |
| 4504 | cg25282715 | 0.0015 | 0.025872 | 17 | *ADAM11* | Body | TRUE | N_Shelf |
| 4505 | cg05425754 | 0.0012 | 0.025933 | 5 | *CCNO* | Body | TRUE | Island |
| 4506 | cg06075222 | -0.0031 | 0.025938 | 12 | *PLBD2* | Body | NA | S_Shore |
| 4507 | cg15698545 | -0.0091 | 0.02595 | 11 | *ME3* | TSS1500 | TRUE | S_Shore |
| 4508 | cg07180988 | 0.002 | 0.025956 | 12 | *-* | - | TRUE | - |
| 4509 | cg11827101 | -0.0024 | 0.025975 | 2 | *-* | - | TRUE | - |
| 4510 | cg27064949 | -0.0043 | 0.025984 | X | *DGAT2L6* | 1stExon | TRUE | - |
| 4511 | cg10951552 | -0.0025 | 0.025984 | 7 | *PLX-4* | Body | NA | - |
| 4512 | cg24312520 | 0.0048 | 0.026007 | 17 | *STAT3* | Body | TRUE | - |
| 4513 | cg09300343 | 0.0046 | 0.026011 | X | *-* | - | TRUE | N_Shore |
| 4514 | cg14090520 | -0.0016 | 0.026033 | 3 | *ITPR1* | Body | NA | - |
| 4515 | cg16406708 | 0.0023 | 0.026078 | 4 | *UCHL1* | Body | TRUE | S_Shore |
| 4516 | cg18317026 | -0.0033 | 0.026091 | 15 | *UNC13C* | Body | NA | - |
| 4517 | cg17435683 | -0.0089 | 0.026137 | 1 | *PRDM16* | Body | TRUE | - |
| 4518 | cg04444450 | -0.0034 | 0.026141 | 8 | *NCOA2* | 5'UTR | NA | - |
| 4519 | cg08517938 | -0.0015 | 0.02616 | 20 | *-* | - | NA | - |
| 4520 | cg25341338 | 0.001 | 0.026221 | 1 | *NHLH2* | 3'UTR | TRUE | Island |
| 4521 | cg22709988 | -0.0024 | 0.026244 | 5 | *-* | - | NA | - |
| 4522 | cg04198545 | 0.003 | 0.026251 | 3 | *-* | - | NA | S_Shore |
| 4523 | cg09481780 | 0.0043 | 0.026254 | 1 | *NFASC* | Body | NA | S_Shelf |
| 4524 | cg02151301 | -0.0028 | 0.026263 | 20 | *HM13* | TSS1500 | TRUE | N_Shore |
| 4525 | cg06698971 | -0.0023 | 0.026263 | 2 | *SPRED2* | Body | NA | - |
| 4526 | cg01712484 | -0.0041 | 0.026286 | 14 | *-* | - | NA | - |
| 4527 | cg14699112 | -0.0028 | 0.026287 | 4 | *-* | - | TRUE | N_Shelf |
| 4528 | cg16639915 | -0.002 | 0.026287 | 15 | *ZNF609* | Body | TRUE | - |
| 4529 | cg02304767 | -0.0039 | 0.026287 | 18 | *PHLPP1* | Body | NA | - |
| 4530 | cg06844864 | -0.0011 | 0.026287 | 12 | *INHBE* | Body | TRUE | N_Shelf |
| 4531 | cg00137168 | 0.0014 | 0.026287 | 5 | *HK3* | TSS200 | NA | - |
| 4532 | cg06126721 | -0.0043 | 0.026292 | 17 | *SLC43A2* | 3'UTR | TRUE | Island |
| 4533 | cg18733931 | 0.0035 | 0.026309 | 8 | *-* | - | NA | - |
| 4534 | cg24969141 | 0.0056 | 0.026334 | 14 | *WDR25* | Body | NA | - |
| 4535 | cg24321933 | -0.0018 | 0.026334 | 4 | *-* | - | NA | - |
| 4536 | cg06635946 | -0.003 | 0.026334 | 22 | *-* | - | TRUE | S_Shore |
| 4537 | cg25907671 | -0.0035 | 0.026338 | 17 | *ASB16* | Body | NA | - |
| 4538 | cg14947101 | -0.0033 | 0.02635 | 2 | *DYTN* | Body | NA | - |
| 4539 | cg27340201 | -0.0056 | 0.02635 | 12 | *HMGA2* | Body | NA | - |
| 4540 | cg02822921 | -0.0025 | 0.02635 | 1 | *TNFSF4* | TSS1500 | NA | - |
| 4541 | cg01586330 | 0.0032 | 0.026356 | 9 | *TMEM210* | TSS1500 | NA | N_Shore |
| 4542 | cg19613578 | -0.0032 | 0.026356 | 8 | *-* | - | NA | - |
| 4543 | cg19577080 | 0.0022 | 0.02638 | 4 | *INPP4B* | 5'UTR | TRUE | - |
| 4544 | cg00582970 | -0.0039 | 0.02639 | 17 | *-* | - | TRUE | - |
| 4545 | cg09897456 | -0.0033 | 0.0264 | 12 | *-* | - | NA | - |
| 4546 | cg14195952 | -0.0025 | 0.026421 | 8 | *FAM167AAS1* | Body | NA | - |
| 4547 | cg09949502 | -0.0021 | 0.026425 | 10 | *C10orf54* | Body | NA | - |
| 4548 | cg15951977 | -0.0031 | 0.026464 | 19 | *CARD8* | TSS1500 | NA | - |
| 4549 | cg01320510 | -0.0041 | 0.026464 | 17 | *PIK3R6* | TSS1500 | NA | - |
| 4550 | cg11954572 | -0.0023 | 0.026487 | 6 | *-* | - | NA | - |
| 4551 | cg25091134 | -0.003 | 0.02649 | 2 | *ODC1* | TSS1500 | NA | S_Shore |
| 4552 | cg22989649 | 9.00E-04 | 0.02649 | 19 | *UNC13A* | TSS1500 | TRUE | Island |
| 4553 | cg20374765 | 0.0031 | 0.026507 | 6 | *JARID2* | Body | NA | - |
| 4554 | cg23085662 | 0.0014 | 0.026508 | 18 | *-* | - | TRUE | Island |
| 4555 | cg17953764 | 0.0035 | 0.026521 | 4 | *ZAR1* | 1stExon | TRUE | Island |
| 4556 | cg08807759 | 0.0017 | 0.026521 | 1 | *-* | - | NA | - |
| 4557 | cg13449484 | 0.0029 | 0.026521 | 1 | *DYRK3* | 5'UTR | NA | S_Shore |
| 4558 | cg18818299 | -0.0025 | 0.026524 | 16 | *SRCAP* | Body | NA | - |
| 4559 | cg27262041 | -0.005 | 0.026529 | 11 | *0* | Body | TRUE | - |
| 4560 | cg04477962 | -0.0043 | 0.026529 | 12 | *METTL7A* | TSS1500 | TRUE | - |
| 4561 | cg01138819 | -0.0023 | 0.026536 | 5 | *-* | - | TRUE | N_Shore |
| 4562 | cg20822499 | -0.0031 | 0.026536 | 14 | *PCNX* | TSS1500 | NA | N_Shore |
| 4563 | cg14079719 | -0.0053 | 0.026542 | 6 | *OPRM1* | Body | NA | - |
| 4564 | cg08200404 | -0.0032 | 0.026548 | 15 | *C2CD4A* | 5'UTR | TRUE | N_Shore |
| 4565 | cg12051559 | -0.0038 | 0.026549 | 19 | *PLIN5* | Body | NA | S_Shore |
| 4566 | cg11199014 | 0.0089 | 0.026549 | 19 | *LRFN1* | Body | TRUE | Island |
| 4567 | cg16786458 | -0.0057 | 0.02655 | 5 | *PPARGC1B* | TSS1500 | TRUE | N_Shore |
| 4568 | cg17204113 | -0.008 | 0.026571 | 6 | *-* | - | TRUE | - |
| 4569 | cg10134924 | -0.0036 | 0.026577 | 2 | *-* | - | NA | - |
| 4570 | cg26559209 | -0.003 | 0.026585 | 10 | *-* | - | TRUE | N_Shore |
| 4571 | cg14856755 | -0.0038 | 0.026585 | 13 | *-* | - | NA | - |
| 4572 | cg09183450 | -0.0049 | 0.026585 | 11 | *AH-K* | 5'UTR | TRUE | - |
| 4573 | cg17472686 | -0.002 | 0.026594 | 17 | *-* | - | NA | - |
| 4574 | cg02493602 | -0.0117 | 0.026655 | 11 | *ME3* | TSS1500 | TRUE | S_Shore |
| 4575 | cg06675981 | 0.0033 | 0.026658 | 12 | *IQCD* | Body | NA | - |
| 4576 | cg22299535 | -0.0061 | 0.026664 | 18 | *-* | - | NA | - |
| 4577 | cg02766106 | -0.003 | 0.026664 | 9 | *C9orf43* | Body | NA | - |
| 4578 | cg21148642 | -0.002 | 0.026687 | 19 | *SULT2B1* | Body | NA | S_Shelf |
| 4579 | cg09559780 | -0.0032 | 0.026687 | 16 | *-* | - | TRUE | - |
| 4580 | cg19238380 | -0.0033 | 0.026687 | 1 | *LM-* | Body | TRUE | - |
| 4581 | cg19789165 | -9.00E-04 | 0.026687 | 1 | *-* | - | TRUE | Island |
| 4582 | cg05291964 | -0.0027 | 0.026687 | 3 | *CADPS* | Body | NA | - |
| 4583 | cg10956183 | -0.0044 | 0.026762 | 22 | *-* | - | NA | - |
| 4584 | cg04900982 | -0.0023 | 0.026762 | 9 | *-* | - | NA | - |
| 4585 | cg08756033 | 0.005 | 0.026762 | 13 | *C13orf33* | TSS200 | TRUE | N_Shore |
| 4586 | cg11590282 | -0.0034 | 0.026764 | 11 | *-* | - | TRUE | S_Shelf |
| 4587 | cg25460142 | -0.0044 | 0.026776 | 15 | *MEGF11* | Body | NA | - |
| 4588 | cg01533332 | 0.0015 | 0.026788 | 22 | *-* | - | NA | - |
| 4589 | cg19549040 | -0.0018 | 0.026795 | 7 | *-* | - | NA | - |
| 4590 | cg16368147 | -0.0026 | 0.026796 | 4 | *-* | - | TRUE | - |
| 4591 | cg16899991 | -0.0025 | 0.026796 | 3 | *-* | - | TRUE | N_Shelf |
| 4592 | cg02305709 | 0.0022 | 0.026796 | 17 | *NEK8* | Body | NA | - |
| 4593 | cg08088818 | -0.0024 | 0.026796 | 19 | *-* | - | NA | - |
| 4594 | cg16833817 | -0.0029 | 0.026822 | 11 | *CCDC88B* | Body | NA | N_Shelf |
| 4595 | cg25272902 | -0.0028 | 0.026823 | 5 | *CAMK2A* | Body | NA | - |
| 4596 | cg13945718 | -0.0032 | 0.02685 | 10 | *MORN4* | TSS1500 | NA | S_Shore |
| 4597 | cg18080106 | -0.0038 | 0.02686 | 2 | *ADAM23* | Body | NA | - |
| 4598 | cg15604132 | -0.0034 | 0.026862 | 5 | *ZNF300* | TSS1500 | NA | S_Shore |
| 4599 | cg07211259 | -0.0024 | 0.026925 | 9 | *PDCD1LG2* | TSS200 | TRUE | - |
| 4600 | cg00563932 | -0.0029 | 0.026925 | 9 | *PTGDS* | TSS1500 | TRUE | N_Shore |
| 4601 | cg24770595 | -0.0022 | 0.026944 | 6 | *ROS1* | Body | NA | - |
| 4602 | cg12817634 | -0.0016 | 0.026965 | 12 | *CLSTN3* | Body | NA | - |
| 4603 | cg02013841 | -0.0037 | 0.026971 | 13 | *-* | - | TRUE | - |
| 4604 | cg08674349 | -0.0032 | 0.026988 | 6 | *PACRG* | Body | NA | - |
| 4605 | cg20182237 | -0.0051 | 0.026988 | 5 | *IQGAP2* | Body | NA | - |
| 4606 | cg22655507 | 8.00E-04 | 0.027 | 16 | *EXOSC6* | TSS200 | NA | Island |
| 4607 | cg10056203 | -0.0032 | 0.027023 | 10 | *-* | - | TRUE | S_Shore |
| 4608 | cg27535502 | 0.002 | 0.027037 | 15 | *-* | - | TRUE | - |
| 4609 | cg23315423 | -0.0033 | 0.027038 | 10 | *SNCG* | TSS200 | TRUE | - |
| 4610 | cg05719180 | -0.0045 | 0.027038 | 12 | *-* | - | NA | - |
| 4611 | cg04283570 | -0.0025 | 0.027114 | 7 | *-* | - | NA | - |
| 4612 | cg04213679 | -0.0019 | 0.027116 | 5 | *RAI14* | Body | NA | - |
| 4613 | cg07946633 | 0.004 | 0.027151 | 1 | *FLJ42875* | Body | TRUE | Island |
| 4614 | cg01919189 | -0.0028 | 0.027151 | 11 | *TECTA* | Body | NA | - |
| 4615 | cg15353501 | 0.0013 | 0.027171 | 20 | *NKX22* | Body | NA | Island |
| 4616 | cg11842415 | 0.0018 | 0.027175 | 1 | *LHX8* | Body | TRUE | N_Shore |
| 4617 | cg02836987 | -0.0041 | 0.027177 | 10 | *LOC101927964* | TSS1500 | NA | - |
| 4618 | cg21732948 | 0.001 | 0.027177 | 1 | *ZNF697* | TSS200 | NA | Island |
| 4619 | cg16290275 | -0.0035 | 0.027191 | 1 | *-* | - | TRUE | S_Shore |
| 4620 | cg18644227 | -0.0028 | 0.027202 | 1 | *-* | - | NA | - |
| 4621 | cg02254885 | -0.0021 | 0.027202 | 8 | *-* | - | TRUE | N_Shore |
| 4622 | cg26718433 | 0.0016 | 0.027215 | 10 | *CXCL12* | TSS200 | TRUE | Island |
| 4623 | cg13917589 | 0.0021 | 0.027215 | 4 | *PCGF3* | Body | TRUE | S_Shore |
| 4624 | cg24152351 | 0.004 | 0.027239 | 9 | *LHX6* | 5'UTR | NA | Island |
| 4625 | cg26543757 | 0.0019 | 0.027252 | 5 | *-* | - | NA | - |
| 4626 | cg04097078 | 0.0084 | 0.027296 | 22 | *SCARF2* | Body | TRUE | Island |
| 4627 | cg17461571 | 0.0038 | 0.027335 | 19 | *POLD1* | TSS1500 | NA | S_Shore |
| 4628 | cg23922645 | 0.0011 | 0.02736 | 18 | *GACAT2* | Body | NA | Island |
| 4629 | cg20595453 | -0.003 | 0.02736 | 6 | *VPS52* | Body | TRUE | S_Shore |
| 4630 | cg06399965 | 0.005 | 0.02736 | 16 | *-* | - | NA | - |
| 4631 | cg14874742 | -0.0039 | 0.02736 | 5 | *-* | - | TRUE | - |
| 4632 | cg15206000 | -0.0023 | 0.02736 | 13 | *-* | - | NA | - |
| 4633 | cg27549720 | 0.0038 | 0.027395 | 1 | *HTR6* | 5'UTR | TRUE | Island |
| 4634 | cg21296230 | 0.0015 | 0.027401 | 15 | *GREM1* | 5'UTR | TRUE | Island |
| 4635 | cg00598772 | 0.0029 | 0.027413 | 8 | *-* | - | NA | - |
| 4636 | cg12652118 | 0.002 | 0.027436 | 8 | *RHOBTB2* | 5'UTR | NA | S_Shore |
| 4637 | cg03529189 | -0.004 | 0.027436 | 12 | *SRGAP1* | Body | TRUE | - |
| 4638 | cg22242842 | -0.0047 | 0.02745 | 2 | *-* | - | TRUE | - |
| 4639 | cg01669880 | 0.0026 | 0.02745 | 12 | *EP400* | Body | NA | Island |
| 4640 | cg23741006 | -0.003 | 0.027466 | 11 | *TCN1* | TSS200 | TRUE | - |
| 4641 | cg03227963 | 0.0029 | 0.027474 | 5 | *-* | - | TRUE | - |
| 4642 | cg00468146 | 0.0018 | 0.027494 | 6 | *ID4* | 1stExon | TRUE | Island |
| 4643 | cg24127251 | -0.0018 | 0.0275 | 1 | *-* | - | NA | - |
| 4644 | cg10493067 | -0.0018 | 0.027502 | 3 | *XXYLT1* | Body | NA | - |
| 4645 | cg08259169 | 0.0052 | 0.027509 | X | *-* | - | NA | Island |
| 4646 | cg17158002 | -0.0026 | 0.027527 | 15 | *GPR176* | Body | NA | - |
| 4647 | cg11601477 | -0.0044 | 0.027531 | 16 | *-* | - | NA | - |
| 4648 | cg25162533 | 0.0053 | 0.027531 | 17 | *-* | - | TRUE | N_Shore |
| 4649 | cg24154948 | -0.003 | 0.027551 | 12 | *-* | - | NA | - |
| 4650 | cg00448395 | -0.0046 | 0.027551 | 7 | *POT1* | TSS1500 | TRUE | S_Shore |
| 4651 | cg26236972 | -0.005 | 0.027559 | 2 | *-* | - | TRUE | - |
| 4652 | cg01419539 | -0.004 | 0.027587 | 4 | *ZNF595* | TSS1500 | TRUE | N_Shore |
| 4653 | cg08924275 | -0.0019 | 0.027594 | 2 | *-* | - | NA | - |
| 4654 | cg25031151 | -0.0011 | 0.027594 | 5 | *CDH6* | 5'UTR | NA | - |
| 4655 | cg25130357 | -0.003 | 0.027597 | 16 | *TRAP1* | Body | NA | - |
| 4656 | cg02691005 | -0.003 | 0.027642 | 12 | *PDE3A* | Body | NA | S_Shore |
| 4657 | cg26328936 | 0.0026 | 0.027642 | 12 | *ABCD2* | Body | NA | - |
| 4658 | cg05939495 | -0.002 | 0.027653 | 15 | *CYP11A1* | TSS200 | TRUE | S_Shore |
| 4659 | cg01860943 | 0.0026 | 0.027664 | 8 | *-* | - | NA | - |
| 4660 | cg24371584 | -0.003 | 0.027664 | 5 | *-* | - | NA | - |
| 4661 | cg16742481 | -0.0053 | 0.027671 | 4 | *-* | - | TRUE | - |
| 4662 | cg03069943 | 0.0023 | 0.027673 | 17 | *MLLT6* | Body | TRUE | - |
| 4663 | cg13552026 | -0.0024 | 0.027679 | 9 | *-* | - | TRUE | - |
| 4664 | cg13978175 | -0.0036 | 0.027701 | 7 | *IQCE* | Body | TRUE | S_Shelf |
| 4665 | cg13683194 | -0.0017 | 0.027713 | 9 | *C9orf125* | 3'UTR | TRUE | - |
| 4666 | cg18632612 | -0.004 | 0.027713 | 8 | *-* | - | NA | - |
| 4667 | cg14360579 | -0.0034 | 0.027729 | 1 | *-* | - | NA | - |
| 4668 | cg13362823 | 0.0029 | 0.027729 | 3 | *LINC00870* | Body | NA | - |
| 4669 | cg23340957 | 0.0021 | 0.027758 | 2 | *RHBDD1* | Body | NA | - |
| 4670 | cg19571979 | -0.0028 | 0.027765 | 11 | *PLEKHA7* | Body | NA | - |
| 4671 | cg22067300 | -0.0022 | 0.027765 | 1 | *-* | - | NA | - |
| 4672 | cg24022879 | -0.0034 | 0.027766 | 7 | *-* | - | NA | - |
| 4673 | cg00379249 | 0.0019 | 0.027774 | 7 | *-* | - | NA | S_Shelf |
| 4674 | cg05656812 | 0.0023 | 0.027797 | 1 | *DISC1* | Body | TRUE | - |
| 4675 | cg12834820 | -0.0012 | 0.027797 | 17 | *ADAM11* | Body | TRUE | N_Shore |
| 4676 | cg13783238 | 0.0042 | 0.027797 | 3 | *SMC4* | Body | TRUE | S_Shelf |
| 4677 | cg14036830 | 0.0016 | 0.027809 | 19 | *GRIK5* | Body | TRUE | Island |
| 4678 | cg03289416 | -0.0022 | 0.027824 | 15 | *SCAMP2* | TSS1500 | TRUE | S_Shore |
| 4679 | cg00613827 | -0.0031 | 0.027832 | 1 | *CR1L* | Body | NA | S_Shelf |
| 4680 | cg13844402 | 0.001 | 0.027832 | 13 | *-* | - | TRUE | Island |
| 4681 | cg03131366 | -0.0052 | 0.027841 | 12 | *-* | - | TRUE | - |
| 4682 | cg18585103 | -0.0037 | 0.027843 | 14 | *-* | - | NA | - |
| 4683 | cg06348484 | 0.0028 | 0.027852 | 3 | *KLHL6* | Body | NA | - |
| 4684 | cg19514721 | -0.003 | 0.027852 | 5 | *-* | - | TRUE | - |
| 4685 | cg07671819 | -0.0015 | 0.027852 | 12 | *-* | - | NA | - |
| 4686 | cg03954917 | 0.0023 | 0.027852 | 2 | *-* | - | NA | - |
| 4687 | cg11622938 | 0.0022 | 0.027866 | 14 | *REC8* | Body | NA | - |
| 4688 | cg19412808 | 0.0037 | 0.027866 | 1 | *-* | - | TRUE | - |
| 4689 | cg01611017 | -0.0019 | 0.027866 | 14 | *CLMN* | Body | TRUE | - |
| 4690 | cg01346501 | -0.002 | 0.027866 | 17 | *NR1D1* | Body | TRUE | N_Shore |
| 4691 | cg07359386 | 0.0023 | 0.027866 | 5 | *ADCY2* | Body | NA | - |
| 4692 | cg06501000 | 0.0018 | 0.027866 | 6 | *ANKRD6* | Body | NA | - |
| 4693 | cg20413392 | 0.0052 | 0.027895 | 9 | *MIR2192* | TSS200 | TRUE | Island |
| 4694 | cg19341927 | 0.0023 | 0.027901 | 14 | *RIN3* | Body | NA | - |
| 4695 | cg23320649 | -0.0022 | 0.027943 | 3 | *C3orf18* | 5'UTR | TRUE | N_Shore |
| 4696 | cg01150646 | 0.0023 | 0.027945 | 17 | *MYO1C* | Body | NA | S_Shore |
| 4697 | cg06601891 | -0.0097 | 0.027982 | 12 | *MGP* | 1stExon | TRUE | - |
| 4698 | cg12686273 | -0.0028 | 0.027995 | 16 | *CCDC64B* | TSS1500 | TRUE | N_Shore |
| 4699 | cg17045015 | -0.0033 | 0.028002 | 11 | *-* | - | NA | - |
| 4700 | cg04923560 | 0.0013 | 0.028052 | 4 | *HHIP* | 5'UTR | NA | Island |
| 4701 | cg07408494 | -0.0042 | 0.028052 | 5 | *-* | - | NA | - |
| 4702 | cg01348594 | -0.0015 | 0.028052 | 3 | *-* | - | TRUE | N_Shore |
| 4703 | cg12732334 | -0.0053 | 0.02807 | 1 | *EIF4G3* | TSS200 | NA | N_Shore |
| 4704 | cg03301240 | 0.0022 | 0.028075 | 11 | *SPI1* | TSS1500 | TRUE | S_Shore |
| 4705 | cg15301794 | 0.002 | 0.028162 | 11 | *PAX6* | Body | TRUE | S_Shore |
| 4706 | cg25354657 | 0.0023 | 0.028162 | 11 | *APLP2* | Body | TRUE | N_Shelf |
| 4707 | cg00577449 | 0.0035 | 0.028176 | 3 | *-* | - | TRUE | N_Shore |
| 4708 | cg03922471 | 0.003 | 0.028194 | 9 | *-* | - | NA | - |
| 4709 | cg06083669 | 0.0029 | 0.028194 | 20 | *ITPA* | TSS1500 | TRUE | N_Shore |
| 4710 | cg03858365 | -0.0044 | 0.028197 | 13 | *-* | - | NA | - |
| 4711 | cg12446246 | -0.0025 | 0.028217 | 1 | *PLX-2* | TSS1500 | TRUE | S_Shore |
| 4712 | cg01360261 | 0.0023 | 0.028227 | 20 | *FAM65C* | Body | NA | - |
| 4713 | cg11934096 | 0.0025 | 0.028243 | 8 | *EXT1* | Body | NA | - |
| 4714 | cg26987281 | -0.0022 | 0.028246 | 15 | *DAPK2* | Body | NA | - |
| 4715 | cg25595005 | 0.0025 | 0.028294 | 6 | *-* | - | TRUE | - |
| 4716 | cg23135545 | 0.0028 | 0.028294 | 15 | *LRRK1* | Body | NA | - |
| 4717 | cg22266222 | -0.0084 | 0.028294 | 3 | *ITGA9* | Body | NA | S_Shore |
| 4718 | cg08076830 | 0.0017 | 0.028294 | 18 | *-* | - | TRUE | Island |
| 4719 | cg16328494 | -0.0052 | 0.028294 | 5 | *-* | - | NA | - |
| 4720 | cg04219468 | -0.0018 | 0.028294 | 22 | *ZNRF3AS1* | TSS1500 | NA | S_Shore |
| 4721 | cg05728215 | 0.0023 | 0.028305 | 9 | *-* | - | NA | - |
| 4722 | cg18219522 | -0.0047 | 0.028305 | 11 | *MAML2* | Body | TRUE | - |
| 4723 | cg26818820 | -0.0018 | 0.028305 | 5 | *SMAD5* | TSS1500 | TRUE | N_Shore |
| 4724 | cg16737517 | 0.007 | 0.028312 | 20 | *ZBTB46* | Body | TRUE | Island |
| 4725 | cg17215601 | -0.0013 | 0.028325 | 14 | *-* | - | NA | - |
| 4726 | cg20886104 | -0.003 | 0.028325 | 6 | *ENPP1* | Body | NA | - |
| 4727 | cg12951216 | 0.0033 | 0.028343 | 14 | *-* | - | NA | - |
| 4728 | cg14764944 | -0.0037 | 0.028344 | 8 | *-* | - | NA | - |
| 4729 | cg12061886 | 0.0038 | 0.028372 | 14 | *FOS* | Body | TRUE | Island |
| 4730 | cg25103337 | -0.0022 | 0.028372 | 1 | *H6PD* | TSS1500 | TRUE | N_Shore |
| 4731 | cg21415227 | 0.0026 | 0.028385 | 11 | *SPON1* | Body | TRUE | Island |
| 4732 | cg24678767 | 0.0023 | 0.028385 | 15 | *CLK3* | 5'UTR | TRUE | N_Shelf |
| 4733 | cg00223245 | -0.0037 | 0.028385 | 3 | *-* | - | TRUE | - |
| 4734 | cg26789411 | -0.0031 | 0.028407 | 3 | *MECOM* | 5'UTR | NA | - |
| 4735 | cg09648467 | 0.003 | 0.02841 | 5 | *ADAM19* | Body | TRUE | - |
| 4736 | cg18565342 | 0.0018 | 0.028411 | 10 | *C10orf88* | TSS200 | TRUE | Island |
| 4737 | cg18011691 | -0.0033 | 0.028411 | 8 | *PPP2R2A* | Body | TRUE | - |
| 4738 | cg03817107 | -0.0035 | 0.028411 | 5 | *FOXI1* | TSS200 | TRUE | N_Shore |
| 4739 | cg11279021 | 0.0016 | 0.028411 | 7 | *ETV1* | 5'UTR | TRUE | N_Shore |
| 4740 | cg19894966 | 0.004 | 0.028424 | 6 | *LACE1* | Body | NA | - |
| 4741 | cg13495277 | 0.0024 | 0.028442 | 2 | *LOC101929715* | Body | NA | - |
| 4742 | cg21594918 | 0.0021 | 0.028451 | 14 | *DEGS2* | TSS1500 | NA | S_Shore |
| 4743 | cg19680554 | 0.0049 | 0.028494 | 7 | *C7orf43* | Body | NA | N_Shore |
| 4744 | cg16562730 | 0.0016 | 0.028498 | 16 | *CHST4* | 1stExon | TRUE | - |
| 4745 | cg10333806 | 0.0036 | 0.028499 | X | *BCOR* | Body | NA | - |
| 4746 | cg25790232 | -0.0056 | 0.028508 | 16 | *-* | - | NA | - |
| 4747 | cg13203811 | -0.0019 | 0.02854 | 12 | *AGAP2* | TSS1500 | TRUE | S_Shelf |
| 4748 | cg02802514 | 0.0028 | 0.028556 | 6 | *HSD17B8* | Body | TRUE | S_Shore |
| 4749 | cg08473169 | 0.0019 | 0.028568 | 9 | *TTLL11* | Body | NA | - |
| 4750 | cg08949815 | -0.0018 | 0.028568 | 2 | *FAM132B* | 3'UTR | NA | S_Shelf |
| 4751 | cg11290779 | -0.0024 | 0.028641 | 1 | *SOX13* | Body | NA | - |
| 4752 | cg10361922 | 0.0022 | 0.028642 | 17 | *VPS25* | Body | TRUE | - |
| 4753 | cg09195098 | -0.0047 | 0.028649 | 6 | *GCNT2* | Body | TRUE | - |
| 4754 | cg26088888 | -0.0027 | 0.028702 | 12 | *-* | - | NA | - |
| 4755 | cg08902940 | 0.0034 | 0.028763 | 1 | *-* | - | TRUE | - |
| 4756 | cg06221746 | -0.0023 | 0.028783 | 16 | *PAGR1* | 3'UTR | NA | - |
| 4757 | cg03624070 | 0.0021 | 0.028811 | 20 | *LINC01272* | Body | NA | - |
| 4758 | cg16764037 | -0.0019 | 0.028811 | 15 | *-* | - | NA | - |
| 4759 | cg13451703 | -0.0027 | 0.028811 | 2 | *LIMS1* | TSS1500 | NA | - |
| 4760 | cg12172916 | -0.0024 | 0.028811 | 17 | *-* | - | NA | - |
| 4761 | cg18732257 | -0.0026 | 0.028813 | 1 | *-* | - | NA | - |
| 4762 | cg24996282 | 0.0023 | 0.028814 | 15 | *-* | - | TRUE | - |
| 4763 | cg11629812 | -0.0055 | 0.028849 | 5 | *-* | - | TRUE | - |
| 4764 | cg09337174 | -0.003 | 0.02885 | 2 | *-* | - | NA | - |
| 4765 | cg10405023 | 0.0024 | 0.028866 | 12 | *NR4A1* | 5'UTR | NA | S_Shore |
| 4766 | cg06589222 | 0.0038 | 0.028866 | 1 | *CTPS1* | 5'UTR | NA | S_Shore |
| 4767 | cg23197073 | 0.0042 | 0.0289 | 2 | *HDAC4* | Body | NA | - |
| 4768 | cg25880623 | -0.0029 | 0.028915 | 9 | *MVB12B* | Body | NA | - |
| 4769 | cg08828036 | 0.0057 | 0.028934 | 15 | *ATP10A* | TSS1500 | TRUE | S_Shore |
| 4770 | cg11020047 | -0.0054 | 0.028938 | 17 | *-* | - | NA | - |
| 4771 | cg23446587 | 0.0039 | 0.028942 | 15 | *MGA* | TSS1500 | NA | N_Shore |
| 4772 | cg07143259 | 0.0026 | 0.028994 | 22 | *PLXNB2* | 5'UTR | NA | N_Shelf |
| 4773 | cg16746462 | -8.00E-04 | 0.028997 | 5 | *LNPEP* | TSS1500 | TRUE | Island |
| 4774 | cg04982834 | -0.0035 | 0.029008 | 1 | *EVI5* | TSS1500 | TRUE | - |
| 4775 | cg24554140 | -0.0021 | 0.029008 | 3 | *DUSP7* | TSS1500 | NA | S_Shore |
| 4776 | cg02457781 | -0.0018 | 0.029008 | 2 | *ARMC9* | Body | TRUE | N_Shelf |
| 4777 | cg13085338 | 8.00E-04 | 0.029008 | 1 | *CENPF* | TSS200 | TRUE | Island |
| 4778 | cg08823954 | -0.0023 | 0.029008 | 11 | *PLEKHB1* | 5'UTR | TRUE | - |
| 4779 | cg12884169 | 8.00E-04 | 0.029008 | 21 | *ERG* | 5'UTR | TRUE | Island |
| 4780 | cg22015888 | 0.0022 | 0.029023 | 5 | *-* | - | NA | - |
| 4781 | cg17569828 | -0.0028 | 0.029036 | 3 | *CPN2* | 5'UTR | NA | - |
| 4782 | cg15128842 | -0.0038 | 0.029043 | 7 | *CALD1* | Body | NA | - |
| 4783 | cg16763513 | -0.0033 | 0.029058 | 12 | *STX2* | Body | NA | N_Shore |
| 4784 | cg20480663 | -0.0031 | 0.029075 | 15 | *CCDC33* | Body | NA | - |
| 4785 | cg19532307 | 0.0015 | 0.029082 | 3 | *SLITRK3* | 5'UTR | TRUE | - |
| 4786 | cg26191447 | -0.0031 | 0.029114 | 2 | *-* | - | TRUE | - |
| 4787 | cg16687095 | -0.003 | 0.029137 | 7 | *-* | - | NA | - |
| 4788 | cg03779603 | -0.0032 | 0.029137 | 17 | *-* | - | NA | - |
| 4789 | cg09011891 | -0.0027 | 0.029161 | 5 | *-* | - | TRUE | N_Shore |
| 4790 | cg23595403 | -0.0018 | 0.029168 | 6 | *-* | - | NA | - |
| 4791 | cg05055822 | 0.0029 | 0.029189 | 4 | *-* | - | NA | - |
| 4792 | cg13278353 | 0.0038 | 0.029197 | 2 | *-* | - | TRUE | S_Shore |
| 4793 | cg12115681 | 8.00E-04 | 0.029205 | 3 | *PCNP* | TSS1500 | NA | N_Shore |
| 4794 | cg18100950 | -0.0033 | 0.029211 | 2 | *-* | - | NA | - |
| 4795 | cg26477856 | 0.0022 | 0.029239 | 12 | *DGKA* | TSS1500 | TRUE | N_Shore |
| 4796 | cg09906752 | -0.0044 | 0.029239 | 6 | *CYP39A1* | TSS1500 | TRUE | S_Shore |
| 4797 | cg17738521 | -0.0027 | 0.029284 | 6 | *HIVEP2* | 5'UTR | TRUE | - |
| 4798 | cg14532755 | -0.0033 | 0.029313 | 4 | *-* | - | TRUE | N_Shelf |
| 4799 | cg24848615 | -0.0039 | 0.029323 | 19 | *NFIC* | Body | TRUE | N_Shore |
| 4800 | cg08349142 | 0.0015 | 0.029324 | 1 | *-* | - | NA | - |
| 4801 | cg13327052 | -0.004 | 0.029324 | 5 | *LOC101927421* | Body | NA | - |
| 4802 | cg08173973 | 0.0042 | 0.029324 | 20 | *CDS2* | Body | NA | - |
| 4803 | cg01794805 | -0.008 | 0.029324 | 3 | *RARB* | 5'UTR | TRUE | - |
| 4804 | cg09215104 | 0.0051 | 0.029324 | 12 | *SLCO1A2* | 5'UTR | NA | - |
| 4805 | cg03119750 | 0.0024 | 0.029324 | 22 | *GRAP2* | 5'UTR | NA | - |
| 4806 | cg18912768 | -0.0029 | 0.029392 | 2 | *ABCB11* | 5'UTR | TRUE | - |
| 4807 | cg18613324 | -0.0047 | 0.029392 | 22 | *RBM9* | Body | TRUE | - |
| 4808 | cg09106932 | -0.003 | 0.029392 | 5 | *-* | - | TRUE | N_Shelf |
| 4809 | cg04354805 | -0.0011 | 0.029392 | 1 | *ZDHHC18* | TSS1500 | TRUE | N_Shore |
| 4810 | cg21596858 | -0.0028 | 0.029392 | 1 | *DCST1* | Body | TRUE | - |
| 4811 | cg19952260 | -0.0027 | 0.029408 | 11 | *ANO1* | Body | NA | - |
| 4812 | cg15858512 | 0.0025 | 0.029408 | 17 | *CAMTA2* | Body | NA | S_Shore |
| 4813 | cg02497737 | -0.0026 | 0.029435 | 15 | *ADAMTSL3* | Body | NA | - |
| 4814 | cg01047938 | 0.0017 | 0.029442 | 4 | *ARHGEF38* | TSS1500 | NA | - |
| 4815 | cg13199620 | 0.004 | 0.029512 | 17 | *SERPINF1* | Body | NA | - |
| 4816 | cg13227833 | 0.0027 | 0.029517 | 17 | *CEP295NL* | TSS1500 | NA | S_Shelf |
| 4817 | cg15803041 | 0.0013 | 0.02952 | 10 | *FAM13C* | TSS1500 | NA | S_Shore |
| 4818 | cg18822131 | 0.0021 | 0.02952 | 14 | *-* | - | NA | - |
| 4819 | cg17723710 | -0.0046 | 0.02952 | 5 | *-* | - | TRUE | - |
| 4820 | cg02467054 | -7.00E-04 | 0.02952 | 16 | *FAM100A* | 1stExon | TRUE | Island |
| 4821 | cg11032145 | -0.0053 | 0.029532 | 9 | *APBA1* | 3'UTR | NA | - |
| 4822 | cg08502128 | 0.0034 | 0.02955 | 14 | *RIN3* | Body | NA | - |
| 4823 | cg22225886 | -0.0027 | 0.029592 | 8 | *-* | - | TRUE | - |
| 4824 | cg09805010 | 0.0011 | 0.029616 | 3 | *THRB* | 5'UTR | TRUE | Island |
| 4825 | cg07894352 | 0.0061 | 0.029616 | 19 | *ZNF233* | TSS200 | TRUE | Island |
| 4826 | cg08514311 | -0.0025 | 0.029641 | 16 | *-* | - | TRUE | - |
| 4827 | cg14349689 | -0.0025 | 0.029644 | 8 | *LOC101926892* | Body | NA | - |
| 4828 | cg17064907 | 0.0016 | 0.029648 | 3 | *FAM43A* | 1stExon | TRUE | Island |
| 4829 | cg14454522 | 0.0015 | 0.029672 | 4 | *-* | - | NA | - |
| 4830 | cg01781081 | 0.0018 | 0.029672 | 20 | *-* | - | NA | - |
| 4831 | cg23500537 | 0.0023 | 0.029702 | 5 | *-* | - | TRUE | - |
| 4832 | cg18437808 | -0.0031 | 0.029703 | 17 | *C17orf62* | Body | TRUE | Island |
| 4833 | cg14562930 | 0.0021 | 0.029764 | 11 | *FADS2* | TSS1500 | NA | Island |
| 4834 | cg22325145 | -0.002 | 0.029766 | 22 | *KCNJ4* | 5'UTR | TRUE | S_Shore |
| 4835 | cg09365147 | -0.0032 | 0.029783 | 6 | *REV3L* | Body | TRUE | - |
| 4836 | cg01291665 | 0.0054 | 0.029789 | 10 | *C10orf47* | Body | TRUE | N_Shore |
| 4837 | cg06777568 | 0.0014 | 0.029789 | 7 | *-* | - | NA | - |
| 4838 | cg03039990 | -0.004 | 0.029798 | 14 | *MIR770* | TSS1500 | TRUE | - |
| 4839 | cg18628483 | -0.0029 | 0.029825 | 12 | *KIF5A* | TSS1500 | TRUE | N_Shore |
| 4840 | cg09959687 | -0.0077 | 0.029831 | 6 | *-* | - | NA | - |
| 4841 | cg05691158 | 0.0038 | 0.029863 | 12 | *-* | - | TRUE | - |
| 4842 | cg01568519 | 0.002 | 0.029863 | 9 | *TRPM3* | Body | NA | - |
| 4843 | cg26599122 | -0.0025 | 0.029863 | 1 | *UBXN10AS1* | TSS1500 | NA | S_Shore |
| 4844 | cg24616450 | -0.0046 | 0.029863 | 1 | *MINOS1* | Body | NA | - |
| 4845 | cg03746976 | -0.0022 | 0.029889 | 16 | *C16orf57* | Body | TRUE | S_Shore |
| 4846 | cg05630213 | -0.0016 | 0.029903 | 17 | *LRRC3C* | Body | NA | - |
| 4847 | cg23733857 | -0.003 | 0.029916 | 1 | *MARK1* | 3'UTR | NA | - |
| 4848 | cg10620680 | -0.0024 | 0.029916 | 1 | *-* | - | TRUE | - |
| 4849 | cg23774365 | -0.0034 | 0.029916 | 19 | *-* | - | NA | - |
| 4850 | cg18693069 | 0.0023 | 0.029917 | 12 | *DTX1* | Body | NA | - |
| 4851 | cg26372160 | 0.0029 | 0.02992 | 8 | *-* | - | NA | - |
| 4852 | cg11569198 | 9.00E-04 | 0.029922 | 4 | *RBPJ* | TSS1500 | NA | N_Shore |
| 4853 | cg24635075 | -0.0038 | 0.029937 | 8 | *RAD54B* | Body | NA | - |
| 4854 | cg01357135 | -0.0029 | 0.029959 | 3 | *PPM1L* | Body | TRUE | S_Shore |
| 4855 | cg22021226 | -0.0024 | 0.029989 | 8 | *HGS-T* | Body | NA | - |
| 4856 | cg20449726 | -0.0016 | 0.029989 | 1 | *KIAA1026* | Body | TRUE | - |
| 4857 | cg00962793 | 0.0022 | 0.029995 | 14 | *-* | - | TRUE | - |
| 4858 | cg20327730 | 0.002 | 0.030017 | 10 | *-* | - | NA | - |
| 4859 | cg18637203 | -0.0037 | 0.030071 | 18 | *L3MBTL4* | Body | NA | - |
| 4860 | cg24382249 | -0.0132 | 0.030103 | 15 | *-* | - | TRUE | N_Shore |
| 4861 | cg26932379 | -0.0024 | 0.030103 | 16 | *-* | - | NA | - |
| 4862 | cg18181703 | 0.0037 | 0.03012 | 17 | *SOCS3* | Body | TRUE | N_Shore |
| 4863 | cg08207604 | -0.0015 | 0.030127 | 6 | *DEF6* | TSS1500 | TRUE | - |
| 4864 | cg12091331 | -0.0045 | 0.030129 | 8 | *PLAT* | TSS200 | TRUE | - |
| 4865 | cg10517328 | 0.0023 | 0.030137 | 11 | *-* | - | NA | - |
| 4866 | cg27189822 | -0.0019 | 0.03014 | 11 | *CD44* | Body | NA | - |
| 4867 | cg13284921 | -0.0016 | 0.030185 | 11 | *CD44* | TSS200 | NA | N_Shore |
| 4868 | cg10246446 | -0.0028 | 0.030197 | 5 | *-* | - | NA | - |
| 4869 | cg01606885 | -0.0029 | 0.030222 | 5 | *-* | - | TRUE | - |
| 4870 | cg00383006 | -0.0018 | 0.03023 | 17 | *CFAP52* | Body | NA | - |
| 4871 | cg26022214 | 0.0011 | 0.030241 | 5 | *EBF1* | Body | NA | N_Shore |
| 4872 | cg03416035 | -0.0019 | 0.030241 | 19 | *C5AR2* | TSS200 | NA | - |
| 4873 | cg11580759 | 0.0019 | 0.030271 | 11 | *LOC100130987* | Body | NA | - |
| 4874 | cg05135851 | 0.0024 | 0.030271 | 3 | *EXOSC7* | Body | NA | - |
| 4875 | cg08126665 | -0.0027 | 0.030285 | 5 | *-* | - | NA | - |
| 4876 | cg15069849 | -0.0036 | 0.030305 | 12 | *NUAK1* | Body | NA | - |
| 4877 | cg19952829 | -0.0018 | 0.030309 | 9 | *-* | - | NA | - |
| 4878 | cg26781511 | -0.0034 | 0.03032 | 6 | *-* | - | NA | - |
| 4879 | cg11580704 | 0.0038 | 0.030352 | 9 | *LHX3* | Body | TRUE | Island |
| 4880 | cg23105827 | -0.002 | 0.030368 | 1 | *ARHGEF19* | TSS200 | TRUE | - |
| 4881 | cg24931632 | 0.0017 | 0.030378 | 1 | *MGC12982* | Body | TRUE | Island |
| 4882 | cg10714501 | -0.0025 | 0.030387 | 14 | *DCAF5* | Body | NA | - |
| 4883 | cg08498585 | -0.0023 | 0.03039 | 19 | *-* | - | TRUE | N_Shelf |
| 4884 | cg06770790 | 0.004 | 0.03039 | 6 | *-* | - | TRUE | S_Shelf |
| 4885 | cg01558960 | -9.00E-04 | 0.0304 | 16 | *-* | - | TRUE | N_Shelf |
| 4886 | cg22378341 | -0.0038 | 0.030401 | 3 | *-* | - | NA | - |
| 4887 | cg25744957 | -0.0025 | 0.030423 | 4 | *FLJ36777* | TSS1500 | TRUE | - |
| 4888 | cg24724506 | 0.0031 | 0.030423 | 19 | *ISOC2* | 3'UTR | TRUE | S_Shore |
| 4889 | cg03853945 | -0.0035 | 0.030425 | 13 | *-* | - | TRUE | - |
| 4890 | cg09613281 | -0.002 | 0.030436 | 2 | *-* | - | TRUE | - |
| 4891 | cg19219106 | 0.004 | 0.030436 | 13 | *LRCH1* | Body | NA | - |
| 4892 | cg08735279 | 0.002 | 0.030453 | 12 | *ULK1* | Body | TRUE | N_Shelf |
| 4893 | cg17440248 | -0.0022 | 0.030511 | 1 | *CR1L* | Body | TRUE | N_Shore |
| 4894 | cg24350475 | -0.0029 | 0.030539 | 10 | *KIAA1462* | Body | TRUE | - |
| 4895 | cg14817951 | -0.0041 | 0.030543 | X | *-* | - | NA | - |
| 4896 | cg23111990 | 0.0018 | 0.030543 | 13 | *TM9SF2* | Body | NA | - |
| 4897 | cg22976533 | -0.0058 | 0.030543 | 14 | *PACS2* | Body | TRUE | S_Shelf |
| 4898 | cg16822208 | 0.0026 | 0.030545 | 6 | *-* | - | NA | - |
| 4899 | cg26692804 | 0.003 | 0.030545 | 2 | *-* | - | NA | - |
| 4900 | cg06729997 | -0.002 | 0.030559 | 2 | *FHL2* | 5'UTR | NA | - |
| 4901 | cg19912660 | 0.0014 | 0.030572 | 6 | *-* | - | NA | - |
| 4902 | cg02121447 | -0.0027 | 0.030572 | 20 | *SNX21* | TSS1500 | TRUE | N_Shore |
| 4903 | cg21186098 | -0.004 | 0.030572 | 8 | *-* | - | TRUE | - |
| 4904 | cg05743112 | -0.0031 | 0.030572 | 17 | *WBP2* | Body | NA | N_Shore |
| 4905 | cg08839697 | 0.0043 | 0.03058 | 3 | *FRG2C* | TSS1500 | TRUE | - |
| 4906 | cg25389463 | -0.0023 | 0.030589 | 1 | *RBM34* | TSS1500 | TRUE | S_Shore |
| 4907 | cg09877009 | -0.0034 | 0.030624 | 10 | *PRKCQ* | 5'UTR | TRUE | - |
| 4908 | cg10007045 | -0.0021 | 0.030624 | 1 | *UCK2* | TSS1500 | NA | N_Shore |
| 4909 | cg05886087 | -0.0032 | 0.03065 | 2 | *-* | - | TRUE | N_Shore |
| 4910 | cg17975423 | -0.0061 | 0.030659 | 4 | *-* | - | NA | - |
| 4911 | cg04152466 | -0.0035 | 0.030701 | 6 | *LOC101930010* | Body | NA | - |
| 4912 | cg18248586 | -0.0039 | 0.030701 | 11 | *DRD2* | 5'UTR | TRUE | - |
| 4913 | cg09313461 | 0.002 | 0.030701 | 11 | *-* | - | NA | - |
| 4914 | cg26906670 | -0.0028 | 0.030701 | 21 | *-* | - | NA | - |
| 4915 | cg13851904 | 0.0022 | 0.030701 | 1 | *MGC12982* | Body | TRUE | Island |
| 4916 | cg18114755 | -0.0026 | 0.030706 | 8 | *-* | - | NA | - |
| 4917 | cg10514830 | -0.0019 | 0.030713 | 1 | *ALPL* | Body | NA | - |
| 4918 | cg05256719 | -0.0021 | 0.030715 | 6 | *-* | - | TRUE | - |
| 4919 | cg12904135 | -0.0044 | 0.030715 | 2 | *-* | - | TRUE | N_Shore |
| 4920 | cg26107250 | -0.003 | 0.030745 | 1 | *-* | - | NA | - |
| 4921 | cg27640316 | -0.0032 | 0.030745 | 2 | *-* | - | TRUE | - |
| 4922 | cg01068621 | -0.0027 | 0.030745 | 1 | *-* | - | TRUE | - |
| 4923 | cg17994679 | 0.0048 | 0.030745 | 7 | *EEPD1* | Body | NA | - |
| 4924 | cg03420907 | -0.0102 | 0.030745 | 16 | *MGRN1* | Body | TRUE | - |
| 4925 | cg20001658 | 0.0034 | 0.030778 | 12 | *-* | - | NA | - |
| 4926 | cg11570271 | 0.0034 | 0.030821 | 22 | *CECR3* | TSS200 | NA | - |
| 4927 | cg12494143 | 0.0034 | 0.030854 | 20 | *SMOX* | 5'UTR | NA | - |
| 4928 | cg25220748 | 0.0014 | 0.03088 | 20 | *CYP24A1* | 1stExon | NA | Island |
| 4929 | cg01066175 | -0.0031 | 0.030896 | 2 | *AGPS* | Body | NA | - |
| 4930 | cg18907109 | -0.0017 | 0.030944 | 7 | *HEPACAM2* | TSS1500 | NA | - |
| 4931 | cg06957155 | -0.0034 | 0.030944 | 9 | *-* | - | NA | - |
| 4932 | cg23309670 | -0.0029 | 0.030951 | 6 | *CPNE5* | Body | TRUE | - |
| 4933 | cg04261087 | 0.0011 | 0.030951 | 18 | *CABLES1* | 1stExon | NA | Island |
| 4934 | cg21580016 | 0.0018 | 0.030951 | 12 | *KDM2B* | Body | TRUE | N_Shelf |
| 4935 | cg08279217 | -0.0039 | 0.030951 | 12 | *-* | - | NA | - |
| 4936 | cg25522867 | 0.0025 | 0.030951 | 11 | *ABTB2* | Body | TRUE | - |
| 4937 | cg21953769 | 0.0019 | 0.030951 | 19 | *-* | - | NA | S_Shelf |
| 4938 | cg08458745 | 0.002 | 0.03096 | 11 | *ETS1* | Body | TRUE | S_Shelf |
| 4939 | cg16284589 | 0.0018 | 0.03098 | 12 | *KDM2B* | Body | NA | - |
| 4940 | cg23670190 | 0.0028 | 0.03098 | 8 | *FGF17* | TSS1500 | NA | - |
| 4941 | cg04478095 | 0.0015 | 0.03098 | 6 | *-* | - | NA | Island |
| 4942 | cg23595516 | -0.0023 | 0.03098 | 11 | *KIRREL3* | Body | NA | - |
| 4943 | cg24349631 | -0.0082 | 0.03098 | 22 | *C22orf26* | 1stExon | TRUE | Island |
| 4944 | cg14646311 | 0.0044 | 0.031044 | 20 | *ZMYND8* | 5'UTR | NA | - |
| 4945 | cg18468844 | -0.0026 | 0.03106 | 1 | *PTAFR* | TSS1500 | TRUE | - |
| 4946 | cg20854275 | -0.0025 | 0.03106 | 21 | *C21orf58* | TSS1500 | NA | N_Shore |
| 4947 | cg15845673 | -0.0057 | 0.031069 | 12 | *BCAT1* | Body | TRUE | N_Shelf |
| 4948 | cg23174662 | 0.0026 | 0.031069 | 14 | *HIF1A* | Body | TRUE | S_Shelf |
| 4949 | cg19453093 | -0.0026 | 0.031069 | 14 | *KCNK10* | Body | TRUE | - |
| 4950 | cg10830713 | 0.0011 | 0.031151 | 1 | *USP1* | 5'UTR | TRUE | S_Shore |
| 4951 | cg16839129 | -0.0024 | 0.031155 | 17 | *RBFOX3* | 5'UTR | NA | - |
| 4952 | cg10249542 | 0.0033 | 0.031247 | 7 | *-* | - | TRUE | - |
| 4953 | cg25674102 | -0.003 | 0.031247 | 10 | *KIAA1217* | Body | NA | - |
| 4954 | cg13575542 | -0.0025 | 0.031247 | 3 | *-* | - | TRUE | N_Shore |
| 4955 | cg22956254 | -0.002 | 0.03132 | 12 | *GDF3* | TSS1500 | TRUE | - |
| 4956 | cg18031326 | 0.0053 | 0.031322 | 11 | *LRP5* | TSS1500 | TRUE | N_Shore |
| 4957 | cg11190915 | 0.0017 | 0.031322 | 15 | *-* | - | NA | - |
| 4958 | cg18217031 | 0.0019 | 0.031322 | 11 | *BCL9L* | Body | NA | N_Shore |
| 4959 | cg23442766 | -0.0022 | 0.031398 | 14 | *TEP1* | TSS1500 | NA | S_Shore |
| 4960 | cg02450064 | 0.0035 | 0.031402 | 17 | *DHX58* | Body | TRUE | S_Shore |
| 4961 | cg12987318 | -0.0027 | 0.031451 | 6 | *RAET1EAS1* | Body | NA | - |
| 4962 | cg13732465 | -0.003 | 0.031451 | 9 | *-* | - | TRUE | N_Shore |
| 4963 | cg05623986 | 0.004 | 0.031515 | 6 | *-* | - | NA | - |
| 4964 | cg04868238 | -0.0021 | 0.031515 | 8 | *FLJ14107* | TSS1500 | TRUE | - |
| 4965 | cg08844745 | -0.0042 | 0.031525 | 19 | *FBXO17* | TSS1500 | NA | S_Shore |
| 4966 | cg05954280 | -0.0033 | 0.031571 | 6 | *UST* | Body | NA | N_Shelf |
| 4967 | cg13713821 | 0.0053 | 0.031571 | 9 | *-* | - | TRUE | S_Shelf |
| 4968 | cg00075597 | -0.0023 | 0.031577 | 16 | *-* | - | NA | - |
| 4969 | cg25395188 | -0.0023 | 0.031583 | 11 | *VPS37C* | 3'UTR | TRUE | - |
| 4970 | cg15268832 | -0.0036 | 0.031585 | 14 | *CGRRF1* | TSS1500 | NA | N_Shore |
| 4971 | cg24094469 | -0.0034 | 0.031585 | 8 | *-* | - | NA | - |
| 4972 | cg02727835 | 0.0026 | 0.031629 | 11 | *PEX16* | TSS1500 | NA | S_Shore |
| 4973 | cg11602784 | -0.0021 | 0.031648 | 3 | *-* | - | NA | - |
| 4974 | cg15143202 | -0.0057 | 0.031649 | 3 | *MECOM* | TSS1500 | NA | N_Shore |
| 4975 | cg04039397 | -0.0058 | 0.031699 | 3 | *CD96* | TSS200 | TRUE | - |
| 4976 | cg10421346 | -0.0041 | 0.031706 | 2 | *-* | - | NA | - |
| 4977 | cg04918831 | 0.0024 | 0.03176 | 1 | *CHML* | 3'UTR | TRUE | - |
| 4978 | cg08316204 | -0.0029 | 0.03176 | 20 | *SRC* | TSS1500 | NA | N_Shore |
| 4979 | cg07346516 | 0.0024 | 0.031784 | 17 | *9-Sep* | Body | NA | - |
| 4980 | cg09693106 | -0.0095 | 0.031784 | 22 | *C22orf26* | 1stExon | TRUE | Island |
| 4981 | cg18108335 | -0.0031 | 0.031784 | 9 | *SYK* | Body | NA | - |
| 4982 | cg01550554 | -0.0026 | 0.031795 | 13 | *-* | - | NA | - |
| 4983 | cg18954541 | 0.0027 | 0.031802 | 20 | *SLC13A3* | 1stExon | TRUE | Island |
| 4984 | cg00804517 | -0.0022 | 0.03181 | 3 | *CAC-1D* | Body | NA | - |
| 4985 | cg07591270 | -0.0029 | 0.03181 | 7 | *MGAM* | Body | NA | - |
| 4986 | cg24411778 | -0.0033 | 0.031824 | 5 | *PDE4D* | 5'UTR | NA | - |
| 4987 | cg16168656 | 0.0036 | 0.031824 | 10 | *KNDC1* | Body | NA | S_Shelf |
| 4988 | cg15587829 | -0.0024 | 0.031824 | 17 | *TSR1* | TSS1500 | NA | S_Shore |
| 4989 | cg02250171 | -0.0062 | 0.031827 | 12 | *GLIPR1L2* | TSS1500 | NA | N_Shore |
| 4990 | cg07569994 | -0.0018 | 0.031827 | 1 | *-* | - | TRUE | - |
| 4991 | cg06615123 | -0.0028 | 0.031827 | 12 | *VWF* | Body | NA | - |
| 4992 | cg05429117 | -0.0044 | 0.031862 | 10 | *-* | - | TRUE | N_Shelf |
| 4993 | cg17651247 | -0.0018 | 0.031872 | 16 | *TMEM204* | TSS1500 | NA | S_Shelf |
| 4994 | cg04844693 | 0.0021 | 0.031904 | 1 | *EPB41* | 5'UTR | TRUE | N_Shore |
| 4995 | cg21263455 | -0.0035 | 0.031923 | 3 | *-* | - | NA | - |
| 4996 | cg12351358 | 0.0029 | 0.031936 | 14 | *YLPM1* | 3'UTR | NA | - |
| 4997 | cg02881300 | 0.0019 | 0.031937 | 9 | *-* | - | NA | - |
| 4998 | cg03859028 | -0.0025 | 0.031937 | 15 | *-* | - | TRUE | - |
| 4999 | cg03722970 | -0.0052 | 0.031945 | 5 | *-* | - | NA | - |
| 5000 | cg00580965 | 0.0037 | 0.031967 | 8 | *-* | - | TRUE | Island |
| 5001 | cg14201462 | 0.0039 | 0.031967 | 10 | *-* | - | NA | - |
| 5002 | cg09134593 | 0.0013 | 0.031967 | 11 | *ADAMTS8* | 1stExon | TRUE | Island |
| 5003 | cg13370452 | 0.0019 | 0.031967 | 17 | *SLC43A2* | Body | NA | N_Shore |
| 5004 | cg06678722 | -0.0048 | 0.031967 | 2 | *-* | - | NA | - |
| 5005 | cg01331772 | 0.0122 | 0.031967 | 2 | *-* | - | TRUE | - |
| 5006 | cg09321307 | -0.0023 | 0.031967 | 16 | *-* | - | TRUE | N_Shelf |
| 5007 | cg21055197 | -0.0026 | 0.031974 | 3 | *TMPRSS7* | Body | NA | - |
| 5008 | cg03352106 | 0.0046 | 0.031977 | 12 | *KCNJ8* | Body | TRUE | - |
| 5009 | cg26514191 | -0.0024 | 0.031981 | 3 | *LIMD1* | Body | NA | - |
| 5010 | cg18312429 | 0.0035 | 0.031988 | 13 | *SLC10A2* | 1stExon | TRUE | - |
| 5011 | cg11346946 | -0.0028 | 0.031988 | 22 | *-* | - | NA | - |
| 5012 | cg06777902 | -0.0063 | 0.031988 | 10 | *FANK1* | Body | TRUE | - |
| 5013 | cg04084749 | -0.0038 | 0.032005 | 2 | *-* | - | NA | - |
| 5014 | cg13434274 | -0.0025 | 0.03201 | 14 | *-* | - | NA | - |
| 5015 | cg16726026 | 0.0036 | 0.03201 | 1 | *-* | - | NA | - |
| 5016 | cg25787886 | 0.0011 | 0.03201 | 22 | *APOBEC3B* | TSS200 | TRUE | - |
| 5017 | cg21555783 | 0.0029 | 0.03201 | 3 | *-* | - | NA | - |
| 5018 | cg14772615 | -0.0045 | 0.03201 | 6 | *-* | - | TRUE | - |
| 5019 | cg07013148 | 0.0061 | 0.032017 | 8 | *-* | - | TRUE | - |
| 5020 | cg06438724 | -0.0031 | 0.032025 | 15 | *CEP152* | Body | NA | - |
| 5021 | cg05495344 | -0.0047 | 0.032036 | 17 | *TNK1* | TSS1500 | NA | N_Shore |
| 5022 | cg21584396 | 0.0025 | 0.032087 | 11 | *-* | - | NA | - |
| 5023 | cg04509100 | -0.002 | 0.032095 | 16 | *IL34* | TSS200 | TRUE | - |
| 5024 | cg14429033 | -0.0027 | 0.032133 | 22 | *MYO18B* | Body | NA | - |
| 5025 | cg20571136 | 0.0033 | 0.032139 | 11 | *KCNQ1* | TSS200 | NA | - |
| 5026 | cg12500503 | -0.0026 | 0.032139 | 1 | *-* | - | NA | - |
| 5027 | cg09552402 | -0.0046 | 0.032194 | 20 | *STMN3* | Body | TRUE | N_Shore |
| 5028 | cg19795292 | -0.0037 | 0.0323 | 2 | *IL1RL1* | 5'UTR | TRUE | - |
| 5029 | cg22128197 | -0.003 | 0.0323 | 1 | *UBE2Q1* | Body | TRUE | N_Shore |
| 5030 | cg16849075 | -0.0058 | 0.032305 | 7 | *GSAP* | Body | NA | - |
| 5031 | cg10668363 | -0.0027 | 0.032322 | 19 | *ZNF175* | Body | TRUE | S_Shelf |
| 5032 | cg17645919 | 0.0042 | 0.03237 | 6 | *SLC35F1* | Body | NA | N_Shore |
| 5033 | cg12675417 | 0.0025 | 0.03237 | 11 | *ENDOD1* | Body | TRUE | S_Shore |
| 5034 | cg15197679 | -0.0025 | 0.03237 | 6 | *-* | - | NA | - |
| 5035 | cg12784701 | -0.0029 | 0.032391 | 5 | *MAST4* | Body | NA | - |
| 5036 | cg26839356 | -0.0033 | 0.032399 | 15 | *-* | - | TRUE | S_Shore |
| 5037 | cg15994579 | 0.0014 | 0.032429 | 3 | *-* | - | NA | - |
| 5038 | cg16734637 | -0.0029 | 0.032454 | 3 | *FOXP1* | Body | NA | - |
| 5039 | cg02483204 | -0.0039 | 0.032454 | 12 | *-* | - | TRUE | - |
| 5040 | cg08729686 | 0.0028 | 0.032471 | 15 | *IGF1R* | Body | TRUE | S_Shore |
| 5041 | cg10576139 | 0.0051 | 0.032475 | 19 | *NFIC* | Body | NA | S_Shore |
| 5042 | cg16406967 | 0.0011 | 0.032475 | 7 | *HOXA3* | 5'UTR | TRUE | Island |
| 5043 | cg26248284 | 0.0015 | 0.032512 | 4 | *LIMCH1* | 1stExon | TRUE | Island |
| 5044 | cg08764872 | -0.0024 | 0.032512 | 14 | *-* | - | NA | - |
| 5045 | cg11629213 | -0.0026 | 0.032529 | 3 | *GLYCTK* | TSS1500 | NA | N_Shore |
| 5046 | cg11512009 | -0.0038 | 0.032546 | 17 | *THRA* | 5'UTR | TRUE | S_Shore |
| 5047 | cg10812644 | -0.0026 | 0.032569 | 8 | *KCNQ3* | Body | NA | - |
| 5048 | cg23488804 | 0.0014 | 0.032582 | 17 | *RUNDC3A* | Body | TRUE | S_Shore |
| 5049 | cg14188862 | -0.0025 | 0.032586 | 9 | *FAM73B* | 5'UTR | TRUE | S_Shore |
| 5050 | cg06874016 | -0.002 | 0.032587 | 17 | *NKIRAS2* | 3'UTR | TRUE | - |
| 5051 | cg13293618 | -0.0037 | 0.032643 | 11 | *-* | - | NA | Island |
| 5052 | cg03566671 | -0.0041 | 0.03265 | 21 | *LOC100506403* | Body | NA | - |
| 5053 | cg02127509 | 0.002 | 0.03265 | 6 | *MYB* | Body | TRUE | - |
| 5054 | cg19334176 | -0.0022 | 0.032698 | 10 | *DIP2C* | Body | TRUE | N_Shore |
| 5055 | cg15551915 | 0.0016 | 0.032842 | 1 | *BCAR3* | 5'UTR | NA | - |
| 5056 | cg08046466 | 0.0022 | 0.032852 | 16 | *ANKRD11* | Body | NA | - |
| 5057 | cg05667097 | -0.0043 | 0.032861 | 6 | *C6orf25* | Body | TRUE | Island |
| 5058 | cg06825661 | -0.0025 | 0.032861 | 18 | *C18orf1* | 5'UTR | TRUE | N_Shore |
| 5059 | cg27307087 | 0.0069 | 0.032871 | 6 | *HTR1B* | TSS1500 | NA | S_Shore |
| 5060 | cg04496811 | -0.0036 | 0.032941 | 3 | *-* | - | NA | - |
| 5061 | cg23006040 | -0.0029 | 0.032941 | 4 | *-* | - | TRUE | - |
| 5062 | cg24077465 | -0.0027 | 0.032941 | 5 | *GALNT10* | Body | NA | - |
| 5063 | cg08983814 | -0.005 | 0.032941 | 17 | *LINC01483* | Body | NA | - |
| 5064 | cg17215278 | -0.0025 | 0.032941 | 1 | *CD58* | TSS1500 | TRUE | S_Shore |
| 5065 | cg00077872 | 0.0026 | 0.032952 | 12 | *-* | - | NA | - |
| 5066 | cg26395601 | -0.001 | 0.033038 | 22 | *THOC5* | 5'UTR | NA | Island |
| 5067 | cg16139228 | -0.0032 | 0.033046 | 20 | *PIGU* | TSS1500 | NA | S_Shore |
| 5068 | cg07697321 | 0.0023 | 0.03305 | 17 | *OTOP2* | Body | TRUE | S_Shore |
| 5069 | cg06933525 | 0.0026 | 0.033064 | 13 | *R-SEH2BAS1* | TSS1500 | NA | S_Shore |
| 5070 | cg15954235 | -0.0021 | 0.033074 | 6 | *SPDEF* | 5'UTR | TRUE | S_Shelf |
| 5071 | cg22642485 | 0.0011 | 0.033081 | 5 | *-* | - | TRUE | Island |
| 5072 | cg14485097 | -0.0038 | 0.033115 | 7 | *-* | - | TRUE | - |
| 5073 | cg02308718 | -0.0039 | 0.033129 | 14 | *PCNX* | Body | NA | - |
| 5074 | cg05270224 | -0.0038 | 0.033129 | 1 | *-* | - | TRUE | - |
| 5075 | cg14063772 | -0.0039 | 0.033129 | 4 | *-* | - | NA | - |
| 5076 | cg02066364 | -0.0024 | 0.03313 | 1 | *-* | - | NA | - |
| 5077 | cg10982824 | -0.0021 | 0.033135 | 17 | *RTN4RL1* | Body | NA | - |
| 5078 | cg18953068 | -0.0018 | 0.033159 | 19 | *-* | - | NA | - |
| 5079 | cg01508380 | -0.0044 | 0.033159 | 14 | *MMP14* | TSS1500 | TRUE | N_Shore |
| 5080 | cg04462378 | -0.0035 | 0.033159 | 11 | *NUMA1* | 5'UTR | TRUE | - |
| 5081 | cg22487516 | -0.0036 | 0.033159 | 1 | *-* | - | NA | - |
| 5082 | cg18543366 | -0.0024 | 0.033159 | 11 | *-* | - | NA | - |
| 5083 | cg02859129 | 0.0037 | 0.033168 | 16 | *IRX6* | TSS1500 | TRUE | N_Shore |
| 5084 | cg05504719 | -0.0024 | 0.033171 | 10 | *NRP1* | Body | NA | - |
| 5085 | cg00343092 | -0.0039 | 0.033176 | 22 | *TSPO* | 5'UTR | TRUE | Island |
| 5086 | cg15053869 | -0.0021 | 0.03321 | 18 | *CXXC1* | TSS1500 | NA | S_Shore |
| 5087 | cg15281614 | -0.0027 | 0.03321 | 1 | *-* | - | NA | - |
| 5088 | cg03207917 | -0.0041 | 0.033231 | 2 | *-* | - | NA | - |
| 5089 | cg24311507 | 0.0025 | 0.03327 | 17 | *-* | - | NA | - |
| 5090 | cg07432111 | -0.0029 | 0.03327 | 17 | *-* | - | TRUE | - |
| 5091 | cg18396403 | -0.0022 | 0.03327 | 22 | *TMEM184B* | 3'UTR | TRUE | - |
| 5092 | cg12373600 | 0.0022 | 0.03327 | 12 | *ZNF384* | TSS1500 | NA | S_Shore |
| 5093 | cg04793813 | 0.0024 | 0.03327 | 16 | *MT1E* | Body | TRUE | S_Shore |
| 5094 | cg11597131 | 0.0025 | 0.03327 | 2 | *HOXD9* | 3'UTR | TRUE | N_Shore |
| 5095 | cg02931540 | -0.002 | 0.03327 | 2 | *-* | - | NA | - |
| 5096 | cg03016496 | -0.0023 | 0.03327 | 7 | *-* | - | TRUE | - |
| 5097 | cg14096249 | -0.0048 | 0.033288 | 11 | *-* | - | NA | - |
| 5098 | cg20542798 | -0.0028 | 0.033309 | 4 | *MFAP3L* | 5'UTR | NA | - |
| 5099 | cg05467090 | -0.0043 | 0.033342 | 4 | *-* | - | NA | - |
| 5100 | cg14436379 | -0.0042 | 0.033342 | 3 | *CASR* | 5'UTR | NA | - |
| 5101 | cg20974826 | -0.0019 | 0.033348 | 6 | *COL11A2* | Body | TRUE | - |
| 5102 | cg25502475 | -0.0034 | 0.033348 | 2 | *PAIP2B* | 5'UTR | NA | - |
| 5103 | cg20513062 | -0.0031 | 0.03337 | 10 | *SORCS3* | Body | NA | - |
| 5104 | cg00377239 | 0.0018 | 0.033418 | 16 | *ANKRD11* | Body | NA | - |
| 5105 | cg00653615 | -0.0016 | 0.033418 | 1 | *CDC42SE1* | 5'UTR | TRUE | N_Shelf |
| 5106 | cg21282629 | -0.0026 | 0.033418 | 12 | *-* | - | NA | - |
| 5107 | cg08536507 | -0.0019 | 0.033429 | 18 | *-* | - | NA | N_Shelf |
| 5108 | cg21593001 | -0.0023 | 0.03346 | 12 | *DTX1* | Body | TRUE | Island |
| 5109 | cg21800232 | 0.0014 | 0.03346 | 5 | *ANKRD34B* | TSS200 | TRUE | Island |
| 5110 | cg23607810 | 0.0022 | 0.033483 | 1 | *MAN1C1* | Body | NA | - |
| 5111 | cg20422722 | -0.0029 | 0.033484 | 8 | *MAPK15* | Body | TRUE | S_Shelf |
| 5112 | cg16767506 | 0.0082 | 0.033497 | 7 | *-* | - | TRUE | N_Shore |
| 5113 | cg07869023 | -0.0032 | 0.033505 | 20 | *PCSK2* | Body | TRUE | S_Shelf |
| 5114 | cg12959691 | -0.0035 | 0.03352 | 4 | *BMPR1B* | 5'UTR | NA | - |
| 5115 | cg02867869 | 0.0053 | 0.033521 | 13 | *SLC15A1* | Body | NA | - |
| 5116 | cg21658129 | -0.0028 | 0.033521 | 6 | *CD83* | TSS1500 | NA | N_Shore |
| 5117 | cg16783864 | 0.0032 | 0.033621 | 3 | *CMTM7* | Body | NA | - |
| 5118 | cg20750889 | -0.0031 | 0.033656 | 16 | *CDH1* | Body | NA | - |
| 5119 | cg26958260 | 0.0021 | 0.033701 | 11 | *-* | - | NA | - |
| 5120 | cg10137837 | 0.0034 | 0.033749 | 17 | *BCL6B* | 5'UTR | TRUE | Island |
| 5121 | cg26670562 | -0.0021 | 0.03382 | 4 | *MAPK10* | Body | TRUE | - |
| 5122 | cg04983276 | 0.0035 | 0.033827 | 22 | *PPIL2* | TSS200 | TRUE | N_Shore |
| 5123 | cg21595233 | -0.0016 | 0.033828 | 2 | *-* | - | TRUE | - |
| 5124 | cg16015397 | -0.001 | 0.033829 | 21 | *ITGB2* | 5'UTR | NA | - |
| 5125 | cg02509232 | 0.0019 | 0.033851 | 10 | *KNDC1* | Body | TRUE | S_Shelf |
| 5126 | cg17445166 | 0.0026 | 0.033873 | 6 | *RREB1* | 5'UTR | NA | - |
| 5127 | cg18246739 | -6.00E-04 | 0.033873 | 18 | *TGIF1* | 5'UTR | NA | Island |
| 5128 | cg15950279 | -0.0022 | 0.033892 | 2 | *ACOXL* | Body | NA | - |
| 5129 | cg11333314 | 9.00E-04 | 0.033892 | 4 | *PRSS12* | TSS1500 | NA | Island |
| 5130 | cg17969123 | -0.0025 | 0.034012 | 19 | *-* | - | TRUE | - |
| 5131 | cg26039042 | -0.0023 | 0.034058 | 8 | *FAM83H* | 5'UTR | TRUE | Island |
| 5132 | cg26763855 | 0.005 | 0.034058 | 19 | *CCER2* | TSS200 | NA | - |
| 5133 | cg22798948 | -0.0023 | 0.034059 | 3 | *-* | - | NA | - |
| 5134 | cg13439195 | 0.0025 | 0.03409 | 18 | *ABHD3* | Body | NA | - |
| 5135 | cg08578216 | 0.0022 | 0.03409 | 8 | *PEBP4* | Body | TRUE | - |
| 5136 | cg10703273 | -0.0035 | 0.034145 | 22 | *PRR5ARHGAP8* | Body | NA | - |
| 5137 | cg20470236 | -0.0023 | 0.034156 | 16 | *MYLK3* | 5'UTR | NA | - |
| 5138 | cg06692169 | -0.0046 | 0.034176 | 1 | *LYPLAL1* | TSS1500 | NA | N_Shore |
| 5139 | cg13156411 | 0.0014 | 0.03422 | 3 | *PTH1R* | 5'UTR | TRUE | N_Shelf |
| 5140 | cg21161820 | -0.0037 | 0.03422 | 6 | *LOC285768* | Body | NA | - |
| 5141 | cg16464659 | -0.0028 | 0.034255 | 10 | *PLCE1* | Body | NA | - |
| 5142 | cg06159344 | 0.0026 | 0.034272 | 7 | *MAD1L1* | Body | NA | S_Shore |
| 5143 | cg10653504 | -0.0018 | 0.034272 | 19 | *ATP8B3* | 5'UTR | NA | Island |
| 5144 | cg01383082 | -0.0041 | 0.034275 | 8 | *FAM167A* | 5'UTR | TRUE | N_Shore |
| 5145 | cg00626957 | -0.0041 | 0.03431 | 7 | *-* | - | NA | - |
| 5146 | cg09659587 | 0.0025 | 0.03432 | 22 | *ATXN10* | Body | TRUE | S_Shelf |
| 5147 | cg04155830 | 0.004 | 0.034321 | 3 | *NDUFB4* | TSS1500 | TRUE | N_Shore |
| 5148 | cg18567079 | -0.0044 | 0.034321 | 2 | *-* | - | NA | - |
| 5149 | cg14499385 | -0.003 | 0.034324 | 2 | *SLC40A1* | TSS1500 | TRUE | S_Shore |
| 5150 | cg15346115 | -0.0024 | 0.034354 | 11 | *-* | - | NA | - |
| 5151 | cg19832723 | -0.002 | 0.034354 | 22 | *ZMAT5* | Body | NA | - |
| 5152 | cg03753191 | 0.0022 | 0.034354 | 13 | *EPSTI1* | TSS1500 | TRUE | S_Shore |
| 5153 | cg12534424 | 0.0021 | 0.034354 | 7 | *PRRT4* | Body | TRUE | Island |
| 5154 | cg09197432 | -0.0071 | 0.034354 | 4 | *-* | - | TRUE | Island |
| 5155 | cg27039034 | 0.0025 | 0.03438 | 4 | *NFKB1* | Body | NA | - |
| 5156 | cg06038491 | 0.0049 | 0.034401 | 8 | *PCMTD1* | Body | NA | - |
| 5157 | cg11674335 | 0.0026 | 0.034405 | 6 | *-* | - | NA | - |
| 5158 | cg17516907 | -0.0023 | 0.03443 | 17 | *-* | - | NA | - |
| 5159 | cg15139153 | -0.0081 | 0.03443 | 6 | *RNGTT* | Body | NA | - |
| 5160 | cg15679320 | -0.0037 | 0.03443 | 12 | *GLTP* | Body | NA | N_Shore |
| 5161 | cg07375851 | 0.0021 | 0.03443 | 19 | *PGLYRP2* | TSS1500 | TRUE | - |
| 5162 | cg07402808 | -0.004 | 0.034448 | 7 | *PTPRN2* | Body | NA | N_Shore |
| 5163 | cg05946647 | -0.0023 | 0.034448 | 15 | *BAHD1* | 5'UTR | NA | S_Shore |
| 5164 | cg08942051 | 0.005 | 0.034455 | 3 | *-* | - | TRUE | - |
| 5165 | cg24189790 | -0.0025 | 0.034455 | 12 | *SYT1* | 5'UTR | NA | - |
| 5166 | cg09287359 | -0.0018 | 0.034455 | 19 | *LGALS7B* | TSS1500 | TRUE | N_Shore |
| 5167 | cg03650729 | -0.0075 | 0.034455 | 1 | *TAL1* | 5'UTR | NA | S_Shore |
| 5168 | cg04194840 | -0.003 | 0.034473 | 16 | *NDRG4* | 3'UTR | TRUE | N_Shelf |
| 5169 | cg02912379 | -0.0026 | 0.034477 | 1 | *PDE4DIP* | Body | TRUE | - |
| 5170 | cg03144619 | 0.0062 | 0.034493 | 1 | *GALNT2* | 3'UTR | TRUE | Island |
| 5171 | cg12591805 | -0.003 | 0.034497 | 7 | *-* | - | NA | - |
| 5172 | cg04579668 | 0.0029 | 0.034497 | 1 | *-* | - | NA | - |
| 5173 | cg22112832 | -0.0032 | 0.034501 | 2 | *-* | - | TRUE | N_Shore |
| 5174 | cg23460823 | -0.004 | 0.034501 | 3 | *-* | - | NA | - |
| 5175 | cg04134528 | 0.0024 | 0.034532 | 5 | *-* | - | TRUE | - |
| 5176 | cg16077353 | -0.0041 | 0.034556 | 6 | *STXBP5AS1* | Body | NA | - |
| 5177 | cg15600670 | 0.003 | 0.034578 | 15 | *-* | - | NA | - |
| 5178 | cg10121508 | -0.0012 | 0.034619 | 22 | *GAS2L1* | TSS1500 | TRUE | N_Shore |
| 5179 | cg04261821 | 0.0021 | 0.034631 | 1 | *NTRK1* | Body | NA | - |
| 5180 | cg21283823 | -0.0058 | 0.034696 | 9 | *ASTN2* | Body | NA | - |
| 5181 | cg10478319 | -0.0027 | 0.034696 | 6 | *PKHD1* | Body | NA | - |
| 5182 | cg03872447 | -0.0068 | 0.034747 | 7 | *-* | - | NA | - |
| 5183 | cg21123679 | 0.0033 | 0.034758 | 14 | *NRXN3* | Body | NA | - |
| 5184 | cg08901046 | 0.0033 | 0.034765 | 11 | *ETS1* | Body | NA | - |
| 5185 | cg03592636 | 0.0012 | 0.034765 | 6 | *BTBD9* | Body | NA | - |
| 5186 | cg13002996 | -0.0022 | 0.034765 | 6 | *GRM4* | 5'UTR | NA | - |
| 5187 | cg06986667 | -0.0026 | 0.034768 | 15 | *-* | - | NA | - |
| 5188 | cg13679841 | 0.0023 | 0.034772 | 14 | *CEP128* | Body | NA | - |
| 5189 | cg08781559 | 0.0016 | 0.034777 | 1 | *ZYG11A* | 5'UTR | NA | Island |
| 5190 | cg22692362 | -0.0031 | 0.034777 | 11 | *LRRC4C* | TSS1500 | TRUE | - |
| 5191 | cg02272851 | -0.003 | 0.034777 | 10 | *-* | - | TRUE | - |
| 5192 | cg06930745 | 0.0022 | 0.034781 | 1 | *MEF2D* | TSS1500 | NA | - |
| 5193 | cg26758283 | 0.0023 | 0.034787 | 18 | *-* | - | NA | - |
| 5194 | cg02945601 | -0.0041 | 0.03479 | 5 | *ADGRV1* | Body | NA | - |
| 5195 | cg21402844 | 0.0019 | 0.03479 | 6 | *PLN* | 1stExon | NA | - |
| 5196 | cg25503259 | 0.0029 | 0.03479 | 17 | *RAB11FIP4* | Body | NA | - |
| 5197 | cg20302236 | -0.0019 | 0.03479 | 8 | *INTS9* | Body | NA | - |
| 5198 | cg17739917 | -0.0032 | 0.03479 | 17 | *RARA* | 5'UTR | NA | S_Shelf |
| 5199 | cg00970304 | 0.0021 | 0.034793 | 17 | *MPP3* | Body | NA | - |
| 5200 | cg22060161 | -0.002 | 0.034808 | 1 | *PALMD* | Body | NA | - |
| 5201 | cg09874472 | 0.0031 | 0.034808 | 9 | *PGM5* | Body | NA | - |
| 5202 | cg15230342 | -0.0036 | 0.034809 | 21 | *MIR99AHG* | Body | NA | - |
| 5203 | cg09828520 | 0.0015 | 0.034817 | 12 | *METTL21B* | Body | NA | S_Shelf |
| 5204 | cg00839579 | 0.0014 | 0.034864 | 7 | *MEOX2* | TSS1500 | TRUE | - |
| 5205 | cg25123232 | -0.0029 | 0.034864 | 3 | *-* | - | NA | - |
| 5206 | cg16540981 | -0.0038 | 0.034874 | 1 | *C1orf162* | 1stExon | NA | - |
| 5207 | cg06565605 | -0.0027 | 0.034875 | 22 | *-* | - | NA | N_Shelf |
| 5208 | cg24258347 | -0.0043 | 0.034884 | 11 | *SPON1* | TSS200 | TRUE | N_Shore |
| 5209 | cg07848854 | -0.0027 | 0.034884 | 4 | *ADAMTS3* | Body | NA | - |
| 5210 | cg09122999 | -0.0039 | 0.034908 | 7 | *POU6F2* | Body | TRUE | - |
| 5211 | cg08847162 | -0.0018 | 0.034908 | 15 | *-* | - | NA | - |
| 5212 | cg27530093 | -0.0033 | 0.034961 | 19 | *CCDC97* | TSS1500 | TRUE | N_Shore |
| 5213 | cg11634198 | 0.0016 | 0.034961 | 17 | *NEUROD2* | 5'UTR | TRUE | Island |
| 5214 | cg22013564 | -0.0044 | 0.034961 | 7 | *-* | - | TRUE | - |
| 5215 | cg26337602 | 0.004 | 0.034961 | 17 | *NXN* | Body | NA | - |
| 5216 | cg04971650 | -0.003 | 0.034974 | 15 | *-* | - | NA | - |
| 5217 | cg20732539 | 0.0034 | 0.034987 | 16 | *IL21R* | 5'UTR | NA | - |
| 5218 | cg20119745 | 0.0049 | 0.034987 | 6 | *RNF39* | Body | TRUE | Island |
| 5219 | cg24198765 | 0.0023 | 0.035006 | 4 | *ARHGEF38* | TSS1500 | NA | - |
| 5220 | cg23638455 | -0.0086 | 0.035014 | 8 | *SLC45A4* | Body | TRUE | Island |
| 5221 | cg21159778 | 0.001 | 0.035015 | 9 | *DFNB31* | TSS1500 | TRUE | Island |
| 5222 | cg00613089 | 0.0021 | 0.035016 | 12 | *-* | - | NA | - |
| 5223 | cg26666736 | 0.002 | 0.035021 | 1 | *-* | - | NA | - |
| 5224 | cg17573365 | 0.0025 | 0.035021 | 11 | *PACS1* | Body | NA | - |
| 5225 | cg09099082 | -0.003 | 0.035041 | 5 | *CPLX2* | 5'UTR | NA | - |
| 5226 | cg05783613 | -0.0024 | 0.035042 | 9 | *AMBP* | TSS1500 | NA | - |
| 5227 | cg10247798 | -0.002 | 0.035042 | 3 | *ABHD14B* | TSS200 | TRUE | N_Shore |
| 5228 | cg10481065 | 0.0012 | 0.035042 | 20 | *SLC32A1* | 3'UTR | TRUE | Island |
| 5229 | cg26263915 | -0.0039 | 0.035042 | 9 | *-* | - | NA | - |
| 5230 | cg20284207 | -0.002 | 0.035042 | 19 | *-* | - | NA | S_Shore |
| 5231 | cg00398421 | -0.0035 | 0.035042 | 11 | *-* | - | NA | - |
| 5232 | cg19673176 | -0.0024 | 0.035042 | 17 | *HRNBP3* | 5'UTR | TRUE | - |
| 5233 | cg04021592 | -0.0032 | 0.035052 | 2 | *-* | - | TRUE | N_Shore |
| 5234 | cg26304744 | -0.0019 | 0.035075 | 15 | *RORA* | Body | TRUE | - |
| 5235 | cg01297756 | 0.0021 | 0.035077 | 13 | *CLYBL* | 3'UTR | TRUE | Island |
| 5236 | cg01262290 | -0.0036 | 0.035102 | 7 | *OR6V1* | TSS1500 | TRUE | - |
| 5237 | cg05245794 | 0.0028 | 0.035102 | 8 | *-* | - | TRUE | - |
| 5238 | cg12577010 | -0.0013 | 0.035113 | 7 | *TMEM140* | TSS1500 | TRUE | - |
| 5239 | cg05062747 | -0.0016 | 0.035113 | 8 | *-* | - | NA | - |
| 5240 | cg18274510 | -0.0019 | 0.035113 | 10 | *HERC4* | Body | TRUE | - |
| 5241 | cg02092514 | 0.0026 | 0.035124 | 17 | *TBCD* | Body | TRUE | S_Shelf |
| 5242 | cg20539142 | 0.0043 | 0.035129 | 8 | *CHR-6* | TSS200 | TRUE | - |
| 5243 | cg21381949 | -0.0023 | 0.035154 | 3 | *LEPREL1* | Body | TRUE | - |
| 5244 | cg21609875 | 0.0053 | 0.035154 | 18 | *CDH2* | Body | NA | - |
| 5245 | cg22377345 | 0.0024 | 0.035154 | 11 | *-* | - | NA | S_Shelf |
| 5246 | cg10174333 | -0.003 | 0.035154 | 1 | *-* | - | TRUE | - |
| 5247 | cg12464671 | 0.0032 | 0.035154 | 10 | *PDLIM1* | Body | NA | - |
| 5248 | cg08262220 | -0.0034 | 0.035156 | 1 | *PRDM16* | Body | TRUE | N_Shore |
| 5249 | cg03636295 | 0.0015 | 0.035156 | 4 | *APBB2* | Body | NA | - |
| 5250 | cg12393166 | -0.0031 | 0.035156 | 12 | *-* | - | NA | - |
| 5251 | cg17283083 | -0.0017 | 0.035158 | 10 | *HK1* | Body | TRUE | - |
| 5252 | cg23768539 | -0.0019 | 0.035219 | 2 | *ABHD1* | Body | NA | - |
| 5253 | cg05195247 | 7.00E-04 | 0.035247 | 20 | *C20orf26* | 5'UTR | TRUE | Island |
| 5254 | cg17186221 | 0.0018 | 0.03529 | 9 | *ADAMTSL2* | Body | NA | S_Shelf |
| 5255 | cg22986941 | 0.0032 | 0.035349 | 10 | *FAM53B* | 5'UTR | NA | - |
| 5256 | cg11640529 | -0.0045 | 0.035349 | 20 | *PKIG* | 5'UTR | NA | - |
| 5257 | cg19025497 | -0.0021 | 0.035352 | 5 | *SIL1* | TSS1500 | TRUE | S_Shore |
| 5258 | cg18016128 | 0.0021 | 0.035372 | 17 | *C17orf76* | TSS1500 | TRUE | S_Shore |
| 5259 | cg01812045 | 0.0015 | 0.035386 | 2 | *DES* | 1stExon | TRUE | Island |
| 5260 | cg22851821 | 0.0045 | 0.035386 | 4 | *-* | - | NA | - |
| 5261 | cg08545213 | -0.0026 | 0.035386 | 3 | *CTDSPL* | Body | TRUE | - |
| 5262 | cg13702217 | -0.0041 | 0.035417 | 11 | *-* | - | NA | - |
| 5263 | cg02654775 | 0.0033 | 0.035417 | 12 | *LRP1* | Body | NA | - |
| 5264 | cg03444488 | 0.0018 | 0.035417 | 4 | *ACSL1* | TSS1500 | NA | - |
| 5265 | cg04307162 | -0.0038 | 0.035472 | 1 | *EIF2B3* | TSS1500 | NA | S_Shore |
| 5266 | cg22967717 | -0.003 | 0.035496 | 5 | *-* | - | TRUE | - |
| 5267 | cg07008355 | 0.0017 | 0.035511 | 11 | *MOB2* | TSS1500 | NA | - |
| 5268 | cg26396680 | 0.0032 | 0.035542 | 11 | *PPP1R32* | Body | NA | - |
| 5269 | cg04542977 | -0.0029 | 0.035623 | 1 | *EPS15* | Body | TRUE | N_Shore |
| 5270 | cg18677212 | -0.0023 | 0.035639 | 9 | *RFX3* | Body | NA | - |
| 5271 | cg26316404 | 0.0024 | 0.035661 | 17 | *-* | - | NA | - |
| 5272 | cg27274542 | 0.0032 | 0.035666 | 20 | *NPEPL1* | TSS200 | TRUE | Island |
| 5273 | cg08233602 | 0.0019 | 0.035666 | 3 | *NCBP2* | Body | NA | N_Shelf |
| 5274 | cg04696619 | -0.0032 | 0.035709 | 9 | *-* | - | NA | - |
| 5275 | cg03773647 | 0.0033 | 0.035709 | 11 | *YPEL4* | TSS1500 | TRUE | S_Shelf |
| 5276 | cg15577126 | -0.0035 | 0.035731 | 2 | *-* | - | TRUE | - |
| 5277 | cg00459119 | 0.0028 | 0.035772 | 16 | *SNX29* | Body | TRUE | - |
| 5278 | cg23331484 | 0.0039 | 0.035776 | 19 | *SLC7A10* | TSS1500 | TRUE | S_Shore |
| 5279 | cg04444104 | 0.0034 | 0.035804 | 16 | *-* | - | TRUE | - |
| 5280 | cg23346544 | -0.0043 | 0.035817 | 8 | *-* | - | TRUE | - |
| 5281 | cg03305963 | -0.0036 | 0.035817 | 11 | *MSANTD2* | Body | NA | - |
| 5282 | cg02024925 | -0.0034 | 0.035817 | 13 | *-* | - | TRUE | S_Shelf |
| 5283 | cg07119912 | 0.0019 | 0.035833 | 12 | *KIF5A* | 1stExon | NA | - |
| 5284 | cg14836018 | -0.0032 | 0.035834 | 14 | *TMX1* | TSS1500 | TRUE | N_Shore |
| 5285 | cg04422227 | -0.0038 | 0.035835 | 12 | *E2F7* | Body | NA | - |
| 5286 | cg27449365 | 0.0024 | 0.035855 | 4 | *-* | - | NA | - |
| 5287 | cg00474850 | -0.0032 | 0.035855 | 20 | *COL9A3* | Body | NA | S_Shore |
| 5288 | cg09259179 | 0.0021 | 0.035855 | 1 | *ILDR2* | Body | TRUE | S_Shore |
| 5289 | cg07584928 | 0.002 | 0.035855 | 1 | *B4GALT3* | TSS1500 | NA | S_Shore |
| 5290 | cg23625660 | 0.0014 | 0.035891 | 5 | *FBXL21* | 5'UTR | TRUE | Island |
| 5291 | cg05432134 | -0.0023 | 0.035896 | 16 | *-* | - | NA | - |
| 5292 | cg00920130 | -0.0029 | 0.035896 | 5 | *LCP2* | Body | NA | - |
| 5293 | cg13007701 | -0.0031 | 0.035896 | 8 | *DLC1* | Body | TRUE | - |
| 5294 | cg22057584 | -0.0032 | 0.035896 | 19 | *-* | - | NA | - |
| 5295 | cg02389682 | 0.0018 | 0.035896 | 8 | *HEATR7A* | Body | TRUE | S_Shelf |
| 5296 | cg22133576 | -0.0031 | 0.035896 | 4 | *PALLD* | Body | NA | - |
| 5297 | cg03725414 | -0.0025 | 0.035904 | 16 | *CRISPLD2* | 5'UTR | NA | - |
| 5298 | cg17570569 | -0.0034 | 0.035904 | 1 | *-* | - | NA | - |
| 5299 | cg05204491 | -0.0033 | 0.035904 | 7 | *RAPGEF5* | Body | NA | - |
| 5300 | cg12378111 | -0.0013 | 0.035904 | 8 | *-* | - | NA | - |
| 5301 | cg04306184 | -0.0026 | 0.035904 | 6 | *ARID1B* | Body | NA | - |
| 5302 | cg06714006 | -0.0055 | 0.035904 | 10 | *COL13A1* | Body | NA | - |
| 5303 | cg26099045 | -0.0041 | 0.035904 | 2 | *-* | - | TRUE | - |
| 5304 | cg01177168 | -0.003 | 0.035904 | 7 | *-* | - | TRUE | - |
| 5305 | cg05194346 | -0.005 | 0.035904 | 15 | *-* | - | TRUE | - |
| 5306 | cg00533407 | -0.0021 | 0.035904 | 7 | *ACCN3* | TSS1500 | TRUE | N_Shore |
| 5307 | cg12500949 | 0.0023 | 0.035904 | 2 | *-* | - | TRUE | S_Shelf |
| 5308 | cg03301331 | 0.0013 | 0.035904 | 1 | *RAB4A* | TSS200 | TRUE | Island |
| 5309 | cg17155829 | 0.0032 | 0.03591 | 19 | *-* | - | NA | - |
| 5310 | cg05144285 | 0.005 | 0.035915 | 7 | *VIPR2* | Body | TRUE | N_Shore |
| 5311 | cg24654279 | -0.0063 | 0.035959 | 6 | *-* | - | NA | - |
| 5312 | cg23298492 | 8.00E-04 | 0.035973 | 19 | *ANO8* | TSS200 | TRUE | Island |
| 5313 | cg25220336 | -0.0014 | 0.035973 | 12 | *PXN* | Body | NA | - |
| 5314 | cg25260699 | 0.0025 | 0.035973 | 17 | *PGS1* | Body | NA | - |
| 5315 | cg03757250 | 0.0021 | 0.036056 | 19 | *TMIGD2* | TSS1500 | TRUE | N_Shore |
| 5316 | cg14383544 | 0.003 | 0.036084 | 11 | *KCNJ5* | 5'UTR | NA | S_Shelf |
| 5317 | cg22511388 | 0.0024 | 0.036106 | 10 | *LOC101927472* | TSS1500 | NA | - |
| 5318 | cg07770855 | -0.0034 | 0.036106 | 11 | *CD151* | 5'UTR | NA | S_Shore |
| 5319 | cg12008779 | -0.0058 | 0.036175 | 6 | *QKI* | Body | NA | - |
| 5320 | cg15254559 | 0.0025 | 0.036212 | 7 | *-* | - | TRUE | Island |
| 5321 | cg05525812 | 0.0027 | 0.036223 | 2 | *-* | - | TRUE | N_Shore |
| 5322 | cg03984919 | -0.0015 | 0.036258 | 2 | *NCOA1* | TSS1500 | TRUE | - |
| 5323 | cg15904523 | 0.0018 | 0.036297 | 19 | *ZNF233* | TSS200 | TRUE | Island |
| 5324 | cg04747102 | -0.0023 | 0.036307 | 2 | *-* | - | NA | - |
| 5325 | cg11960359 | -0.0042 | 0.036312 | 8 | *-* | - | NA | - |
| 5326 | cg03060555 | -0.0031 | 0.036359 | 3 | *ZBTB38* | 5'UTR | TRUE | - |
| 5327 | cg04619882 | -0.0023 | 0.036387 | 1 | *KIAA1324* | Body | TRUE | - |
| 5328 | cg20513976 | 0.0031 | 0.036387 | 20 | *LIME1* | TSS200 | TRUE | N_Shore |
| 5329 | cg09109730 | 0.0045 | 0.036407 | 18 | *SALL3* | Body | NA | - |
| 5330 | cg03662064 | -0.0033 | 0.036407 | 13 | *FLT1* | TSS1500 | TRUE | Island |
| 5331 | cg18797778 | -0.0037 | 0.036418 | X | *GPR143* | Body | NA | - |
| 5332 | cg05529039 | -0.0026 | 0.036465 | 17 | *PSME3* | 3'UTR | TRUE | - |
| 5333 | cg08516641 | -0.003 | 0.036466 | 10 | *PTPRE* | 5'UTR | NA | - |
| 5334 | cg14319451 | -0.0033 | 0.036473 | 11 | *RCOR2* | TSS1500 | TRUE | Island |
| 5335 | cg17526301 | 0.0014 | 0.036537 | 20 | *-* | - | TRUE | - |
| 5336 | cg15576436 | -0.0054 | 0.036562 | 5 | *-* | - | NA | - |
| 5337 | cg11945507 | -0.0035 | 0.036607 | 8 | *SLC45A4* | Body | TRUE | S_Shore |
| 5338 | cg22466575 | 0.0019 | 0.03662 | 20 | *SLC9A8* | Body | NA | - |
| 5339 | cg03648784 | -0.0034 | 0.036641 | 6 | *NRM* | TSS1500 | NA | S_Shelf |
| 5340 | cg20028002 | -0.0022 | 0.036641 | 13 | *SPATA13* | Body | NA | - |
| 5341 | cg00145253 | 0.0014 | 0.036641 | 1 | *LHX8* | Body | TRUE | Island |
| 5342 | cg19291946 | -0.0024 | 0.036649 | 3 | *PTPN23* | TSS1500 | NA | N_Shore |
| 5343 | cg07852684 | -0.0025 | 0.036693 | 11 | *ACAT1* | Body | NA | - |
| 5344 | cg00203621 | -0.0055 | 0.036715 | 8 | *SYBU* | TSS1500 | NA | - |
| 5345 | cg16121136 | -0.0023 | 0.036777 | 20 | *HNF4A* | TSS1500 | TRUE | - |
| 5346 | cg01518398 | 0.0025 | 0.036782 | 2 | *MOBKL1B* | TSS1500 | TRUE | S_Shore |
| 5347 | cg14793287 | 0.0018 | 0.036789 | 12 | *C12orf68* | 3'UTR | TRUE | Island |
| 5348 | cg13232402 | -0.003 | 0.036851 | 1 | *DENND2D* | TSS1500 | NA | S_Shore |
| 5349 | cg14221852 | -0.0034 | 0.036855 | 3 | *KIAA0226* | Body | NA | - |
| 5350 | cg22123156 | -0.002 | 0.036861 | 11 | *PDE2A* | Body | TRUE | - |
| 5351 | cg25491287 | -0.0023 | 0.036872 | 12 | *VWF* | Body | NA | - |
| 5352 | cg16398761 | -0.003 | 0.036884 | 14 | *C14orf43* | 5'UTR | TRUE | - |
| 5353 | cg12812662 | -0.0021 | 0.036893 | 17 | *DUSP3* | TSS1500 | TRUE | S_Shore |
| 5354 | cg15990646 | 0.002 | 0.03691 | 3 | *-* | - | NA | - |
| 5355 | cg00458274 | -0.002 | 0.03691 | 17 | *GUCY2D* | Body | TRUE | Island |
| 5356 | cg16350446 | -0.0023 | 0.036915 | 2 | *DCAF17* | Body | TRUE | - |
| 5357 | cg18938861 | -0.0019 | 0.036915 | 14 | *-* | - | NA | - |
| 5358 | cg11420764 | -0.003 | 0.036915 | 11 | *-* | - | NA | - |
| 5359 | cg00289431 | -0.0028 | 0.036915 | 11 | *-* | - | NA | - |
| 5360 | cg23750151 | 0.0024 | 0.036915 | 6 | *PSMB8* | 3'UTR | TRUE | S_Shore |
| 5361 | cg08977866 | 0.0019 | 0.036915 | 8 | *-* | - | NA | - |
| 5362 | cg07948323 | -0.0044 | 0.036915 | 4 | *-* | - | NA | - |
| 5363 | cg09522017 | 0.0016 | 0.036915 | 20 | *CEP250* | 1stExon | NA | - |
| 5364 | cg12892688 | -0.002 | 0.036915 | 11 | *-* | - | NA | - |
| 5365 | cg21607248 | 0.0018 | 0.036915 | 20 | *-* | - | TRUE | N_Shore |
| 5366 | cg00448707 | 0.0041 | 0.036915 | 1 | *DIRAS3* | Body | TRUE | Island |
| 5367 | cg06721502 | 0.0019 | 0.036915 | 20 | *-* | - | NA | N_Shore |
| 5368 | cg23913069 | 0.0024 | 0.036943 | 10 | *PTPRE* | Body | NA | - |
| 5369 | cg23420528 | 8.00E-04 | 0.036962 | 22 | *PEX26* | TSS200 | TRUE | Island |
| 5370 | cg26316885 | 0.0071 | 0.036979 | 19 | *ICAM5* | 3'UTR | TRUE | Island |
| 5371 | cg04249191 | -0.0021 | 0.037017 | 1 | *-* | - | TRUE | - |
| 5372 | cg21015019 | 0.0023 | 0.037039 | 2 | *-* | - | NA | - |
| 5373 | cg25214674 | 0.0013 | 0.037049 | 16 | *ATXN1L* | 1stExon | NA | Island |
| 5374 | cg23942160 | -0.0033 | 0.037049 | 16 | *-* | - | NA | N_Shore |
| 5375 | cg10290276 | 0.0018 | 0.037049 | 11 | *ASCL2* | 1stExon | TRUE | Island |
| 5376 | cg11199639 | 0.0107 | 0.03705 | 8 | *ADAM5P* | TSS200 | TRUE | - |
| 5377 | cg10204912 | -0.0031 | 0.037079 | 20 | *NFATC2* | Body | NA | N_Shore |
| 5378 | cg26830053 | -0.0046 | 0.037081 | 16 | *-* | - | NA | - |
| 5379 | cg21479515 | -0.0033 | 0.037081 | 7 | *-* | - | NA | - |
| 5380 | cg13465542 | -0.0027 | 0.037081 | 12 | *PLEKHG6* | Body | NA | - |
| 5381 | cg13043297 | -0.0096 | 0.037084 | 3 | *MAGI1* | Body | NA | - |
| 5382 | cg16922810 | -0.0017 | 0.037084 | 8 | *ARC* | TSS1500 | TRUE | S_Shore |
| 5383 | cg03305185 | 0.0053 | 0.037096 | X | *ZNF75D* | 5'UTR | TRUE | Island |
[truncated: 72,494 more chars]
