# Supplementary material for: Age prediction of children and adolescents aged 6-17 years: an epigenome-wide analysis of DNA methylation
Source: Aging (Albany NY). 2018 May 12;10(5):1015–26. doi: 10.18632/aging.101445 (PMC5990383; doi:10.18632/aging.101445)
Supplement: Table S2 [file aging-10-101445-s003.docx]

| **Table S2. 116 overlapped age predictive CpG sites between GSE27097 data and the present study in children and adolescents** | | | | | | | | | |
| --- | --- | --- | --- | --- | --- | --- | --- | --- | --- |
|  | probename | eAGE | pAGE | FDR | CHR | Gene Name | Gene Group | Methyl450 Loci | Relation to CpG Island |
| 1 | cg00022866 | 0.0041 | 2.62E-04 | 3.87E-02 | 11 | *CCDC88B* | Body | TRUE | N_Shore |
| 2 | cg00059225 | 0.0031 | 7.39E-06 | 3.13E-03 | 5 | *GLRA1* | 1stExon | TRUE | Island |
| 3 | cg00187686 | -0.0034 | 3.63E-05 | 1.01E-02 | 11 | *TCN1* | TSS200 | TRUE | Other |
| 4 | cg00343092 | -0.0039 | 2.07E-04 | 3.32E-02 | 22 | *TSPO* | 5'UTR | TRUE | Island |
| 5 | cg00431114 | -0.0030 | 1.02E-06 | 7.02E-04 | 20 | *TTPAL* | TSS1500 | TRUE | N_Shore |
| 6 | cg00468146 | 0.0018 | 1.56E-04 | 2.75E-02 | 6 | *ID4* | 1stExon | TRUE | Island |
| 7 | cg00503840 | 0.0016 | 3.23E-04 | 4.44E-02 | 7 | *DLX5* | Body | TRUE | Island |
| 8 | cg00563932 | -0.0029 | 1.52E-04 | 2.69E-02 | 9 | *PTGDS* | TSS1500 | TRUE | N_Shore |
| 9 | cg01797043 | -0.0028 | 1.68E-07 | 1.74E-04 | 16 | *RPL3L* | TSS200 | TRUE | Other |
| 10 | cg01806928 | 0.0040 | 3.71E-05 | 1.02E-02 | 11 | *SYT9* | TSS1500 | TRUE | Island |
| 11 | cg01899253 | -0.0052 | 7.18E-06 | 3.06E-03 | 13 | *FLT1* | TSS1500 | TRUE | S_Shore |
| 12 | cg01946401 | -0.0076 | 4.32E-05 | 1.14E-02 | 6 | *SUPT3H* | 5'UTR | TRUE | Other |
| 13 | cg02151301 | -0.0028 | 1.46E-04 | 2.63E-02 | 20 | *HM13* | TSS1500 | TRUE | N_Shore |
| 14 | cg02657721 | -0.0031 | 6.36E-08 | 8.05E-05 | 3 | *SEMA3B* | TSS1500 | NA | Other |
| 15 | cg02988947 | 0.0044 | 1.23E-07 | 1.36E-04 | 17 | *LIMD2* | TSS1500 | TRUE | S_Shore |
| 16 | cg03165378 | 0.0023 | 7.83E-05 | 1.70E-02 | 1 | *S100A9* | TSS1500 | TRUE | Other |
| 17 | cg03224418 | -0.0033 | 3.54E-08 | 4.98E-05 | 20 | *SAMD10* | TSS1500 | TRUE | N_Shore |
| 18 | cg03365437 | 0.0014 | 3.22E-05 | 9.24E-03 | 15 | *ALDH1A2* | 5'UTR | TRUE | Island |
| 19 | cg03972838 | -0.0019 | 9.99E-05 | 2.01E-02 | 22 | *SMCR7L* | TSS1500 | TRUE | N_Shore |
| 20 | cg03996822 | -0.0042 | 3.78E-05 | 1.04E-02 | 4 | *RASSF6* | TSS1500 | TRUE | S_Shore |
| 21 | cg04039397 | -0.0058 | 1.93E-04 | 3.17E-02 | 3 | *CD96* | TSS200 | TRUE | Other |
| 22 | cg04084157 | 0.0012 | 9.82E-06 | 3.86E-03 | 7 | *VGF* | TSS200 | TRUE | Island |
| 23 | cg04126866 | 0.0026 | 6.84E-05 | 1.55E-02 | 10 | *C10orf99* | TSS1500 | TRUE | Other |
| 24 | cg04527918 | 0.0044 | 4.79E-05 | 1.23E-02 | 2 | *UCN* | TSS200 | TRUE | Island |
| 25 | cg04586023 | -0.0026 | 1.02E-04 | 2.03E-02 | 22 | *HDAC10* | TSS1500 | TRUE | S_Shore |
| 26 | cg04662594 | -0.0043 | 1.22E-05 | 4.50E-03 | 8 | *EPB49* | 5'UTR | TRUE | S_Shelf |
| 27 | cg05155595 | -0.0037 | 3.43E-04 | 4.62E-02 | 2 | *ANXA4* | 5'UTR | TRUE | Other |
| 28 | cg05921699 | 0.0030 | 4.90E-06 | 2.30E-03 | 19 | *CD79A* | TSS1500 | TRUE | Other |
| 29 | cg06493994 | 0.0021 | 8.08E-10 | 2.39E-06 | 6 | *SCGN* | 1stExon | TRUE | Island |
| 30 | cg07211259 | -0.0024 | 1.52E-04 | 2.69E-02 | 9 | *PDCD1LG2* | TSS200 | TRUE | Other |
| 31 | cg07313155 | -0.0013 | 2.49E-05 | 7.67E-03 | 17 | *THRA* | TSS1500 | TRUE | N_Shore |
| 32 | cg07337598 | 0.0029 | 5.71E-06 | 2.58E-03 | 1 | *ANXA9* | TSS1500 | TRUE | Other |
| 33 | cg07408456 | -0.0060 | 7.85E-09 | 1.52E-05 | 19 | *PGLYRP2* | TSS1500 | TRUE | Other |
| 34 | cg08090640 | -0.0025 | 1.38E-04 | 2.53E-02 | 17 | *IFI35* | Body | TRUE | Other |
| 35 | cg08241785 | -0.0025 | 3.38E-05 | 9.57E-03 | 5 | *F2RL2* | TSS200 | TRUE | Other |
| 36 | cg08695830 | -0.0022 | 1.01E-04 | 2.03E-02 | 7 | *CLIP2* | TSS1500 | TRUE | Island |
| 37 | cg08828036 | 0.0057 | 1.69E-04 | 2.89E-02 | 15 | *ATP10A* | TSS1500 | TRUE | S_Shore |
| 38 | cg09118625 | 0.0036 | 7.75E-05 | 1.69E-02 | 1 | *DIRAS3* | Body | TRUE | Island |
| 39 | cg09563216 | 0.0043 | 7.13E-06 | 3.04E-03 | 1 | *C1orf51* | 1stExon | TRUE | S_Shore |
| 40 | cg09704415 | 0.0048 | 3.50E-04 | 4.68E-02 | 1 | *SPATA6* | Body | TRUE | N_Shore |
| 41 | cg09809672 | -0.0079 | 3.91E-07 | 3.28E-04 | 1 | *EDARADD* | TSS1500 | TRUE | N_Shore |
| 42 | cg09863066 | -0.0030 | 7.75E-07 | 5.66E-04 | 22 | *PVALB* | 5'UTR | TRUE | S_Shore |
| 43 | cg10084993 | -0.0029 | 1.53E-05 | 5.34E-03 | 16 | *SLC9A3R2* | Body | TRUE | S_Shore |
| 44 | cg10734665 | 0.0052 | 5.76E-07 | 4.46E-04 | 15 | *ATP10A* | Body | TRUE | N_Shore |
| 45 | cg10784813 | 0.0040 | 7.40E-05 | 1.64E-02 | 16 | *SOCS1* | 3'UTR | TRUE | Island |
| 46 | cg10822172 | -0.0044 | 2.45E-07 | 2.30E-04 | 7 | *CREB5* | TSS200 | TRUE | Other |
| 47 | cg10986043 | -0.0044 | 1.10E-10 | 4.71E-07 | 17 | *TCAP* | TSS1500 | TRUE | N_Shelf |
| 48 | cg11105610 | 0.0051 | 2.84E-08 | 4.20E-05 | 17 | *LGALS3BP* | TSS1500 | TRUE | Other |
| 49 | cg11126134 | 0.0036 | 1.02E-05 | 3.97E-03 | 13 | *C13orf33* | TSS200 | TRUE | Island |
| 50 | cg11299964 | -0.0021 | 3.42E-06 | 1.75E-03 | 9 | *MAPKAP1* | TSS1500 | TRUE | S_Shore |
| 51 | cg11314684 | -0.0039 | 1.30E-04 | 2.43E-02 | 1 | *AKT3* | Body | TRUE | Other |
| 52 | cg11438428 | 0.0013 | 3.18E-04 | 4.41E-02 | 10 | *PTF1A* | 1stExon | TRUE | Island |
| 53 | cg11634198 | 0.0016 | 2.24E-04 | 3.50E-02 | 17 | *NEUROD2* | 5'UTR | TRUE | Island |
| 54 | cg11752275 | -0.0036 | 3.08E-05 | 8.97E-03 | 2 | *GNLY* | TSS1500 | TRUE | Other |
| 55 | cg11827101 | -0.0024 | 1.44E-04 | 2.60E-02 | 2 | *--* | Other | TRUE | Other |
| 56 | cg12091331 | -0.0045 | 1.80E-04 | 3.01E-02 | 8 | *PLAT* | TSS200 | TRUE | Other |
| 57 | cg12261786 | -0.0036 | 1.36E-06 | 8.74E-04 | 10 | *C10orf116* | TSS1500 | TRUE | N_Shore |
| 58 | cg12467090 | 0.0037 | 2.23E-06 | 1.27E-03 | 1 | *PIK3C2B* | 5'UTR | TRUE | Other |
| 59 | cg12532667 | -0.0037 | 6.94E-05 | 1.57E-02 | 4 | *EMCN* | TSS1500 | TRUE | Other |
| 60 | cg12564453 | -0.0117 | 2.42E-07 | 2.30E-04 | 16 | *CETP* | 5'UTR | TRUE | Other |
| 61 | cg12902039 | 0.0012 | 6.71E-05 | 1.54E-02 | 15 | *OCA2* | 1stExon | TRUE | Island |
| 62 | cg13269407 | -0.0065 | 7.48E-05 | 1.65E-02 | 22 | *C22orf26* | TSS200 | TRUE | Island |
| 63 | cg13302154 | -0.0074 | 4.56E-07 | 3.73E-04 | 12 | *MGP* | TSS1500 | TRUE | Other |
| 64 | cg13931228 | -0.0028 | 3.04E-05 | 8.90E-03 | 7 | *MPP6* | TSS1500 | TRUE | Other |
| 65 | cg14918082 | 0.0148 | 1.22E-06 | 8.06E-04 | 17 | *KCNAB3* | TSS1500 | TRUE | N_Shore |
| 66 | cg15195412 | -0.0029 | 2.45E-04 | 3.71E-02 | 16 | *CX3CL1* | Body | TRUE | Other |
| 67 | cg15297650 | -0.0036 | 1.04E-05 | 4.03E-03 | 2 | *TMEM163* | TSS1500 | TRUE | S_Shore |
| 68 | cg15377518 | -0.0031 | 4.06E-06 | 1.99E-03 | 2 | *ZEB2* | TSS200 | TRUE | N_Shelf |
| 69 | cg15417244 | -0.0083 | 9.24E-05 | 1.90E-02 | 12 | *PDZRN4* | 5'UTR | TRUE | Other |
| 70 | cg15538427 | -0.0022 | 1.52E-05 | 5.34E-03 | 11 | *LRRN4CL* | 5'UTR | TRUE | S_Shore |
| 71 | cg15853125 | 0.0012 | 2.72E-05 | 8.21E-03 | 21 | *TIAM1* | TSS1500 | TRUE | S_Shore |
| 72 | cg16273597 | -0.0025 | 3.65E-04 | 4.80E-02 | 6 | *CD83* | TSS1500 | TRUE | N_Shore |
| 73 | cg16313343 | 0.0030 | 1.11E-09 | 3.11E-06 | 14 | *BRF1* | TSS1500 | TRUE | S_Shore |
| 74 | cg16786458 | -0.0057 | 1.49E-04 | 2.66E-02 | 5 | *PPARGC1B* | TSS1500 | TRUE | N_Shore |
| 75 | cg17431739 | -0.0033 | 6.09E-05 | 1.44E-02 | 10 | *MSRB2* | TSS1500 | TRUE | N_Shore |
| 76 | cg17471102 | -0.0044 | 1.84E-08 | 2.97E-05 | 19 | *FUT3* | 5'UTR | TRUE | Other |
| 77 | cg18053607 | -0.0027 | 8.45E-05 | 1.79E-02 | 22 | *INPP5J* | 5'UTR | TRUE | Other |
| 78 | cg18236477 | 0.0020 | 3.20E-05 | 9.20E-03 | 13 | *ATP8A2* | Body | TRUE | Island |
| 79 | cg18468844 | -0.0026 | 1.88E-04 | 3.11E-02 | 1 | *PTAFR* | TSS1500 | TRUE | Other |
| 80 | cg18486150 | 0.0025 | 5.25E-05 | 1.31E-02 | 1 | *KIF17* | TSS1500 | TRUE | S_Shore |
| 81 | cg18490846 | -0.0018 | 3.65E-04 | 4.81E-02 | 17 | *C17orf73* | TSS1500 | TRUE | Other |
| 82 | cg18628483 | -0.0029 | 1.77E-04 | 2.98E-02 | 12 | *KIF5A* | TSS1500 | TRUE | N_Shore |
| 83 | cg18788940 | -0.0042 | 1.11E-05 | 4.24E-03 | 11 | *HTATIP2* | TSS1500 | TRUE | N_Shore |
| 84 | cg18992688 | -0.0026 | 8.45E-07 | 6.08E-04 | 1 | *AVPR1B* | TSS1500 | TRUE | N_Shore |
| 85 | cg19118077 | -0.0068 | 6.00E-05 | 1.43E-02 | 10 | *AKR1C3* | Body | NA | Other |
| 86 | cg19426827 | -0.0024 | 1.42E-04 | 2.58E-02 | 14 | *ADSSL1* | Body | TRUE | S_Shore |
| 87 | cg19722847 | -0.0033 | 7.60E-05 | 1.67E-02 | 12 | *IPO8* | TSS1500 | TRUE | S_Shore |
| 88 | cg19759064 | -0.0045 | 2.96E-05 | 8.72E-03 | 7 | *PHKG1* | 1stExon | TRUE | Other |
| 89 | cg19761273 | -0.0039 | 6.30E-17 | 4.28E-12 | 17 | *CSNK1D* | TSS1500 | TRUE | S_Shore |
| 90 | cg20143092 | -0.0013 | 2.93E-05 | 8.67E-03 | 11 | *CD44* | TSS200 | TRUE | N_Shore |
| 91 | cg21296230 | 0.0015 | 1.56E-04 | 2.74E-02 | 15 | *GREM1* | 5'UTR | TRUE | Island |
| 92 | cg22022041 | 0.0036 | 4.70E-07 | 3.82E-04 | 3 | *CCR9* | 5'UTR | TRUE | Other |
| 93 | cg22171829 | -0.0069 | 5.87E-05 | 1.40E-02 | 7 | *PDK4* | 1stExon | TRUE | Island |
| 94 | cg22730004 | -0.0069 | 6.34E-05 | 1.48E-02 | 1 | *SPTA1* | TSS1500 | TRUE | Other |
| 95 | cg22736354 | 0.0025 | 1.20E-06 | 7.99E-04 | 6 | *NHLRC1* | 1stExon | TRUE | Island |
| 96 | cg22901840 | 0.0034 | 9.63E-06 | 3.81E-03 | 1 | *DIRAS3* | Body | TRUE | Island |
| 97 | cg22947000 | -0.0059 | 2.38E-06 | 1.34E-03 | 16 | *BCMO1* | TSS200 | TRUE | Other |
| 98 | cg22956254 | -0.0020 | 1.90E-04 | 3.13E-02 | 12 | *GDF3* | TSS1500 | TRUE | Other |
| 99 | cg22971191 | 0.0064 | 3.91E-05 | 1.06E-02 | 13 | *SLC10A2* | TSS1500 | TRUE | Other |
| 100 | cg23213217 | 0.0027 | 5.88E-05 | 1.40E-02 | 1 | *DEGS1* | TSS1500 | TRUE | N_Shore |
| 101 | cg23320649 | -0.0022 | 1.61E-04 | 2.79E-02 | 3 | *C3orf18* | 5'UTR | TRUE | N_Shore |
| 102 | cg23587449 | -0.0015 | 9.75E-06 | 3.84E-03 | 4 | *LRAT* | TSS1500 | TRUE | N_Shore |
| 103 | cg23665568 | -0.0048 | 8.00E-08 | 9.77E-05 | 1 | *BCL10* | TSS1500 | TRUE | S_Shelf |
| 104 | cg23760945 | 0.0025 | 3.24E-04 | 4.46E-02 | 19 | *ELOF1* | Body | TRUE | Other |
| 105 | cg24642523 | 0.0018 | 2.51E-04 | 3.77E-02 | 14 | *HSPA2* | 1stExon | TRUE | S_Shore |
| 106 | cg24821554 | -0.0026 | 3.73E-04 | 4.86E-02 | 13 | *GUCY1B2* | Body | TRUE | Other |
| 107 | cg24871743 | 0.0046 | 8.57E-07 | 6.13E-04 | 1 | *DIRAS3* | Body | TRUE | Island |
| 108 | cg25268718 | -0.0030 | 3.70E-05 | 1.02E-02 | 14 | *PSME1* | TSS1500 | TRUE | N_Shore |
| 109 | cg25711779 | 0.0019 | 3.24E-04 | 4.46E-02 | 2 | *EFEMP1* | 5'UTR | TRUE | N_Shore |
| 110 | cg25809905 | -0.0071 | 5.11E-11 | 2.48E-07 | 17 | *ITGA2B* | TSS1500 | TRUE | Other |
| 111 | cg26394940 | -0.0059 | 1.01E-04 | 2.02E-02 | 22 | *C22orf26* | Body | TRUE | N_Shore |
| 112 | cg27015931 | -0.0017 | 1.39E-06 | 8.89E-04 | 16 | *C16orf65* | 1stExon | TRUE | Other |
| 113 | cg27035169 | -0.0036 | 7.43E-05 | 1.64E-02 | 14 | *SLC7A8* | TSS1500 | TRUE | Other |
| 114 | cg27210390 | -0.0035 | 9.02E-05 | 1.87E-02 | 17 | *TOM1L1* | Body | TRUE | S_Shore |
| 115 | cg27236973 | -0.0030 | 2.67E-07 | 2.47E-04 | 17 | *KRT17* | TSS1500 | TRUE | Other |
| 116 | cg27553955 | 0.0031 | 3.69E-07 | 3.16E-04 | 2 | *KCNG3* | 1stExon | TRUE | Island |

“Probename”: CpG sites. “eAGE”: model coefficients of age extracted by elastic net regression, higher absolute value of the coefficient reflect the contribution of the CpG sites to the model is higher. “FDR”: false discovery rate. “CHR”: chromosome. “TRUE”: the CpG site can also be found on Illumina 450K BeadChip. “NA”: only on Illumina 850K BeadChip, not on the 450K BeadChip.
