# Supplementary material for: Age prediction of children and adolescents aged 6-17 years: an epigenome-wide analysis of DNA methylation
Source: Aging (Albany NY). 2018 May 12;10(5):1015–26. doi: 10.18632/aging.101445 (PMC5990383; doi:10.18632/aging.101445)
Supplement: Table S3 [file aging-10-101445-s004.docx]

| **Table S3. 83 predictive sites with annotation** | | | | | | | |
| --- | --- | --- | --- | --- | --- | --- | --- |
|  | Probename | eAGE | CHR | Gene Name | Gene Group | Methyl 450 Loci | Relation to CpG Island |
| 1 | cg00223245 | -0.8730 | 3 | *-* | - | TRUE | Open Sea |
| 2 | cg00303541 | 3.8646 | 3 | *GRM2* | 5'UTR | TRUE | Island |
| 3 | cg00329615 | -1.7760 | 3 | *IGSF11* | Body | TRUE | Open Sea |
| 4 | cg00443981 | 0.0915 | 17 | *C17orf64* | TSS200 | TRUE | S_Shore |
| 5 | cg00497086 | 10.0040 | 16 | *PRKCB* | Body | NA | Open Sea |
| 6 | cg00577449 | 0.8022 | 3 | *-* | - | TRUE | N_Shore |
| 7 | cg00589520 | -0.4777 | 7 | *-* | - | TRUE | N_Shore |
| 8 | cg01119503 | -0.8711 | 12 | *-* | - | NA | Open Sea |
| 9 | cg01231611 | -9.7149 | 1 | *REG4* | TSS200 | NA | Open Sea |
| 10 | cg01318665 | -0.3878 | 16 | *-* | - | NA | Open Sea |
| 11 | cg01949324 | -0.8261 | 2 | *-* | - | NA | Open Sea |
| 12 | cg02172773 | -1.1470 | 6 | *KIF25* | Body; | TRUE | N_Shelf |
| 13 | cg02307957 | 0.6810 | 3 | *-* | - | NA | Open Sea |
| 14 | cg02478540 | -2.0031 | 4 | *-* | - | NA | Open Sea |
| 15 | cg02772754 | 2.6007 | 22 | *MED15* | Body | NA | Open Sea |
| 16 | cg02822838 | -0.7880 | 6 | *TFEB* | TSS1500 | TRUE | S_Shore |
| 17 | cg02844688 | -0.7437 | 20 | *-* | - | NA | Open Sea |
| 18 | cg02872426 | -0.7179 | 6 | *DDO* | TSS200 | TRUE | Open Sea |
| 19 | cg03132729 | -1.3295 | 1 | *RAP1GAP* | Body | TRUE | N_Shore |
| 20 | cg03579624 | 3.5695 | 3 | *-* | - | TRUE | N_Shore |
| 21 | cg03653399 | -0.2678 | 8 | *SLC45A4* | Body | TRUE | S_Shore |
| 22 | cg03905236 | -0.3205 | 15 | *SH3GL3* | 5'UT | NA | S_Shore |
| 23 | cg03922748 | -1.1319 | 2 | *DNAJB2* | TSS1500 | TRUE | N_Shore |
| 24 | cg04436528 | -0.8767 | 8 | *BAI1* | Body | TRUE | N_Shore |
| 25 | cg04455146 | 0.4092 | 7 | *CADPS2* | Body | NA | Open Sea |
| 26 | cg04925748 | 0.0094 | 2 | *ABI2* | 5'UTR | NA | S_Shore |
| 27 | cg04955914 | -3.5037 | 2 | *C2orf24* | Body | TRUE | N_Shore |
| 28 | cg05596756 | -1.2892 | 12 | *FAM113B* | 5'UTR | TRUE | Open Sea |
| 29 | cg05613083 | 0.2838 | 16 | *KLHDC4* | Body | TRUE | N_Shelf |
| 30 | cg06072257 | -8.5780 | 1 | *-* | - | NA | Open Sea |
| 31 | cg06094762 | -0.7441 | 11 | *LDLRAD3* | 5'UTR | NA | Open Sea |
| 32 | cg06269443 | -0.5934 | 18 | *LINC00907* | Body | NA | Open Sea |
| 33 | cg06711259 | -4.0322 | 22 | *JOSD1* | 1stExon | TRUE | N_Shore |
| 34 | cg07219494 | -2.4714 | 5 | *-* | - | TRUE | S_Shelf |
| 35 | cg07382691 | -1.6851 | 2 | *LHCGR* | TSS1500 | NA | S_Shore |
| 36 | cg07465899 | -2.0805 | 4 | *-* | - | TRUE | N_Shore |
| 37 | cg07553761 | 0.6632 | 3 | *TRIM59* | TSS1500 | TRUE | Island |
| 38 | cg07662935 | -1.4580 | 1 | *CAMTA1* | Body | NA | Open Sea |
| 39 | cg07976733 | -0.2922 | 17 | *-* | - | NA | Open Sea |
| 40 | cg08349142 | 0.2318 | 1 | *-* | - | NA | Open Sea |
| 41 | cg08516641 | -0.4430 | 10 | *PTPRE* | 5'UTR | NA | Open Sea |
| 42 | cg09116468 | 1.3689 | 8 | *FAM135B* | 5'UTR | NA | Open Sea |
| 43 | cg09124496 | -0.9307 | 7 | *LOC285954* | Body | TRUE | Open Sea |
| 44 | cg09310092 | -1.6075 | 19 | *SCN1B* | Body | TRUE | N_Shelf |
| 45 | cg09461021 | -1.9820 | 22 | *-* | - | NA | Open Sea |
| 46 | cg10328813 | -0.0518 | 9 | *-* | - | NA | Open Sea |
| 47 | cg10816468 | -3.1004 | 6 | *-* | - | NA | Open Sea |
| 48 | cg10989288 | 1.4809 | 19 | *TMEM91* | Body | NA | S_Shore |
| 49 | cg11271492 | -1.9703 | 5 | *SLC4A9* | Body | NA | S_Shore |
| 50 | cg12642568 | -2.3633 | 1 | *CALML6* | 5'UTR | NA | N_Shelf |
| 51 | cg13274149 | 2.3751 | 9 | *C9orf167* | 3'UTR | TRUE | Island |
| 52 | cg13612317 | -2.1116 | 10 | *KIF5B* | TSS1500 | TRUE | S_Shore |
| 53 | cg13993467 | -2.8597 | 3 | *CNTN4* | Body | NA | Open Sea |
| 54 | cg14112935 | 0.2490 | 16 | *SLC6A2* | TSS200 | NA | N_Shore |
| 55 | cg14176099 | -0.0971 | 1 | *TDRD10* | TSS1500 | NA | N_Shore |
| 56 | cg14190522 | -1.7517 | 9 | *DAB2IP* | Body | TRUE | Open Sea |
| 57 | cg14584292 | -0.2336 | 11 | *MIR100HG* | Body | NA | Open Sea |
| 58 | cg15308037 | -1.9034 | 2 | *NPAS2* | Body | NA | Open Sea |
| 59 | cg16119613 | -2.0018 | 12 | *-* | - | TRUE | N_Shelf |
| 60 | cg16202624 | -1.3384 | 6 | *AIM1* | Body | TRUE | Island |
| 61 | cg16746462 | -1.0752 | 5 | *LNPEP* | TSS1500 | TRUE | Island |
| 62 | cg16867657 | 1.5653 | 6 | *ELOVL2* | TSS1500 | TRUE | Island |
| 63 | cg16902294 | 1.0071 | 4 | *LRAT* | TSS200 | NA | Island |
| 64 | cg17238334 | -1.0219 | 5 | *LOC102477328* | Body | NA | Open Sea |
| 65 | cg17723057 | -0.1222 | 6 | *-* | - | NA | Open Sea |
| 66 | cg18114755 | -0.0473 | 8 | *-* | - | NA | Open Sea |
| 67 | cg18219522 | -1.0433 | 11 | *MAML2* | Body | TRUE | Open Sea |
| 68 | cg18311495 | -0.1130 | 15 | *-* | - | NA | Open Sea |
| 69 | cg18488521 | 1.5640 | 18 | *-* | - | NA | Open Sea |
| 70 | cg18597220 | 1.5970 | 14 | *NRXN3* | Body | TRUE | Open Sea |
| 71 | cg21192606 | -1.6143 | 12 | *-* | - | NA | Open Sea |
| 72 | cg21242642 | 6.2130 | 1 | *-* | - | NA | Open Sea |
| 73 | cg22029239 | -1.2812 | 13 | *-* | - | NA | S_Shelf |
| 74 | cg22493216 | -0.6306 | 10 | *PLEKHS1* | Body | NA | Open Sea |
| 75 | cg22603452 | -1.9846 | 1 | *FCER1A* | 5'UTR | NA | Open Sea |
| 76 | cg23204757 | -1.2799 | 21 | *-* | - | NA | Open Sea |
| 77 | cg23565569 | -1.2712 | 7 | *CPED1* | 5'UTR | NA | Open Sea |
| 78 | cg23656415 | -0.0271 | 3 | *-* | - | NA | Open Sea |
| 79 | cg23749518 | -0.0204 | 12 | *-* | - | NA | Open Sea |
| 80 | cg24382249 | -0.1185 | 15 | *-* | - | TRUE | N_Shore |
| 81 | cg24388008 | -2.7052 | 12 | *-* | - | NA | Open Sea |
| 82 | cg27367871 | -1.6092 | 2 | *CXCR7* | TSS1500 | TRUE | S_Shore |
| 83 | cg27406001 | -3.4879 | 10 | *-* | - | NA | Open Sea |

“Probename”: CpG sites. “eAGE”: model coefficients of age extracted by elastic net regression, higher absolute value of the coefficient reflect the contribution of the CpG sites to the model is higher. “CHR”: chromosome. “TRUE”: the CpG site can also be found on Illumina 450K BeadChip. “NA”: only on Illumina 850K BeadChip, not on the 450K BeadChip.
