# Supplementary material for: Age prediction of children and adolescents aged 6-17 years: an epigenome-wide analysis of DNA methylation
Source: Aging (Albany NY). 2018 May 12;10(5):1015–26. doi: 10.18632/aging.101445 (PMC5990383; doi:10.18632/aging.101445)
Supplement: Table S4 [file aging-10-101445-s005.docx]

**Table S4. The gene ontology enrichment analysis results of 83 age predicted CpG sites** **(*P*<10^-3^)**

| GO Term | Description | P-value | FDR | N | B | n | b | Genes |
| --- | --- | --- | --- | --- | --- | --- | --- | --- |
| GO:0007411 | axon guidance | 2.15E-04 | 1.00E+00 | 18643 | 223 | 46 | 5 | *CNTN4, SCN1B， NRXN3，RAP1GAP, KIF5B* |
| GO:0097485 | neuron projection guidance | 2.24E-04 | 1.00E+00 | 18643 | 225 | 46 | 5 | *CNTN4, SCN1B, NRXN3, RAP1GAP, KIF5B* |
| GO:0007158 | neuron cell-cell adhesion | 6.99E-04 | 1.00E+00 | 18643 | 16 | 46 | 2 | *CNTN4, NRXN3* |

We entered 66,137 gene terms. A total of 63,318 genes were recognized by the system. Only 18,643 of these genes are associated with a GO term. 'FDR q-value' is the correction of the p-value for multiple testing using the Benjamini and Hochberg (1995) method. Enrichment (N, B, n, b) is defined as follows: N - is the total number of genes; B - is the total number of genes associated with a specific GO term; n - is the number of genes in the top of the user's input list or in the target set when appropriate; b - is the number of genes in the intersection.
